# Uncovering placemaking needs with(in) a kindergarten community: a cross-disciplinary approach to participatory design

**DOI:** 10.3389/fpsyg.2023.1126276

**Published:** 2023-06-20

**Authors:** Eléni Economidou, Nathalie Gerner, Christina Pichler, Arnulf Hartl, Christopher Frauenberger

**Affiliations:** ^1^Human-Computer Interaction Division, Department of Artificial Intelligence and Human Interfaces, Paris Lodron University of Salzburg, Salzburg, Austria; ^2^Institute of Ecomedicine, Paracelsus Medical University, Salzburg, Austria

**Keywords:** participatory design, early childhood education and care, teacher workplace experiences, kindergarten children impressions, placemaking needs, supportive built environment

## Abstract

**Introduction:**

The design of early childhood education and care facilities faces the double challenge of creating a stimulating environment for young children and a supportive workplace for staff. The existing body of research suggests that placemaking strategies serve both requirements. A promising approach to meet placemaking needs is the participation of future occupants in the building design.

**Methods:**

We pursued a participatory design study with the community of an Austrian kindergarten aiming to inform the future building renovation. We combined novel cultural fiction probes methods with conventional inquiry methods to gather information from children and teachers about their experience of the built environment. Using thematic and content analyzes we explored placemaking needs from different epistemic perspectives and converged findings through iterative exchange.

**Results:**

Returns of children and teachers were interconnected and complementary. From a design-oriented perspective, children’s experience of place was relatable to spatial, temporo-spatial, and acoustic qualities as well as control needs. From a human-centered perspective, teachers’ experience of place was relatable to the needs of feeling embedded, protected, enacted, and socially connected. The converged findings revealed dynamic placemaking processes involving the elements of space, time, and control at different levels.

**Discussion:**

Cross-disciplinary collaboration and research consolidation brought forth valuable insights on supportive structures for both children and teachers, facilitated timely knowledge transfer, and converted into design solutions that foster enacted placemaking. Albeit general transferability is limited, findings are interpretable within a solid framework of existing theories, concepts and evidence.

## 1. Introduction

The design of community buildings calls upon careful consideration and coordination of multiple coexisting requirements to adequately meet the common purpose and all users’ needs under one roof. Beyond general demands on the physical setting, basic functioning, and geographic context, such requirements may be colored by an organizational background, cultural aspects, community values or individual member’s needs. Inhomogeneous communities as typically present in the early childhood education and care (ECEC) setting can exhibit complex, sometimes even conflicting needs, and thus, challenge the design of an overall supportive and inclusive environment.

First and foremost, ECEC plays a decisive role in children’s development including their early education, social experiences, and well-being. Children spend a significant portion of their formative years in ECEC environments and as such they influence their future accomplishments (Şahi̇n and Dostoğlu, 2012), their perception of the world and their place in it (Dudek, 2005, p. 115). Experientially poor or unstimulating environments may hinder children’s development, and education (Moore, 1987; Read et al., 1999; Day and Midbjer, 2007).

At the same time, the quality of ECEC is reliant upon a strong staff capacity. Findings suggest that teachers’ subjective well-being at work as indicated by perceived workplace stress can affect the quality of their practices with children, and consequently, children’s development (Whitaker et al., 2015). Recently, health-related issues, both physical and psychological, have been identified as one of the top reasons for ECEC staff turnover, internationally (OECD, 2019, 2021) which is adding to the urgency of a critical reflection of the ECEC setting as a work environment. According to the job-demand-resources model, job resources counteract high job demands and work-related stressors, and thus, can act as burnout buffers (Bakker and Demerouti, 2007) and elevate teachers’ work engagement (Bakker et al., 2007). In ECEC settings, aside from financial and social factors, the physical quality of the built environment, such as adequate scales and proportions, has been addressed as an important resource for staff’s professional well-being (Kwon et al., 2021).

This background endorses a comprehensive user-centered perspective on ECEC building design that equally considers two main objectives, i.e., creating a stimulating environment for young children and a supportive workplace for the staff.

In theory, different education concepts require different environmental conditions, as in the case of the Reggio Emilia approach, where the environment is seen as the third teacher (Strong-Wilson and Ellis, 2007). There have been various efforts to identify design elements that make environments appealing and valuable for children (Moore, 1987; Olds, 2001; Dudek, 2005). One approach follows the idea of home-like environments stimulating children’s sense of place (Read, 2007). Sense of place describes a particular experience a person attributes to a specific spatial setting, which may elicit a feeling of emotional attachment and add meaning to the experienced space (Gustafson, 2001). As such, sense of place is considered as “the desired result of placemaking” (Aravot, 2002, p. 202), a people-centered approach rooted in urban design. Hitherto, there is no literature on placemaking efforts that targets ameliorating the work conditions of ECEC staff. Research in the office setting, however, offers first insights into the high potential of place experience at work suggesting positive correlations with job satisfaction and motivation (Miller et al., 2001). Such findings support the notion that spatial qualities that elicit a sense of place can be considered as a valuable job resource.

Spatial solutions attuned to both teachers’ and children’s needs are essential when aiming to create a supportive ECEC environment. Actively involving children and teachers in the building design process through a participatory design approach (Dudek, 2005; Clark, 2007) supports architects in creating not only tailored, but also more informed and sustainable solutions (Sanoff, 2015). Such endeavors may reap other benefits such as raising the faculty morale and school and community pride, as well as establishing a sense of ownership for both the process and project in question (Sanoff, 2015). Conversely, findings suggest that ill-designed educational environments can hinder both the mental and physiological well-being (Leather et al., 2003; Branco et al., 2020; Lavdas and Salingaros, 2021).

Within participatory design, children and teachers are no longer seen as passive users of their spaces but are rather considered as active participants with their own contextual needs and experiences and as experts of the places they inhabit. Mere observation is no longer considered to understand a community’s needs, especially when it comes to children; one must listen to them (Iltus and Hart, 1994). As supported by Sanoff (2001), it is in people’s democratic right to take part in decision-making processes that directly affect them, making in turn these processes more effective.

A typical participatory building design process of a new school setting as presented by Rigolon (2011) begins by *recognizing and framing a problem* related to the spatial situation, followed by the *briefing and planning phase* where architects and designers as project leads hold meetings with the stakeholders to set expectations, define the budget, create an agenda with expectations and techniques, and plan the next steps in the design process. Design activities are preceded by an *analysis of the status quo* at the given context and the users’ needs through methods such as interviews and questionnaires, as well as drawings or photographs when involving children. This *needs analysis* provides starting points to debate on for the design phase that follows. In the *design phase*, the community is invited to contribute ideas through creative activities such as design charrettes (Sutton and Kemp, 2006), model making and drawing. The *construction phase* comes after the refinement of a selected idea. In smaller projects such as garden creations or other small spatial modifications the involved community can take part in the construction phase, yet in the case of building construction the phase lies within the responsibility of the building construction professionals. When the construction is complete, a *final evaluation* occurs where the community is asked to reflect about the developed designs and contrast them to their initial aspirations. As a last step, Rigolon (2011) suggests leaving *space for ongoing modifications* so that spaces can be adapted based on the users’ changing needs. It is worth mentioning that in a participatory design, knowledge creation depends on practice, therefore its outcome cannot be entirely foreseen (Luck, 2018).

While the benefits of involving teachers and students in the building design process are recognized by the educational research (Könings and McKenney, 2017) and the educational and child psychology fields (Woolner, 2011) with multiple reported studies, the reports are rather scarce when it comes to involving young children in the design of their ECEC environments. The few studies on this topic do report promising findings (Rasmussen, 2004; Clark and Moss, 2011; Bakr et al., 2018; Beheiry and Gabr, 2021), however, they neglect to take into account and combine their findings with the teachers’ views and needs.

To bridge current research gaps, we deployed a multi-method participatory design approach involving children, teachers, and the management of a public kindergarten. In this article we report on the needs analysis phase of our participatory design process in which we conducted an explorative field study examining children’s and ECEC professionals’ learning and practice processes in their kindergarten reality. The objective of our study was the identification of spatial elements that are significant for children’s and ECEC professionals’ experience of place as well as needs and wishes relevant to their current built environment. The findings of the study should provide information to create innovative design solutions for a user-friendly and supportive kindergarten environment.

This study was conducted as part of a cross-disciplinary project, where researchers and architects closely collaborate with the users of a public kindergarten during the planning and design process of their future kindergarten building. Our two research teams consisted of researchers with backgrounds in the fields of human-computer interaction (HCI), architecture, early education, psychology, and medicine. The research aims to support stepwise decision-making in the building renovation process in the interest of the participating kindergarten community.

## 2. Materials and methods

The case study took place in two parts during May and June 2021 at an under-renovation kindergarten located in the south of the Federal State of Salzburg, Austria. Part A of the study was conducted by the team of Human-Computer Interaction of the University of Salzburg investigating the children’s experience of their kindergarten environment. Part B of the study was conducted by the team of Ecomedicine of the Paracelsus Medical University investigating the ECEC staff’s experience of their work environment. The participating kindergarten was recruited by the partnering company Salzburg Wohnbau GmbH, the municipal construction service provider responsible for the planning and construction of the renovation. All participating ECEC professionals and children’s parents were affiliated with the cooperating kindergarten. Beyond the playful character of children’s activities and the aim of improving the future work environment for the staff there was no incentive or compensation provided to participants. All participating staff and children’s parents signed an informed consent form which was approved by the local ethics board.

As research was conducted during the COVID-19 pandemic which came along with repeating periods of strictly restricted social contact, the study was planned to allow constant remote conduction. A large part of communication between researchers and cooperating stakeholders as well as the communication between researchers and the participating kindergarten community occurred via telephone calls, email exchanges, and online consortium meetings. Moreover, all data collection materials were prepared in a format that allowed non-physical contact with the researchers, but no changes in content were made.

**Figure 1:**
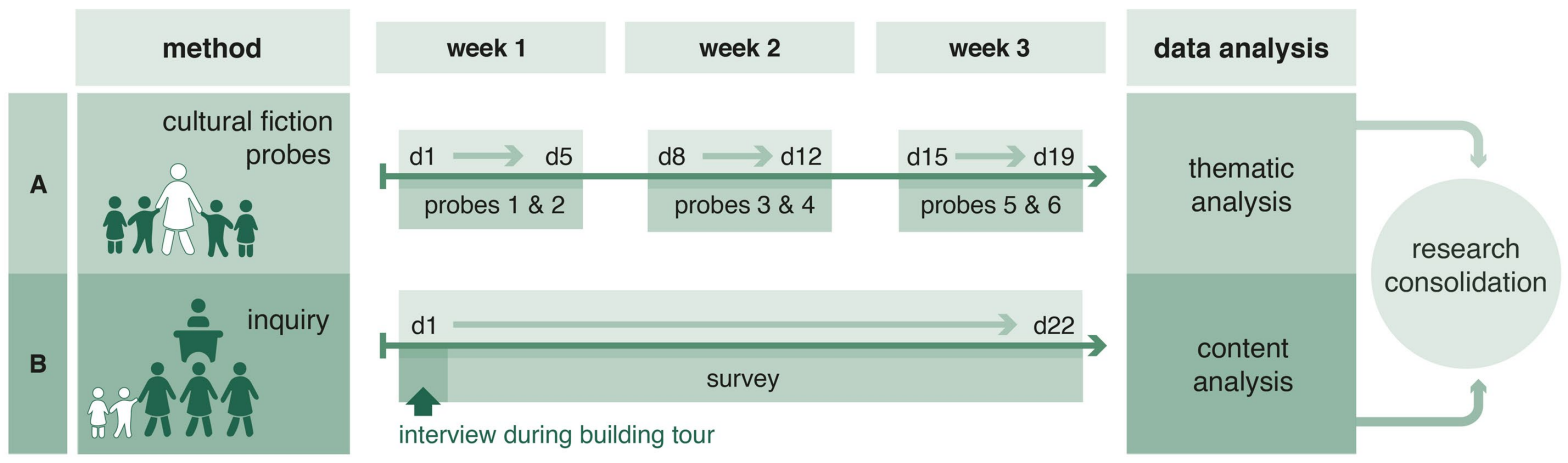
Study conduction with the participating kindergarten community in two independent parts followed by collaborative cross-disciplinary research consolidation. Part **A**: data collection with kindergarten children in three slots with two probing activities each (days 1-19) and analysis of cultural fiction probes. Part **B**: data collection with ECEC staff in two slots - interview with headmistress (day 1) and written survey with teachers (days 1-22) - and analysis of their building evaluation.

The study design, as shown in Figure 1, included five time slots within 3 weeks for data collection followed by two separate data analyzes of, respectively, children’s and ECEC professionals’ perspectives, and a final iterative research consolidation.

### 2.1. Data collection

#### 2.1.1. Cultural fiction probes with young children

For a total duration of 3 weeks, our team of four researchers pursued an explorative participatory design activity under the name cultural fiction probes with the children attending our case kindergarten. Our probes combined aspects by two established methods in the field of HCI, namely, cultural probes and fictional inquiry. Cultural probes is an exploratory qualitative research technique (Gaver et al., 1999) that enquires into the research context via means of packages containing disposable cameras, diaries or other self-logging materials, whereas fictional inquiry is a participatory design approach that circumvents socio-cultural structures by inviting participants to explore fictional scenarios or artifacts (Dindler and Iversen, 2007).

We opted for a combination of these two established approaches aiming to reduce the officialdom of social, cultural, and physical barriers such as age gaps and language, and confer agency and competency to children using methods and research instruments other than verbal and written communication. Our approach maintains a playful and fun character, combining probe activities wrapped around a fictional inquiry narrative. As other researchers reported (Wyeth and Diercke, 2006), we hypothesized that the proxy fictional story would allow us to bypass the reluctancy issue children face when asked to self-report on experiences over a time period. Rather than giving assignments introduced directly by our research team, the fictional plot of alien children seeking advice on their physical environment weaved the activities together and engaged the children in elaborate missions, fueled by their sense of altruism in helping the heroes of the narrative. Our approach, much like its predecessors, required active involvement from the children to surface inspirational yet incomplete biographical accounts of unexpected or invisible issues, emotions, hopes and values regarding their everyday lives and practices and their space of opportunity. Through our cultural fiction probes, children become co-producers of research by collecting their own experiential evidence through photo elicitation, informal interviews and artifact making.

The cultural fiction probes material and fictional narrative was developed iteratively during multiple internal workshops among three experts from the HCI and ECEC research team using whiteboard and paper sketching tools. The result of these creative workshops were six different probing activities that explored the modalities of touch, hearing, vision, scale, and perception of time structures.

We opted to present the narrative around the activities in audio format using a customized communicating box; a carton box which broadcasted pre-recorded voice messages through an iPod and a portable speaker. The probing material consisted of digital cameras for children, voice recorders, color flashlights, moldable materials, coloring markers, and large sheets of paper which were placed in separate material boxes. The activities consisted of (i) photo elicitation, (ii) a light riddle, (iii) material samples, (iv) capturing spatial scale and proportions, (v) a sound inquiry, and (vi) a cease of play inquiry. More concretely, the children were asked to capture favorite play activities and spaces through photo elicitation, enquired on sound conditions via self-recorded audio, examined materiality through instructing the creation of materials that provided superpower capabilities, explored children’s scale in relation to their environment by means of body outlines, and investigated play interruption by interviewing children on having to pause their play activities.

The transcription of the narrative as well as the instruction to the teachers can be found in the Supplementary material S1. A detailed description of the procedure including pictures of the whiteboards, the developed concept behind the elaborated probing activities, pictures of the constructed communicating box and probes materials boxes as well as a detailed description of the different probing activities are available in the Supplementary material S2.

The week before the probe study took place, a member of our research team delivered the probe packages to the kindergarten’s headmistress. The participating teachers received instructions to perform data collection with the children in their groups. The teachers integrated the probe activities within their everyday practice with the children. Children were free to contribute to data collection according to their situational preference. The children completed two activities per week under the supervision and with the help of the respective teachers. Following the completion of the probes study, the packages were collected and delivered to our research facilities for data analyzes.

#### 2.1.2. Building evaluation with ECEC staff

We consulted the participating staff about their work experience in the existing kindergarten building. Initially, one researcher visited the kindergarten for a tour around and inside the facilities with the kindergarten’s headmistress. At the time of the tour, the kindergarten area was minimally occupied. During the tour, the researcher conducted a semi-structured interview with the headmistress who was initially briefed. The interview guide contained two core questions: (1) “Which spatial features are beneficial for teachers and children, why?,” and (2) “Which spatial features are hindering for teachers and children, why?”. If required, a list of typical spatial features (e.g., ambient conditions, furniture etc.) was provided as an impulse. The interviewer documented the headmistress’s responses in the protocol form and took a photographic record of each described area. The headmistress approved or amended the protocol after each room or area evaluation.

Furthermore, we conducted a written survey with all teachers of the cooperating kindergarten. On the same day the building tour was performed, we handed over the paper-and-pencil forms to the kindergarten staff. The survey covered the two core questions from the interview guide with examples of typical spatial features. The adapted interview protocol form was used as a response form. Teachers were instructed to assess each area they use in the kindergarten. It was open to the participants when, under which conditions and to which extent they responded to the open questions. After 3 weeks, a researcher picked up the completed survey forms from the kindergarten.

The interview guide, the corresponding protocol form, and the parallel survey form were developed in a single internal workshop on the requirements to ECEC environments. The materials used for data collection, the photographic record of the building tour, and the anonymized raw data from the building evaluation are available in the Supplementary materials S3–S6.

### 2.2. Data analysis

#### 2.2.1. Thematic analysis of children’s returns

The returns from the probes packages consisted of drawings, photos of children’s favorite toys and spaces, materials created by the children, voice recordings of interviews regarding sound levels, outlines of their bodies in their favorite space, and children’s quotes and reactions captured in text form by the teachers. Each of the received voice recordings and handwritten notes was manually transcribed and split into sentences. When having a first look at the collective returns, we realized that some of children’s artifacts were not analyzable and interpretable in relation to the kindergarten’s spatial qualities. We discarded these returns from further analysis which mainly concerned the activities (ii) and (iii).

We applied reflexive thematic analysis (Braun and Clarke, 2022) due to the theoretically independent flexibility it provides in analyzing qualitative data of multiple media forms and its suitability for our experiential qualitative approach. Based on (Braun and Clarke, 2022, p. 159) we focused on how children act, feel and think about their kindergarten environment, their overall experience and how and what they adhere meaning to as articulated and showcased by them. The first phase of the analysis, data familiarization and initial theme generation, took place during a 3-h group discussion with three team members (one senior and two junior researchers). As a second phase, every data segment (photographs, quotes, and reactions) was entered into a spreadsheet file which resulted in 190 unique entries. One team member inductively generated descriptive codes for each data entry and sorted the codes into the predefined themes which were further refined. Stand-alone probes that did not fit in context were not taken into consideration. The initial coding round culminated in four unique themes, of which were either merged with other themes or were disregarded as less relevant to our research questions. Following the inductive coding, there was a group discussion where the main themes were agreed upon. Iteratively, group discussions and clustering brought forth the resulting four themes: placemaking, control of the environment, noise, and time-making. The thematic analysis spreadsheet can be found in the Supplementary material S7.

#### 2.2.2. Content analysis of ECEC staff’s responses

Complementary, we focused on the ECEC staff’s relationship to their work environment and the spatial qualities contributing to their emotional well-being and experience of place. We were interested in both their positive and negative impressions which formed the core of our inquiry. To this end, our analysis based on the interpretation of experience of place as a temporal subjective feeling of attachment to a dedicated environment which can manifest in both a positive or negative quality depending on the scope of expectations and needs met when acting in and interacting with this environment. Aiming to uncover the needs connected to the experience of place we performed a qualitative content analysis (Mayring, 2021). All recorded responses were transferred to a spreadsheet file, sorted by evaluation format, participant, response count, type of spatial area, and positive or negative quality of content. The inductive theme and category development based on the more elaborate interview content. The emerging codebook underwent formative and summative reliability checks before the agenda was applied to the entire dataset.

We explored the staff’s responses for their needs regarding the built environment as manifested in positive or negative impressions of their workplace. To this end, we considered each response with at least one differentiable semantic content related to (a) the perception of, (b) action in, and (c) interaction with the built environment as an eligible unit of analysis. Each semantic content was analyzed in context of the described functional area. As one quality of space can serve different environmental needs, we allowed for multiple category assignments. We created response duplicates for each unit assignable to different categories, and weighting duplicates for each differentiable semantic content within a response assignable to the same category. We completed the analysis with a final summative reliability check and quantified the findings by calculating the sum of units assigned to the respective categories and themes.

The coding was performed by the interviewing researcher who joined the kindergarten building tour. To prevent loss of contextual information, the photographic record subserved the better understanding and close interpretation of the response contents. All analyzes and reliability checks were performed by the same researcher with intervals of at least 1 week in between each step. The final version of the codebook is available in the Supplementary material S8.

#### 2.2.3. Iterative research consolidation

With special regard to the participatory nature of the study, we presented the separate preliminary findings to the project consortium (i.e., architects involved in the building design, the kindergarten management, a representative of the kindergarten teachers, and the respective partnering research team) to open the research for feedback and to ensure a practicable knowledge transfer. These feedback session contributed to further elaboration and gave reason for research consolidation.

For a holistic picture of both children’s and ECEC professionals’ perspectives on the built environment, we performed a joint analysis based on the respective findings from separate analyzes. For this, the analysts of both research teams converged their different research approaches, methods, and findings in common iterated workshops discussing parallels and differences between children’s and staff’s needs and developing models for a common framework in a bottom-up manner. These workshops included discussions on epistemological perspectives, juxtaposition of the separate findings, and the use of digital canvas tools. In a final analysis both findings were integrated into the evolved framework for common interpretation.

## 3. Findings

### 3.1. Placemaking needs derived from children’s returns

For the data collection from children, we applied a purposive sampling strategy, involving the entire kindergarten children community population of 86 three-to-six-year-old children. Within the six probing activities the participating children totaled to a mean of 75.90 (SD = 6.01). During data collection, no changes to participant numbers were observed. Our thematic analysis led us to four themes that were prominent throughout the entire dataset. Notably, these themes are interconnected and cannot be interpreted in isolation.

#### 3.1.1. Spatial qualities and placemaking

The notion of space as a physical milieu with specific characteristics was mainly apparent in the photographic material. We noticed that the space’s physical qualities (e.g., layout, scale, proportion, furniture positioning, room orientation, materiality, color) dictated the type of activities that could take place at specific areas as they allowed for different action possibilities. A few of the photographs depicted open space where furniture and other objects were placed or pushed to the edges of the room, thus affording a series of bodily-based activities such as walking, running, jumping, sitting, and lying on the floor. The floor seemed to be a central surface where activity takes place. Scale and proportions were identified as an important spatial quality for children. Construction materials of handheld and full-body scale as well as living room and kitchen furniture - proportional to children’s height and size - were present in many photographs indicating children’s affinity for pretend play and role-play activities. Other photographs depict socio-spatial arrangements such as tables and chair arrangements or painted circles on floor areas, both affording sitting and performing group activities such as crafting, storytelling, presenting, and listening. Children- and adult-fabricated places of refuge were favored. The pictured refuge nests allowed occlusion from the public and peeping out through openings and translucent fabrics, preserving one’s privacy. Organizational structures such as cabinets and drawer-adorned furniture seemed to capture children’s attention. Upon close scrutiny, these structures served to classify and store toys based on category or organize documentation of each child’s personal progress and creations. Almost all the collected photographs depict brightly lit environments, with large fenestration allowing in sunlight either directly or indirectly, with views toward the mountains. In terms of materiality, most of the surfaces, furnishings and refuge areas in the photographic material were made of timber. Most floors were tiled while the playing area is carpeted in gray color.

#### 3.1.2. Time-making

Different probing activities revealed children’s temporal structures. We observed children’s practices of organizing their play activities, their aversion to interruptions, and their liking for continuity as well as an affinity for organization and storing systems.

In some of the received photographs, elaborate and unruly three-dimensional building block compositions were placed in a dedicated play area. The area was pronounced by a carpet surrounded by wooden benches which doubled as storage space. In other photographs, construction blocks were stored in the wooden benches, leaving the space tidy and uncluttered.

Our question toward their preference of ending a play activity and tidying up their play constructions was met with great opposition, with children articulating the unfairness of the situation and commenting on their perceived malicious nature of the fictional characters of the activity narrative:

Child 1: *“That’s totally mean actually, because when I do something cool, I have to ruin that on purpose.”*

Child 2: *“I think it’s mean and unfair.”*

Child 3: *“[the] voice was cute before”* [Teacher’s note: *“Now the child does not like the voice”*].

Child 4: *“You get interrupted and then you do not know what you wanted to do.”*

#### 3.1.3. Control of the environment

One of the surfaced themes from the probes was children’s agency in and wish for controlling their environment. In terms of physical surroundings, this theme manifested in photographs depicting child-constructed structures such as building blocks in either hand-held size or in human-scale. The structures had the form of *ad-hoc* three-dimensional cardboard constructions and self-made tent-like dens. The cardboard structures were placed in an otherwise empty corridor floor and children seemed to jump from one to another in the received photographs. The dens in the gym hall were fabricated by putting together tent-resembling structures made of textile and spools of thick metal wire. Through these architectures, children obtained a sense of privacy and personal space, and they marked a territory through visible or invisible boundaries. Aside from the self-made architectures, children captured adult-fabricated nest-like areas for refuge and solitude, either constructed by teachers or prefabricated. These areas appeared in the form of raised floor platforms, curtain separated spaces, tent-like textile structures or other areas with comfortable furniture without any visible border or threshold. All of the dens were equipped with soft furnishings; a large mattress on the floor, pillows and plush toys, and decorative fabrics in an array of patterns and colors. One of the nests included children’s books in a wooden crate.

In terms of controlling the spatial environment, aspects that seemed of importance were light, sound, and temperature; aspects usually associated with ambience. Some photographs captured indoor and outdoor environments that were brightly lit with direct or indirect sunlight, indicating a preference for this type of light quality. Children indicated their preference for quiet environments and exerted control over the sound aspect by changing environments.

Regarding temperature, children periphrastically expressed their wish for a cooler environment during warm days:

Teacher: *“What have you created?”*

Child: *“There’s a straw with a sheet of paper and I glued it together, and if it’s too hot for you, you can blow into the straw and it will cool you down again.”*

Teacher: *“What can it do?”*

Child: *“It’s only for the summer.”*

#### 3.1.4. Noise mitigation

Numerous audio recordings circulated around the topic of sound, especially noise and loudness. Children explicitly voiced their aversion to loud sounds (e.g., shouting, screaming, screeching) and their preference toward quiet areas. A space associated with tranquility and silence was the reading corner while a space that was perceived as loud was the gym hall:

Child: *“Hello, I think the sound of the reading corner is really cool, because it’s so quiet and….”*

Teacher: *“Do you want to show another sound?”*

Child: *“I think so.”*

Teacher: *“What do you think?”*

Child: *“Something loud. Yes, something else loud.”*

Teacher: *“Where is it always particularly loud in our kindergarten?*

Child: *“In the gym.”*

Teacher: *“Then you can still record it. And is it good for your ears when it’s so loud or not?”*

Child: *“No. I’ll show you. (walks towards somewhere loud, presumably the gym).”*

When probed about their favorite sounds, children said they enjoyed music-related sounds (e.g., clapping, singing), crafting sounds (e.g., cutting paper, ironing beads), animal sounds (e.g., horses, cats, dogs) and quiet or low volume sounds. Some children indicated a dislike to technology and machine-related sounds (e.g., ringing telephones, car engines) while others seemed to enjoy them.

### 3.2. Placemaking needs derived from ECEC staff’s building evaluation

Aside from the interview with the headmistress, all of the 11 teachers actively employed at the case kindergarten participated in the survey. All participants were women. Their work experience in the kindergarten building ranged from 10 months up to 10 years (*M* = 3.71, SD = 3.08). Fifty percent of them were employed full-time.

**Figure 2:**
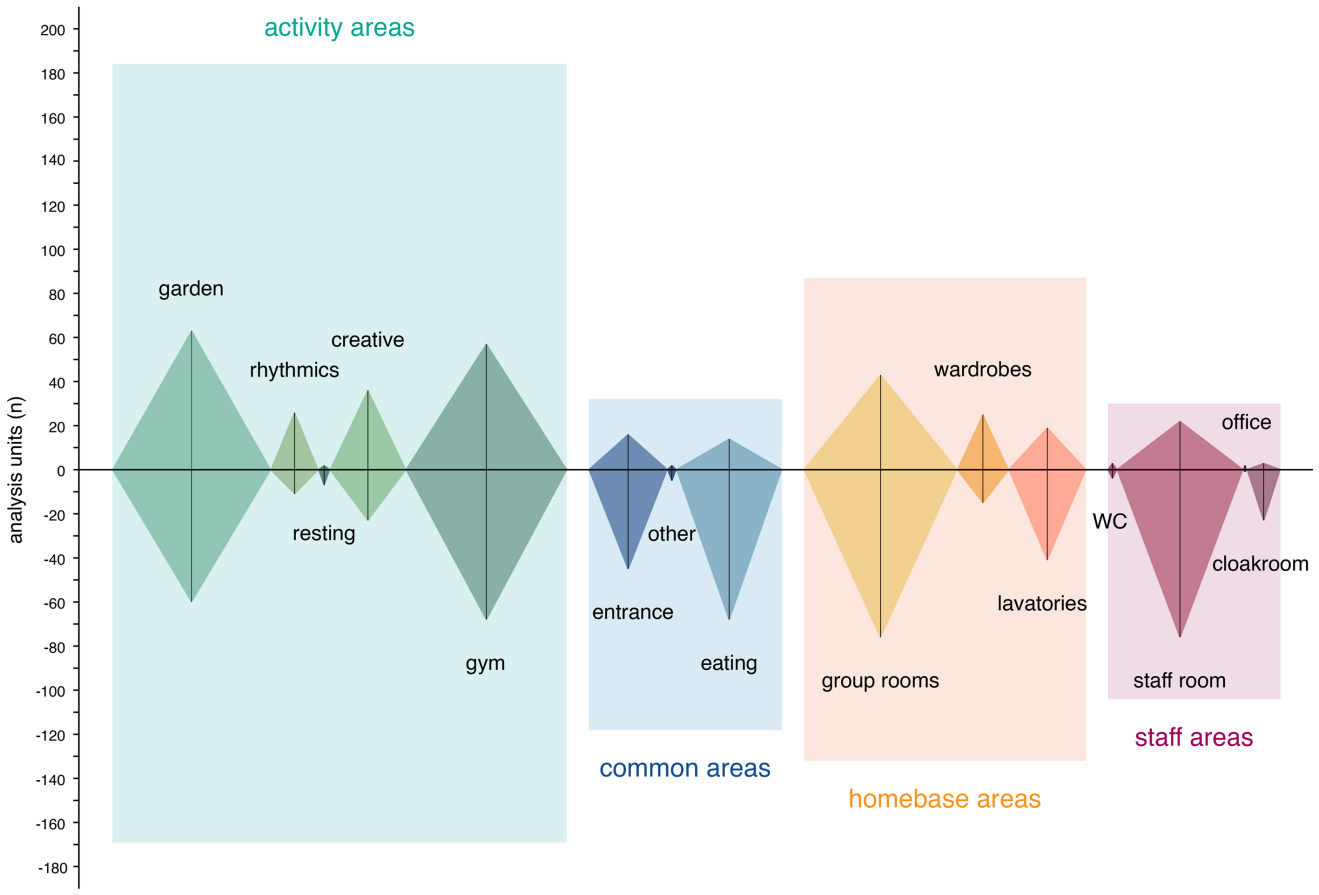
Distribution of analysis units across functional areas.

**Figure 3:**
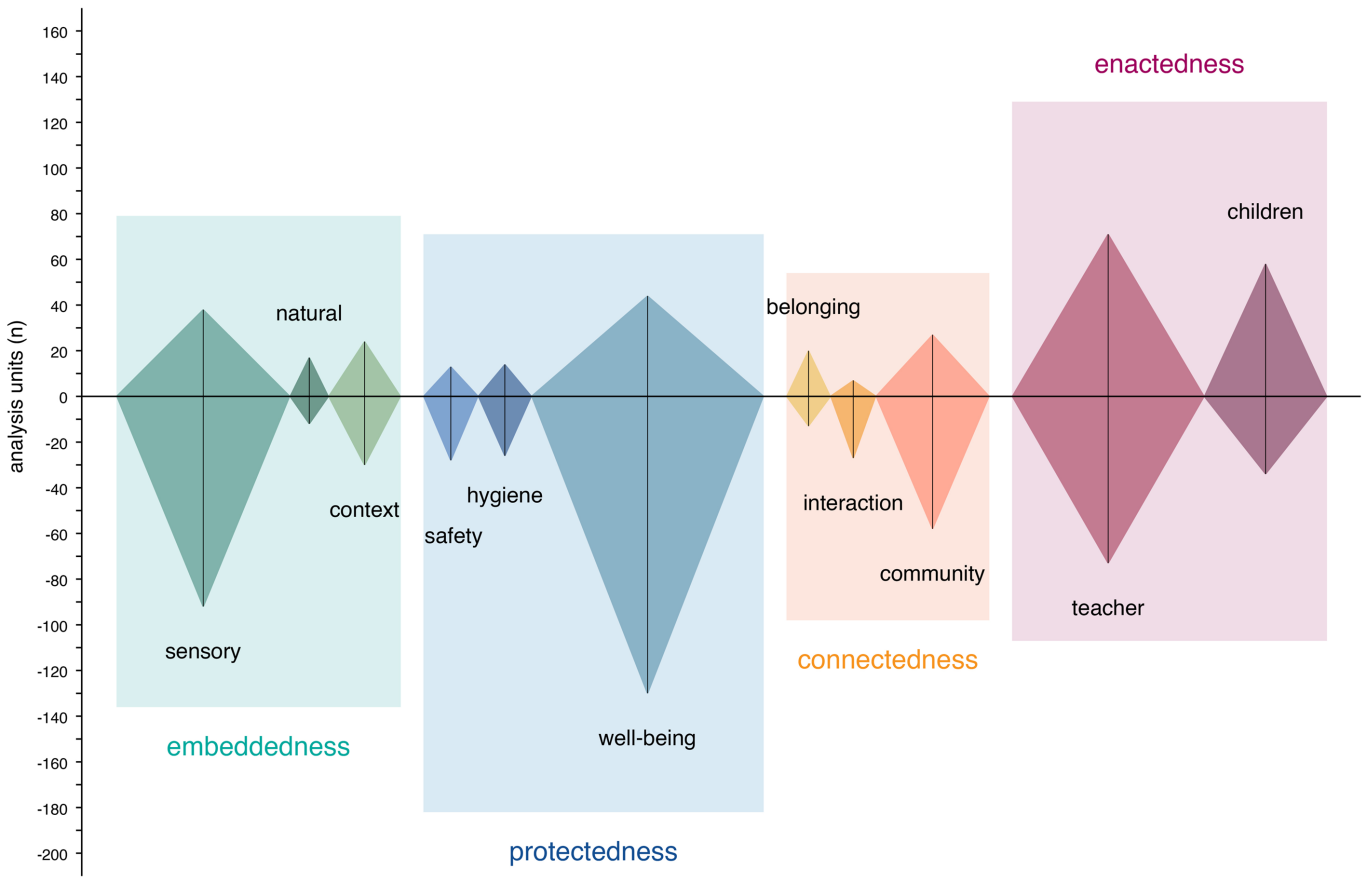
Distribution of analysis units across themes and categories.

We analyzed a total of 420 responses (111 interview responses) resulting in 856 units of analysis (403 response duplicates, 33 weighting duplicates). No responses were excluded. Negatively connotated content dominated (61.10%) across all units. Participants described 15 functional areas as summarized in Figure 2: five types of shared activity areas (353/856 units), three types of common areas (150/856 units), three types of exclusive homebase areas (219/856 units), and four types of staff areas (134/856 units). Qualitative content analysis revealed four themes and 11 categories reflecting the ECEC staff’s impressions of the built environment as summarized in Figure 3. Themes describe their environmental needs associated with experience of place. Categories specify contributing qualities. Exhibiting contextual overlaps, these themes and categories appeared to be complementary and should not be interpreted isolated from each other.

#### 3.2.1. Embeddedness

A broad set of responses adding up the ECEC staff’s impressions of locality, spatial orientation, and the affective atmosphere converged into the theme embeddedness. We discovered this need of being ingrained with the surrounding environment in three content categories: One category reflected the relationship to the contextual environment. This included spatial qualities that affected the experience of territory (e.g., outdoor areas, boundaries), neighborhood (e.g., infrastructure, traffic), and of transition areas between them (e.g., access points, arrival and departure areas, drop-off and pick-up areas for parents). Another category captured the relationship to the natural environment. Participants described their experience of nature indoors (e.g., indoor plants, terrarium, timber material) and mentioned their preference for nature view (e.g., green space, trees). A strong focus was on providing opportunities for children to interact with nature (e.g., snack garden, stone constructions, water-mud-areas, climbing tree trunks, insect terrarium). The third category encapsulates the sensory environment perception. This involved ambient environmental conditions (e.g., lighting, temperature, air quality, acoustic environment) as well as visual anchors and references that support orientation (e.g., color coding, shapes, symbols, window views).

The embeddedness theme covered 25.12% of total units. Negative expressions dominated (63.26%), most prominently related to sensory experiences. Deficits mainly concerned acoustic and climatic inconveniences and insufficient shading options. The ECEC staff aimed to adjust ambient conditions according to situational needs pointing at the importance of more adaptive ambience solutions.

#### 3.2.2. Protectedness

A central concern of the ECEC staff was a safe environment leading to the theme protectedness. We identified three categories exhibiting different facets of safety needs in the kindergarten. First, participants described environmental aspects that affect physical integrity; particularly structural conditions that contribute to risk prevention (e.g., building maintenance, weather protection, appropriate scale of interior) and support the supervision of children (e.g., accessibility and observability of children’s location for teachers, restricted accessibility for children). Second, participants’ perception of a safe place was connected to hygiene conditions. This concerned organizational structures that ensured cleanliness (e.g., wardrobe storage for shoes, laundry supply, easy accessibility of lavatories) and disease prevention (e.g., sanitary aspects in eating areas, separate or alternative spaces for social distancing during epidemic times). Third, participants addressed environmental impacts on general well-being. Aside from spatial qualities that support physical health (e.g., ergonomic furniture, space and equipment for physical activity), and stress prevention (e.g., children’s places of refuge, separate staff areas for breaks and exchange, calm eating areas, enough equipment, efficiency of space), this included ambient conditions that contribute to health prevention (e.g., noise moderation, appropriate indoor climate, lighting).

We found 29.56% of total units linked to the protectedness theme. The theme exhibited the strongest negative expression across the analysis, covering 71.94% of the assigned units. Aside from structural deficiencies, restrictions in space, equipment and control contributed to unfavorable experiences. While the ECEC professionals missed structures that support a smooth workflow and recreational breaks, they stressed their continuous efforts to provide a protected environment for children. Thus, more protective structures may contribute to the experience of a safe and healthy place.

#### 3.2.3. Connectedness

The ECEC staff’s perception of social spaces and associated social dynamics gave rise to the theme connectedness. We identified three categories of content reflecting the need of interacting and being connected with others in the shared environment. One category captured socio-spatial impacts on the sense of belonging. This implied spatial qualities that indicate group identity (e.g., availability and proximity of homebase areas, visual identifiers like color design, symbols, and individual decoration), group privacy (e.g., spatial, visual, or acoustic separation from other groups), and individuals’ own space within the group (e.g., personal space). Another category reflected the dichotomy of social interaction and refuge. Participants described social dynamics mainly in the context of spatial arrangement (e.g., floor plan, furniture arrangement). While social interaction was associated with inclusive spaces that promote teacher-class communication (e.g., circular arrangements, predefined assembly points) and children’s peer interactions (e.g., clustered arrangements), refuge was associated with exclusive spaces that ensure individuals’ privacy (e.g., separate arrangements, elevated room levels, nooks and crannies, dens). A third category dealt with spatial impacts on the sense of community. This concerned structural conditions that outline the community structure (e.g., exclusive building accessibility, user-appropriate qualities of space and equipment) and align to the community culture (e.g., shoe-free kindergarten, space for events, space for parents).

With a 17.76% share of total units, connectedness represented the least prominent theme. The assigned units carried mainly a negative quality (64.47%). Though appreciating the work atmosphere, participants’ descriptions of social spaces were overshadowed by restrictions of space and control. Utilizing socio-spatial arrangements, participants were eager to moderate social dynamics and thus improve communication and prevent stressful situations. This suggests that the ECEC professionals take a key role in social placemaking. Sufficient space and flexible arrangements may support these endeavors.

#### 3.2.4. Enactedness

The enactedness theme emerged from responses addressing the need of acting in and interacting with the environment, independently and effectively. We identified two categories reflecting the need of being enacted for both ECEC professionals and children. First, participants described environmental conditions that affect their teaching, caregiving, and other occupational activities. This included permanent spatial structures that support planning and organization (e.g., separate staff areas, presentation and storage space, kitchen and laundry equipment), and promote time efficiency and a continuous workflow (e.g., spatial options, sufficient equipment, proximity of frequently used areas, easy accessibility of equipment, functional usability of activity areas). The ECEC staff expressed the importance of modifying their work environment according to situational needs. This involved flexible spatial structures that allow for individual design of dedicated areas (e.g., decoration, room arrangement), and adaptation of space for certain activities (e.g., ambience control, furniture arrangement, delineation of activity and social areas). Second, participants described environmental conditions that promote children’s self-directed activities. This included both stimulating conditions (e.g., inviting ambience, visibility and attractivity of materials, delineated functional areas) and empowering conditions (e.g., accessibility of space and equipment, child-appropriate scales and proportions, spatial and material options).

Converging in 27.57% of total units, the enactedness theme slightly dominated the analysis. A narrow majority of assigned units (54.66%) was of positive quality which highlighted flexible arrangements, easy accessibility and adequate equipment. Shortages mainly related to spatial restrictions, and insufficient storage, presentation, design and playing options. Findings suggest that the active involvement of both teachers and children in creating temporal spaces is an essential part of placemaking in the kindergarten setting. Organizational structures and adaptive architecture may support these needs.

### 3.3 Indicators, dynamics, and levels of placemaking within the participating kindergarten community

The findings from our independent analyzes exhibited broad thematic overlaps and no thematic conflicts of children’s and ECEC professionals’ experiences and needs. This was confirmed by representatives of the participating kindergarten community in our feedback sessions with the consortium. Albeit these findings were relatable to each other, their interpretation was based on different research perspectives. While the themes deduced from the ECEC staff’s responses reflected the needs to be met to elicit a sense of place which may serve as indicators of successful placemaking, the themes derived from children’s probes reflected the spatial elements and the dynamics associated with the active placemaking process. This gave reason for further research consolidation.

**Figure 4:**
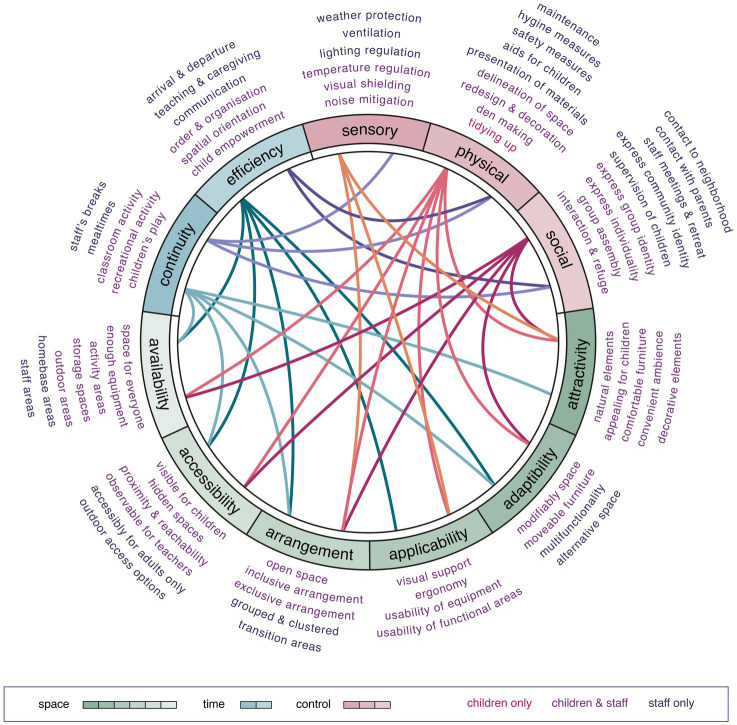
Supportive qualities for placemaking.

Our iterative research consolidation based on the latter interpretation scheme focusing on the participating kindergarten community’s specific placemaking dynamics. The consolidated findings highlight the importance of built environments that allow situational and inclusive placemaking. Figure 4 shows a circular model that summarizes outside to inside the stepwise research consolidation from the very nuanced need expressions of the participants to the interrelationships of the qualities that support active placemaking in the investigated kindergarten community. Six qualities influenced the perception of space, i.e., availability, accessibility, arrangement, applicability, adaptability, and attractivity. These spatial qualities related to participants’ perception of time. We uncovered two relevant time qualities in their daily routine, i.e., continuity, and efficiency. Both parties expressed their wish for better time management and for controlling or modifying their environment according to their needs. Three objectives related to requests of control, i.e., the sensory, physical, and social environment. Conversely, all three key determinants related to the four identified needs and different aspects of need satisfaction as derived from the staff’s responses. Examples of the joint analysis are available in the Supplementary material S9.

**Figure 5:**
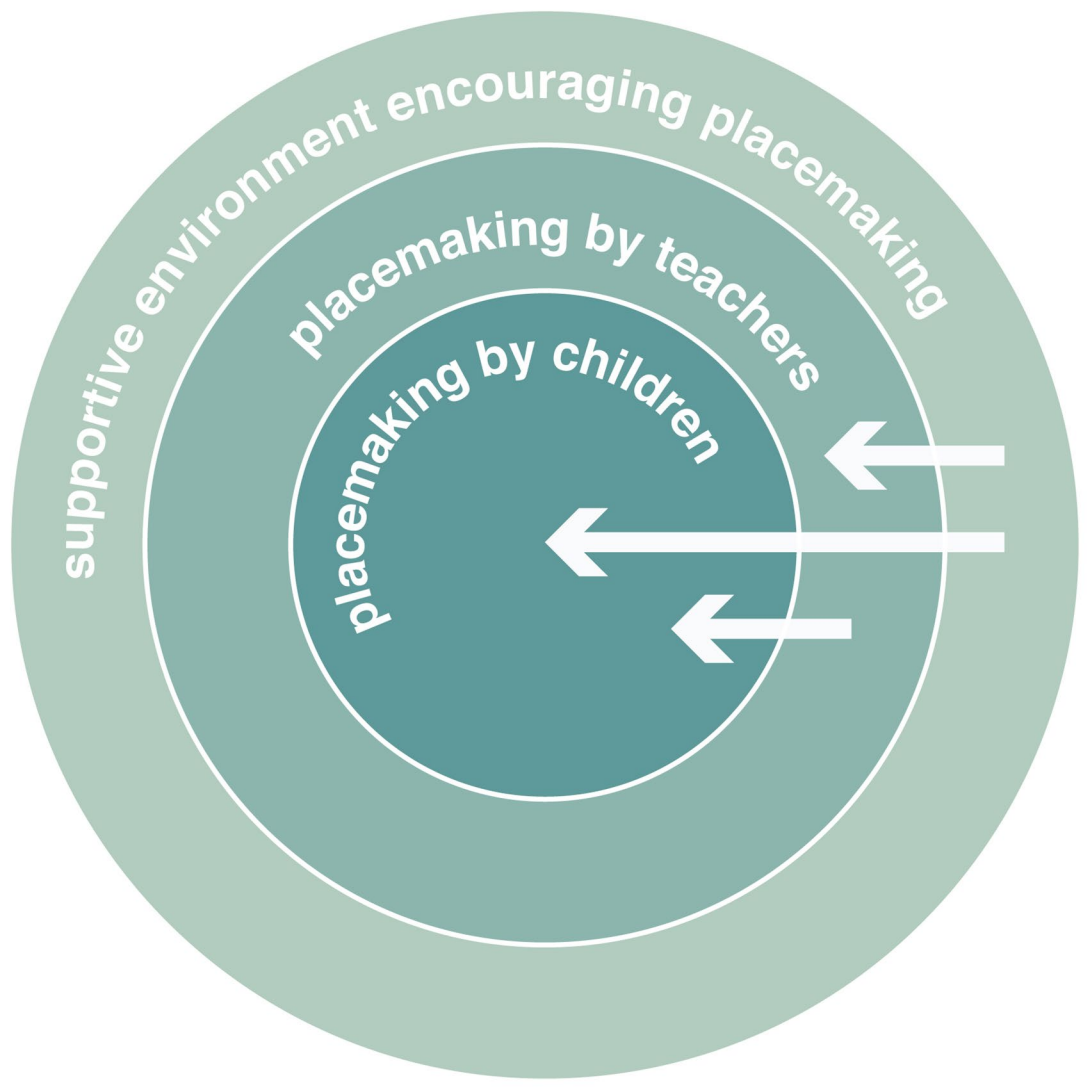
Levels of placemaking.

Finally, our consolidated findings led us to an understanding of placemaking that occurs at three levels in the participating kindergarten community as summarized in Figure 5. Preliminary placemaking occurs in the planning and construction of the built environment. Architecture provides a basic framework with spatial and material resources that considers the general needs of the kindergarten community. Within this framework, placemaking happens continuously within the daily routine of teachers and children. The teachers use the given resources to create a protected and stimulating environment for children that is ensuring their safety and care, promoting their social and learning experiences, and empowering them to explore the environment. Within this protected environment, children themselves create temporal places according to their preferences and needs – independently or with the support of adults.

## 4. Discussion

Our explorative study aimed to shed light on the participating children’s and ECEC professionals’ relationship to their kindergarten environment and to reveal potential placemaking spatial qualities that reflect the kindergarten community’s specific needs. We received insights into the children’s experience of spatial and temporal–spatial structures, and their need for control. Moreover, we received insights into the ECEC staff’s experience of the built environment as both their workplace and children’s place. The consolidated findings led us to a nuanced understanding of the indicators of place experience, as well as different levels and dynamics that reflect placemaking processes in the investigated kindergarten setting. Although our user-centered approach focused on portraying our case kindergarten community’s specific needs, thereby nudging the development of customized solutions, the collective wishes and concerns of our study participants also reflected common needs, which are center part of existing theoretical works, and addressed environmental conditions which have been empirically linked to health outcomes, academic achievements and workplace satisfaction in previous studies. We think these overlaps allow a discussion of the derived placemaking needs within a broader context.

### 4.1. Different theories and concepts support the derived placemaking needs

Different theoretical concepts underpin the needs we associated with the participating kindergarten community’s experience of place. We recognized a strong link to the 4E approach to cognition. The concept behind describes cognitive processes as embodied, embedded, extended, and enacted (Newen et al., 2018, p. 6): Experiences arise from being immersed in, interacting with, and acting in the physical environment. Similarly, the experience of place is largely sensual (Shamai, 1991) involving vision, hearing, touch, smell, taste, and balance. Addressing aspects of the kindergarten community’s orientation in their built environment and the surrounding world, these concepts became apparent in our themes embeddedness and enactedness, as well as time-making and control.

Another dimension of comparison may be found in different motivational theories. The themes protectedness, connectedness, and enactedness/control hold elements of basic human needs such as safety needs (Maslow, 1943, p. 376), social needs (Maslow, 1943, p. 380; Stevens and Fiske, 1995; Ryan and Deci, 2000), needs of competence and autonomy (Ryan and Deci, 2000), as well as the need of nature relatedness (Hurly and Walker, 2019). In line with concepts on biophilia (Kellert and Wilson, 1993), attention restoration (Kaplan and Kaplan, 1989) and stress reduction (Ulrich et al., 1991), expressions of local and nature relatedness, as apparent in our embeddedness theme, highlight the positive influence of environmental exposure on human well-being, and thus, may particularly contribute to the experience of place in our case kindergarten community.

Furthermore, the themes protectedness, connectedness, and enactedness/control reflect ideas of developmental psychology such as the attachment theory (Bowlby, 1997) according to which children’s independent play in the environment and their exploration of the environment builds upon the quality of attachment to a caregiving person and the associated feeling of safety, supporting the notion of socio-spatial structures, such as those pictured in our participants’ returns, playing a central role in children’s experience of place.

Not least, the congruent themes enactedness and control suggest links to concepts of community psychology such as the empowerment theory (Zimmerman, 2000) which positions individuals’ self-determined participation in their community as a key factor of social well-being. Empowerment is seen context and population specific on both physical and psychological level. A supportive physical environment with active placemaking opportunities, as requested in our case study’s kindergarten community, can serve these levels of empowerment in children and teachers, and contribute to a flourishing kindergarten community.

### 4.2. Empirical evidence suggests benefits from building design related to the derived placemaking needs

Evidence from medical, psychological, and educational research suggests that specific environmental conditions, which are inherent to the themes that arose from our participants’ returns, contribute to positive behavior and social experiences of children, smooth classroom management for teachers, and the physical and emotional well-being of both. These parallels became most prominently apparent in our participants’ sensory and spatial experiences, and thus, represent important cornerstones of placemaking in our case kindergarten setting.

#### 4.2.1. Sensory aspects of placemaking

Four sensory aspects influenced the kindergarten reality of our participants as reflected in all our themes, i.e., acoustic environment, lighting conditions, window view, and indoor climate. Noise was a disruptive element in our case setting, hindering recreational and classroom activities as perceived by the ECEC staff. Interestingly, children also expressed their preference for low noise conditions. Thus, we consider noise moderation as a crucial placemaking component in the overall noisy setting. This is underpinned by comprehensive evidence on the negative influences of high noise levels on children’s speech perception (Jamieson et al., 2004), pre-reading skills (Maxwell and Evans, 2000), and classroom behavior (Persson Waye et al., 2019). Concerning the well-being of ECEC professionals, high noise levels have been shown to be associated with various health problems including voice and hearing problems and headache (Kankare et al., 2012; Hadzi-Nikolova et al., 2013; Yassin et al., 2016), increased stress, and risk of burnout (Sjödin et al., 2012). Notably, increased noise at the workplace also negatively correlated with employees’ sense of place at work (Miller et al., 2001).

In our case kindergarten community, lighting conditions affected the staff’s visual comfort and teaching activities. Overall, we noticed preferences for brightness and daylighting, window view onto green space or landscape, and requests for better lighting control. Findings from field studies suggest that lighting qualities, such as color temperature and intensity, correlate with young children’s cognitive performance (Hartstein et al., 2018), and visual impairments (Cohen et al., 2022). Furthermore, Giraldo Vásquez et al. (2019) showed that young children were able to distinguish lighting needs as relevant for the activity performed, and the authors reported preferences for a window view over closed curtains, especially for natural views. Moreover, studies with adults showed that daylight exposure correlated with subjective well-being and better sleep quality (Boubekri et al., 2014), while window view and the perception of nature elements correlated with lower physiological and subjective stress, better workability, and increased job satisfaction (Chang and Chen, 2005; Sop Shin, 2007; Lottrup et al., 2015).

Regarding climate conditions, we captured seasonal thermal preferences from both children and adults, notably focusing on shading and cooling options in the warm seasons of the year when we conducted the study. Literature points at differences in thermal comfort between children and adults: Models based on datasets from a British primary school predicted lower thermal comfort temperatures for children than existing comfort standards suggest for adults (Teli et al., 2012). These findings could be replicated for kindergarten children and adults in an experimental setting in China (Chen et al., 2022) and in a field setting in Korea (Nam et al., 2015).

In addition, in our study, the ECEC staff addressed issues about indoor air quality and ventilation. The negative effects of bad indoor air quality in education facilities have been investigated in a large-scale quantitative study by Branco et al. (2020): Elevated indoor air pollutant levels measured in primary schools and kindergartens in different European countries have been related to increased odds for various health problems including respiratory conditions in children. Thus, temperature and indoor air quality may play a decisive role for children’s and teachers’ short-term and long-term well-being.

#### 4.2.2. Spatial aspects of placemaking

In our case kindergarten community, two spatial aspects influenced children’s and ECEC professionals’ experiences about their shared environment. On the one hand, aspects such as safety measurements, building maintenance and hygiene made up a large part of the ECEC staff’s reality. The sense of safety attributed to the built environment reflects the ethics of care (Noddings, 2013) that governs teachers’ practices and may be a key factor of place experience. Findings from elementary school settings suggest that notable safety measures, building maintenance and cleanliness largely shape the sense of safety and the sense of place of both, children and teachers (Maxwell, 2000); and with special regard to children’s perception, the obvious presence of adults has been identified as another contributing factor (Langhout and Annear, 2011). Spatial qualities fostering the accessibility and visibility of people, as comprised in our protectedness theme, are adding to this.

On the other hand, socio-spatial arrangements strongly shaped our case kindergarten community’s reality in different ways. Due to the rising number of children, the ECEC staff considered the mere availability of space as a major influencing factor on classroom management and social dynamics. While enough space fostered smooth activity, organization, and social inclusiveness, shortage of space hindered activities, communication, and organization, and caused waiting times and stress. Different studies discuss similar observations in context of space-class size ratio: While the benefits of small group classes on children’s achievement were minor (Milesi and Gamoran, 2006), adding the factor of space positively influenced teacher-student interaction, teachers’ enthusiasm and job satisfaction (Şahin et al., 2011).

In line with the perceived lack of space in the investigated kindergarten setting, our collective findings also highlight the importance of exclusive and intimate spaces for the participating kindergarten community. This concerned different social settings, such as individual children’s resting and refuge places, classes’ dedicated areas and sensory privacy, as well as teachers’ personal storage space and separated staff areas. Findings from different field studies strengthen our case observation: Friedmann and Thompson (1995) observed preschool children frequently using intimate spaces for diverse activities, and associated their varying preferences for different types of intimate space with age. In a similar setting, Colwell et al. (2016) encouraged preschoolers to actively create their intimate spaces and observed that the children preferred flexible materials; and moreover, differentiated between hidden spaces and spaces that were observable for adults, which led to the conclusion that intimate spaces give children a sense of safety and control. Similarly, exclusivity of space has been related to employees’ experience of place at work. Consulting desk-bound employees about the qualities of their workplace and their job satisfaction Miller et al. (2001) reported links between their participants’ sense of place at work and the presence of personal objects, the perception of privacy, and the possibility to control furnishing arrangement - aspects of exclusivity that were also present in our participating ECEC staff’s responses.

### 4.3. Implications for the case kindergarten building design and follow-up research

The three placemaking levels we concluded from our consolidated findings (see Figure 5) gave direction to further steps in the planning process. Accordingly, we targeted solutions that foster the empowerment of children and teachers. More concretely, as a follow-up step, we designed, fabricated and deployed three research prototypes that target the emergent placemaking needs. One prototype facilitates space division according to spatial needs and contributes toward lowering noise levels as it has the form of room dividers covered in passive noise-absorbing foam. The second prototype is a voting system that aims to democratize control over environmental factors such as temperature, light conditions, and sound volume. The children are given the chance to cast a vote on their preferred condition (e.g., quieter or louder, darker or brighter, colder or warmer) as well as vote on their own chosen factors (e.g., going outside, playing in the gym, or staying in their classroom). The third prototype is an interactive building block set that metaphorically communicates the passing of time through “tiredness”. After a given amount of time, the interactive properties of the building blocks (i.e., magnetism, illumination, vibration) cease to function indicating the end of the play activity. The artifacts were deployed at the kindergarten with the aim of being further iterated and deployed at the kindergarten indefinitely. The findings from the 4-day evaluation are reported in a follow-up publication.

### 4.4. Strengths and limitations

#### 4.4.1. Strengths and challenges of the participatory design approach in the specific study context

Overall, we see the strengths of the present exploratory study in its cross-disciplinary multi-method design which allowed us to uniquely merge the views of the participating children and ECEC professionals on place requirements in their kindergarten environment. Moreover, the practical approach combined the advantages of research in the field and close collaboration with all parties involved in the participatory design process which allowed timely decision-making in the early building planning phase based on our research findings. At the same time, the COVID-19 pandemic and the concomitant national and regional restrictions challenged the study conduction through deferral of both the probes study with children and the study inquiry meetings with the ECEC staff. Moreover, as the access to the field was limited for researchers, the materials had to be adapted for remote application. The participatory design approach is characterized by good communication and exchange between researchers and the participating end-users. By fostering increased participant motivation and engagement in the building design it aims for more detailed and nuanced data and a more comprehensive understanding of concerns and needs. The restricted contact, thus, may have led to a less nuanced picture of our participants’ reality and limited the strength of the methodology.

#### 4.4.2. Advantages and challenges of developing and deploying cultural fiction probes for participatory design with young children

The cultural fiction probes was a strong approach that integrated children’s perspective. The procedures of probe development reflect the creative design processes used in the HCI field. From a more conservative point of view, these techniques may appear messy. However, this messy process adequately addresses the timely knowledge transfer into design practice and considers the specific context in the field and available resources. One limitation may be the replicability of our approach: Although the presented probes can be deployed in different study contexts based on the provided materials, the process of conceptualization is dependent on the researchers’ expertise and background and remains unique. Similarly, the inductive theme development and the dynamics of cross-disciplinary workshops are colored by the research objective and research team’s expertise.

Concerning the deployment of cultural fiction probes in the field, remote data collection could only be realized with the assistance of teachers, and some probes required comprehensive interpretation, which came along with risks of bias. Table 1 lists potential advantages, limitations, and possibilities of deploying cultural fiction probes to investigate children’s spatial experience in the ECEC setting.

**Table 1:** Advantages, limitations, and possibilities of deploying cultural fiction probes.

Strategy	Advantages	Limitations	Possibilities
Fictional story	A fictional story involving helping imaginary peers can be used for the activity briefing and capture children’s interest	The characters in the story could coerce the children into performing activities they do not comprehend	Researchers must ensure that their activity requests are comprehensible in terms of language and children’s abilities per targeted age group
	Children are more likely to engage in activities involving fictional peers than adult strangers	Requires some storytelling skills	The fictional story must follow a logical sequence, have integrity, and be consistent
	A fictional story may help establish a friendly bond between the children and the researchers		
Informal interviews with each child	Captures opinions of each and every child, not just the ones who are outspoken	The effort of performing the interview relies to the teacher who on one hand is capable to extract answers from children and talk in their own terms yet they are not trained in performing interviews and the data might be skewed to reflect their perspective than the voice of the children	Children’s responses should be the primary data and they should not be influenced by the teachers’ opinions
	Voice recordings rather than notes give a more objective view of children’s perspective	Children might mimic or repeat other children’s responses	The researcher has to distinguish which responses are perspectives of the children or the teachers’
	Children might enjoy talking about things that are important to them in their space. Sharing preferences multiple times might provide more insight into their perceptions and preferences	Young children might have difficulty expressing themselves verbally and explaining their feelings and perceptions	
Child-led photo elicitation	The approach places children’s desires and preferences in the spotlight as the expert of their environment, and it could potentially provide them with empowerment as they have the “steering wheel” in such activities	Adults might pry into a world that children did not want adult eyes to witness and could be seen as a breach of privacy	Adult researchers should set boundaries in their pursuit of comprehending children’s view to protect the children’s privacy rights
	Reveal aspects not captured verbally, provides the opportunity to children who are non-verbal to express themselves through a different medium	Children might mimic or repeat other children’s responses	
		Children might need help operating the camera in the beginning of the activity	
Representational descriptions (models or drawings)	Can be an engaging and fun activity for young children and quite appropriate even for young ages	Children might mimic elements from their peers’ art	Allow opportunities for different artistic expressions
	Children have the control over the outcome of the activity and can express themselves more freely than an interview or other verbal communication means.	Children might create what they think the teacher wishes them to create	Allow children to set their own pace and rules
		The researcher might not be able to interpret the art without any descriptions	Use a combination of data collection strategies, e.g., clay modeling and verbal descriptions of what the children made, or lego constructions and photographs of the creations with short descriptions
		The teacher may misinterpret the children’s descriptions and comments	
		Young children might face difficulties in expressing themselves verbally and explaining their	

#### 4.4.3. Advantages and challenges of deploying conventional inquiry methods for participatory design with ECEC staff

Aside from the external restrictions described above, the conventional methods themselves encountered their limits in the context of building construction reality:

The participatory design approach internalizes the tabula rasa principle that characterizes exploratory research in general. Deploying the chosen methodology required us to take a step back from accustomed procedures as well as from the existing body of research and known evidence-based environmental variables, and consequently, to give up some control over the research process in favor for a better involvement and unbiased understanding of participants’ unique concerns. Although we ensured time-efficient study conduction, conventional content analysis turned out to be rather uneconomic for the purpose of immediate knowledge transfer. On the one hand, the performance of a detailed inductive qualitative content analysis was very time-consuming, and thus, required massive personnel resources that may not be justifiable within the often-tight budgets of community building projects. On the other hand, the analysis method, which is considered as flexible in research, appeared as a very rigid procedure for the rather agile nature of the participatory design processes that require the timely opening of findings for stakeholder feedback and parallel research consolidation. Nonetheless, the chosen methods added value to the present study findings as well as to the associated research project as the detailed analysis of very elaborate information allowed to meet the complexity of the following research consolidation. This not only resulted in a sophisticated model of placemaking dynamics (see Figure 4) but also initiated bottom-up development of more economic tools for future research. In addition, our findings supported the project’s architects and other stakeholders in better understanding the needs of the kindergarten community and thus, taking more informed and sustainable decisions regarding the upcoming building design.

Conversely, from the conservative point of view, the fusion of different methods and epistemologies may have limited the scientific rigor of the present study, especially regarding the high standards established in conventional qualitative research. Different types of bias cannot be excluded:

Despite the participatory character of the study, we aimed to create a protected setting that encourages individual staff members to openly communicate their workplace experience. Thus, we strived for the possibility of anonymous participation in the rather family-like environment. However, we realized that the teachers filled in the survey form on-site during their working hours. It is likely that children and colleagues were present during survey completion. The returned forms gave reason to assume that some teachers might have collaborated and that others might have been hindered by their work duties, restricted time or lack of privacy. As workplace satisfaction is a sensitive topic, we cannot exclude a response bias, though we believe that such a bias would have resulted in more positive workplace evaluations than reflected in the collective data.

Moreover, though the methods we used for consulting the headmistress and teachers matched by content, we recognized varying response behavior associated with the different presentation formats. While the final interview protocol was very elaborate, the survey content was less comprehensive. Merging the headmistress’s and teachers’ responses for analysis, thus, may have led to an interpretation bias in favor of the headmistress’s voice. We conclude that the deployed method is suitable for the interview format in the on-site building tour setting where participants simultaneously experience the environment, better than for the written survey format in the remote setting where participants most likely solely imagined the spatial conditions. Also, the interview format allowed the researcher to check back on more detailed explanations and feedback which supported content elaboration. Voice records and the classical analysis of transcripts may have led to even more detailed insights. Transferring the interview guide into a survey form was an exploratory approach to meet the increased demands of flexibility but also to compensate for the limited building access at the time of study conduction. The written survey allowed flexible participation within a 3-week period and took into account the varying working hours and restricted capacity of ECEC staff. However, based on the data received, we see that the survey requires improvement with special regard on questions that encourage more explanatory responses to gain detailed insight into the staff’s point of view. An alternative method for the purpose of participatory design may be a building tour in focus groups or individual interviews, which would, however, demand more resources from both the staff’s and the researchers’ side and impede anonymous workplace evaluation. Importantly, the simultaneous involvement of teachers in the double role as participants, and as front performers during data acquisition from children may have also influenced their own response behavior. Though the parallel research activity allowed us to compare children’s and ECEC professionals’ perspectives about their shared environment within the same temporal context, the participation of teachers may have been complicated by the added burden and might have been better conducted in a separate time slot.

A point of discrepancy can be made about the limited number of participants in this part of the study. However, the ECEC inquiry participants consisted of all the teaching staff employed at the specific kindergarten, thus, a very homogenous group. Guest et al. (2006) support that in qualitative studies saturation – the point at which no new information or themes are observed in the data – usually occurs with around 12 participants in a homogenous group, with other scholars arguing that saturation could occur at a smaller sample size as well. For example, Hennink and Kaiser (2022) provide the range of 9–17 interview participants to reach saturation within a homogenous group.

Not least, we recognize a risk of bias in the quantification of findings as only one researcher was involved in the content analysis of the kindergarten staff’s returns. To increase reliability of the coding outcome, we opted for reliability checks with intervals of 1 week minimum. A second coder would contribute to improving methodological quality, however, also require more resources.

#### 4.4.4. Advantages and challenges of cross-disciplinary integration for immediate research transfer and recommendations for future research

A major aim of the cross-disciplinary research project was a timely knowledge transfer and the development of innovative solutions with good usability for the collective participating kindergarten community. To ensure independent stakeholder voices, we strictly separated the studies with children and the ECEC staff between our research teams. The double objective required an inclusive methodological design that responded to the inhomogeneous samples’ reality. While the cultural fiction probes method uniquely captured children’s close-up impressions of their environment, established qualitative methods ought to complement the rather innovative approach and to strengthen the common interpretative outcome by capturing a comprehensive picture of the kindergarten reality of teachers and children from the staff’s perspective. In line with this, we chose corresponding analysis methods which allowed us to, respectively, frame and nuance the kindergarten community’s needs.

Notably, parallel findings may largely reflect the shared environmental setting and may be influenced by synchronous data collection as well as by the involvement of participating staff in data collection with children. Moreover, research integration occurred in a long-lasting, iterative process in close collaboration of two representing researchers of the partnering teams which may have additionally influenced the scientific quality of findings. However, the mutual influence and exchange were an integral part of the participatory design activity and resulted in a knowledge conglomerate that was communicated to the partnering architects. This, in turn, allowed the immediate transferability of consolidated findings into the case practice. With regard to the overarching objective of timely knowledge transfer, we encourage the integration of complementary expertise, and correspondingly, a more agile multi-method-use in future cross-disciplinary participatory design projects.

It is important to note that even though our research teams arrived from distinct epistemic traditions, we observed early on that the findings from both adults’ and children’s perspectives complemented each other and provided a deeper overview of their needs, merging objective and socially constructed realities. The unprompted triangulation ratifies the advantage of combining different epistemologies and calls for further integration of cross-disciplinary research in participatory design related to the built environment. Albeit the study design limits the transferability of findings to the construction process in our research setting and only a case-to-case generalizability is to be considered, our consolidated findings are eventually interpretable within the existing theoretical and empirical framework discussed above, and thus, can serve as a base for more comprehensive research endeavors, in particular but not limited to the design of ECEC environments.

Overall, the findings of the present study hold potential implications for future research giving impulses for the development of more time- and cost-efficient tools for the identification of placemaking needs and the assessment and evaluation of experience of place. Regarding practice and policy making, such new tools can support architects in the implementation of co-design strategies along the building construction process that results in more humane, healthier, and empowering architecture.

### 4.5. Conclusion

Apart from Post Occupancy Evaluation, future inhabitants and building users are typically not involved in the architectural design and building process of public architecture. To our best knowledge, this is the first study deploying newly developed methods tailored for participatory design with 3-to-6-year-old children to integrate their experience of their kindergarten into the building design process. The combination with a conventional method was backing this innovative approach, and in parallel, providing insight into the ECEC professionals’ point of view, resulting in a triangulation and interconnection of findings. Although the scope of transferability is limited to a case-to-case basis, our findings are interpretable within a solid framework of existing theories, concepts and evidence. In this study, the key determinants of the participating kindergarten community’s space use and perception of the environment were associated with spatial characteristics, time-constraints, control over the environment and noise pollution. The main placemaking indicators related to the needs of feeling embedded, protected, connected, and enacted in the shared environment. Even though participation in architectural projects is not a novel concept, the participation and cross-disciplinary collaboration of researchers for the purpose of communicating the contextual community’s needs and wishes is breaking new ground toward delivering practice-based design knowledge and an adequate example of research informing and working with industry. In this case, especially the HCI field can serve as a model practice of user-centered approaches for architectural design. The cross-pollination of fields and the transfer of participatory methods from one arena to another come with a rewarding outcome; all parties working in tandem from the pre-design phase to produce *ad-hoc* healthier and more humane built environments with unique interaction possibilities. We see the involvement of future inhabitants as an act of empowerment in itself, bringing children and teachers a step closer to becoming active equitable citizens in the ECEC building design process. Structural solutions that encourage placemaking activities of teachers and children can contribute to their individual well-being, and consequently, may support children’s development, increase teachers’ workability and job satisfaction, reduce rates of absenteeism and dropout, and sustainably strengthen the ECEC system. Future evidence-based design research may pursue such potential benefits.

## Data availability statement

The raw data supporting the conclusions of this article will be made available by the authors, without undue reservation.

## Ethics statement

The studies involving human participants were reviewed and approved by the ethics board of the University of Salzburg (Ethikkommission der Universität Salzburg). Written informed consent to participate in this study was provided by the participants’ legal guardian/next of kin.

## Author contributions

AH and CP acquired the project funding. AH, CF, and CP conceptualized the research. CF and CP contributed to the study design and methodology. CF and EE developed the cultural fiction probes materials. AH, CP, and NG developed the inquiry material for building evaluation. EE and NG contributed to the data collection, integrated, interpreted, and visualized the findings, and wrote the original draft of the manuscript. CF, EE, and NG performed the data curation and data analysis. AH and CF administrated the project and supervised the study conduction. All authors contributed to the manuscript revision, read, and approved the submitted version.

## Funding

This project was supported by the Science and Innovation Strategy 2025 of the Federal State Government of Salzburg, Austria (WISS 2025): PFL142230_01.

## Conflict of interest

The authors declare that the research was conducted in the absence of any commercial or financial relationships that could be construed as a potential conflict of interest.

## Publisher’s note

All claims expressed in this article are solely those of the authors and do not necessarily represent those of their affiliated organizations, or those of the publisher, the editors and the reviewers. Any product that may be evaluated in this article, or claim that may be made by its manufacturer, is not guaranteed or endorsed by the publisher.

## Supplementary material

Supplementary Data Sheet S1 Fictional narrative transcription (English translation).

Supplementary Data Sheet S2 Cultural fiction probes concept.

Supplementary Data Sheet S3 Interview material for building evaluation (English translation).

Supplementary Data Sheet S4 Survey material for building evaluation (English translation).

Supplementary Data Sheet S5 Photographic record of building tour.

Supplementary Data Sheet S6 Data from building evaluation.

Supplementary Data Sheet S7 Thematic analysis of cultural fiction probes.

Supplementary Data Sheet S8 Codebook for content analysis of building evaluation.

Supplementary Data Sheet S9 Joint analysis and cross-disciplinary research consolidation.
