# Supplementary material for: Uncovering placemaking needs with(in) a kindergarten community: a cross-disciplinary approach to participatory design
Source: Front Psychol. 2023 Jun 20;14:1126276. doi: 10.3389/fpsyg.2023.1126276 (PMC10319412; doi:10.3389/fpsyg.2023.1126276)
Supplement: Supplementary Data Sheet S1 — Fictional narrative transcription (English translation). [file Data_Sheet_1.PDF]

FICTIONAL NARRATIVE TRANSCRIPTION

LEGEND:
Instructions for teachers
Text received by the children
Activities
Ideas
Questions
Remarks
Keywords
Capturing

PROBE 1

The teacher says that they found this box in the garden this morning.

Now the teacher plays the first recording from the iPod and the following story is told:

Hello dear children! We hope that this message reaches you. We are contacting you from the distant planet of Harkarosh. To get from your kindergarten to ours, you need a big rocket or a spaceship - our planet is so far away. Only children live on Harkarosh, there are no adults there. In the past, our planet was known for the fact that you could have a lot of fun on it. Our laughter was heard as far away as the other planets. Maybe you even heard us once on Earth? Unfortunately, things are very different now. We children of Harkarosh have become bored. We just don't know what to play with and how to play anymore, so we don't have any fun at all. All the children sleep almost the whole day and we are sad because of boredom. Therefore, we urgently ask you for help! The box from which you hear my voice is a teleporter. A teleporter is a device with which you can talk to each other - almost like a cell phone. We children from Harkarosh and you children from Earth can even send things to each other with this device. Isn't that great? We have sent you digital cameras today. Can you please use these cameras to take pictures of your favorite games, toys, and places you especially like to play? Maybe we'll find the fun in playing again if you remind us with your photos what and what you like to play with and where you like to do it the most! Shukuri! In our language this means "thank you and see you soon"!

Take 2, write down only keywords and let the child write the text.

Give the children the camera(s) and then have them take photos of it

Photos of favorite games/toys/spaces.

40 MIN

PROBE 2

Bright and dark spaces, paint with bright colors

Hello again little earthlings! We loved the suggestions you sent us last week about your favorite toys and spaces. Thank you so much for helping us understand what play is! We send you these secret messages containing things that we have in our planet around that you can offenbaren with the help of magic colored light. We send you these flashlights and their filters to discover our messages. Make the room dark or find a dark spot and radiate our messages. Use the filters to color the light and make the secrets appear! If you want, drag with the special markers most fun object you have discovered and send it back to us!

Children gather in the tent to find ways to play using a flashlight and the other tools.

PROBE 3
Teacher clicks on button again and a new message is played

We have these materials and these interaction possibilities, how can we play with them?

Kinder wählen Material Samples, schauen wie sie mit diesen Spielen können und welche Interaktionsmöglichkeiten, sie sich wünschen. Danach geben die Kinder die Materialien mit den Interaktionsstickern in die Box zurück und beschreiben wie sie sich das spielen damit vorstellen.

Children choose material samples, see how they can play with them and what interaction possibilities they would like to have. Then the children return the materials with the interaction stickers to the box and describe how they imagine playing with them. Teacher puts the prints in the box

Audio recordings + materials + interaction possibilities

Hello dear children! Wow! Thank you for sending us your favorite secret signs! We also sent you something from us with the teleporter today. We sent you a lot of different things from our home planet Harkarosh. Unfortunately, we don't know how to play with these things... Can you please help us? You can do anything with the things we sent you: Paint, cut, glue... Just anything you can think of! We are sure that you have lots of great ideas how to play with these things. And you know what the best part is? On Harkarosh, these things can do even more than on Earth. So you can think about what they should be able to do on our home planet... If you want them to be bright on Harkarosh, or as cold as an icicle, or make beautiful music, or maybe even to be able to fly or, or, or.... you can tell us all about it! We will send you this time with the teleporter a special device that can record your voices so that we can hear your great ideas. We are so excited to see what you come up with and are looking forward to finally hearing your voices! Shukuriiiiii!

40 MIN

PROBE 4

What areas do you like in your kindergarten and can you assess that for us.

Children create drawings of their own bodies. Then place these in places they particularly like or dislike and attach the matching emotion sticker and then photograph this.

Photos of the drawings in the chosen places

Hello children of Earth! Today we are sending you many very large sheets of paper with the teleporter. Can you lie down on one of these large pieces of paper and ask a friend to go around your body with a marker? We would like to know how tall you children from Earth actually are. We children from Harkarosh are so small that you probably wouldn't be able to see us properly if we visited you in your kindergarten. But we've heard that you children from Earth are already really big - much bigger than us children from Harkarosh. When you have finished drawing, please give your own paper body to a place in your kindergarten where you like it best or maybe not so much. Thank you and see you soon! Uhhh, I mean of course: Shukuri! Hahaha...

40 MIN

PROBE 5

Since it is so quiet on our planet, we would like it to be a little louder, what sounds do you like (not)

Children take the audio recorder and record sounds, or create sounds that they record

Audio recordings of sounds

Hello dear children! (an excerpt of classical music plays in the background) Ohhh beautiful! We children of Harkarosh love beautiful music and beautiful sounds. That pleases our ears. But there are also sounds that we don't like at all. Do you feel the same way? Which sounds do you children from Earth actually like and which ones do you dislike at all? Today we have sent you five microphones with the teleporter, with which you can record such sounds in your kindergarten. We are curious to hear what you send back to us with the teleporter. While we're waiting for your message, we'll listen to some more of this beautiful music. (Classical music resumes) Shukuri!

Interruption of the game:
(silence for minutes, so as not to immediately focus the children's attention on the box when the pedagogue turns it on)... (then a previously unfamiliar, deeper (but not fear-inducing ;-)) voice) ALL CHILDREN STOP PLAYING IMMEDIATELY!... NO MORE PLAYING NOW!... ALL CHILDREN PLEASE CLEAN UP!... STOP PLAYING!... ALL CHILDREN CLEAN UP NOW!

40 MIN

PROBE 6

Thank you so much, for all the info, can you also tell us again about your favorite activities in the last week?

Giving the voice recorders to the children

Audio recordings of the children's feedback

Hello children from Earth! Oh dear, now Knorzo has disturbed you while playing. He does the same thing with us on Harkarosh. Whenever we are supposed to eat something or it is time to go to sleep in the evening, Knorzo tells us that we have to stop playing. Now he has done the same with you. How do you find that? We would love to know how you just felt when you suddenly had to stop playing? Please send us a message! Shukuri!

FAREWELL
So, dear children from Earth! You have helped us a lot with your many messages and we are finally having fun playing again. Thank you very much! We have heard that summer will soon begin for you and that you will no longer be in kindergarten every day. Here in Harkarosh the winter will start soon and in winter we children of Harkarosh hibernate. But as soon as it will be spring again here, we will contact you again. In the meantime, we have a little something for you: We'll send you postcards today with the teleporter, which you're welcome to send us summer. You can paint your postcard yourself or ask your moms and dads to write something for you on the card. When you are done, please send the card to [location] where it will be picked up by our space mail and brought to Harkarosh. We are really looking forward to receiving mail from you and wish you a great summer. Shukuri!

40 MIN
