# Supplementary material for: Uncovering placemaking needs with(in) a kindergarten community: a cross-disciplinary approach to participatory design
Source: Front Psychol. 2023 Jun 20;14:1126276. doi: 10.3389/fpsyg.2023.1126276 (PMC10319412; doi:10.3389/fpsyg.2023.1126276)
Supplement: Supplementary Data Sheet S2 — Cultural fiction probes concept. [file Data_Sheet_2.PDF]

APPENDIX S2 - CULTURAL FICTION PROBES CONCEPT

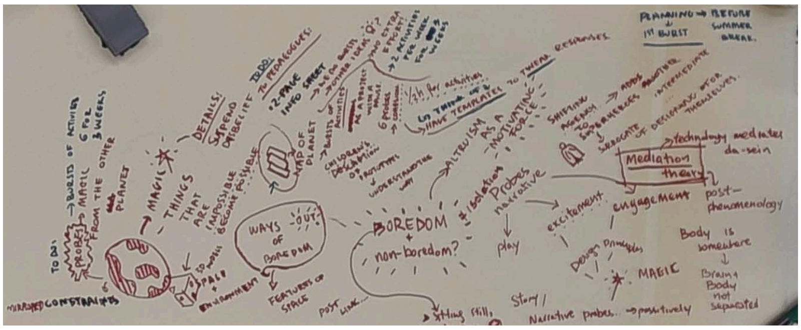

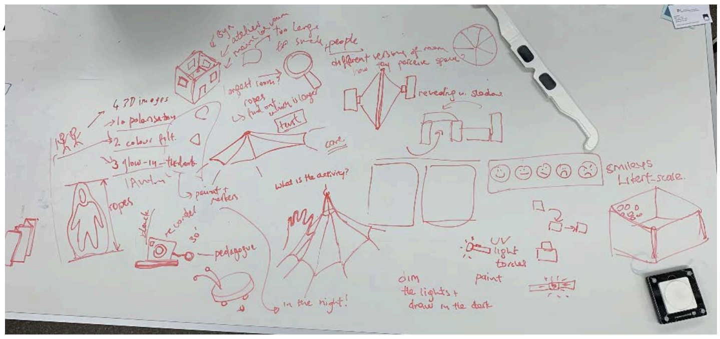

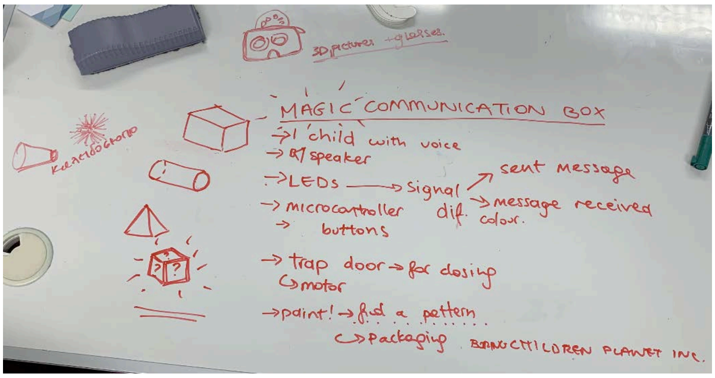

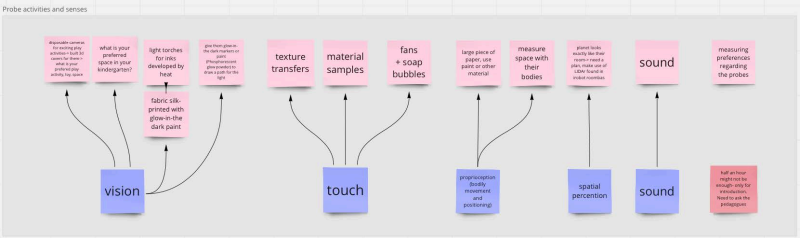

Examples of ideas for the cultural fiction probes approach documented during our iterative workshops using physical and digital whiteboard tools.

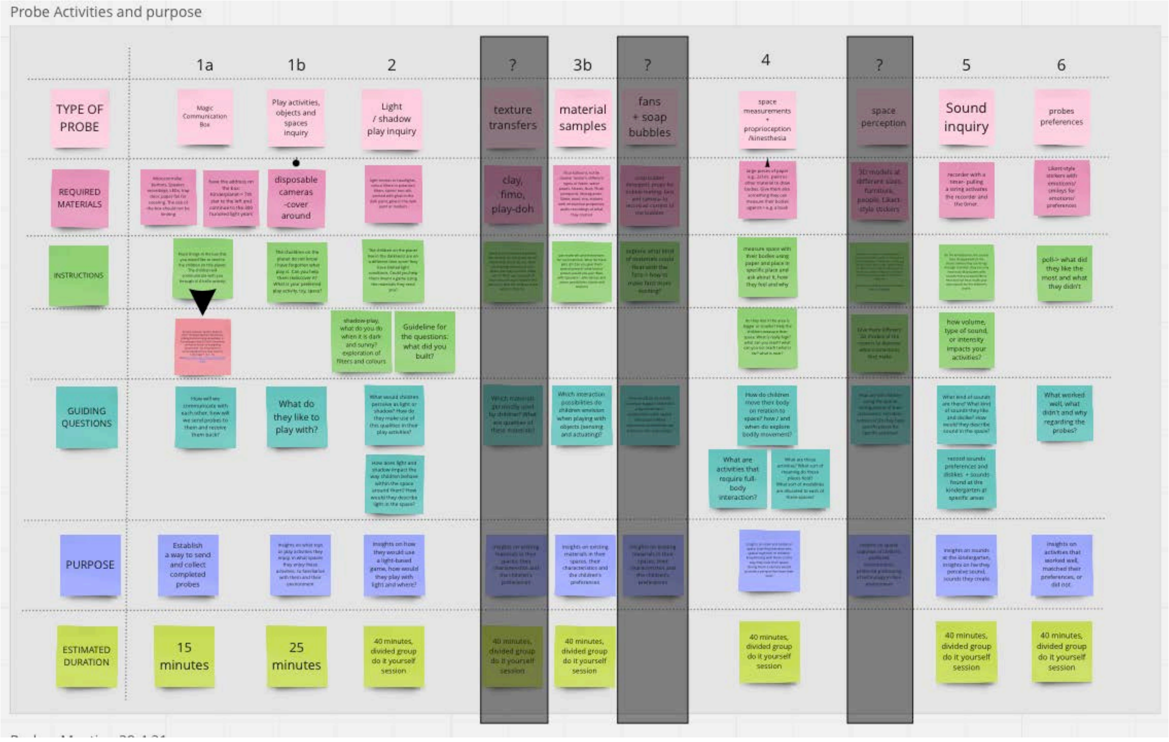

Initial draft of the 6 selected ideas for the cultural fiction probes activities.

PROBE ACTIVITIES DESCRIPTION

1A. Communicator box: a box covered in holographic paper foil containing an iPod and a speaker with all the recordings of the fictional narrative that instructs the six cultural fiction probes. On the box there are decorative push-buttons. Through the presentation of this box the narrative can be introduced and along with the overall purpose of the following activities.

1B. Photo Elicitation activity: In the material's box we placed a digital camera for children. This activity requests from each child to capture their favorite activities, objects, and places around their kindergarten environment.

2. Light riddle: Light torches, coloured filters, activity sheets and markers were placed in this probe's box. The aim of this probe was to let children explore light conditions in their kindergarten environment and document what they see around them by means of drawings when the lights are dimmed.

3. Material samples: The probe box contained crafting materials such as flour balloons, bottle cleaner twisters, wooden sticks, different types of fabric, stickers, among others, material properties (such as light, colorful, magnetic etc) written on paper and four voice recorders shaped as microphones. Our narrative requested children to construct new materials with special capabilities in order to get a sense of what children prefer regarding environments' materiality and material properties. We instructed the pedagogues to interview each child on what they had created and why.

4. Spatial Scale and proportions: Large pieces of paper, paint and other drawing materials were in the box of this activity as well as the digital camera used in activity probe 1B. The children were asked to create body outlines by tracing their bodies on paper and placing them in their favorite areas. Through this activity we wanted to get a better sense of the children's proportions in relation to their favorite spaces and ways they use the spatial configuration of their environment in relation to their body scale.

5A. Sound Inquiry: In this box we included the voice recorders used in probe 3. The activity requested from children to record sounds they like or dislike to hear around their kindergarten environment. The instructions to the teachers included asking the children which sounds they prefer or dislike and why. The aim of this activity was to capture insights on sounds and their volume as well as children's preferences when it comes to sound conditions.

5B. Cease of play: This box contained the voice recorders from the previous activity. We asked teachers to record children's opinions and reactions to the narrative's instruction that they should cease their play activities at once. Our goal for this activity was to gain insights on the children's views on the change of activities in their environment and their thoughts on time passing.

6. Probes feedback: In the final probes box we placed three likert-style sticker sets; green for satisfied, yellow for indifferent, red for dissatisfied. We asked the teachers to host a voting activity on each activity so that children could vote on their preferred or disliked probe. In addition, we asked teachers to take written notes on what worked well or not during the activities. Through this probe we attempted to gain knowledge on the children's preferences regarding the probe activities and feedback on each probe's performance.

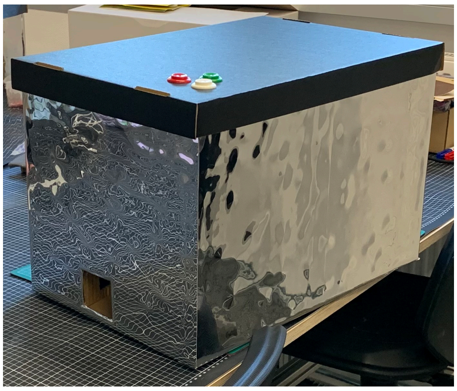

1A communicator box

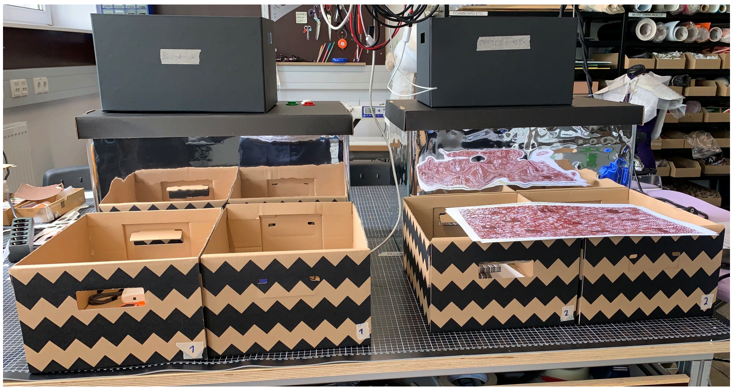

Boxes containing probes' materials

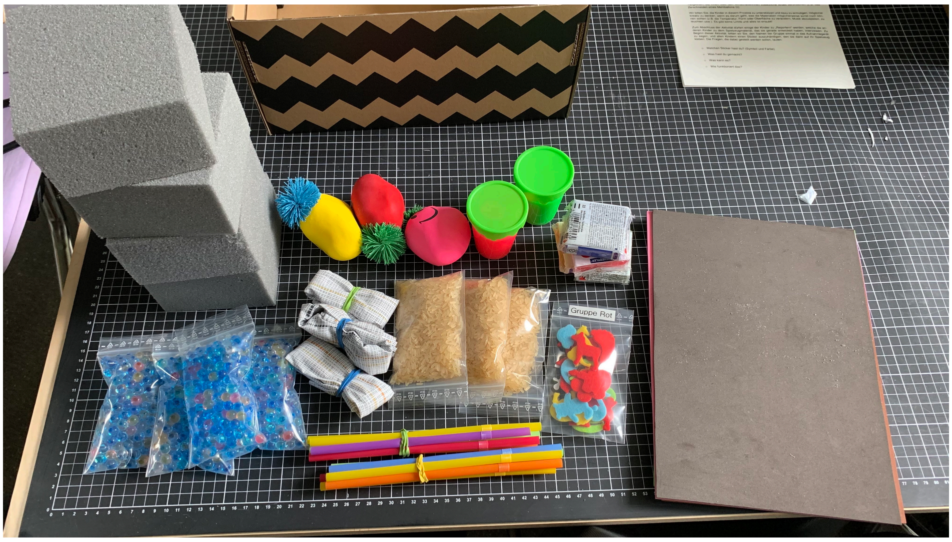

Contents of the probe activity 3. Material samples

	TYPE OF PROBE	PROBE CONTENTS (MATERIALS)	INSTRUCTIONS	GUIDING QUESTIONS	PURPOSE	ESTIMATED DURATION	OUTCOME
1A	Introduction and Communicator Box	Microcontroller, buttons, Speaker, recordings, LEDs, trap door, paper foil for covering. The size of the box should not be limiting	Introduce the fictional narrative and explain why and how these probeactivities are structured	Not applicable	Introduce the story and the purpose of the probe activities	15 minutes	/
1B	Favourite play activities, objects and places inquiry	Digital cameras	The children on the planet do not know / have forgotten what play is. Can you help them rediscover it? What is your preferred play activity, toy, space?	What are your favourite spaces, play activities and toys?	Insights on what toys or play activities they enjoy, in what spaces they enjoy these activities. to familiarise with them and their environment<br><br>Giving them a camera would provide a perspective from their eye level	25 minutes	Photographs
2	Light/shadow conditions inquiry	light torches or headlights, colour filters, paint or markers	Explore shadow conditions and find hidden elements in the provided activity sheets	How does light and shadow impact the way children interact with the space around them? What would be a good application area for implementing a light or shadow-based interface?	insights on how they would use a light-based game, how would they play with light and where?	40 minutes	Drawings
3	Material samples	flour balloons, bottle cleaner twisters, different types of fabric, water pearls, Straws, Nuts, Plush pompoms, Moosgummi, Slime, wool, rice, stickers with interaction properties, audio recordings of what they created	Pair materials and interactions for cool materials. What do these materials do? Can you give them special powers? what kind of powers would you pair them with? (powers-> affordances and action possibilities, inputs and outputs)	Which interaction possibilities do children envision when playing with objects (sensing and actuating)?	insights on existing materials in their spaces, their characteristics and the children's preferences	40 minutes	
4	Spatial Scale and proportions	large pieces of paper e.g., 2x1m, paint or other material to draw bodies. Digital cameras to capture creations	Create body outlines using large pieces of paper and markers and place them in their favourite locales	What are children's favourite spaces? How do they relate to their bodily proportions? How are the children using the spatial configuration of their classrooms, corridors, outdoors? Do they have specific places for specific activities? What are those activities? What sort of meaning do these places hold? What sort of modalities are allocated to each of these spaces?	insights on scale and bodies in space, how they perceive size, spatial cognition of children. Empathising with them on the way they view their space.	40 minutes	Photographs
5A	Sound Inquiry	Voice recorders	On the kinderplanet, the sounds have disappeared! Map spaces with sounds that you would like to hear and not hear, make your own sounds for the children's world.	What kind of sounds are there? What kind of sounds they like and dislike? How would they describe sound in the space?	insights on sounds at the kindergarten, insights on volume, preferences and dislikes and how they perceive sounds	40 minutes	Sound recordings
5B	Cease of play activity	Voice recorders	It's time to finish the play activity, now you have to clean up	How do children react to change of activities? How do they react to the pause of play? What do they think about time and duration?	Insights on children's sense of time and continuity as well as the cease of the current activity.	40 minutes	Sound recordings, Pedagogues Notes
6	Probes feedback	Likert-style stickers with emoticons/ smileys for emotions/ preferences	What did they like the most and what they didn't	What worked well, what didn't and why regarding the probe activities?	insights on activities that worked well, matched their preferences, or did not.	40 minutes	Pedagogues Notes

RESEARCH ACTIVITY CONCEPT

ACTIVITY AIM

To initiate a discussion with young children that attend kindergartens regarding favourite spaces, activity preferences, environmental aspects of their kindergarten, and elements of their play activities and toys. Through this discussion we aim at gathering a deeper understanding of the spatial context and the play activities of this user group by reducing barriers such as social distance, age gap, and language. We are particularly interested in learning about the spaces, routines and activities of young children children at kindergartens regarding socialisation, development (cognitive, motor, other), play, and learning. This deeper understanding will feed into future interaction design interventions as a form of inspiration.
We plan to inform the children and teachers on how the data they will provide us will be handled and inform design research.

Expected Contribution

This research activity contributes to already existing research in the field of Child-Computer Interaction (CCI) since it seeks to understand an under-researched age group, young children in kindergartens. We expect to expand the knowledge on applying and deploying cultural fiction probes for this particular population group. A further contribution would be towards collecting spatial, environmental data in a kindergarten environment from the children's perspective.

Duration: 3 weeks

Participants: Kindergarten children aged 3-6 years old

Schedule: See external document

Research Team: [names]

Material needed:

•Informed consent form
•Information sheets
•Cultural Fiction Probes Packages

Guiding Research Questions:

- How can we adapt cultural probes for young children in kindergartens?
  - Which activities to include and which to exclude?
  - How can we design an engaging cultural probes package for young children in kindergartens?
  - How to accommodate answers from different age groups depending on their development stage at the time?
- How can we design cultural probes for young children in a way that will require as little assistance from an adult as possible?
  - How can we design an intergenerational cultural probes package?
