# Supplementary material for: Uncovering placemaking needs with(in) a kindergarten community: a cross-disciplinary approach to participatory design
Source: Front Psychol. 2023 Jun 20;14:1126276. doi: 10.3389/fpsyg.2023.1126276 (PMC10319412; doi:10.3389/fpsyg.2023.1126276)
Supplement: Supplementary Data Sheet S3 — Interview material for building evaluation (English translation). [file Data_Sheet_3.PDF]

Interview guide: Building tour of the kindergarten

Beginning

- Introduction of the interviewer (name, function)
- Thanks for the interviewee's participation
- Introduction to the research project and the research context (the construction project, interests of staff and children to be considered, studies on spatial experience and satisfaction)
- Information about data protection / anonymization of data for evaluation and publication
- Explanation of the interview procedure, instruction and further steps
- Clarification of open questions
- Obtaining consent for documentation (photographs, statements)

Procedure	Interview during building tour, joint protocol / photo documentation<br><br>(1) interviewee guides through the building (areas of the kindergarten)<br>(2) for each area: interviewee introduces the area<br>(3) for each area: interviewer asks the core questions and prepares area protocol<br>(4) for each area: joint review of the area protocol<br>(5) for each area: photo documentation of the area by the interviewer

Building tour and interview

- Visit of areas
- Questioning per area, core questions (support by examples if necessary)
- Logging and joint review of the protocol for each area visited, and feedback, amendments and/or corrections by interviewee
- Photo documentation

Core questions	(1) Which spatial features are beneficial for teachers and children, why?<br>(2) Which spatial features are hindering for teachers and children, why?
Examples	• Acoustic conditions (e.g., noise level)<br>• Lighting conditions (e.g., artificial light, daylight, sunshade, glare shield)<br>• Temperature conditions<br>• Air quality / drafts (e.g., ventilation)<br>• Furnishing / design<br>• Building materials / structural condition (e.g., wood, green areas)<br>• Socially connecting / creativity promoting spatial features<br>• Spatial quality in general – which spatial features are good / bad for teachers and children, why?

End

- Thanks for the interviewee's participation
- Hand over survey forms for the staff, agreement on date to collect the survey forms
- Announcement of presentation of findings (project consortium meeting)
- Farewell

Interview and building tour protocol

Location	
Interview with	
Interviewer	
Date	
Start	
End	
Study slot	

Areas (amend if necessary)

#01	
#02	
#03	
#04	
#05	
#06	
#07	
#08	
#09	
#10	
#11	
#12	
#13	
#14	
#15	

**Area**	**#**

Beneficial features

What do you find particularly good about this area?

Hindering features

What do you find particularly bad about this area?

<u>Examples</u>

Acoustic conditions / noise level • lighting conditions • temperature conditions • air quality / draft • furnishing / design • building materials / structural condition • socially connecting / creativity promoting features • spatial quality in general – good / bad for teachers and children, why?

**Area #**

Beneficial features

What do you find particularly good about this area?

Hindering features

What do you find particularly bad about this area?

<u>Examples</u>

Acoustic conditions / noise level • lighting conditions • temperature conditions • air quality / draft • furnishing / design • building materials / structural condition • socially connecting / creativity promoting features • spatial quality in general – good / bad for teachers and children, why?

**Area #**

Beneficial features

What do you find particularly good about this area?

Hindering features

What do you find particularly bad about this area?

<u>Examples</u>

Acoustic conditions / noise level • lighting conditions • temperature conditions • air quality / draft • furnishing / design • building materials / structural condition • socially connecting / creativity promoting features • spatial quality in general – good / bad for teachers and children, why?

**Area**	**#**

Beneficial features

What do you find particularly good about this area?

Hindering features

What do you find particularly bad about this area?

<u>Examples</u>

Acoustic conditions / noise level • lighting conditions • temperature conditions • air quality / draft • furnishing / design • building materials / structural condition • socially connecting / creativity promoting features • spatial quality in general – good / bad for teachers and children, why?

**Area** #

Beneficial features

What do you find particularly good about this area?

Hindering features

What do you find particularly bad about this area?

<u>Examples</u>

Acoustic conditions / noise level • lighting conditions • temperature conditions • air quality / draft • furnishing / design • building materials / structural condition • socially connecting / creativity promoting features • spatial quality in general – good / bad for teachers and children, why?

**Area** #

Beneficial features

What do you find particularly good about this area?

Hindering features

What do you find particularly bad about this area?

<u>Examples</u>

Acoustic conditions / noise level • lighting conditions • temperature conditions • air quality / draft • furnishing / design • building materials / structural condition • socially connecting / creativity promoting features • spatial quality in general – good / bad for teachers and children, why?

**Area #**

Beneficial features

What do you find particularly good about this area?

Hindering features

What do you find particularly bad about this area?

<u>Examples</u>

Acoustic conditions / noise level • lighting conditions • temperature conditions • air quality / draft • furnishing / design • building materials / structural condition • socially connecting / creativity promoting features • spatial quality in general – good / bad for teachers and children, why?

**Area** **#**

Beneficial features

What do you find particularly good about this area?

Hindering features

What do you find particularly bad about this area?

Examples

Acoustic conditions / noise level • lighting conditions • temperature conditions • air quality / draft • furnishing / design • building materials / structural condition • socially connecting / creativity promoting features • spatial quality in general – good / bad for teachers and children, why?

**Area**   **#**

Beneficial features

What do you find particularly good about this area?

Hindering features

What do you find particularly bad about this area?

<u>Examples</u>

Acoustic conditions / noise level • lighting conditions • temperature conditions • air quality / draft • furnishing / design • building materials / structural condition • socially connecting / creativity promoting features • spatial quality in general – good / bad for teachers and children, why?

**Area**	**#**

Beneficial features

What do you find particularly good about this area?

Hindering features

What do you find particularly bad about this area?

Examples

Acoustic conditions / noise level • lighting conditions • temperature conditions • air quality / draft • furnishing / design • building materials / structural condition • socially connecting / creativity promoting features • spatial quality in general – good / bad for teachers and children, why?

**Area #**

Beneficial features

What do you find particularly good about this area?

Hindering features

What do you find particularly bad about this area?

<u>Examples</u>

Acoustic conditions / noise level • lighting conditions • temperature conditions • air quality / draft • furnishing / design • building materials / structural condition • socially connecting / creativity promoting features • spatial quality in general – good / bad for teachers and children, why?

**Area** #

Beneficial features

What do you find particularly good about this area?

Hindering features

What do you find particularly bad about this area?

Examples

Acoustic conditions / noise level • lighting conditions • temperature conditions • air quality / draft • furnishing / design • building materials / structural condition • socially connecting / creativity promoting features • spatial quality in general – good / bad for teachers and children, why?

**Area #**

Beneficial features

What do you find particularly good about this area?

Hindering features

What do you find particularly bad about this area?

Examples

Acoustic conditions / noise level • lighting conditions • temperature conditions • air quality / draft • furnishing / design • building materials / structural condition • socially connecting / creativity promoting features • spatial quality in general – good / bad for teachers and children, why?

**Area #**

Beneficial features

What do you find particularly good about this area?

Hindering features

What do you find particularly bad about this area?

<u>Examples</u>

Acoustic conditions / noise level • lighting conditions • temperature conditions • air quality / draft • furnishing / design • building materials / structural condition • socially connecting / creativity promoting features • spatial quality in general – good / bad for teachers and children, why?

**Area** #

Beneficial features

What do you find particularly good about this area?

Hindering features

What do you find particularly bad about this area?

Examples

Acoustic conditions / noise level • lighting conditions • temperature conditions • air quality / draft • furnishing / design • building materials / structural condition • socially connecting / creativity promoting features • spatial quality in general – good / bad for teachers and children, why?
