# Supplementary material for: Uncovering placemaking needs with(in) a kindergarten community: a cross-disciplinary approach to participatory design
Source: Front Psychol. 2023 Jun 20;14:1126276. doi: 10.3389/fpsyg.2023.1126276 (PMC10319412; doi:10.3389/fpsyg.2023.1126276)
Supplement: Supplementary Data Sheet S4 — Survey material for building evaluation (English translation). [file Data_Sheet_4.PDF]

Staff survey: Experience of space in the kindergarten

Dear kindergarten teachers,

a spart of our joint project, we investigate your experience and well-being in the areas you use in the kindergarten building. We are particularly interested in the spatial features you consider to be especially beneficial or hindering for you and the children you care for. Please, describe below all the areas that you use at your workplace (e.g., group room, gym, creative area, eating area, lavatories, entrance area, wardrobes / cloakrooms, garden, and outdoor play areas). The more detailed your description the better!

Please indicate in the corresponding text box which area you are describing and answer the following two questions for each area:

(1) Which spatial features are <u>beneficial</u> for teachers and children, why?
(2) Which spatial features are <u>hindering</u> for teachers and children, why?

If possible, describe your personal experience. Think about how you experience certain spatial features such as:

- Acoustic conditions (e.g., noise level)
- Lighting conditions (e.g., artificial light, daylight, sunshade, glare shield)
- Temperature conditions
- Air quality / drafts (e.g., ventilation)
- Furnishing / design
- Building materials / structural condition (e.g., wood, green areas)
- Socially connecting / creativity promoting spatial features
- Spatial quality in general – which spatial features are good / bad for teachers and children, why?

Area ____

Beneficial features	Hindering features
What do you find particularly good about this area?	*What do you find particularly bad about this area?*

Area ____

Beneficial features	Hindering features
What do you find particularly good about this area?	*What do you find particularly bad about this area?*

Area ____

Beneficial features	Hindering features
What do you find particularly good about this area?	*What do you find particularly bad about this area?*

Area ____

**Beneficial features**	**Hindering features**
What do you find particularly good about this area?	*What do you find particularly bad about this area?*

Area ____

**Beneficial features**	**Hindering features**
What do you find particularly good about this area?	*What do you find particularly bad about this area?*

Area ____

**Beneficial features**	**Hindering features**
What do you find particularly good about this area?	*What do you find particularly bad about this area?*

Area ____

Beneficial features	Hindering features
What do you find particularly good about this area?	*What do you find particularly bad about this area?*

Area ____

Beneficial features	Hindering features
What do you find particularly good about this area?	*What do you find particularly bad about this area?*

Area ____

Beneficial features	Hindering features
What do you find particularly good about this area?	*What do you find particularly bad about this area?*

Area ____

**Beneficial features**	**Hindering features**
What do you find particularly good about this area?	*What do you find particularly bad about this area?*

Area ____

**Beneficial features**	**Hindering features**
What do you find particularly good about this area?	*What do you find particularly bad about this area?*

Area ____

**Beneficial features**	**Hindering features**
What do you find particularly good about this area?	*What do you find particularly bad about this area?*

Area ______________________________________

**Beneficial features**	**Hindering features**
What do you find particularly good about this area?	*What do you find particularly bad about this area?*

Area ______________________________________

**Beneficial features**	**Hindering features**
What do you find particularly good about this area?	*What do you find particularly bad about this area?*

Thank you for your participation!
