# Supplementary figures and images for: Uncovering placemaking needs with(in) a kindergarten community: a cross-disciplinary approach to participatory design
Source: Front Psychol. 2023 Jun 20;14:1126276. doi: 10.3389/fpsyg.2023.1126276 (PMC10319412; doi:10.3389/fpsyg.2023.1126276)

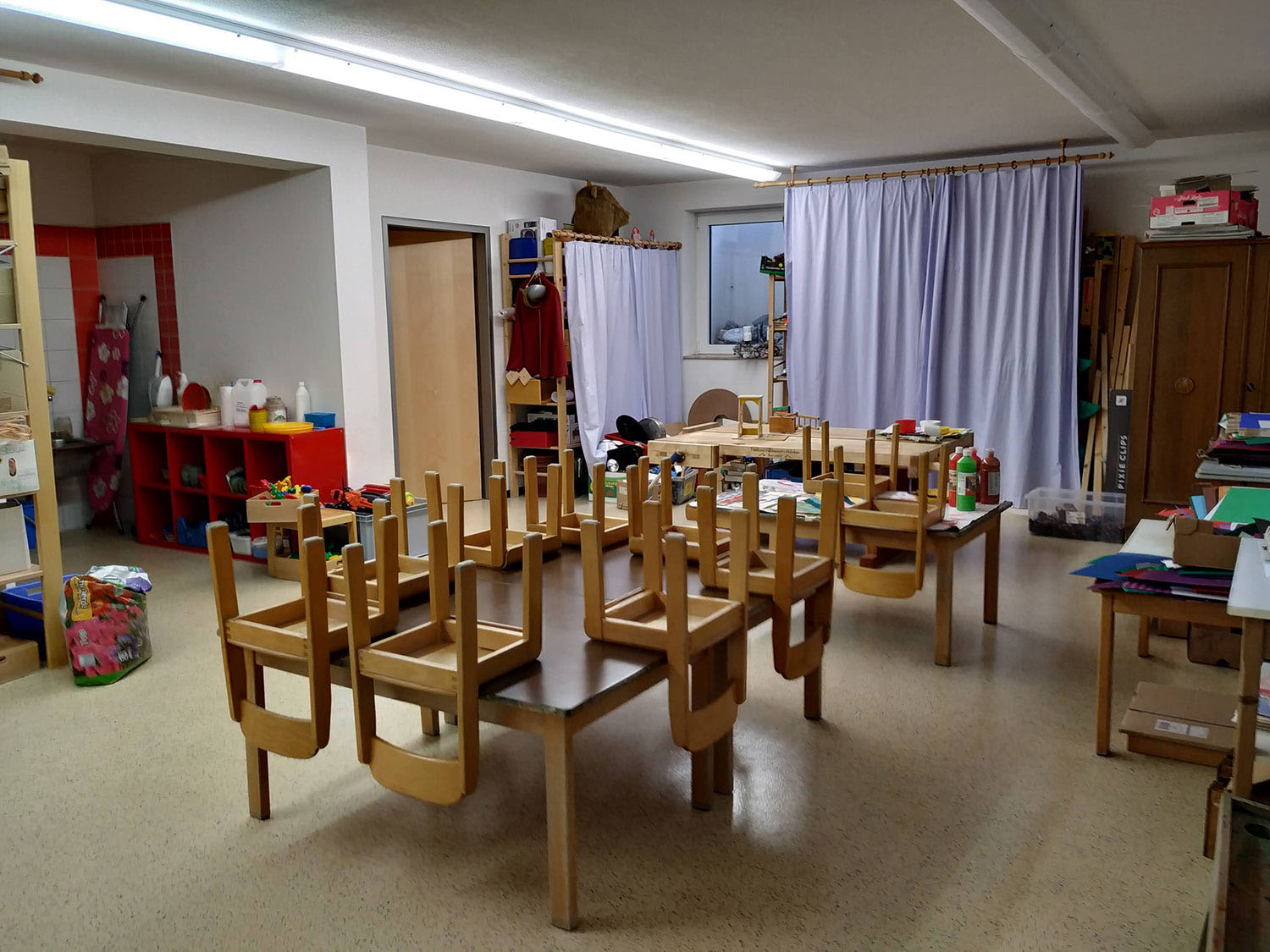

Supplement: Supplementary Data Sheet S5 — Photographic record of building tour. [file Data_Sheet_5.zip › activity areas/creative-area_1.jpg]

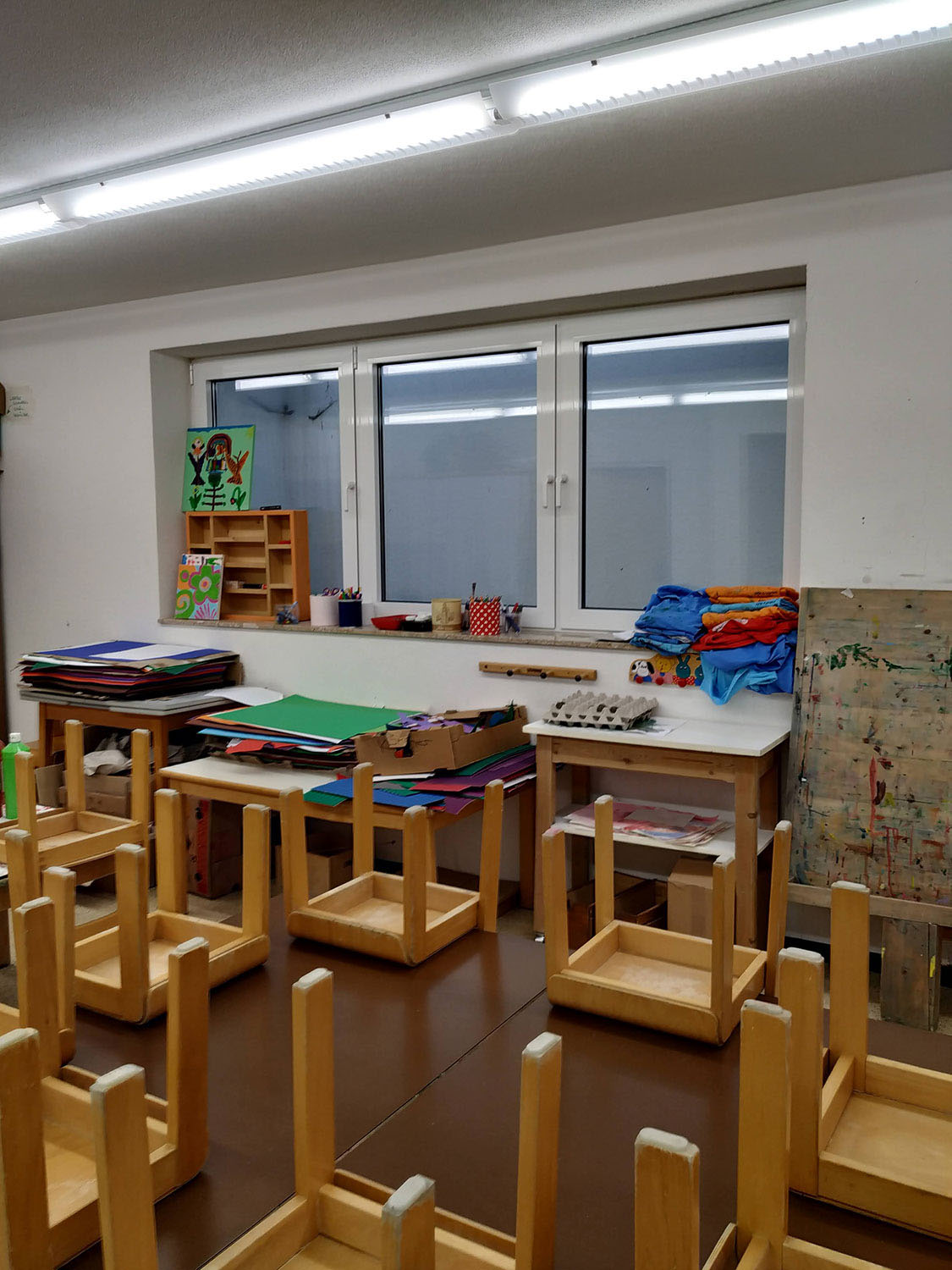

Supplement: Supplementary Data Sheet S5 — Photographic record of building tour. [file Data_Sheet_5.zip › activity areas/creative-area_2.jpg]

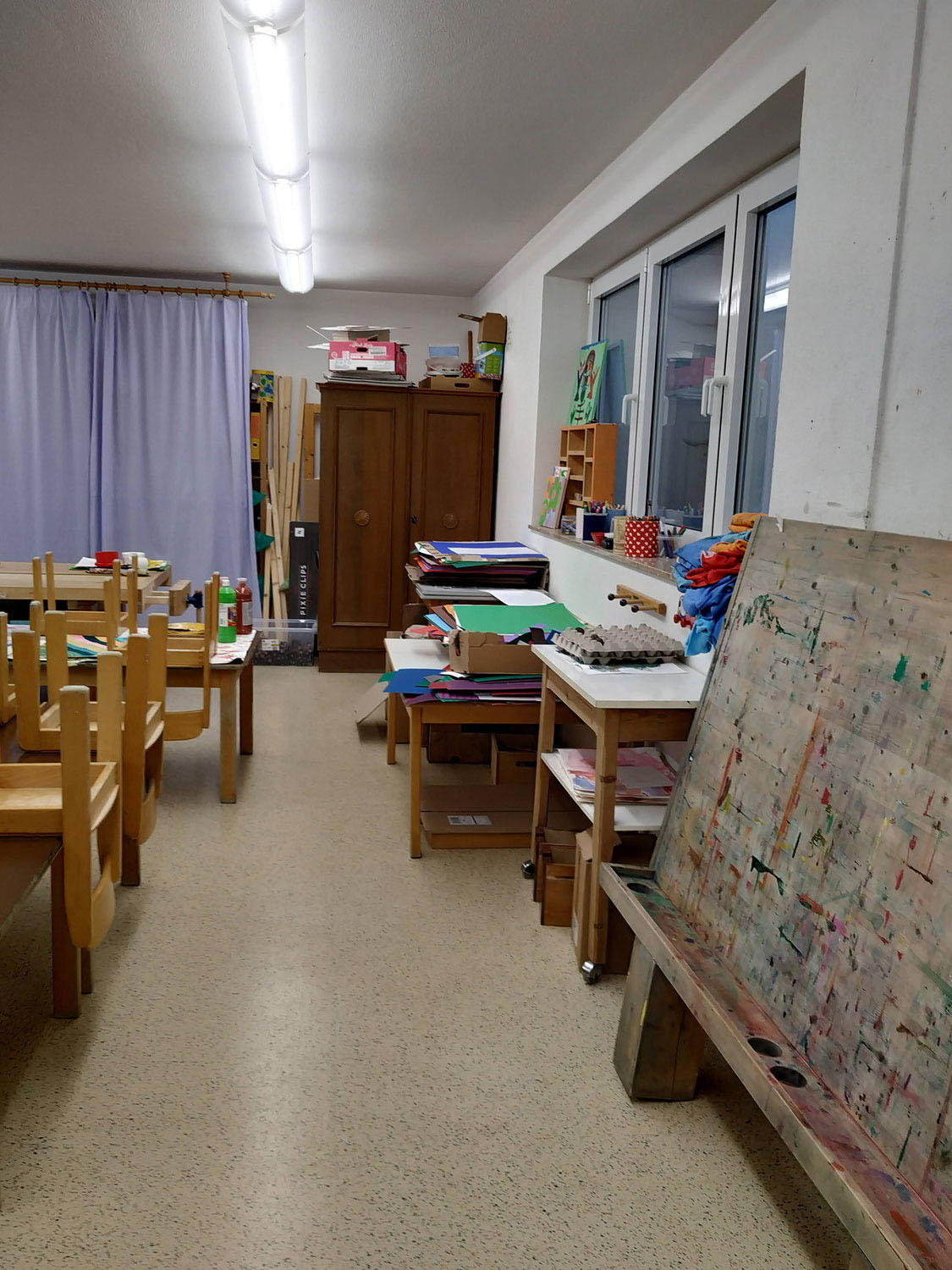

Supplement: Supplementary Data Sheet S5 — Photographic record of building tour. [file Data_Sheet_5.zip › activity areas/creative-area_3.jpg]

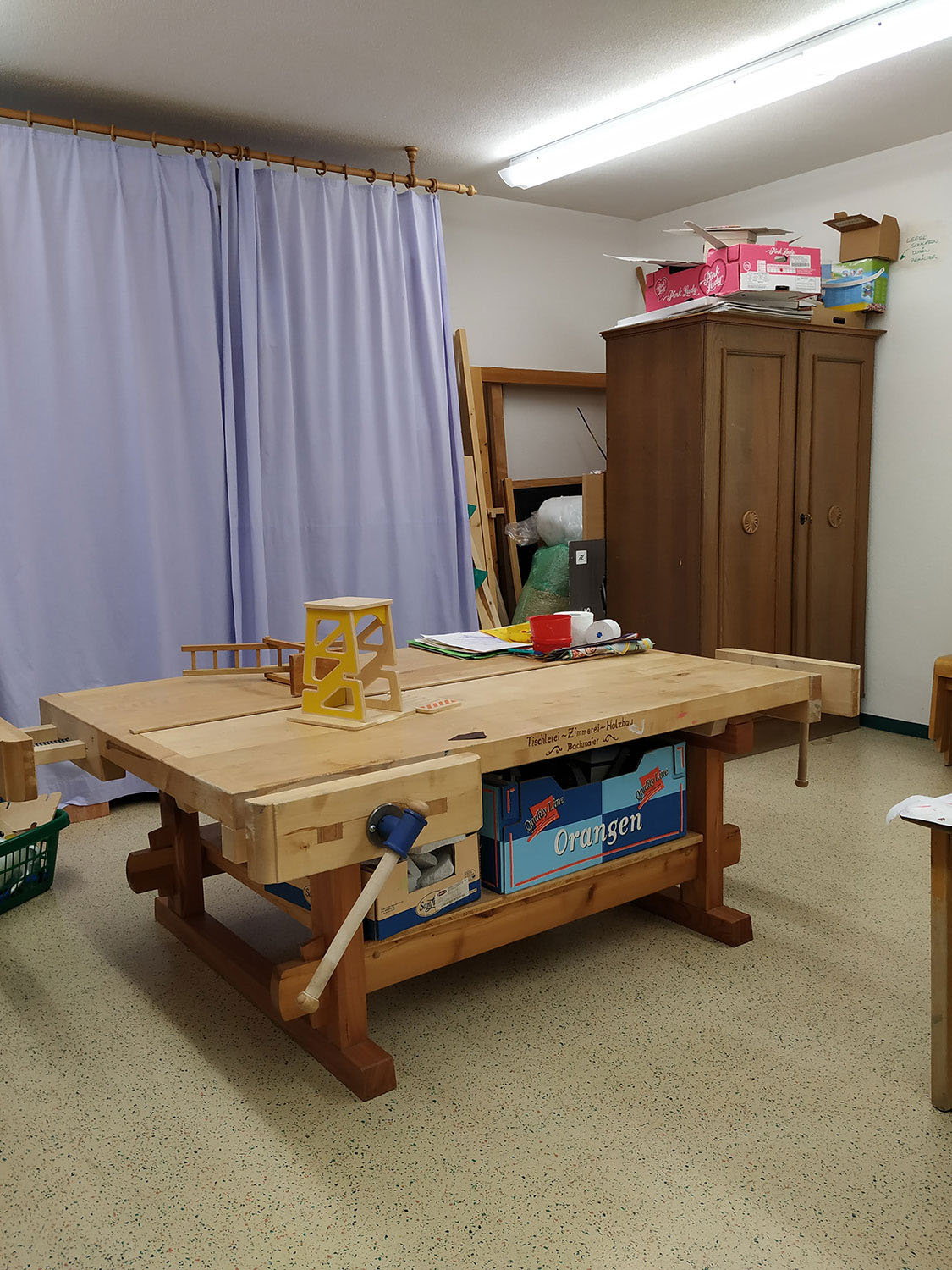

Supplement: Supplementary Data Sheet S5 — Photographic record of building tour. [file Data_Sheet_5.zip › activity areas/creative-area_4.jpg]

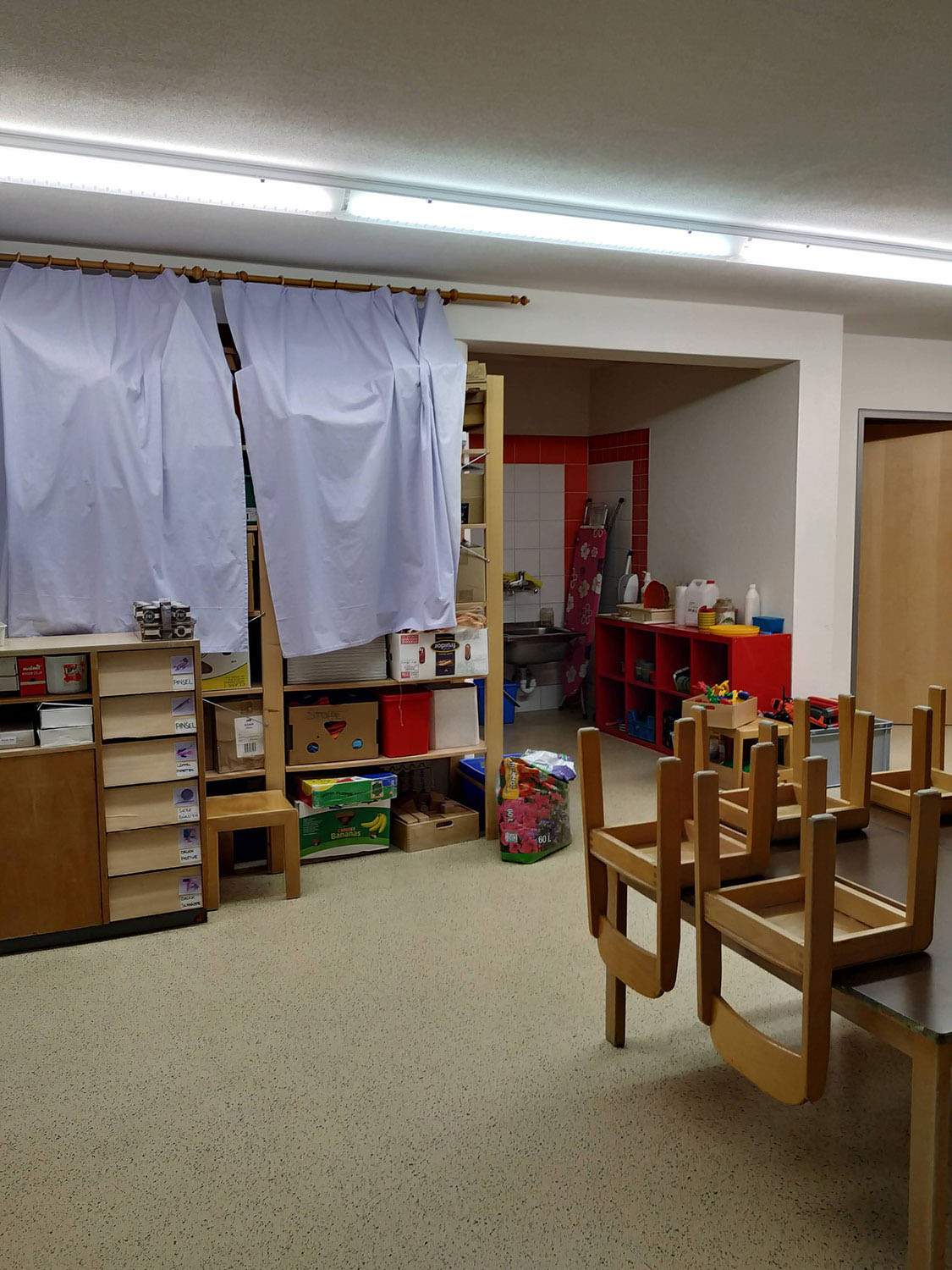

Supplement: Supplementary Data Sheet S5 — Photographic record of building tour. [file Data_Sheet_5.zip › activity areas/creative-area_5.jpg]

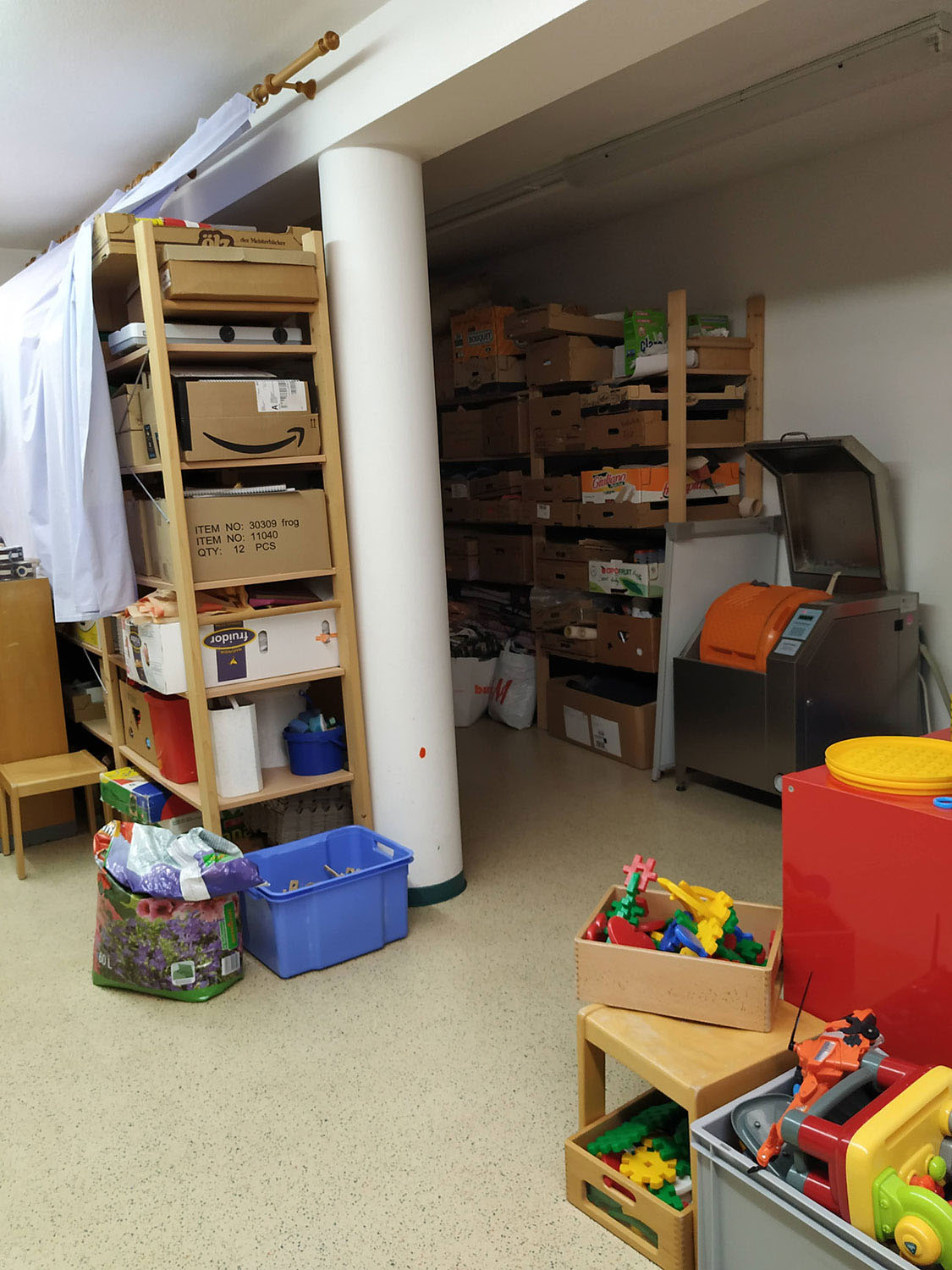

Supplement: Supplementary Data Sheet S5 — Photographic record of building tour. [file Data_Sheet_5.zip › activity areas/creative-area_6.jpg]

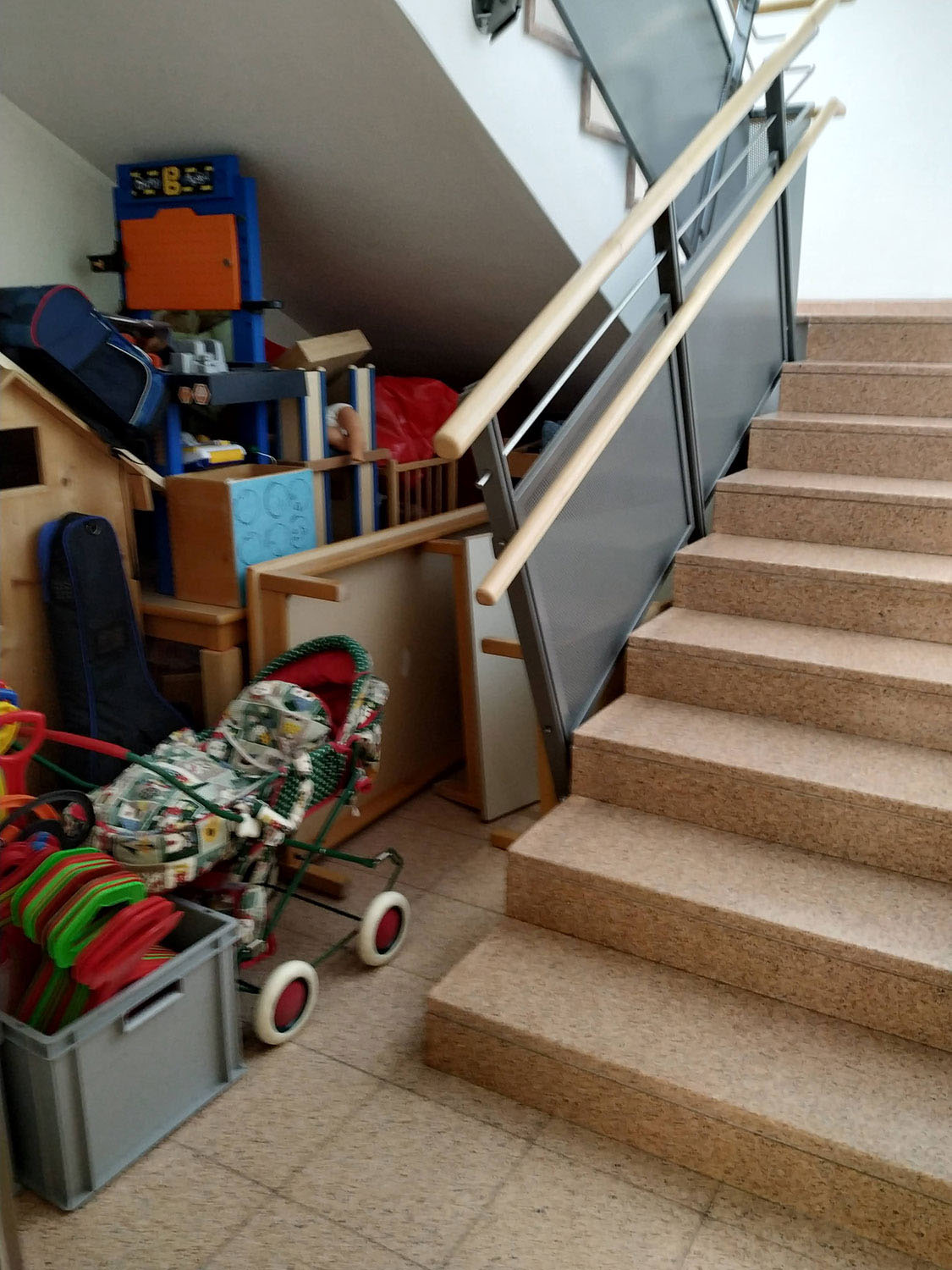

Supplement: Supplementary Data Sheet S5 — Photographic record of building tour. [file Data_Sheet_5.zip › activity areas/creative-area_7.jpg]

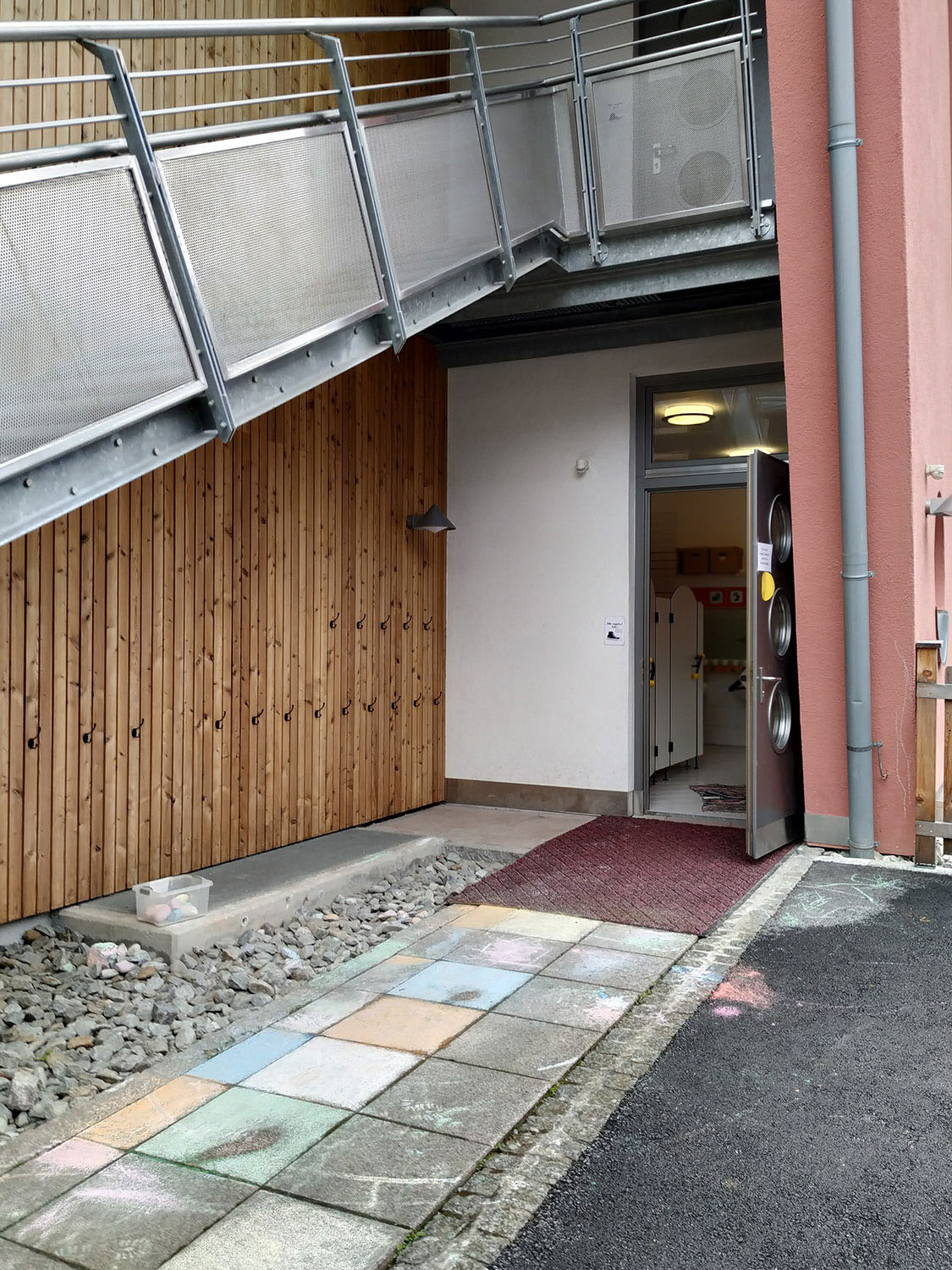

Supplement: Supplementary Data Sheet S5 — Photographic record of building tour. [file Data_Sheet_5.zip › activity areas/garden_1.jpg]

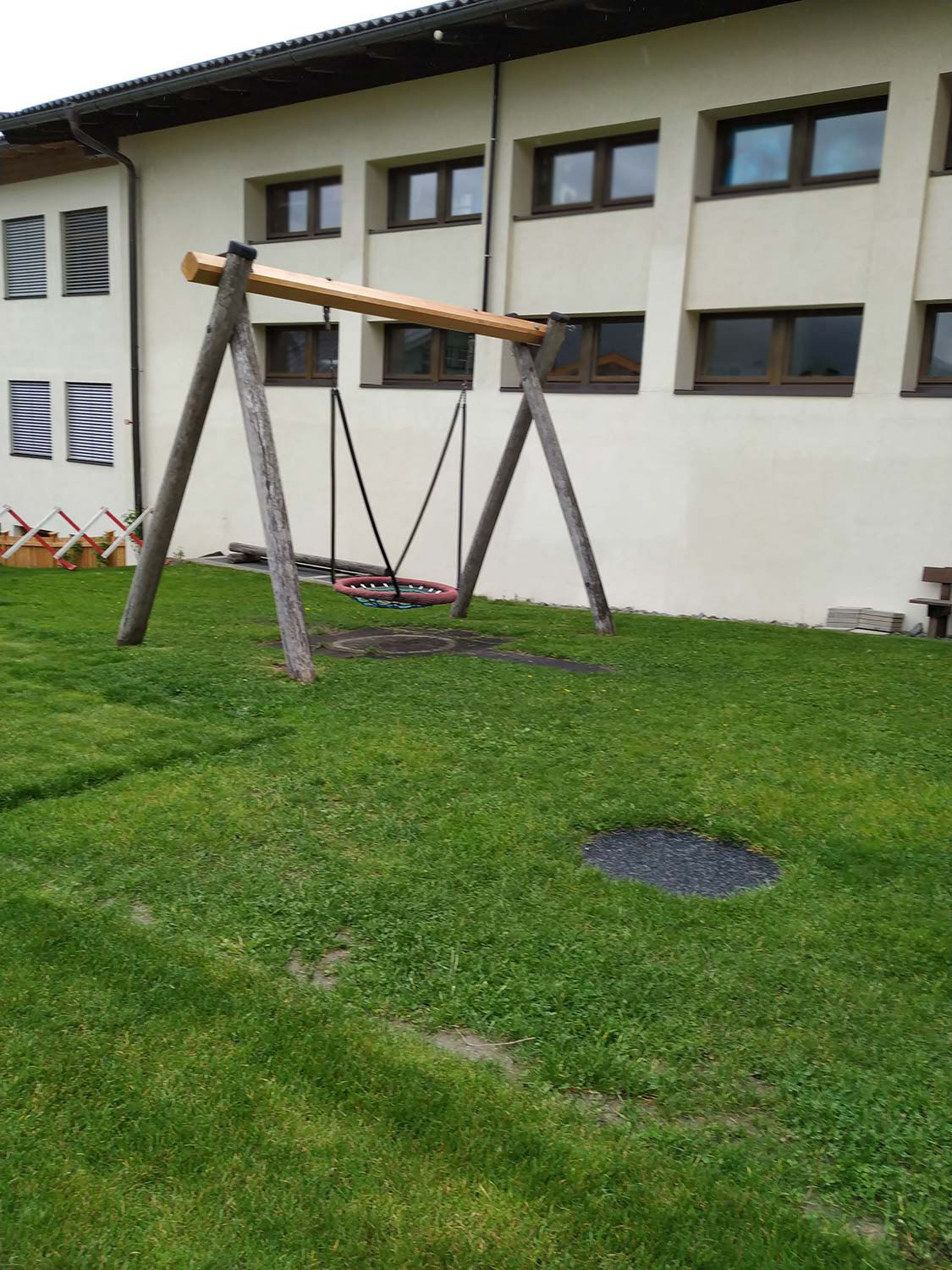

Supplement: Supplementary Data Sheet S5 — Photographic record of building tour. [file Data_Sheet_5.zip › activity areas/garden_10.jpg]

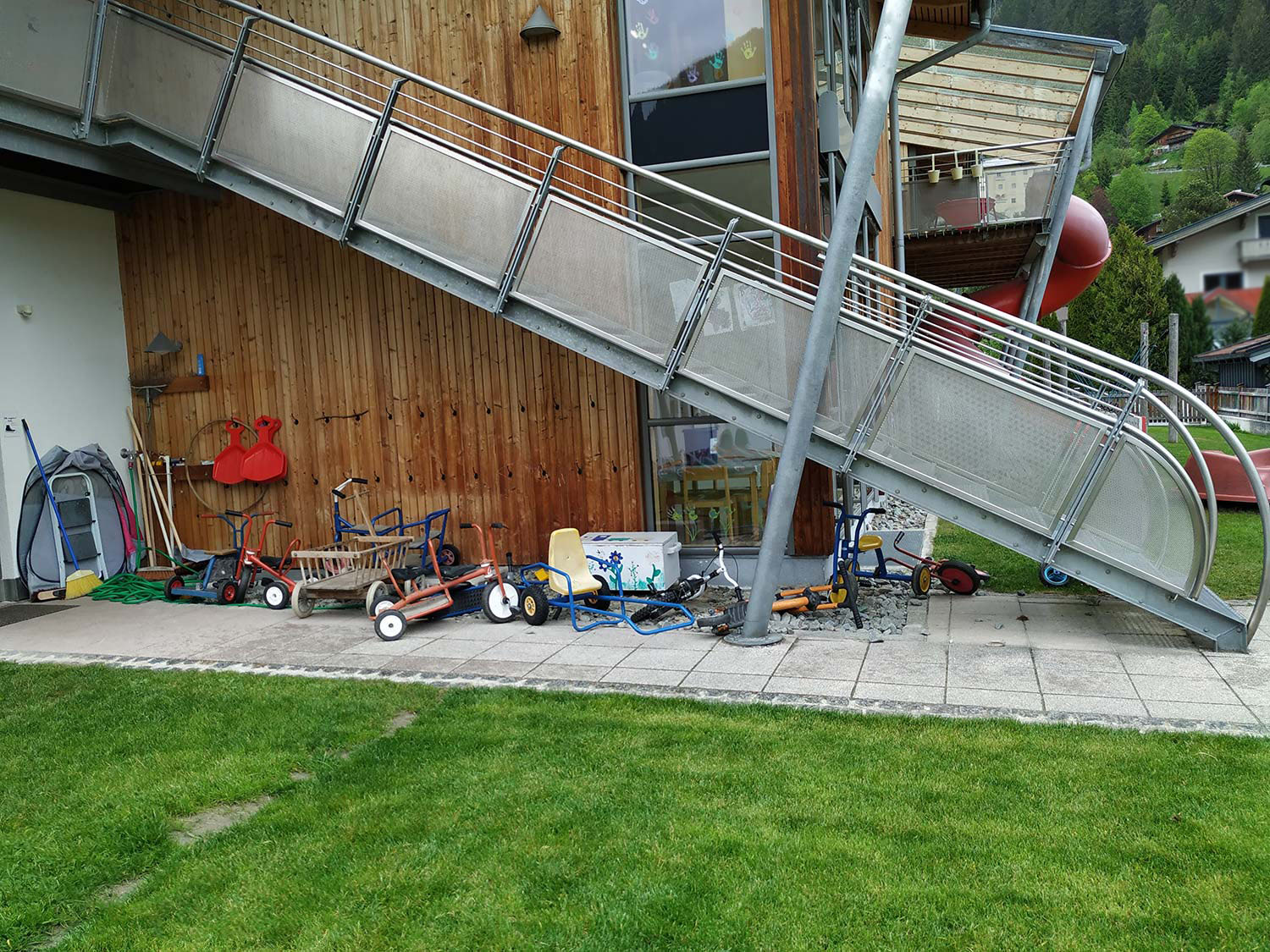

Supplement: Supplementary Data Sheet S5 — Photographic record of building tour. [file Data_Sheet_5.zip › activity areas/garden_11.jpg]

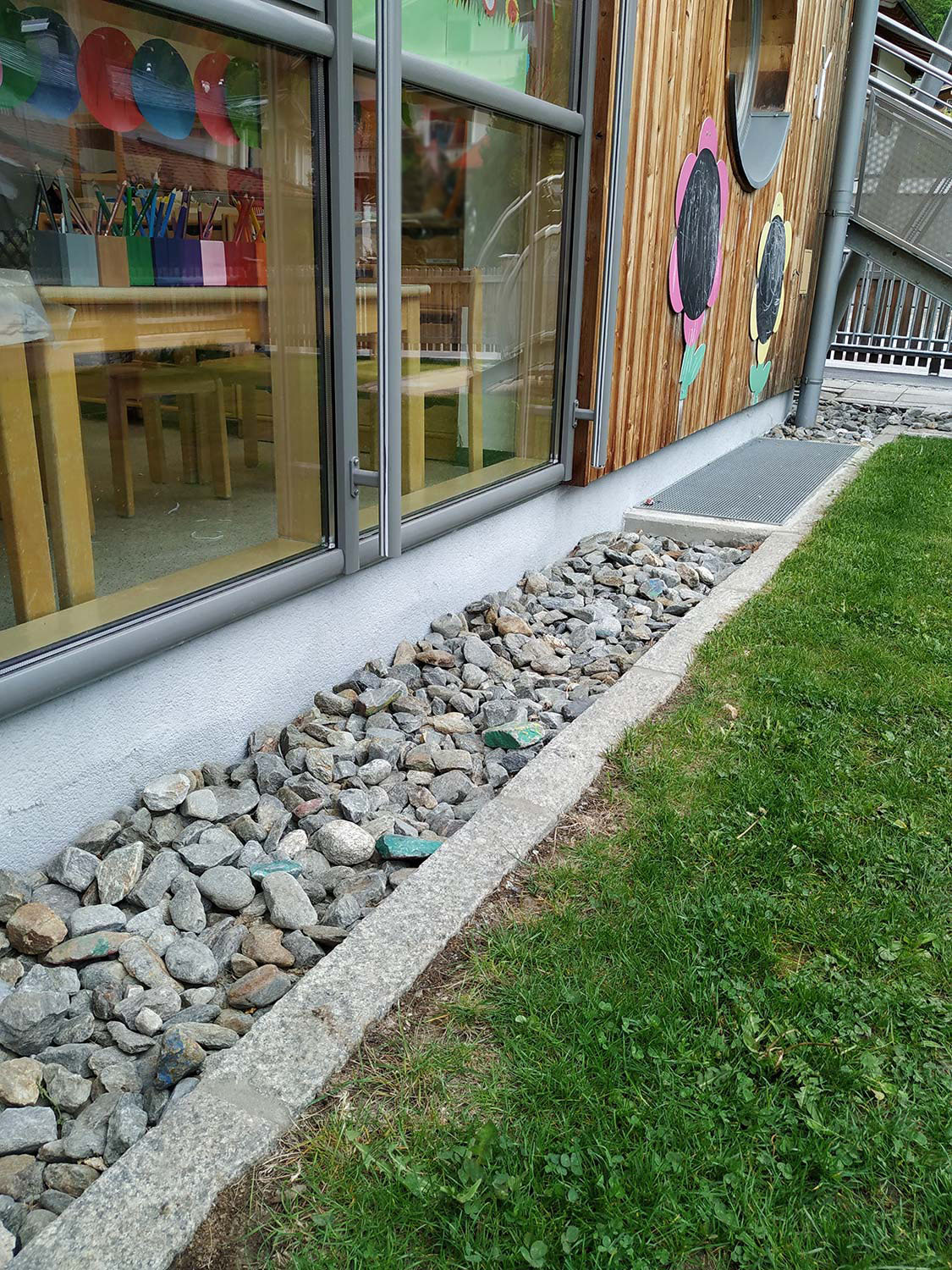

Supplement: Supplementary Data Sheet S5 — Photographic record of building tour. [file Data_Sheet_5.zip › activity areas/garden_12.jpg]

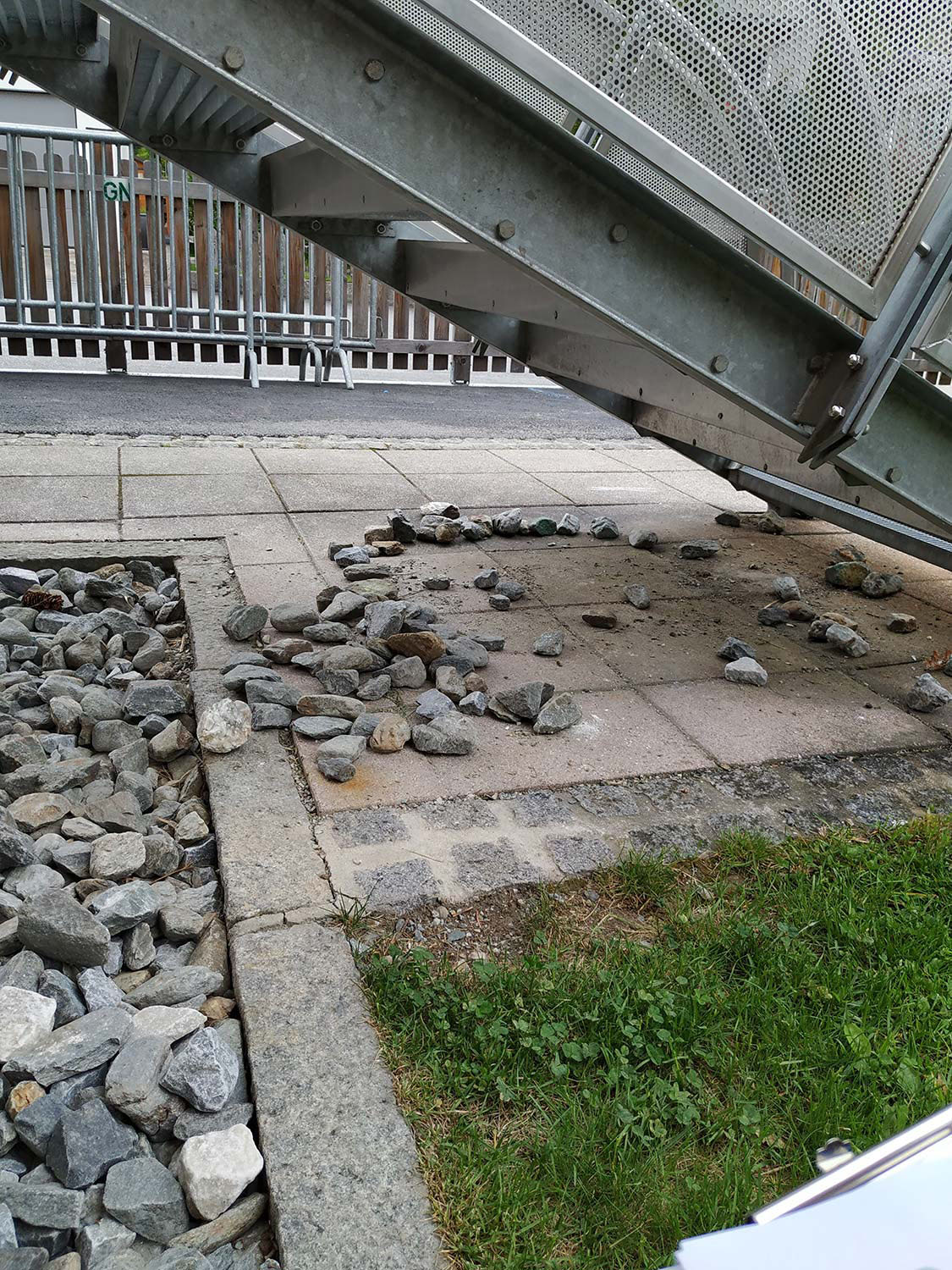

Supplement: Supplementary Data Sheet S5 — Photographic record of building tour. [file Data_Sheet_5.zip › activity areas/garden_13.jpg]

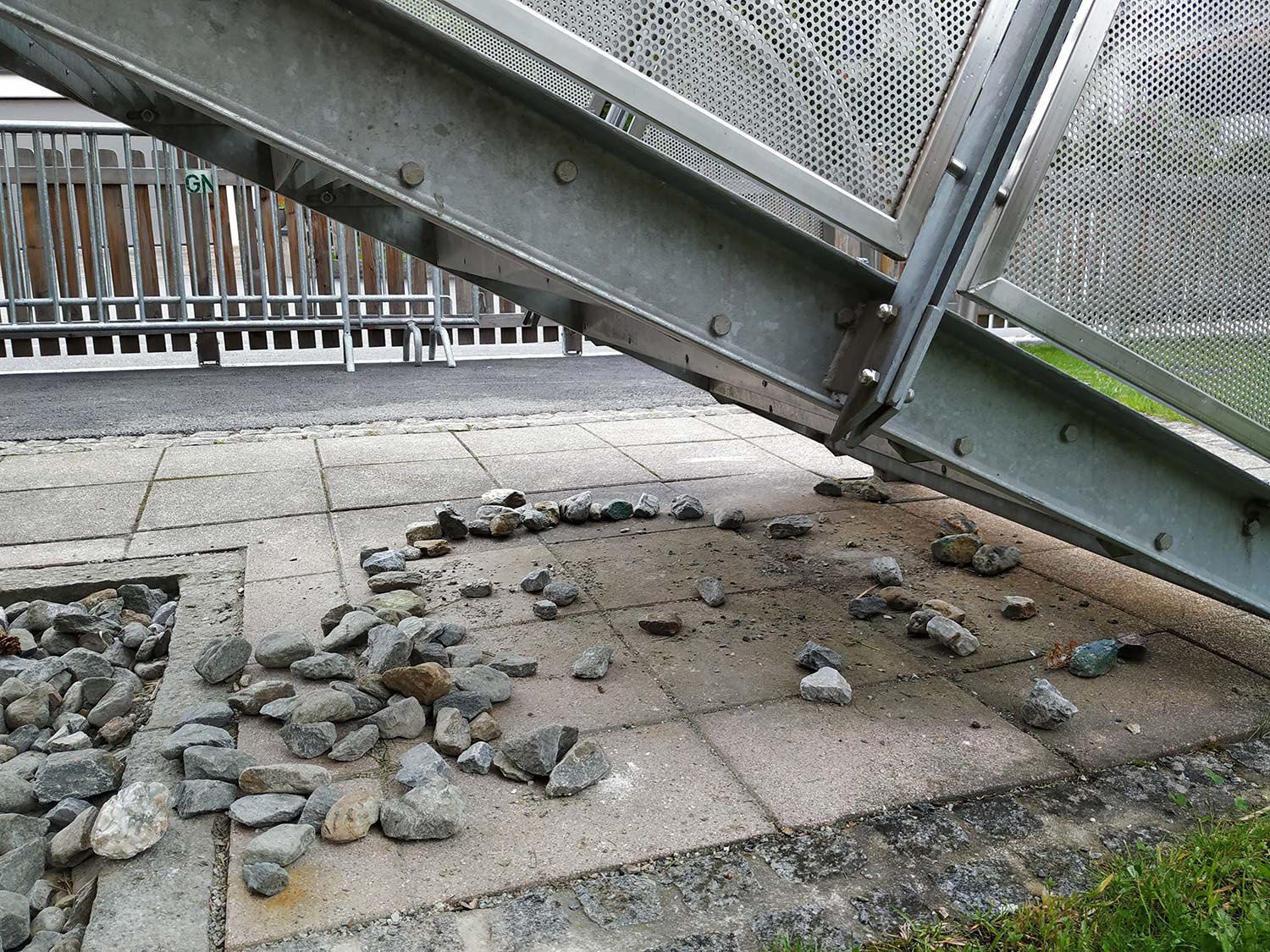

Supplement: Supplementary Data Sheet S5 — Photographic record of building tour. [file Data_Sheet_5.zip › activity areas/garden_14.jpg]

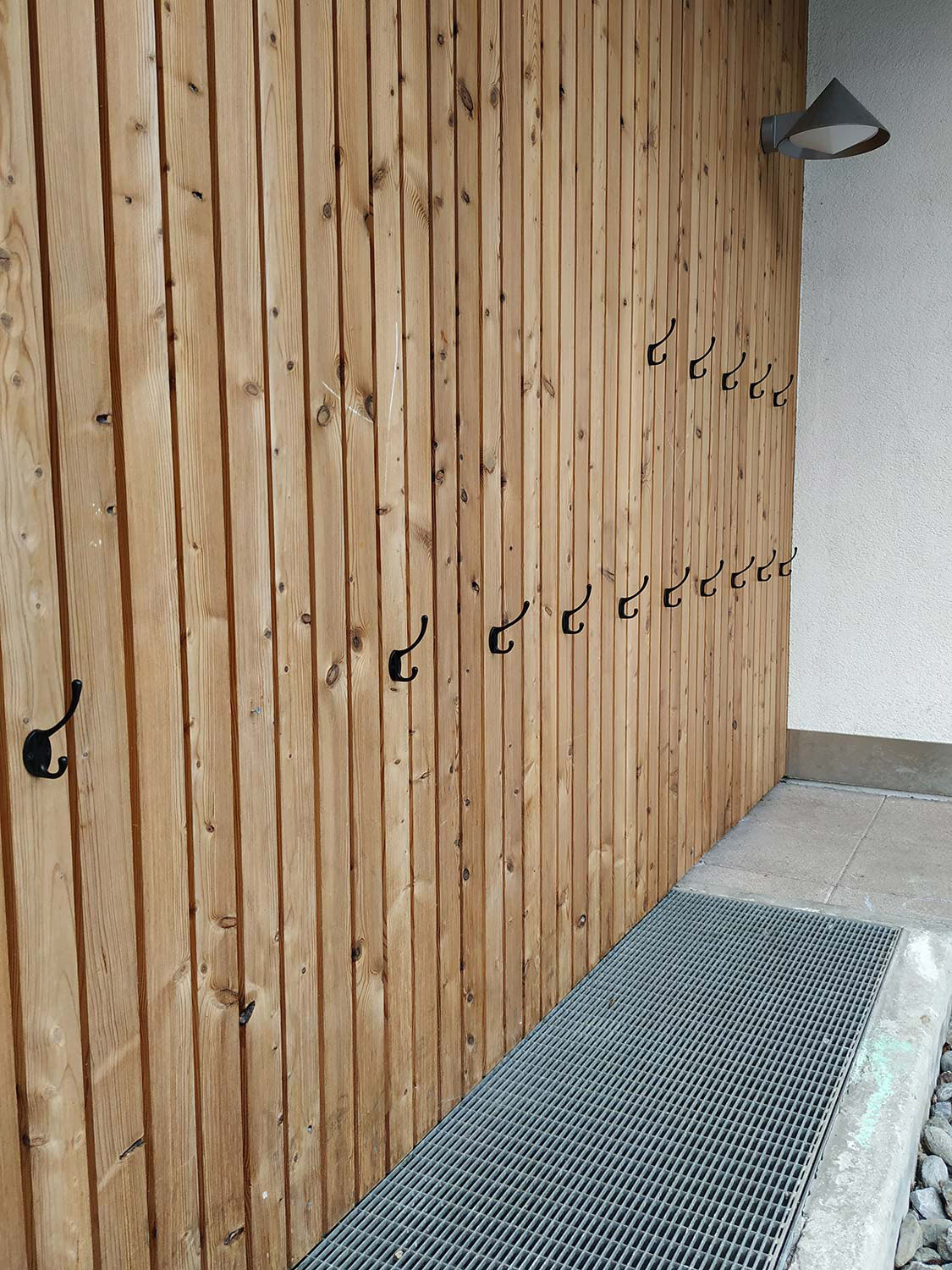

Supplement: Supplementary Data Sheet S5 — Photographic record of building tour. [file Data_Sheet_5.zip › activity areas/garden_15.jpg]

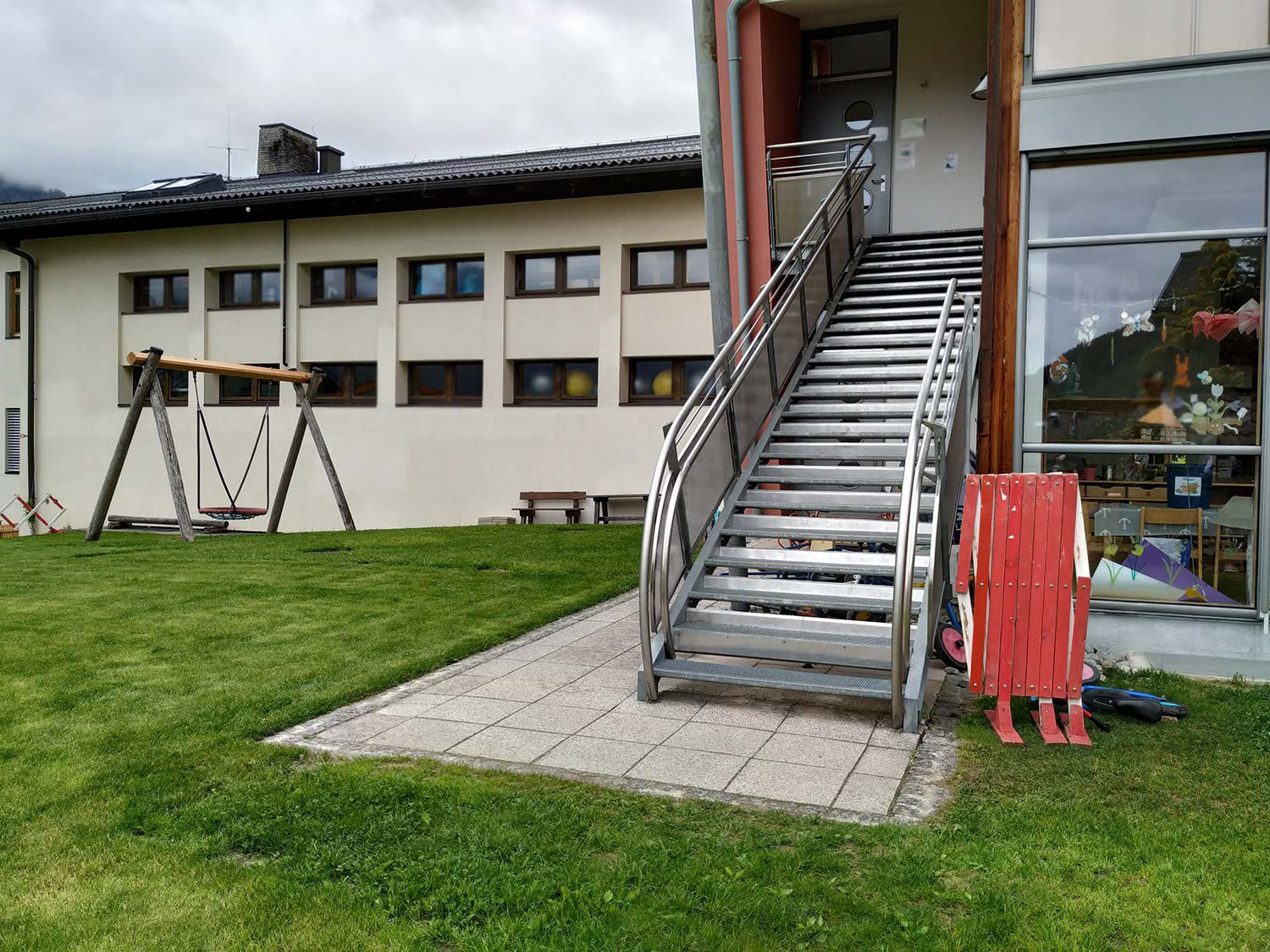

Supplement: Supplementary Data Sheet S5 — Photographic record of building tour. [file Data_Sheet_5.zip › activity areas/garden_16.jpg]

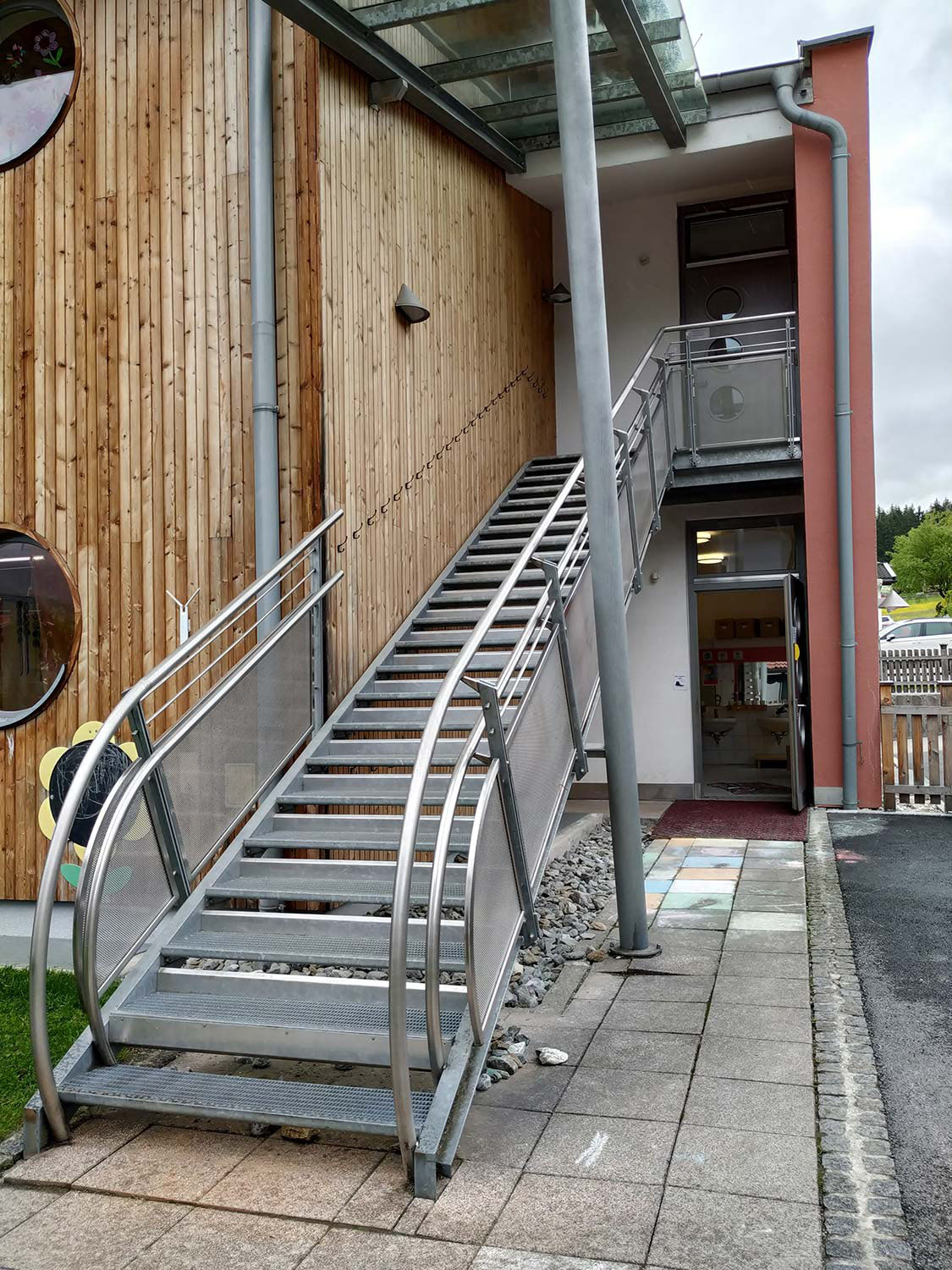

Supplement: Supplementary Data Sheet S5 — Photographic record of building tour. [file Data_Sheet_5.zip › activity areas/garden_17.jpg]

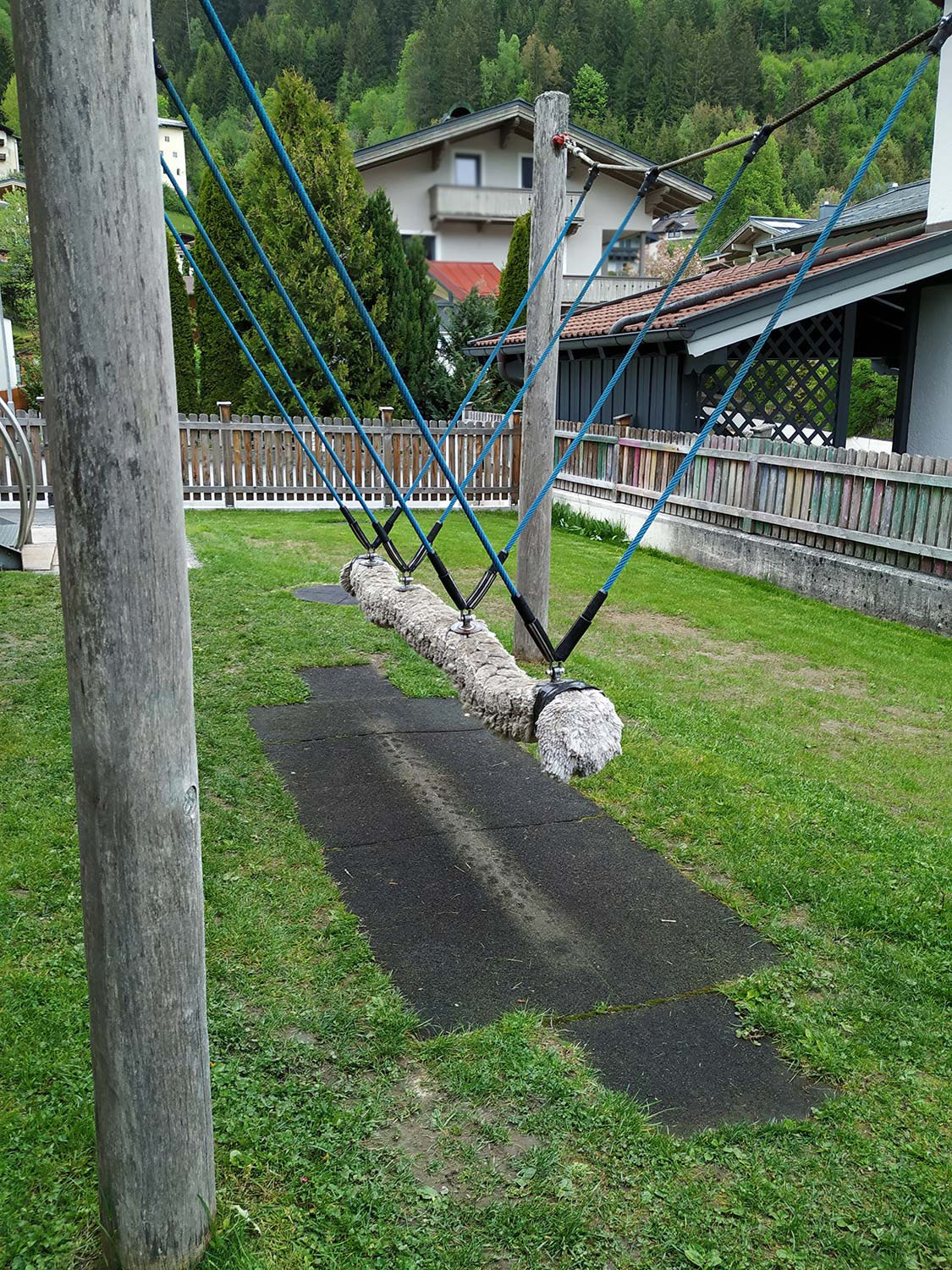

Supplement: Supplementary Data Sheet S5 — Photographic record of building tour. [file Data_Sheet_5.zip › activity areas/garden_2.jpg]

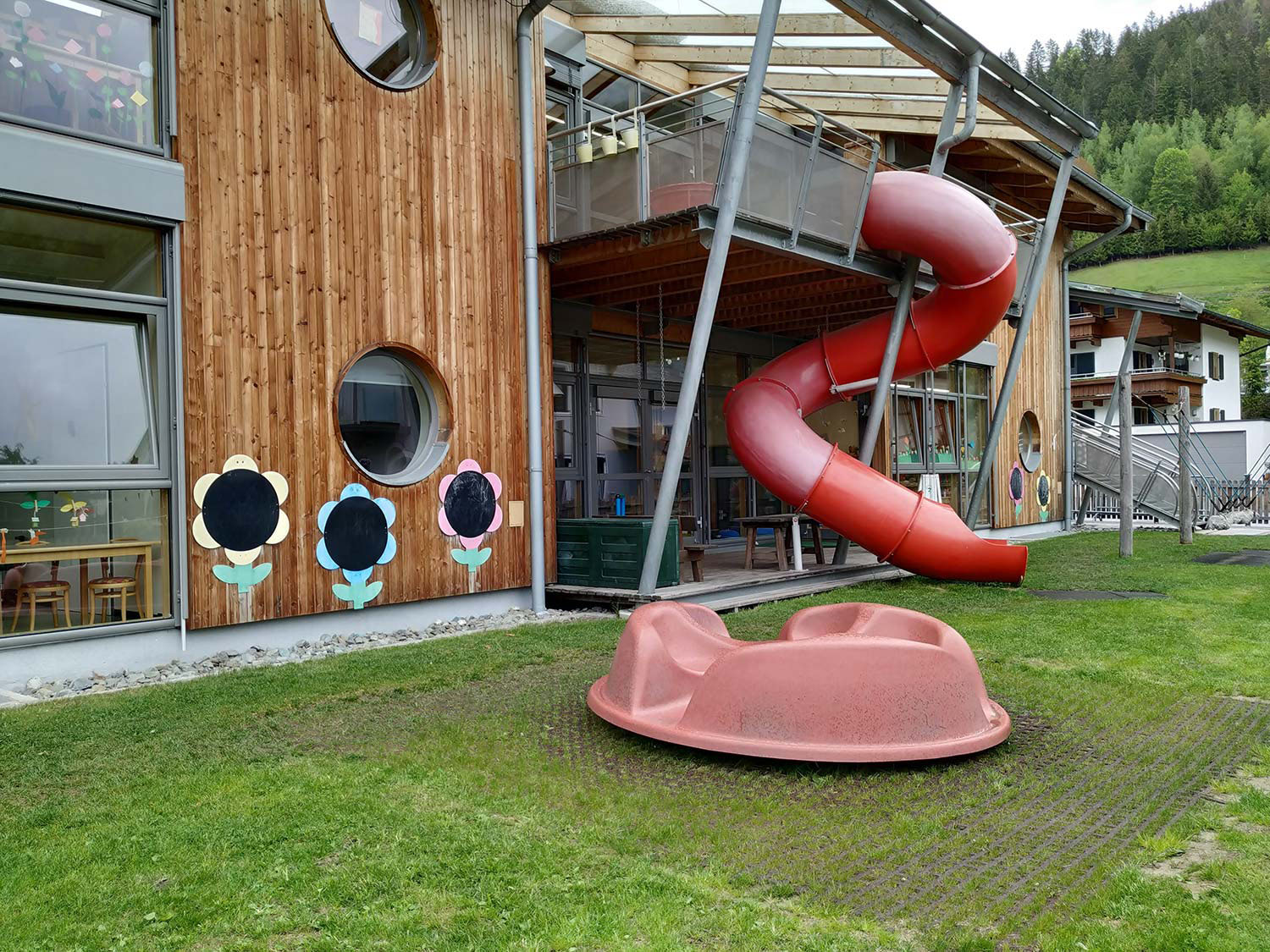

Supplement: Supplementary Data Sheet S5 — Photographic record of building tour. [file Data_Sheet_5.zip › activity areas/garden_3.jpg]

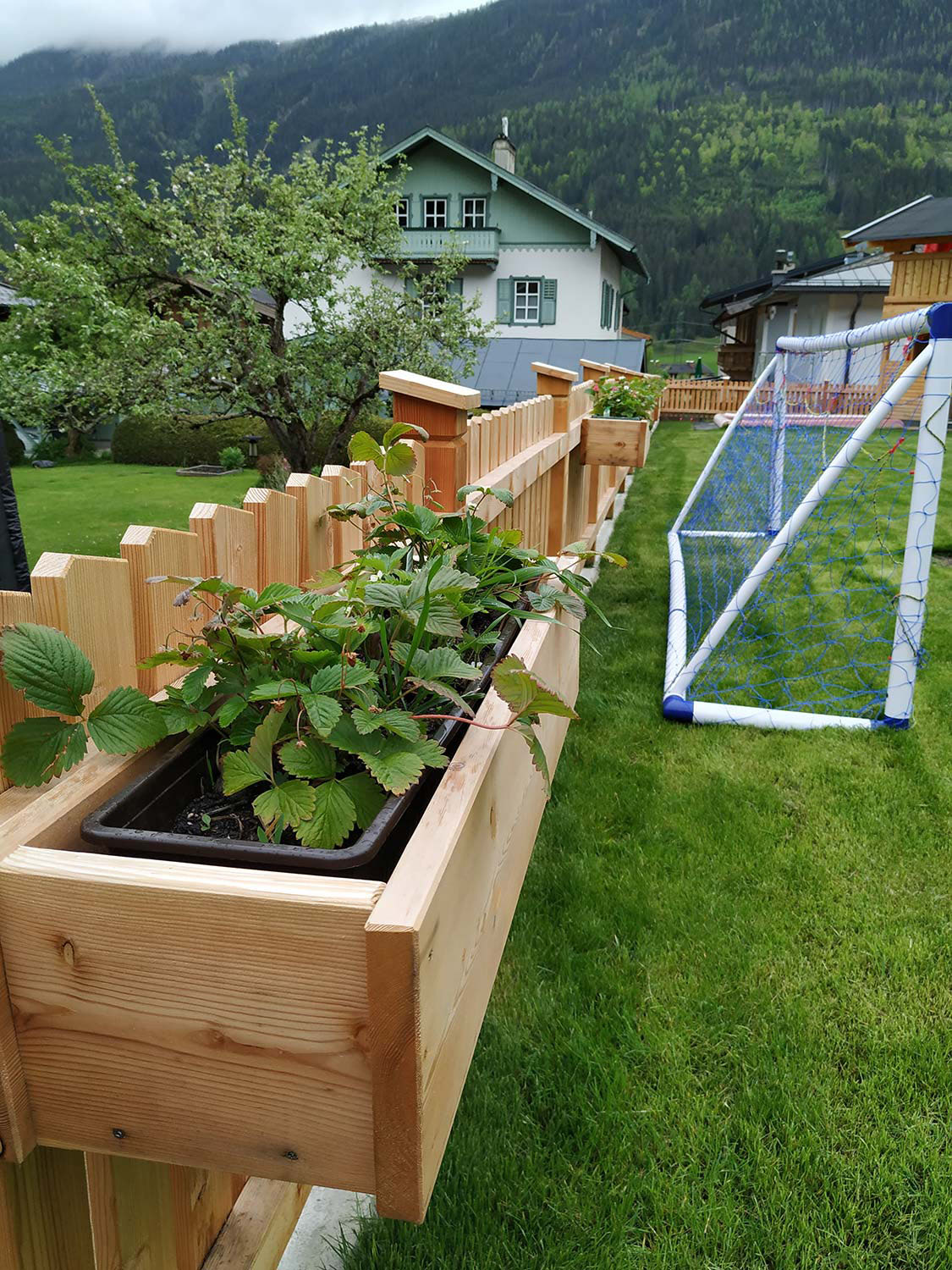

Supplement: Supplementary Data Sheet S5 — Photographic record of building tour. [file Data_Sheet_5.zip › activity areas/garden_4.jpg]

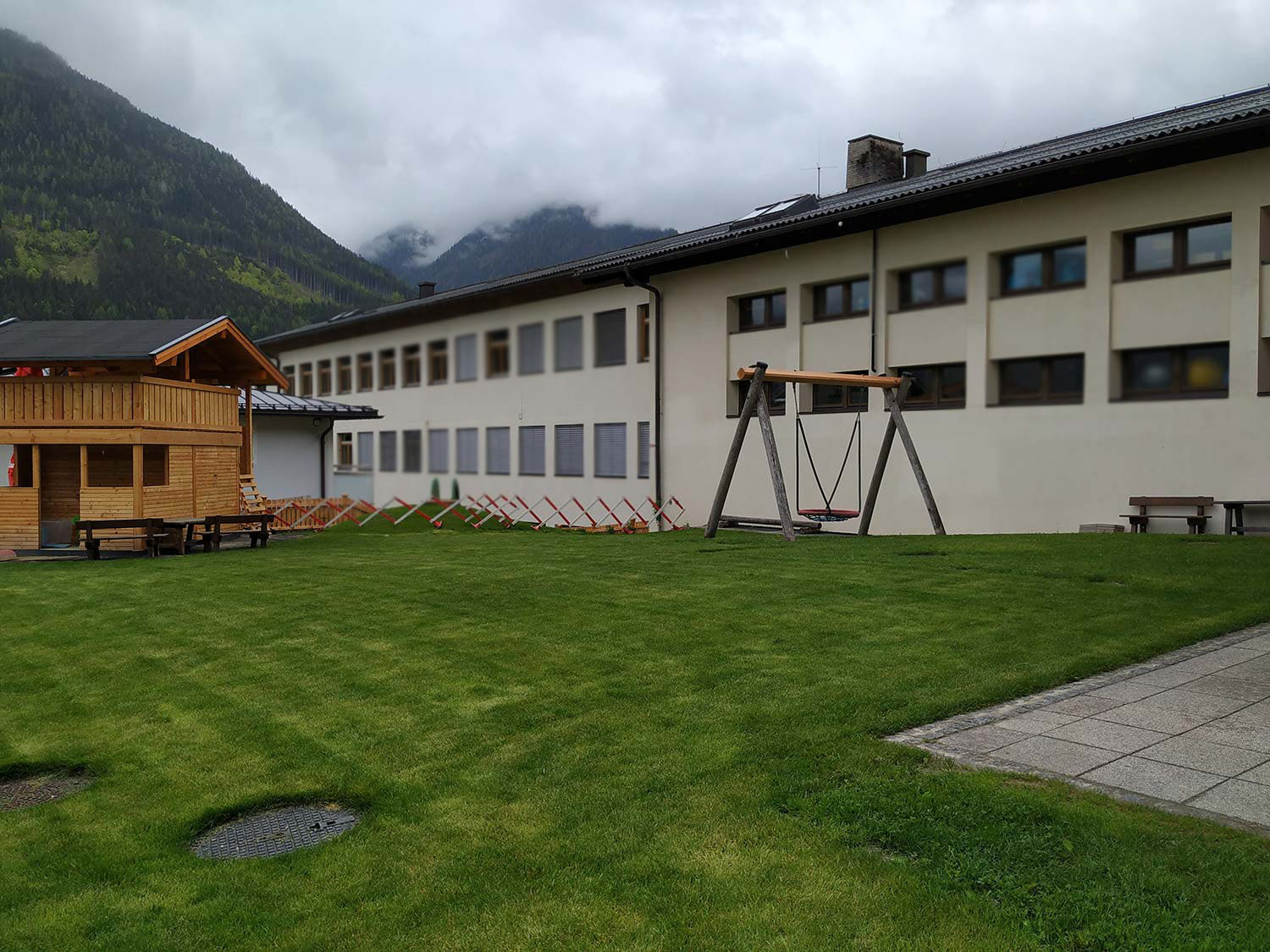

Supplement: Supplementary Data Sheet S5 — Photographic record of building tour. [file Data_Sheet_5.zip › activity areas/garden_5.jpg]

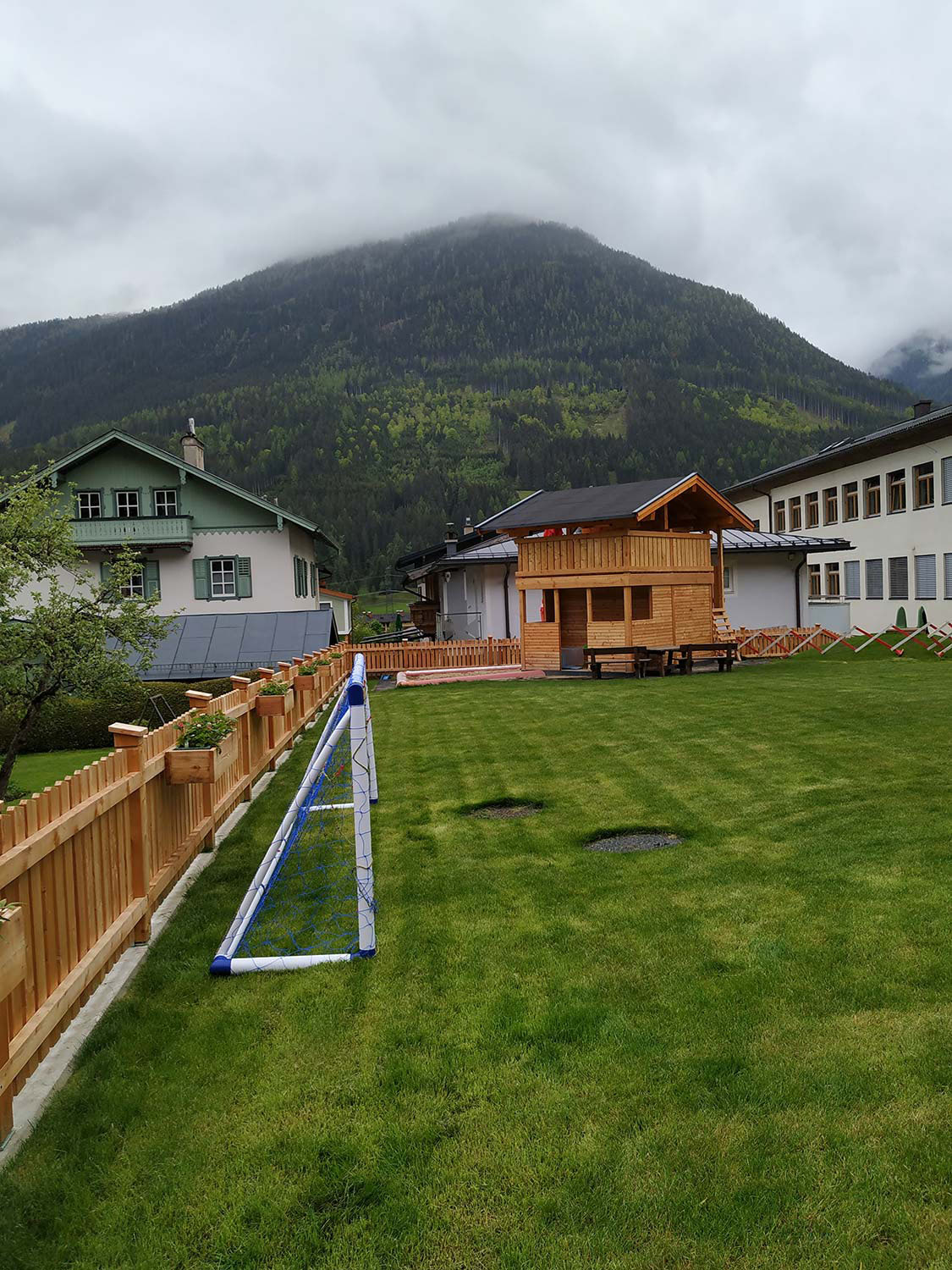

Supplement: Supplementary Data Sheet S5 — Photographic record of building tour. [file Data_Sheet_5.zip › activity areas/garden_6.jpg]

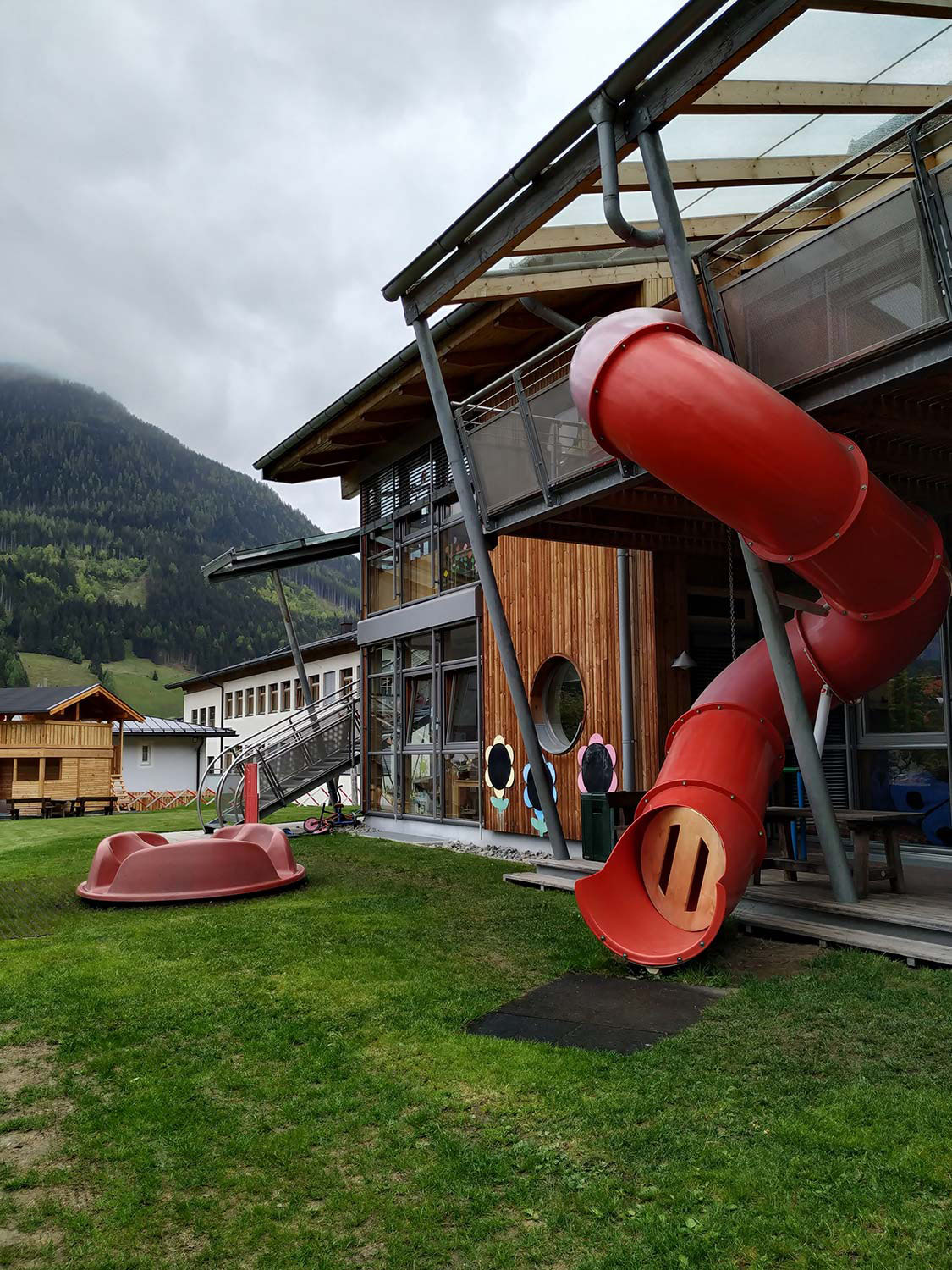

Supplement: Supplementary Data Sheet S5 — Photographic record of building tour. [file Data_Sheet_5.zip › activity areas/garden_7.jpg]

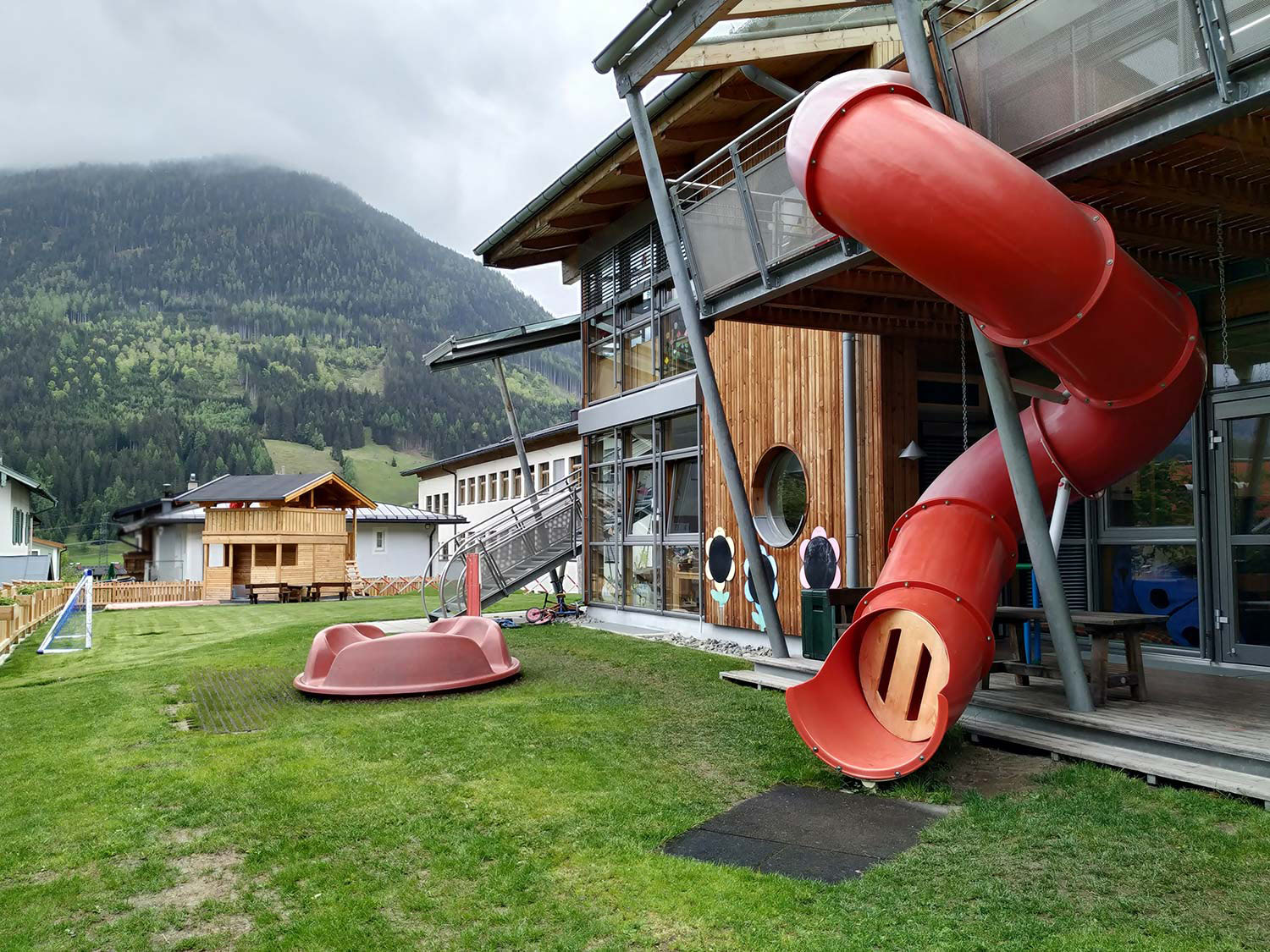

Supplement: Supplementary Data Sheet S5 — Photographic record of building tour. [file Data_Sheet_5.zip › activity areas/garden_8.jpg]

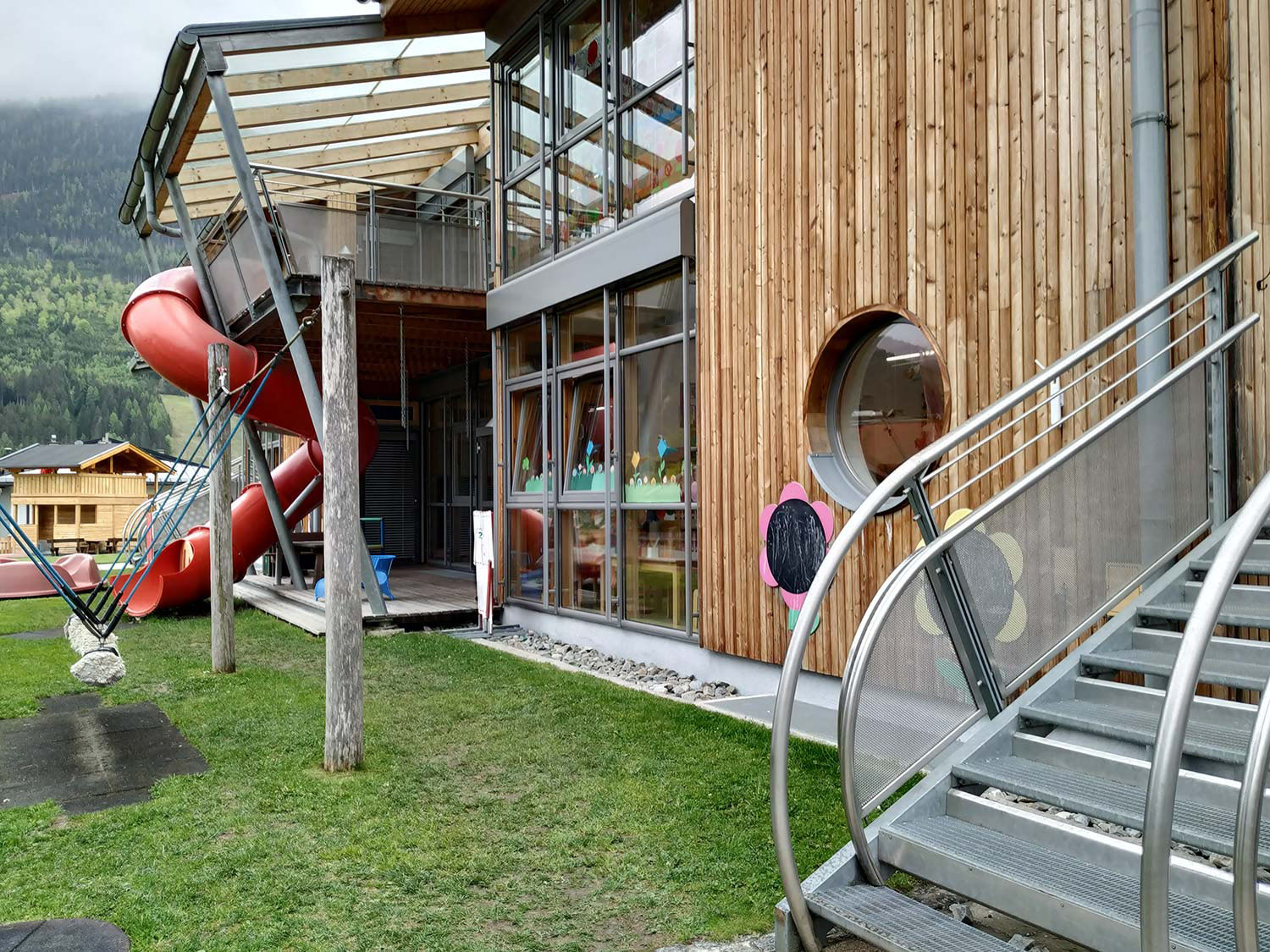

Supplement: Supplementary Data Sheet S5 — Photographic record of building tour. [file Data_Sheet_5.zip › activity areas/garden_9.jpg]

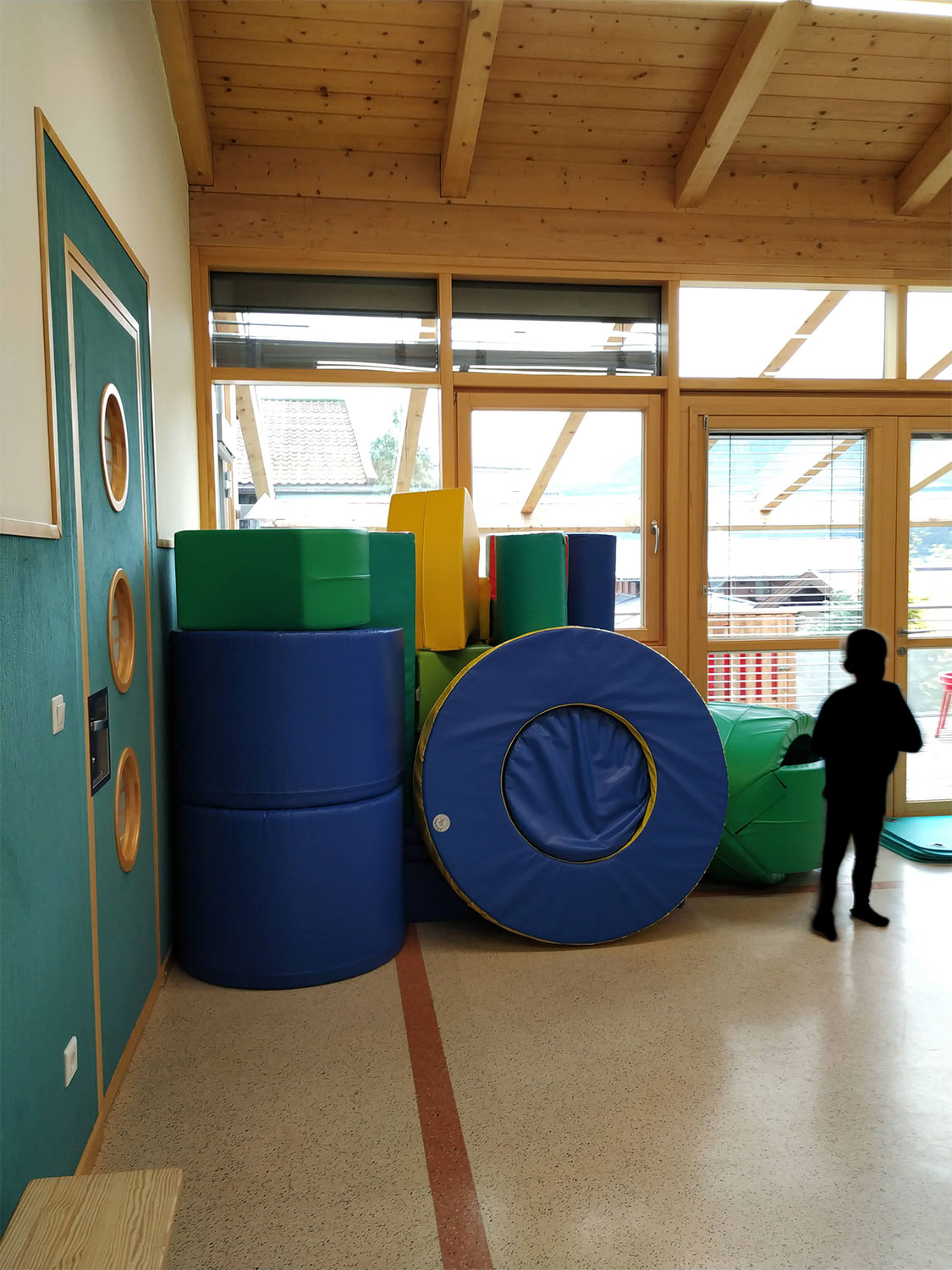

Supplement: Supplementary Data Sheet S5 — Photographic record of building tour. [file Data_Sheet_5.zip › activity areas/gym_1.jpg]

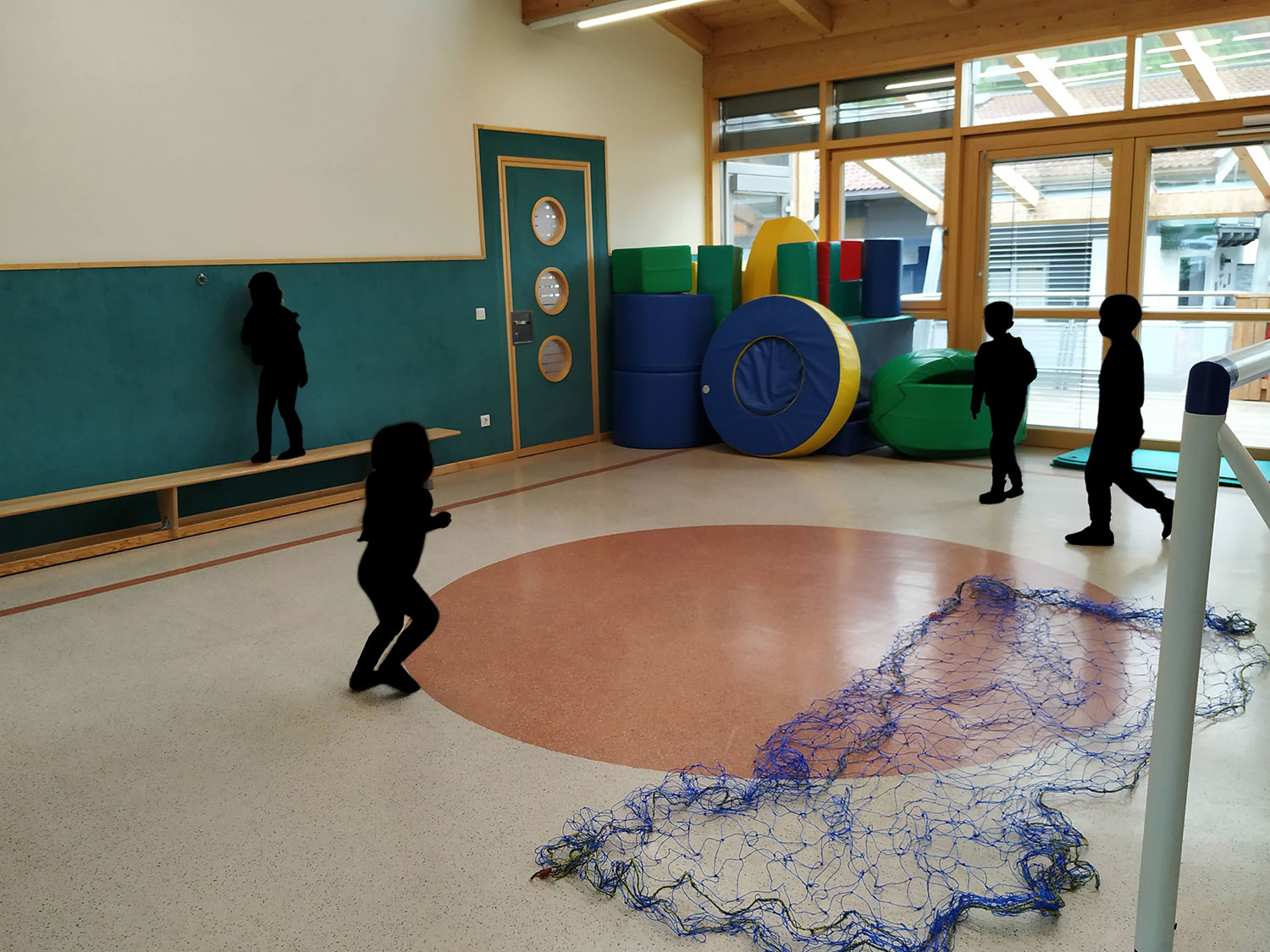

Supplement: Supplementary Data Sheet S5 — Photographic record of building tour. [file Data_Sheet_5.zip › activity areas/gym_2.jpg]

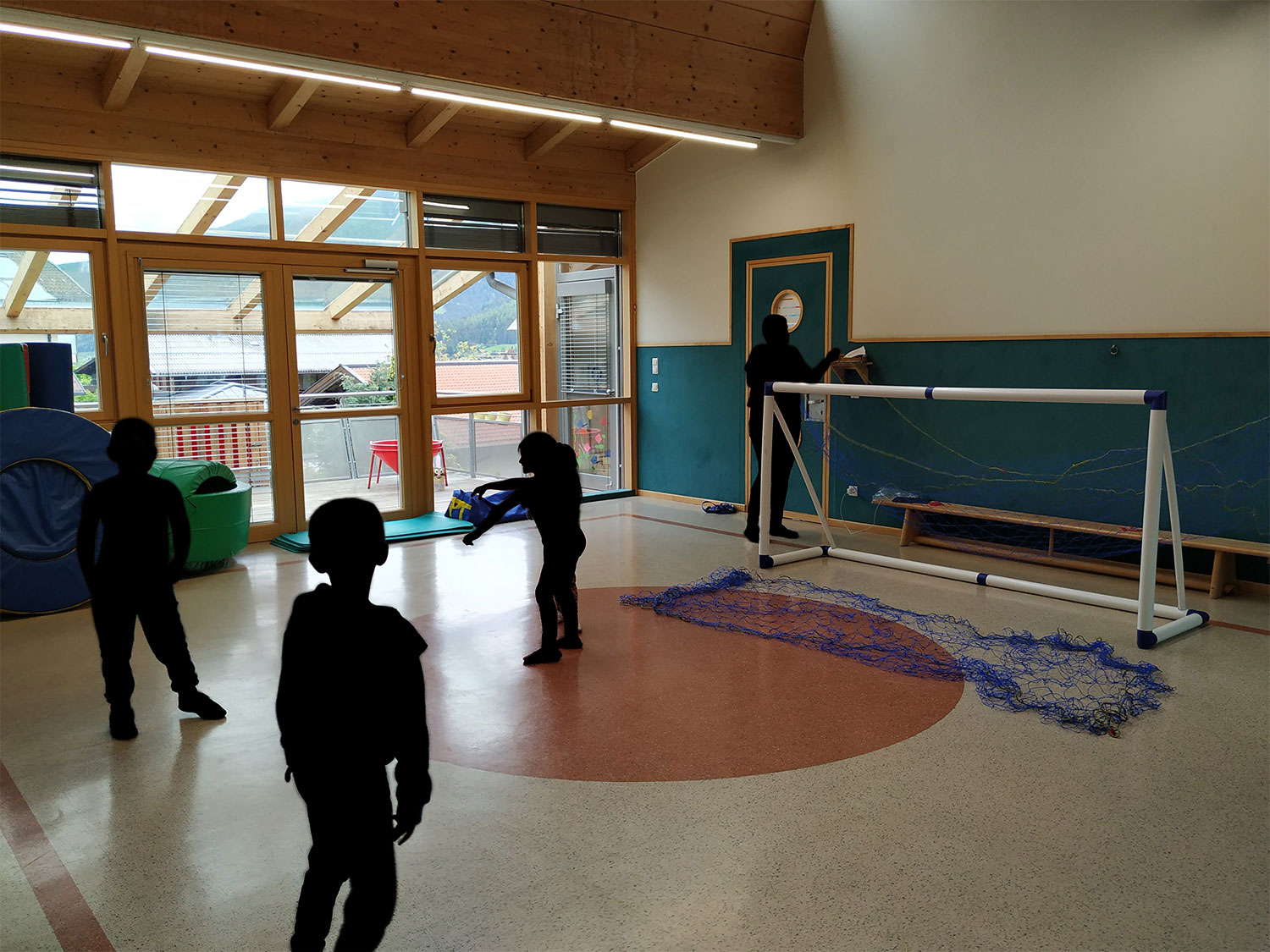

Supplement: Supplementary Data Sheet S5 — Photographic record of building tour. [file Data_Sheet_5.zip › activity areas/gym_3.jpg]

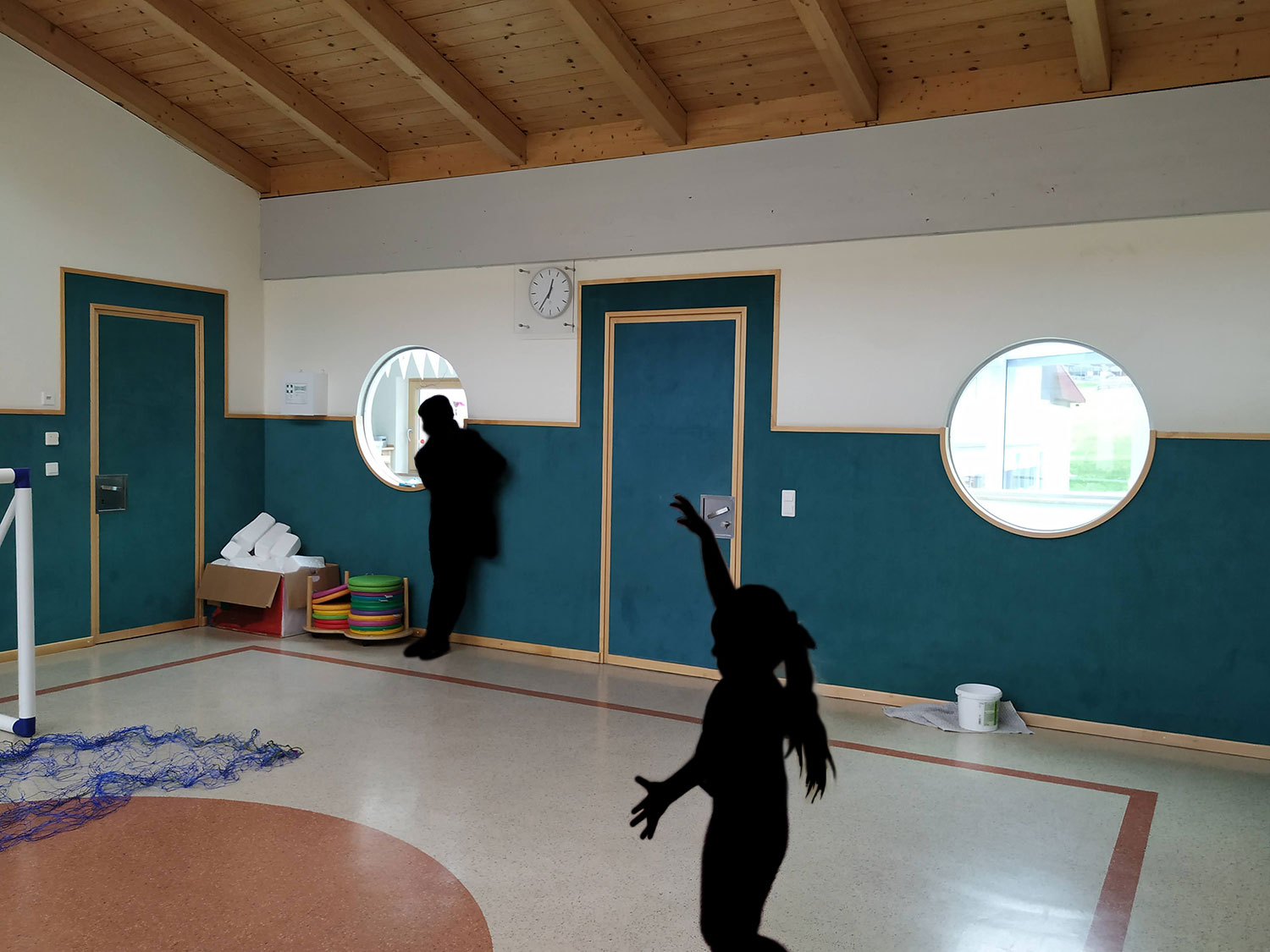

Supplement: Supplementary Data Sheet S5 — Photographic record of building tour. [file Data_Sheet_5.zip › activity areas/gym_4.jpg]

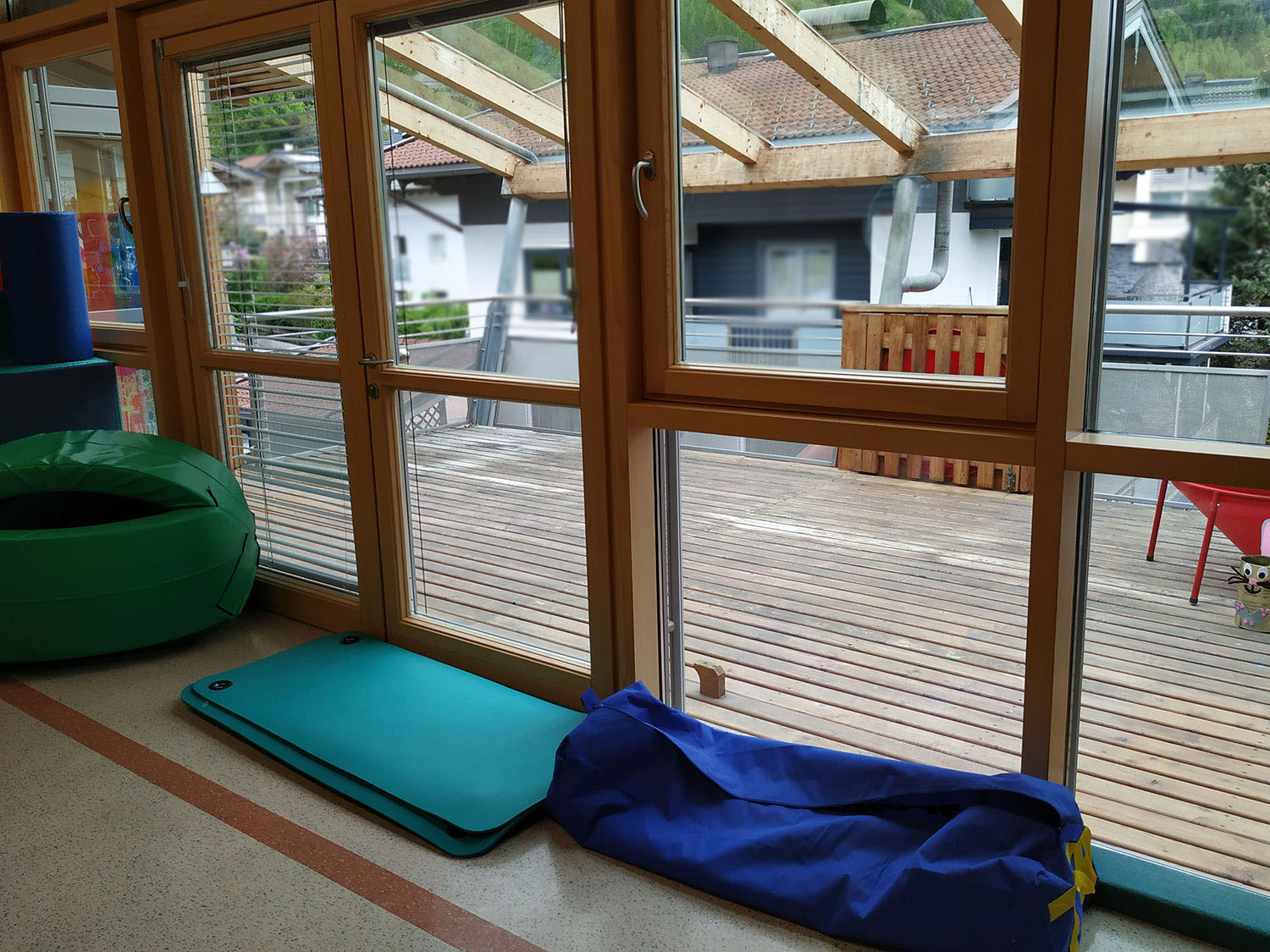

Supplement: Supplementary Data Sheet S5 — Photographic record of building tour. [file Data_Sheet_5.zip › activity areas/gym_5.jpg]

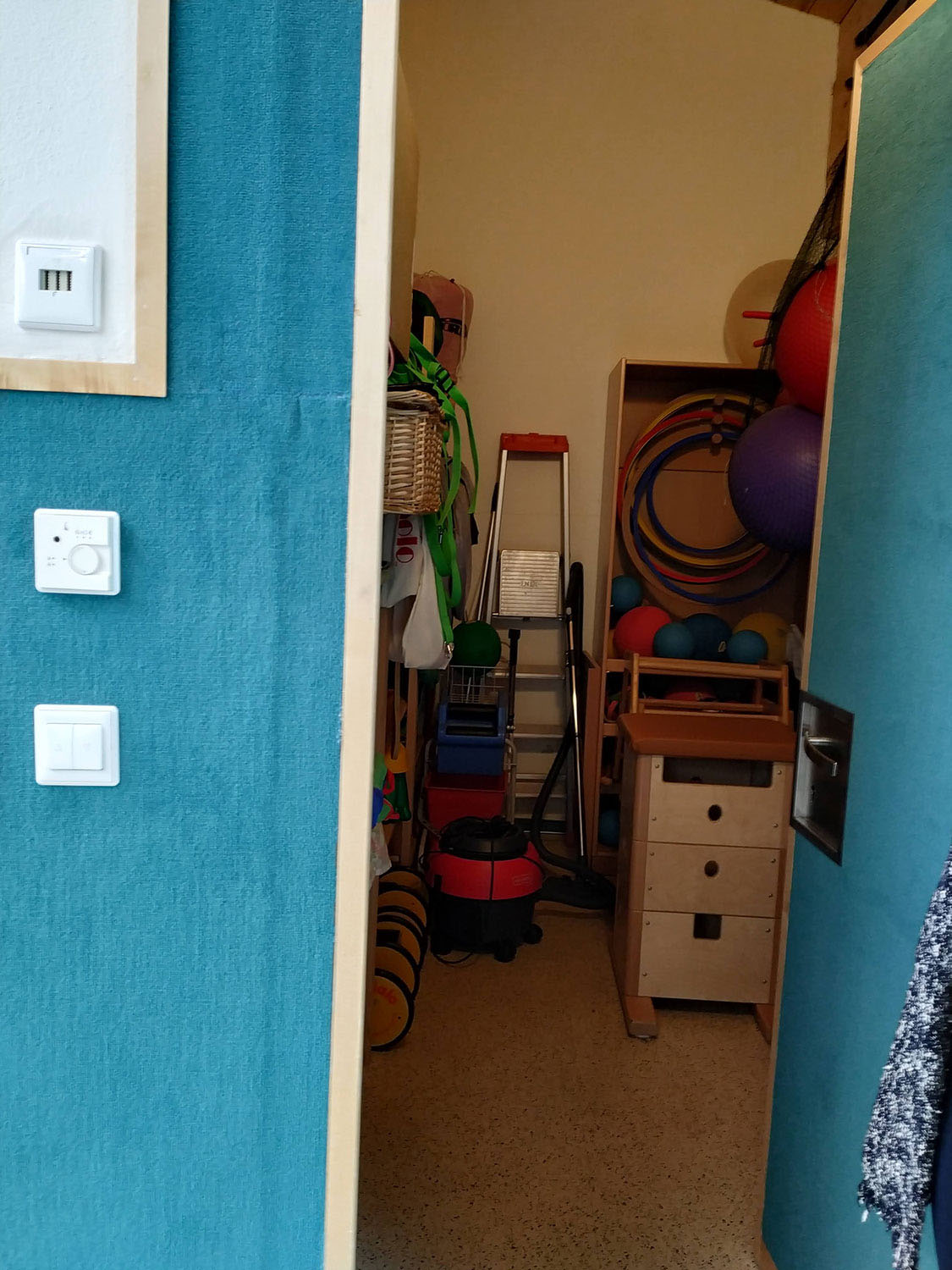

Supplement: Supplementary Data Sheet S5 — Photographic record of building tour. [file Data_Sheet_5.zip › activity areas/gym_6.jpg]

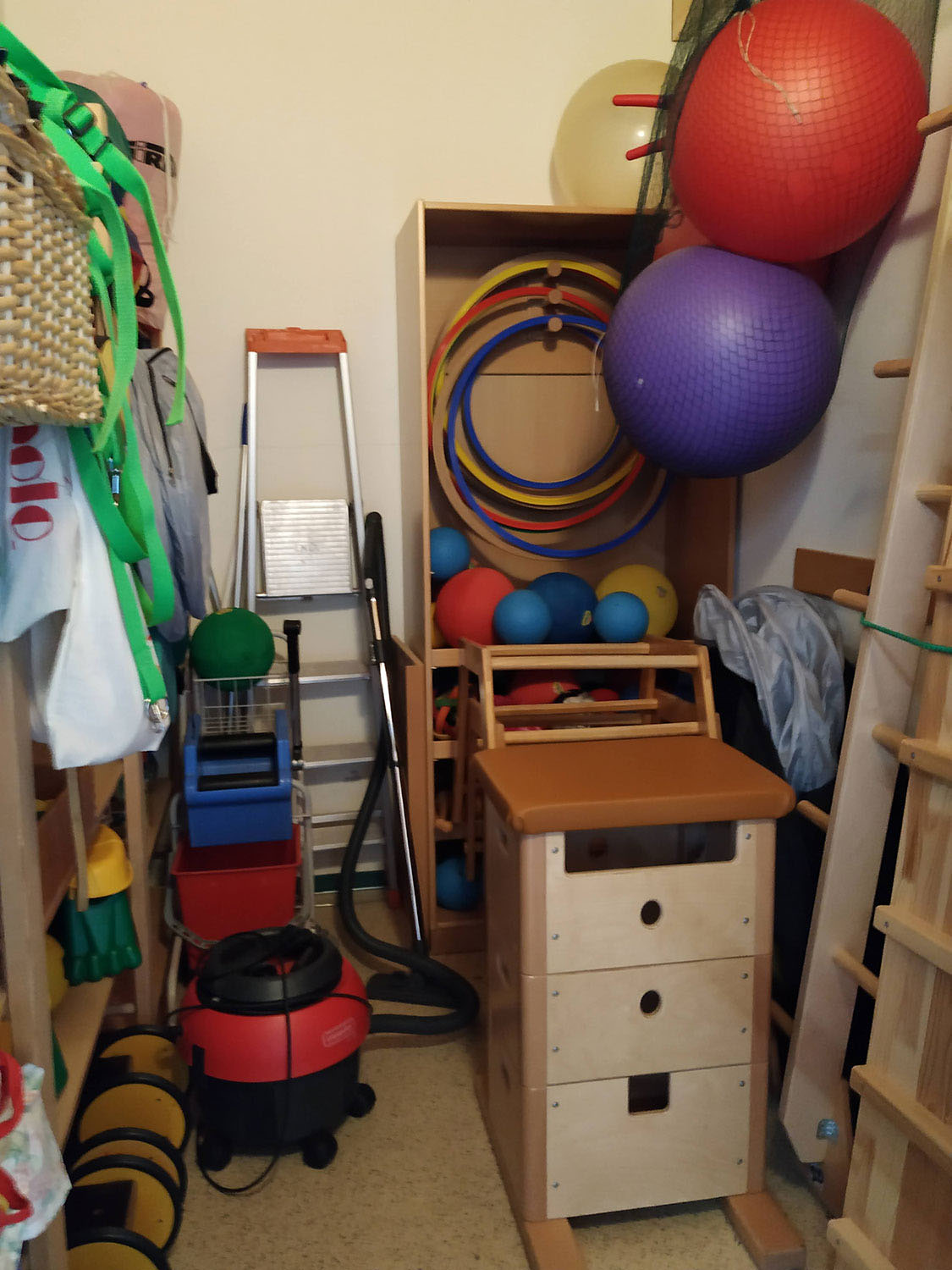

Supplement: Supplementary Data Sheet S5 — Photographic record of building tour. [file Data_Sheet_5.zip › activity areas/gym_7.jpg]

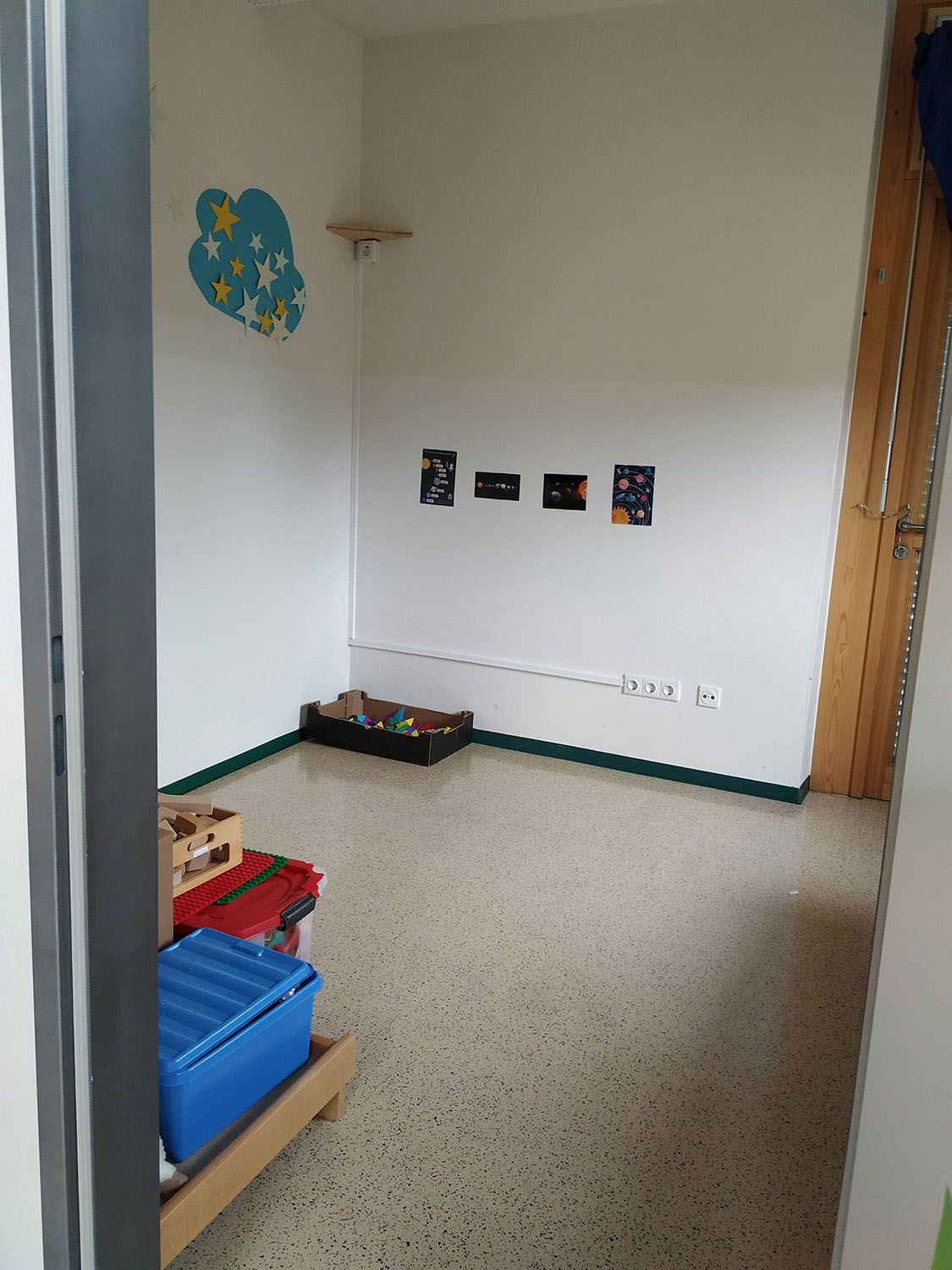

Supplement: Supplementary Data Sheet S5 — Photographic record of building tour. [file Data_Sheet_5.zip › activity areas/resting-area_1.jpg]

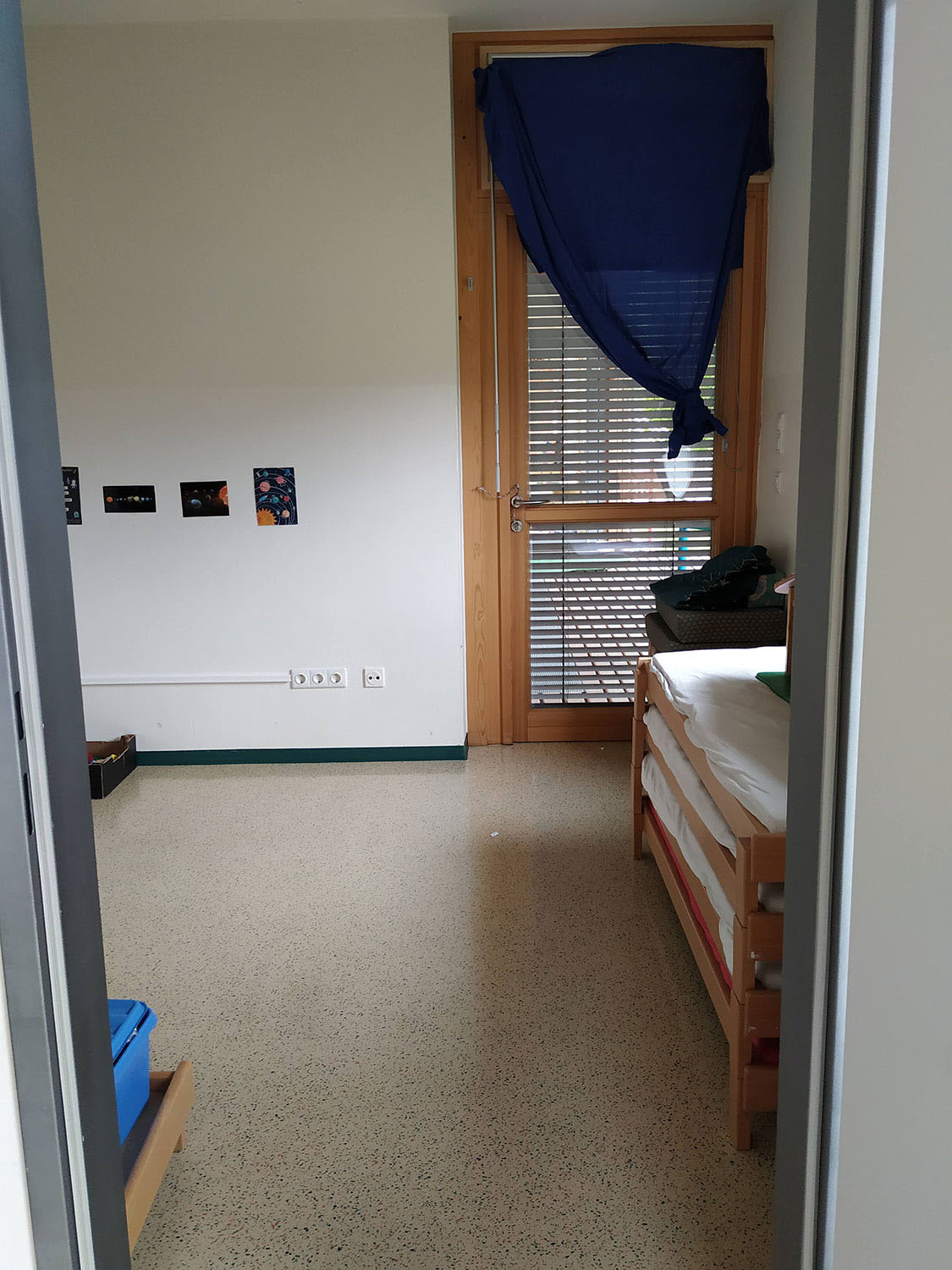

Supplement: Supplementary Data Sheet S5 — Photographic record of building tour. [file Data_Sheet_5.zip › activity areas/resting-area_2.jpg]

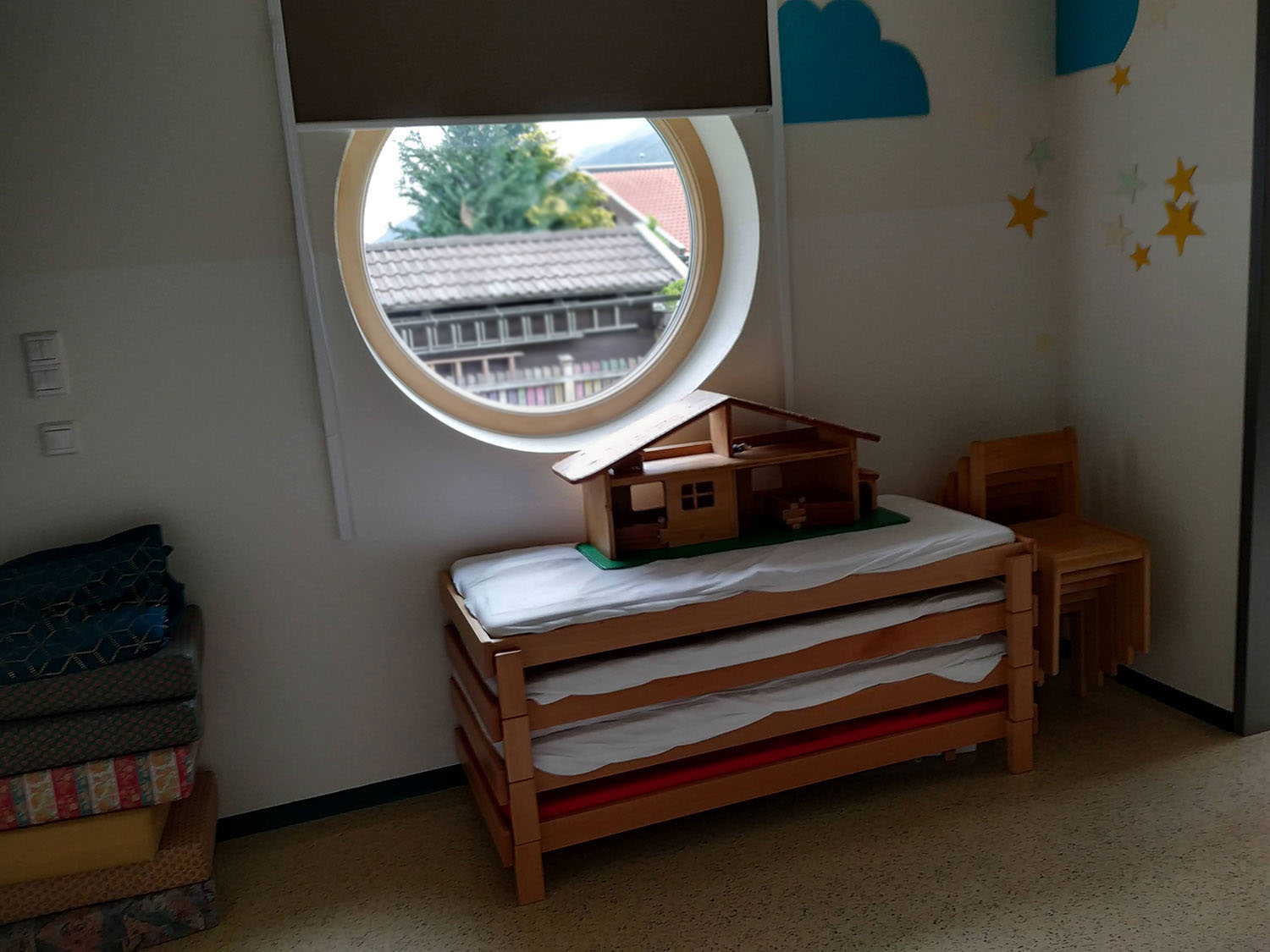

Supplement: Supplementary Data Sheet S5 — Photographic record of building tour. [file Data_Sheet_5.zip › activity areas/resting-area_3.jpg]

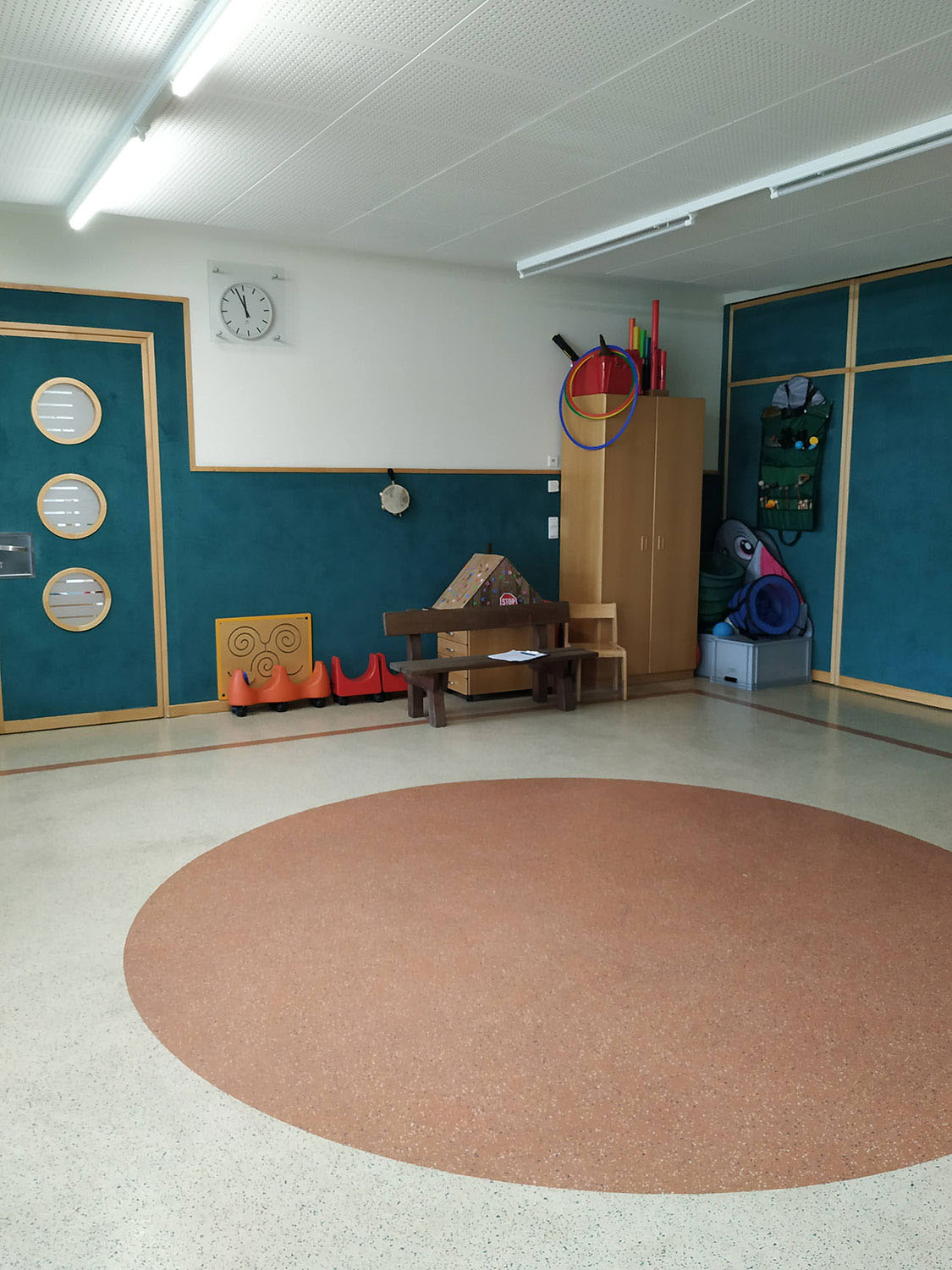

Supplement: Supplementary Data Sheet S5 — Photographic record of building tour. [file Data_Sheet_5.zip › activity areas/rhythmics-area_1.jpg]

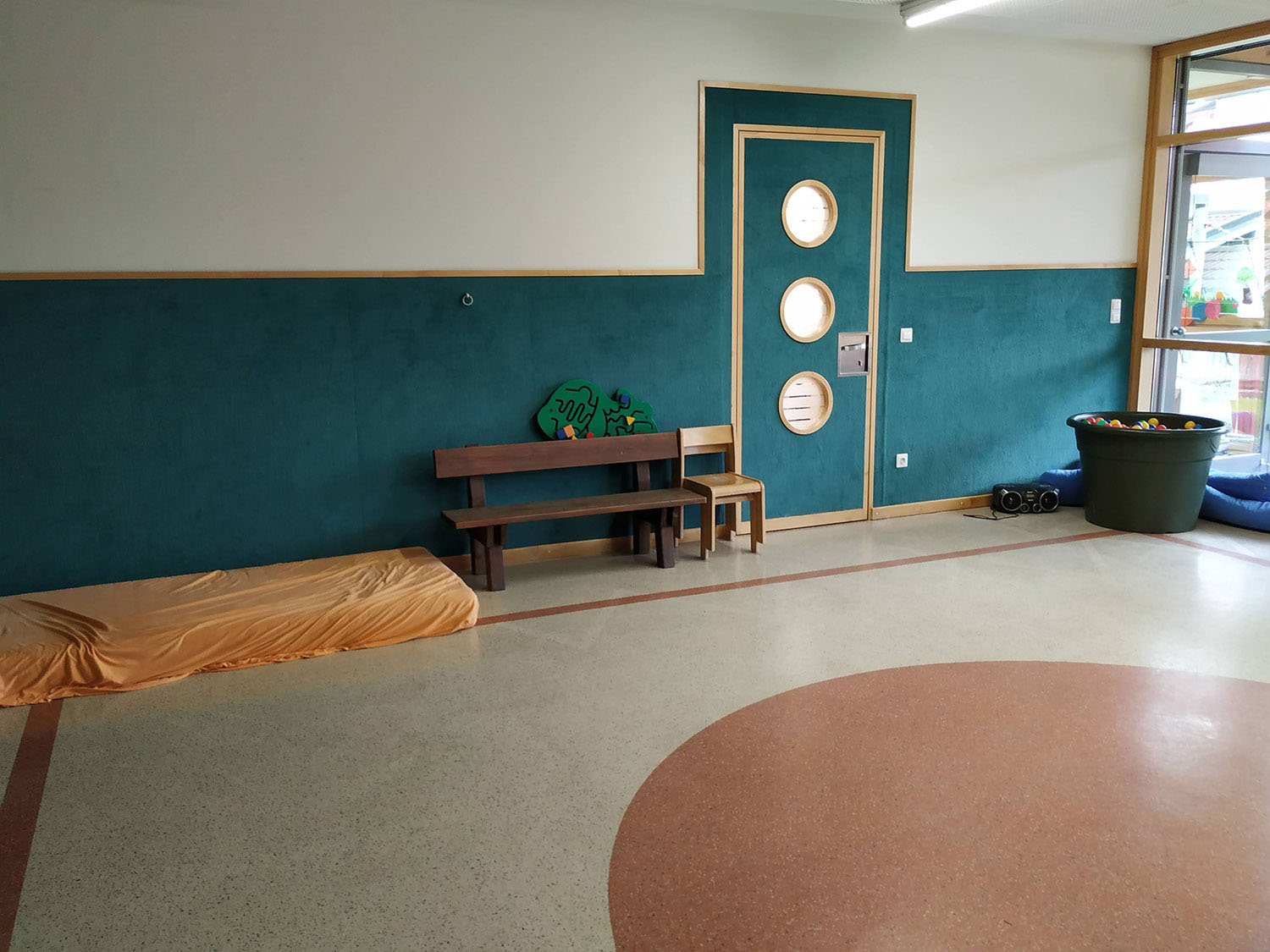

Supplement: Supplementary Data Sheet S5 — Photographic record of building tour. [file Data_Sheet_5.zip › activity areas/rhythmics-area_2.jpg]

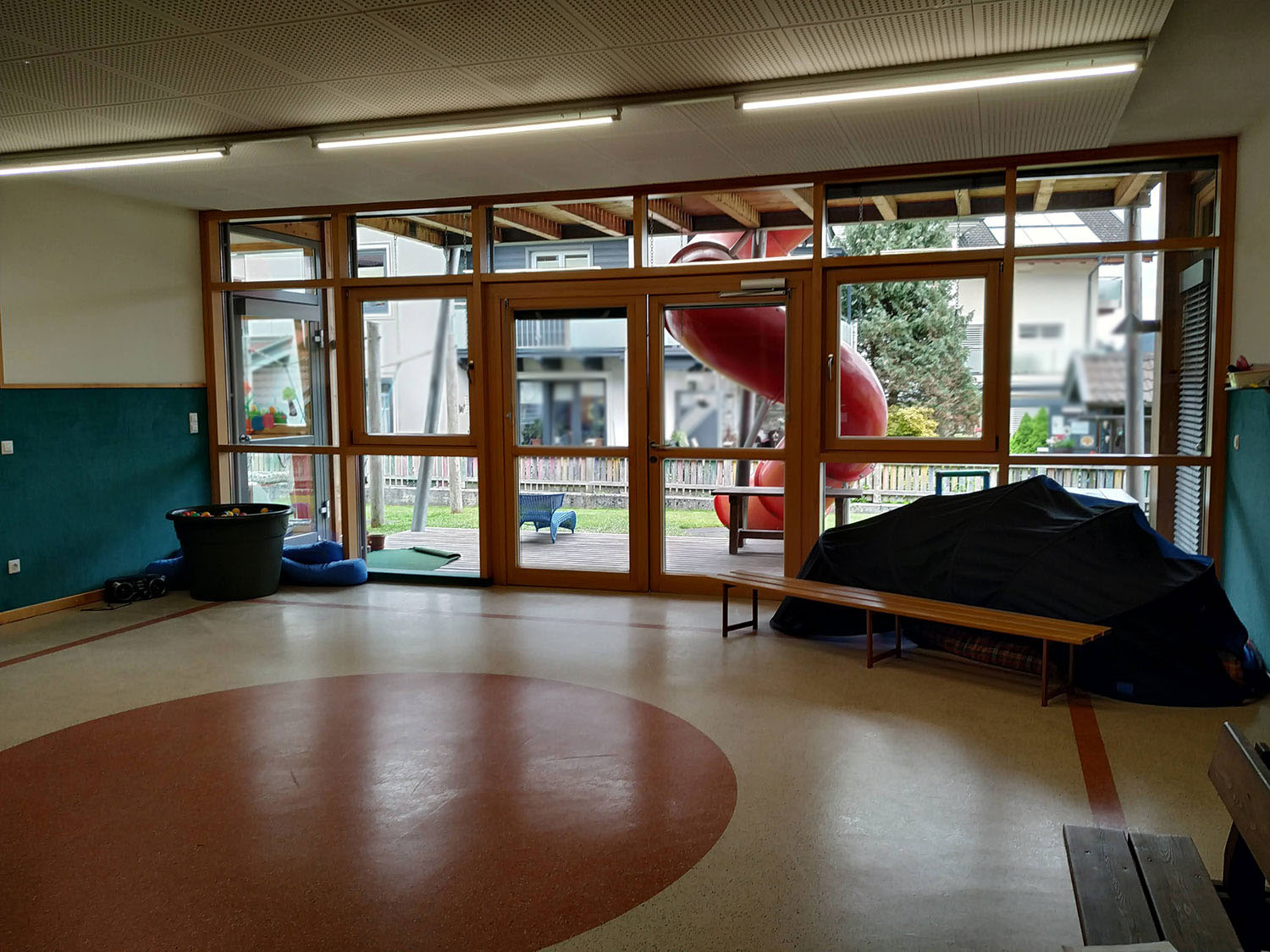

Supplement: Supplementary Data Sheet S5 — Photographic record of building tour. [file Data_Sheet_5.zip › activity areas/rhythmics-area_3.jpg]

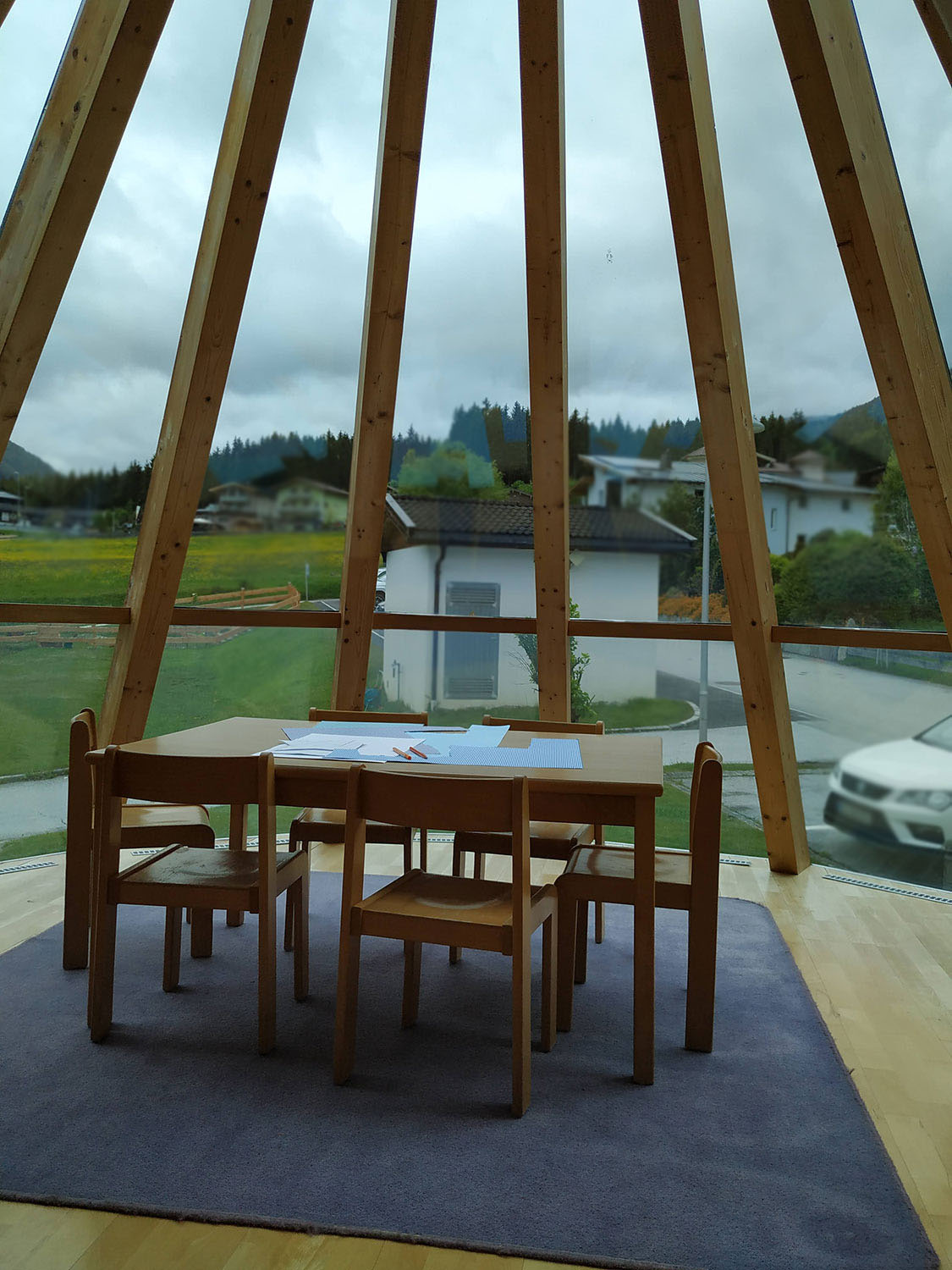

Supplement: Supplementary Data Sheet S5 — Photographic record of building tour. [file Data_Sheet_5.zip › common areas/common-area_1.jpg]

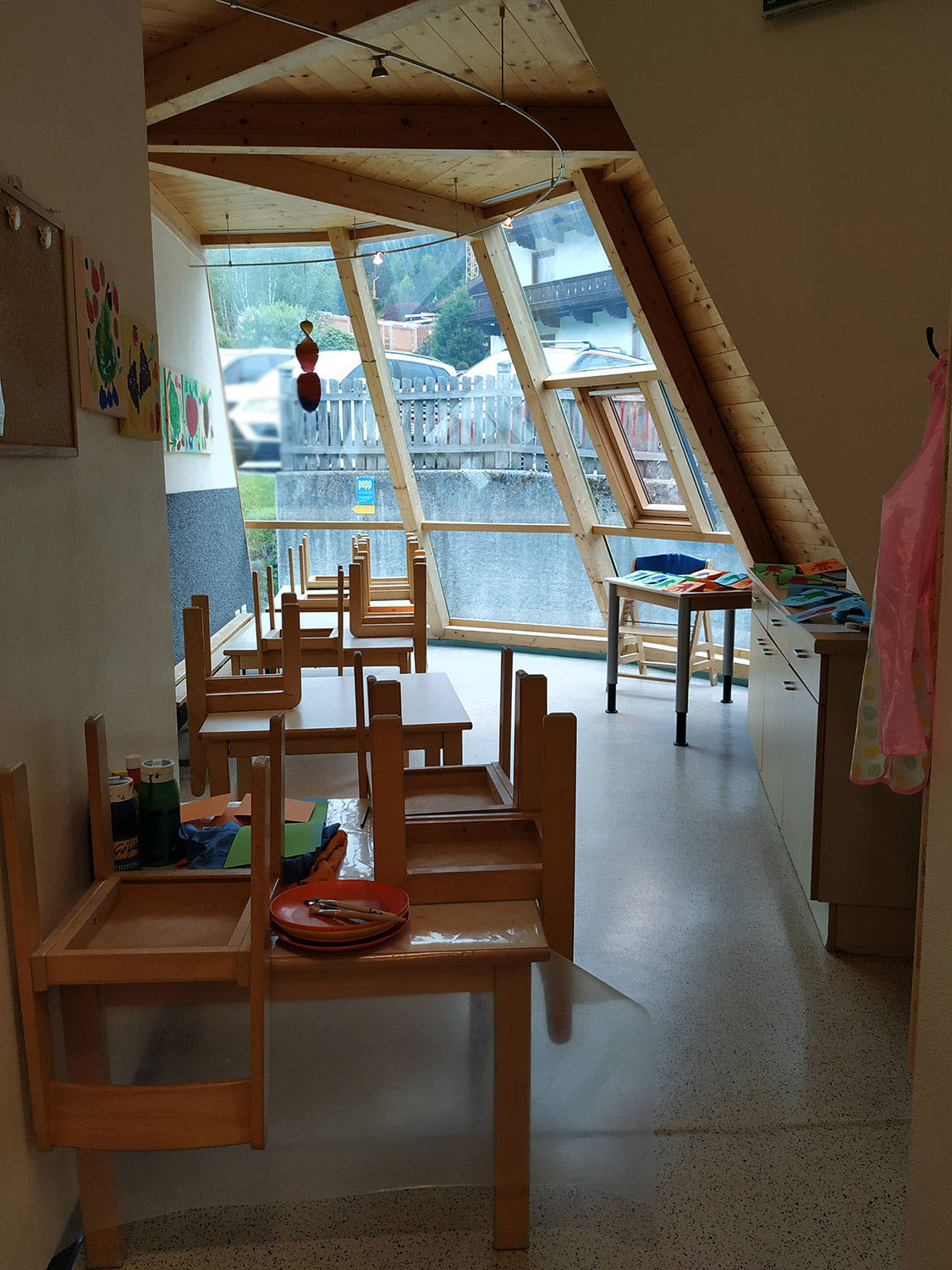

Supplement: Supplementary Data Sheet S5 — Photographic record of building tour. [file Data_Sheet_5.zip › common areas/eating-area_1.jpg]

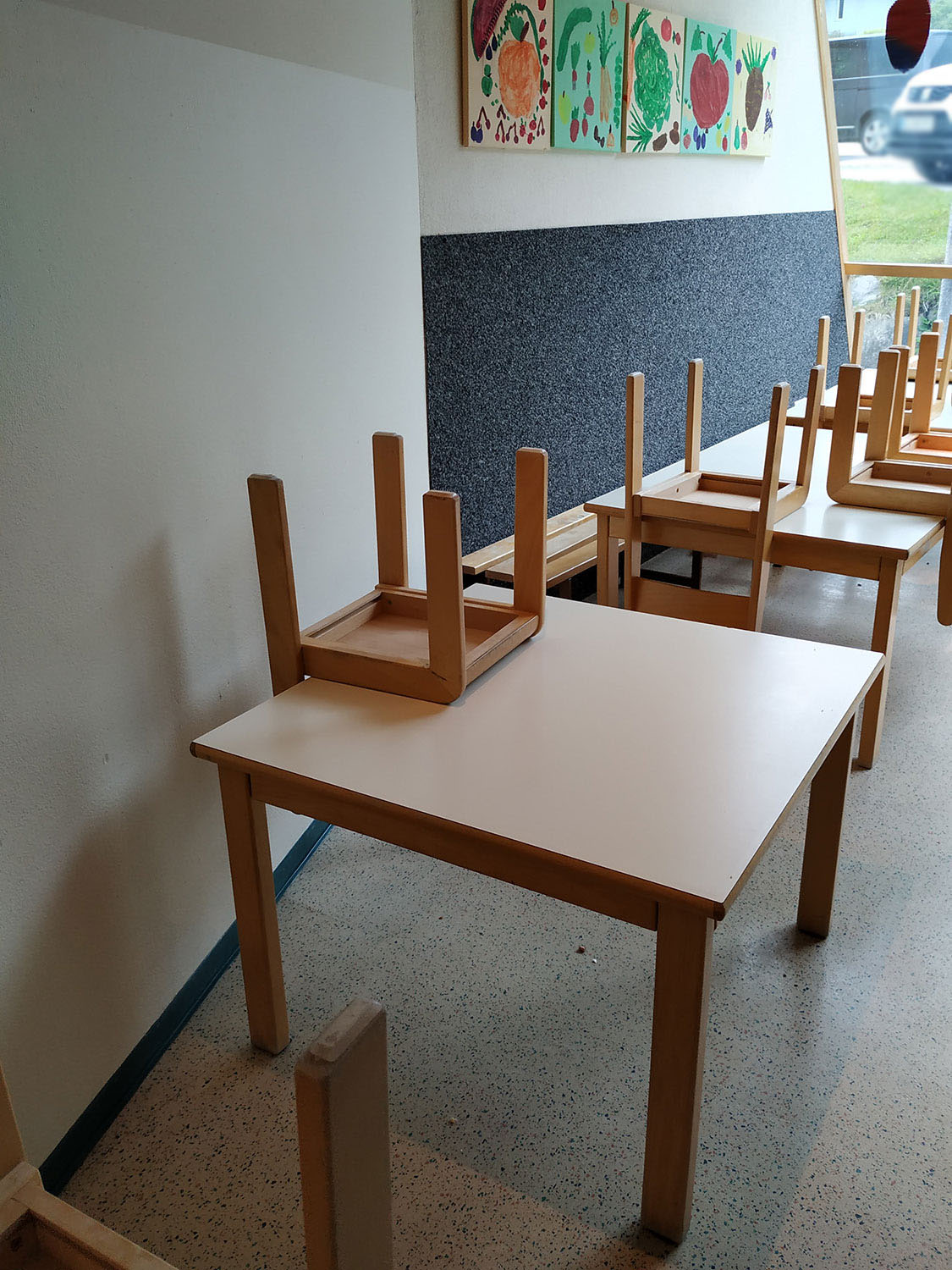

Supplement: Supplementary Data Sheet S5 — Photographic record of building tour. [file Data_Sheet_5.zip › common areas/eating-area_2.jpg]

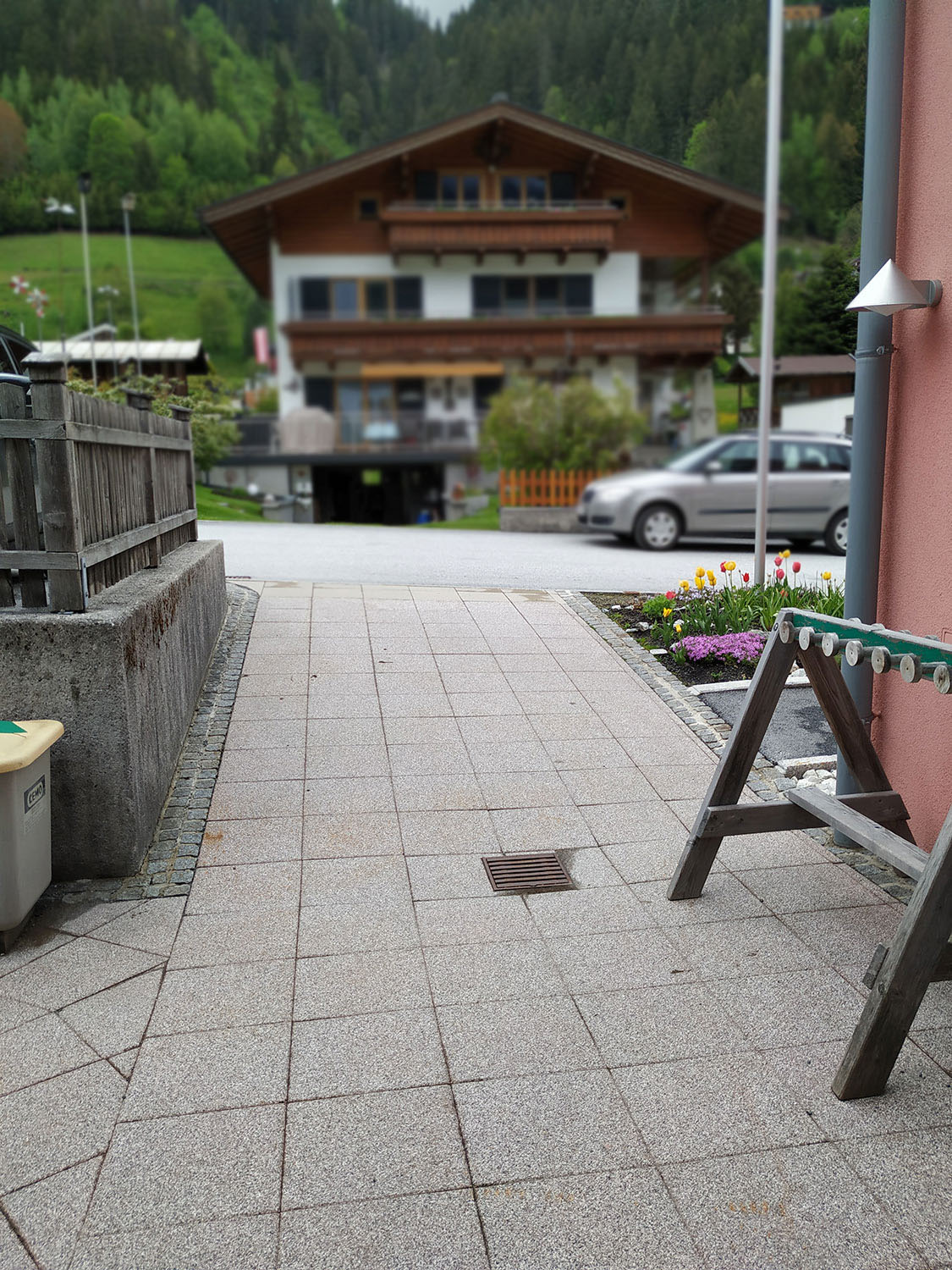

Supplement: Supplementary Data Sheet S5 — Photographic record of building tour. [file Data_Sheet_5.zip › common areas/entrance-area_1.jpg]

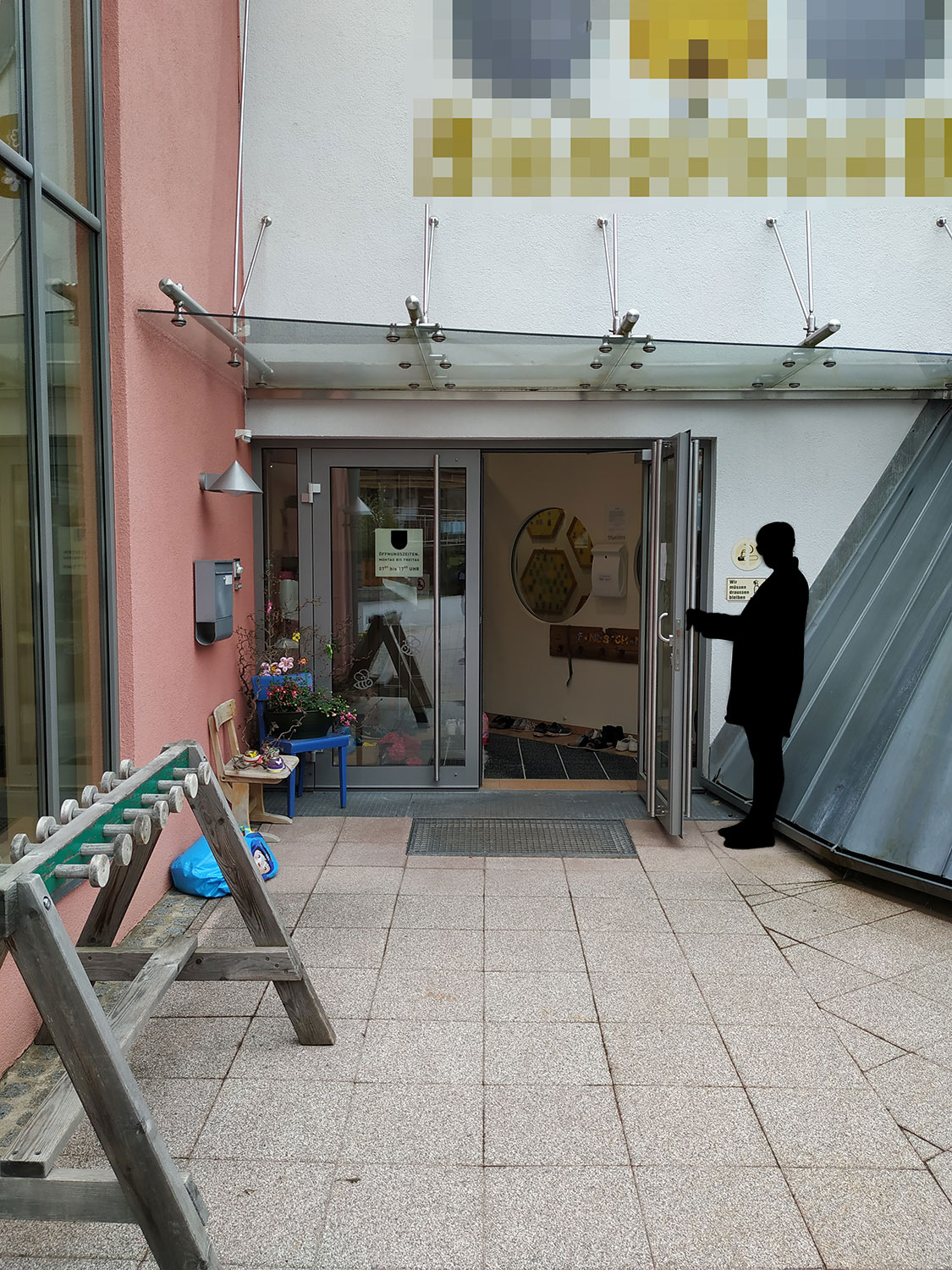

Supplement: Supplementary Data Sheet S5 — Photographic record of building tour. [file Data_Sheet_5.zip › common areas/entrance-area_2.jpg]

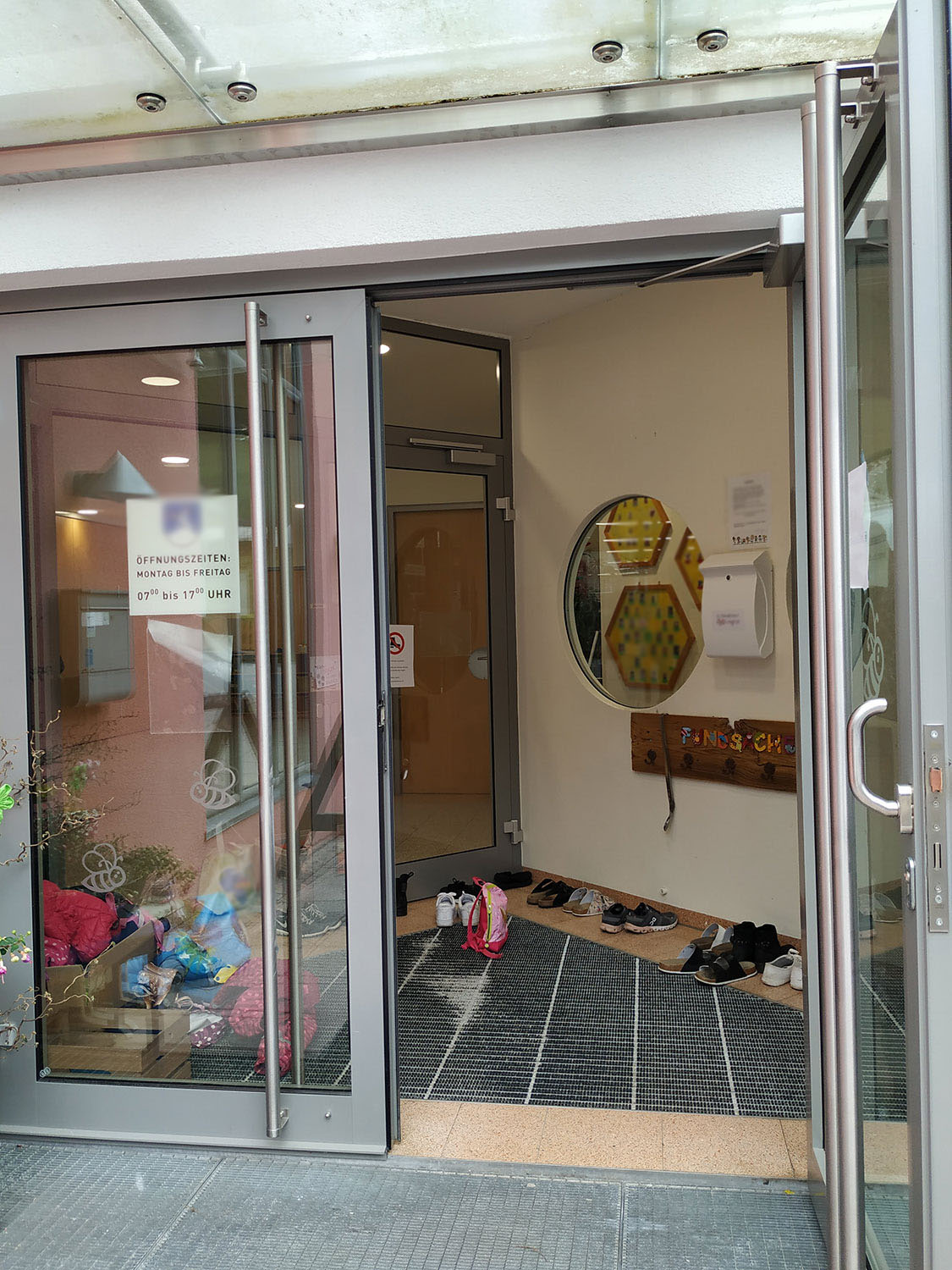

Supplement: Supplementary Data Sheet S5 — Photographic record of building tour. [file Data_Sheet_5.zip › common areas/entrance-area_3.jpg]

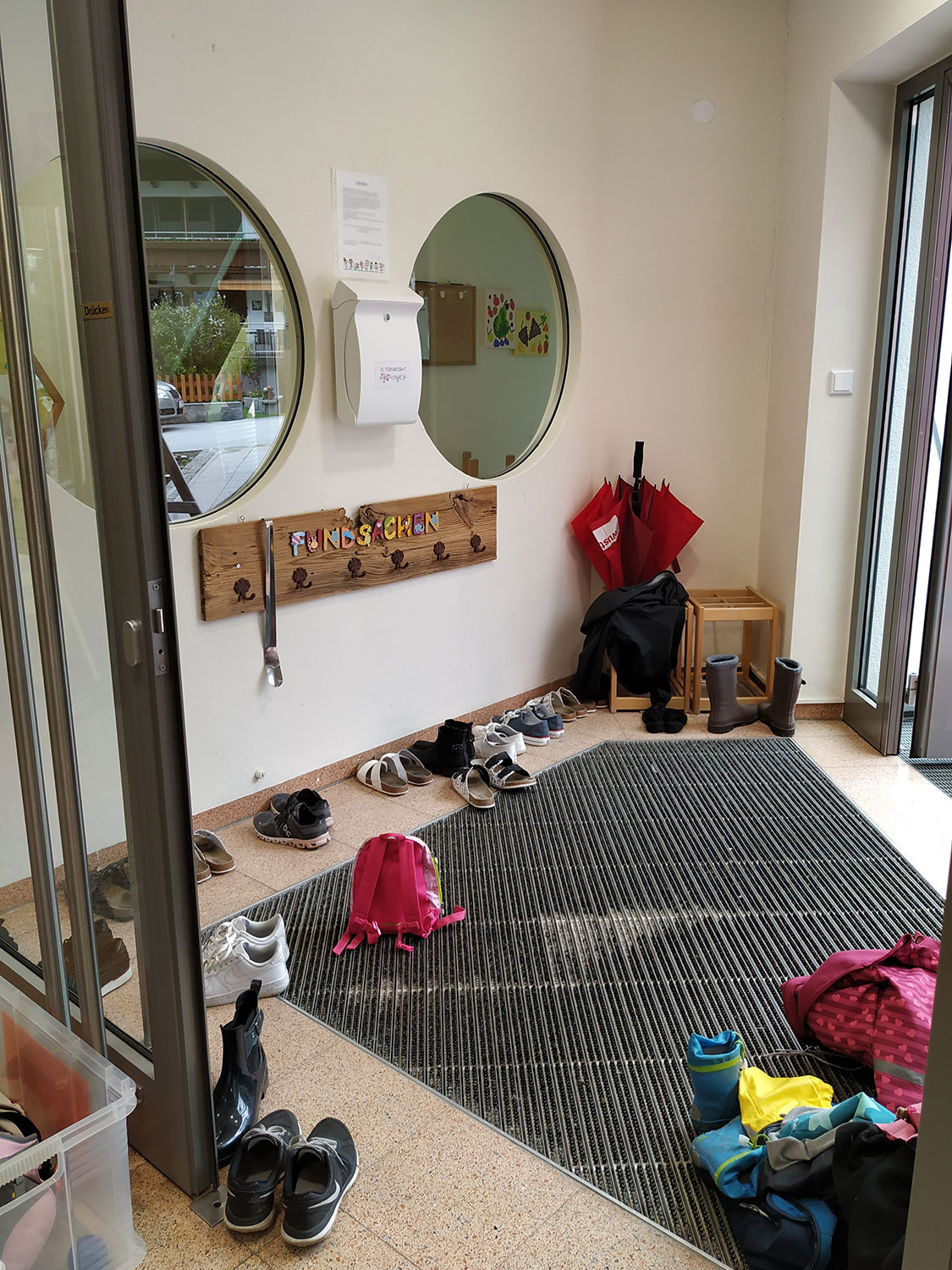

Supplement: Supplementary Data Sheet S5 — Photographic record of building tour. [file Data_Sheet_5.zip › common areas/entrance-area_4.jpg]

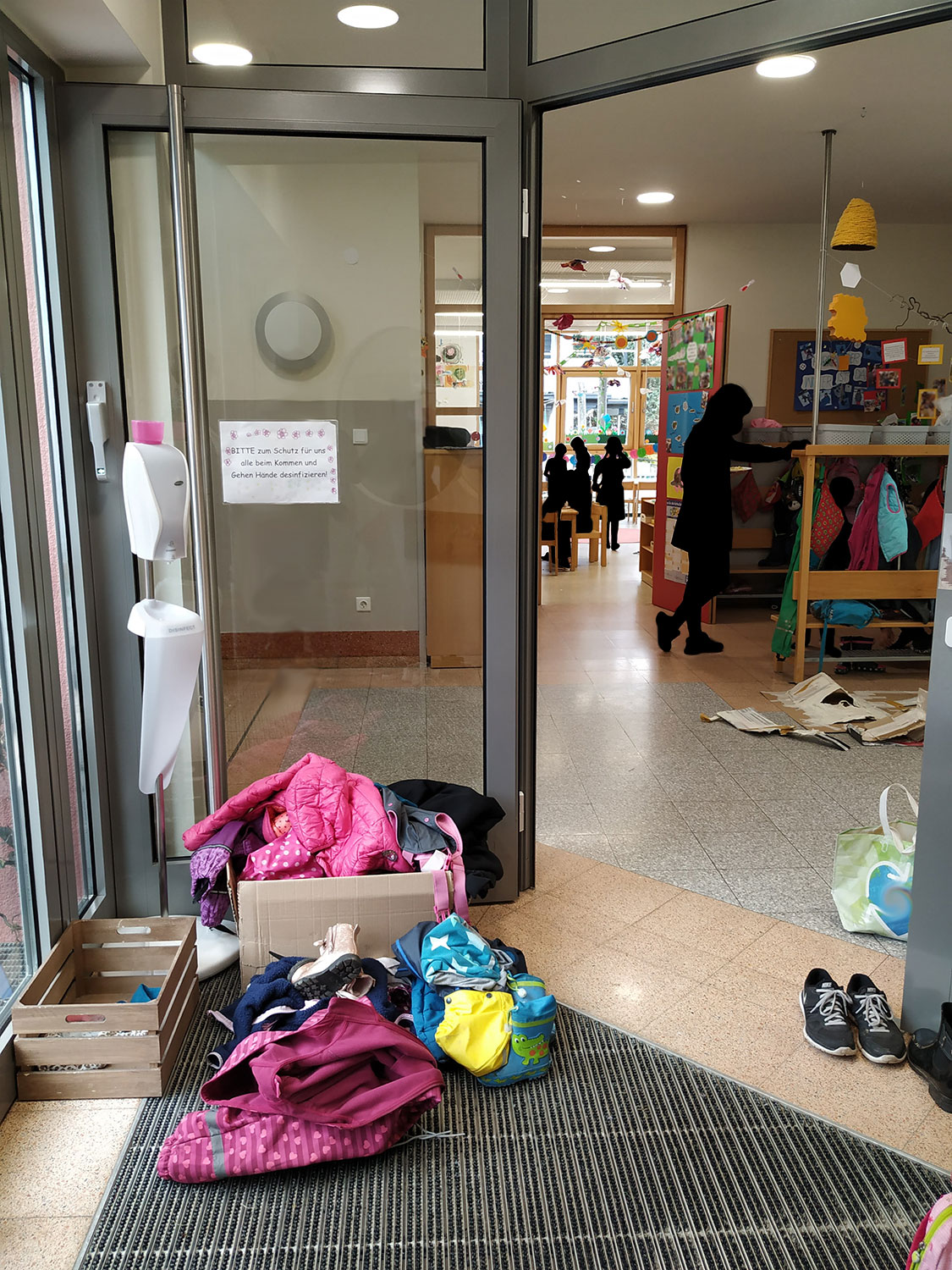

Supplement: Supplementary Data Sheet S5 — Photographic record of building tour. [file Data_Sheet_5.zip › common areas/entrance-area_5.jpg]

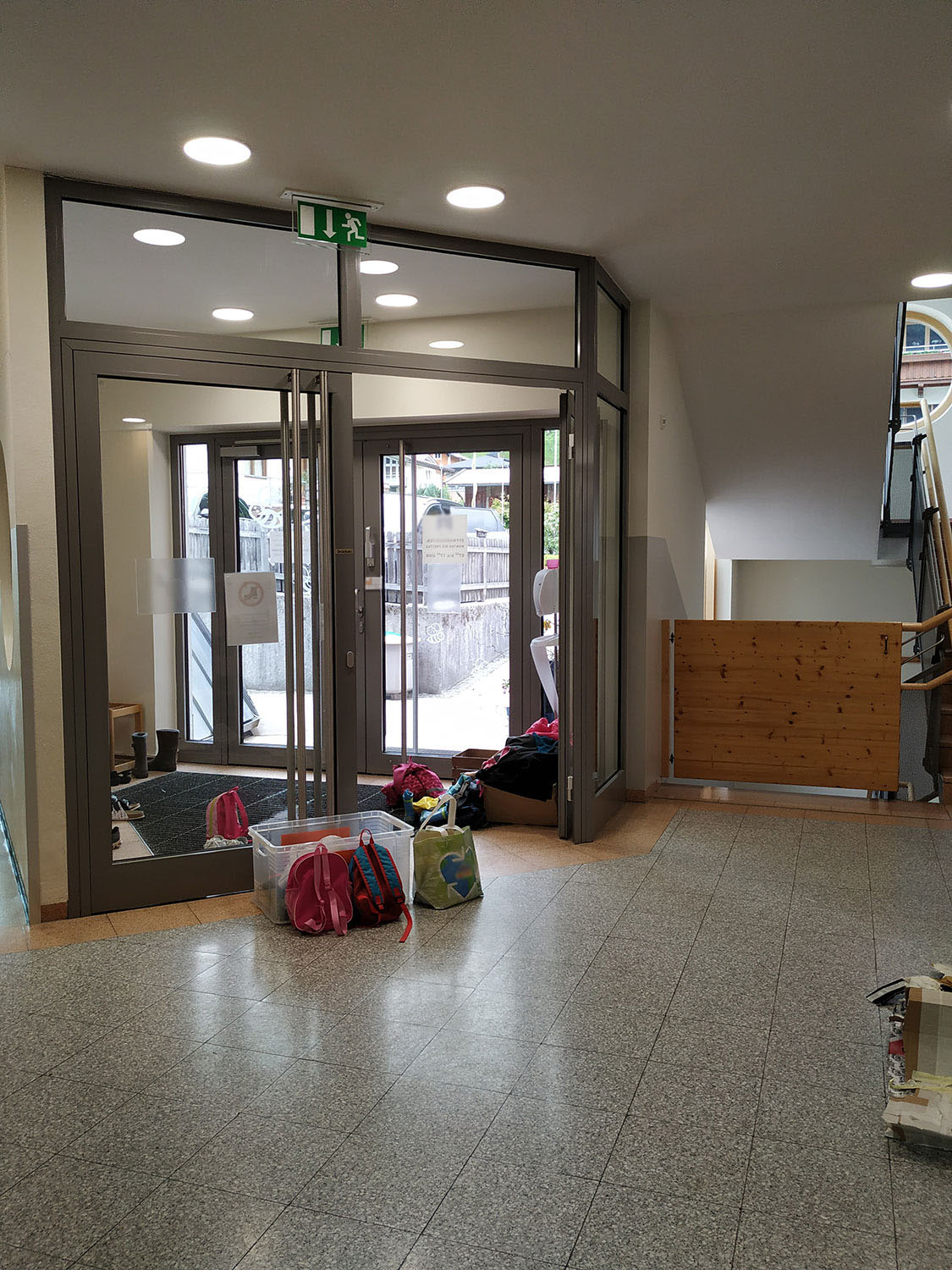

Supplement: Supplementary Data Sheet S5 — Photographic record of building tour. [file Data_Sheet_5.zip › common areas/hall_1.jpg]

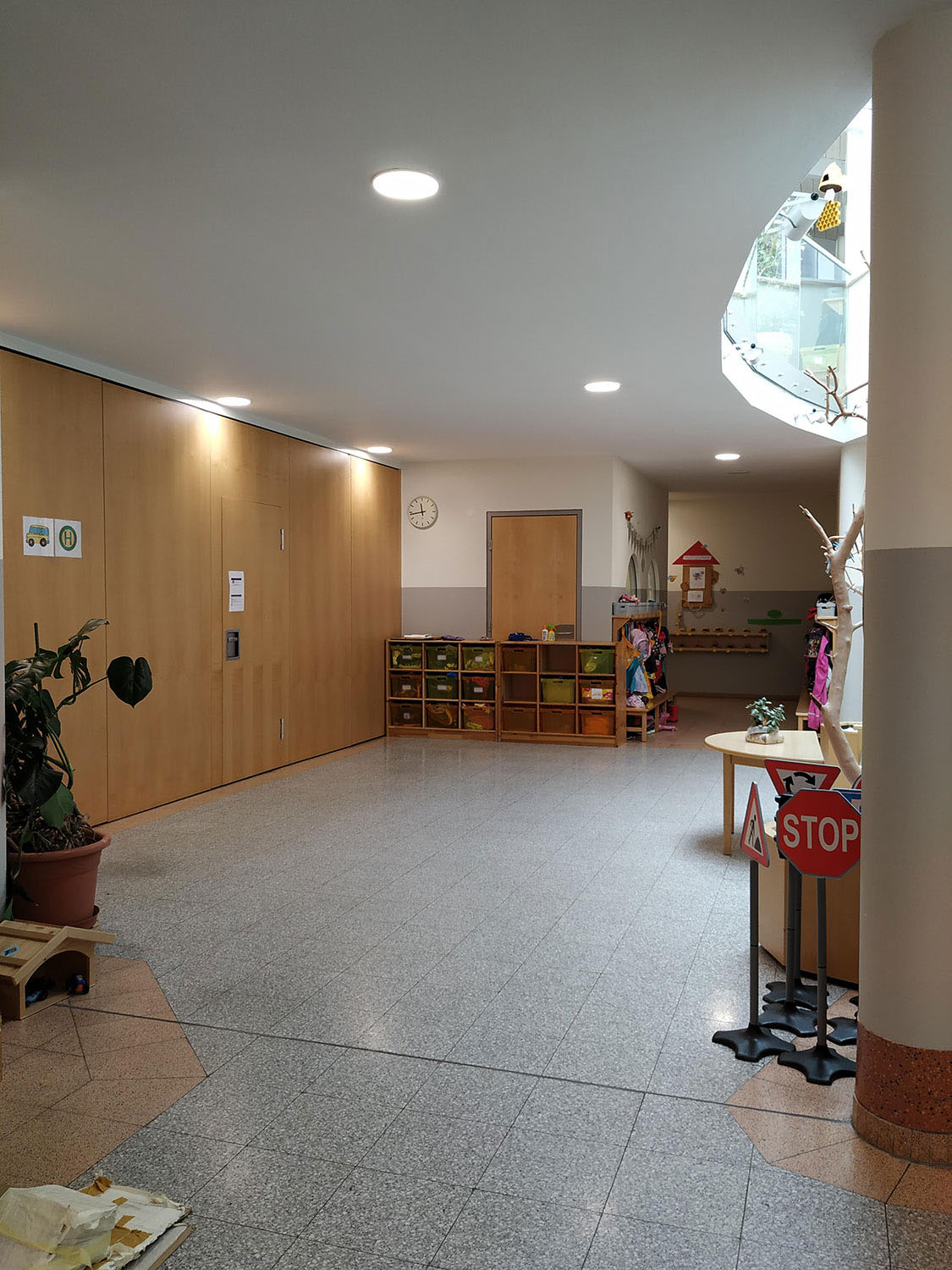

Supplement: Supplementary Data Sheet S5 — Photographic record of building tour. [file Data_Sheet_5.zip › common areas/hall_2.jpg]

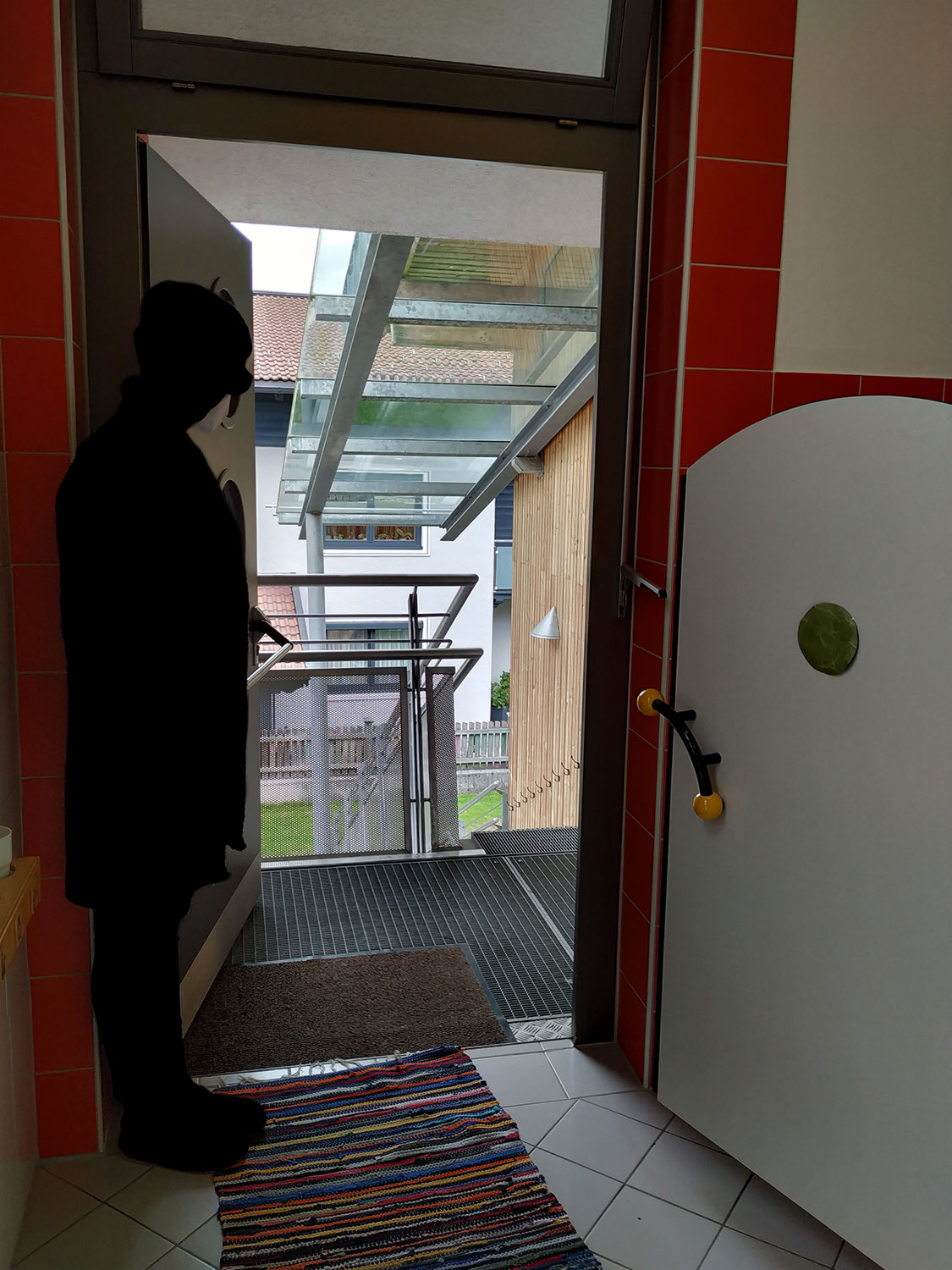

Supplement: Supplementary Data Sheet S5 — Photographic record of building tour. [file Data_Sheet_5.zip › homebase areas/group-exit_1.jpg]

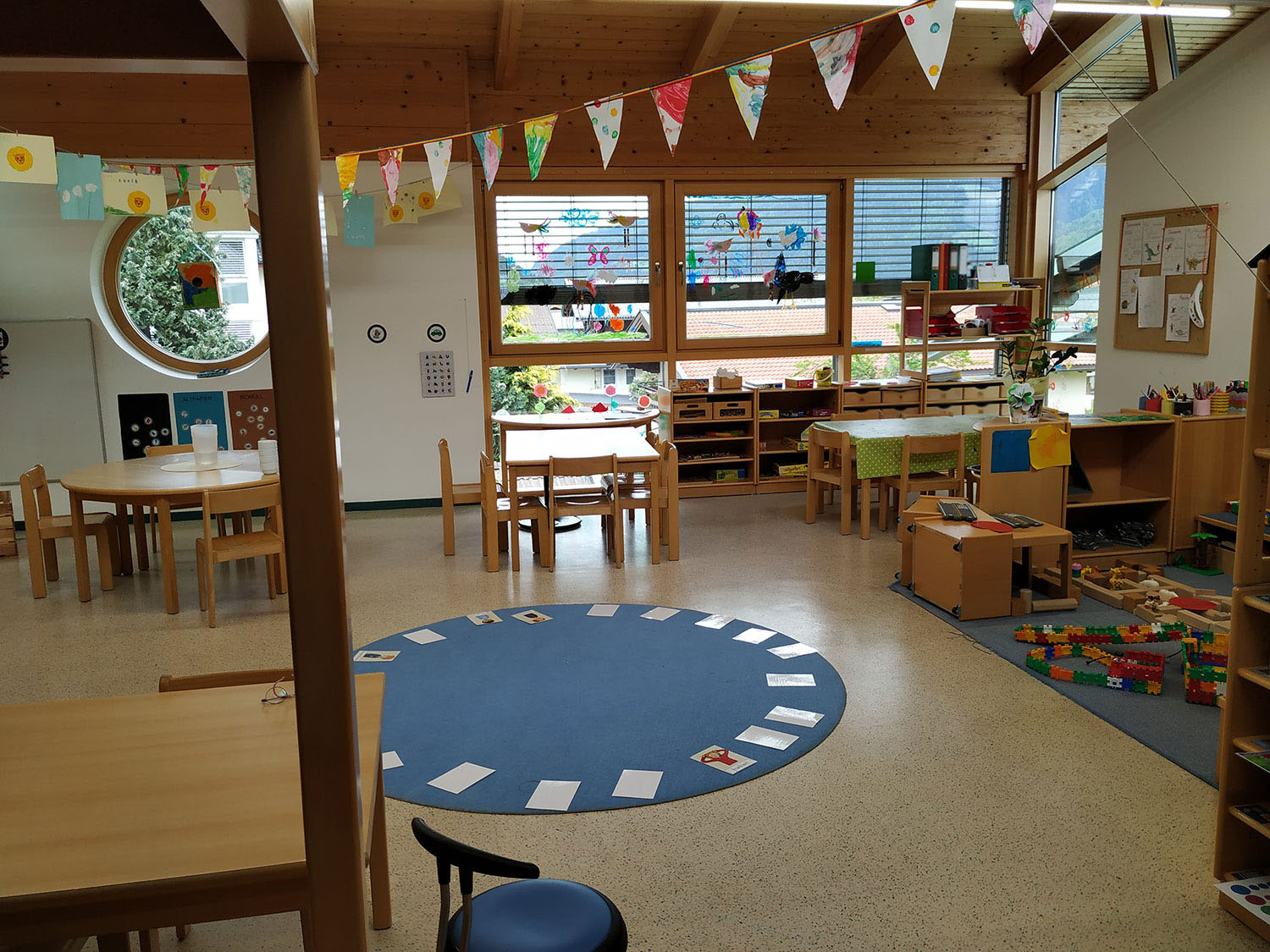

Supplement: Supplementary Data Sheet S5 — Photographic record of building tour. [file Data_Sheet_5.zip › homebase areas/group-room_1.jpg]

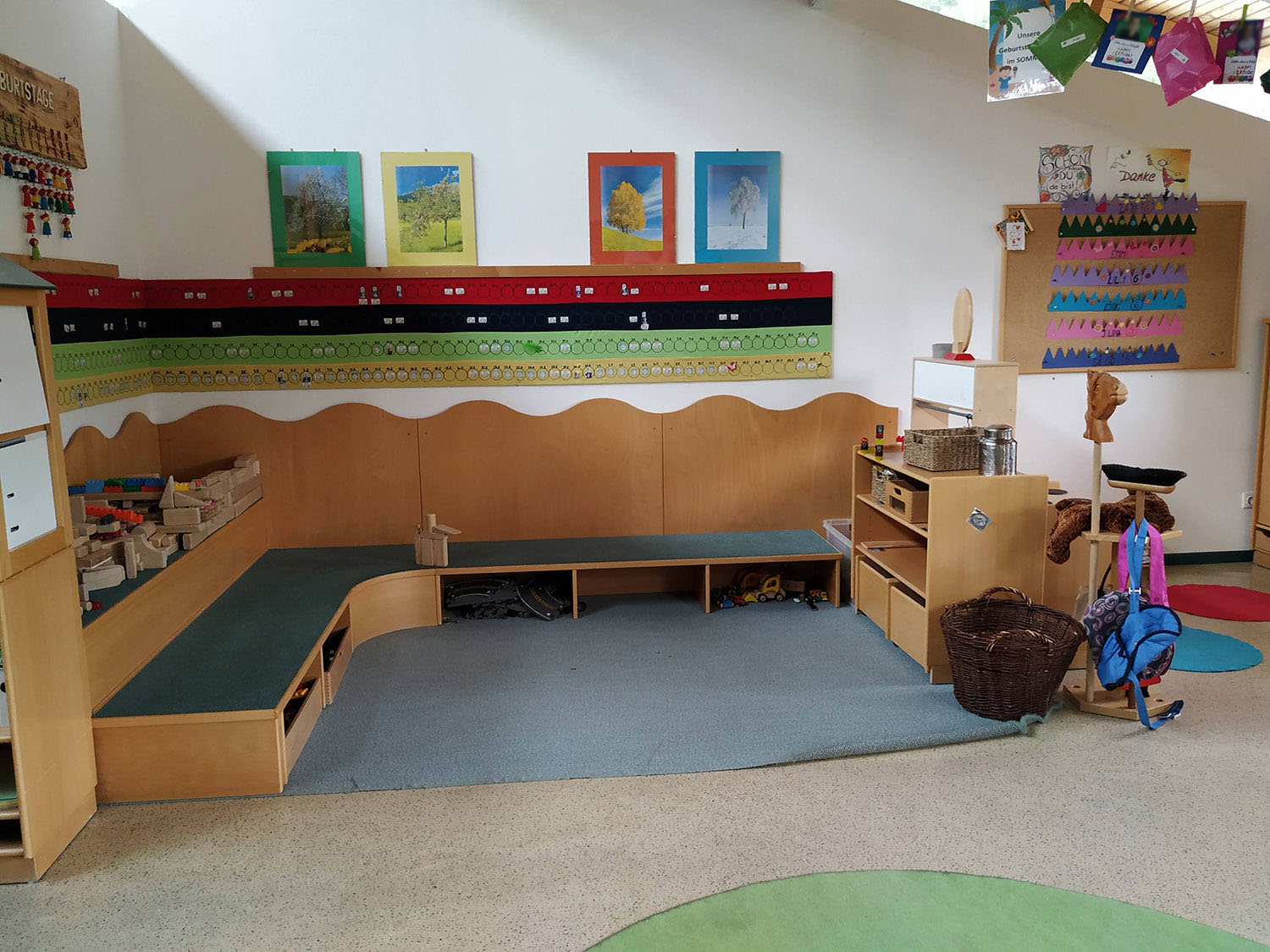

Supplement: Supplementary Data Sheet S5 — Photographic record of building tour. [file Data_Sheet_5.zip › homebase areas/group-room_10.jpg]

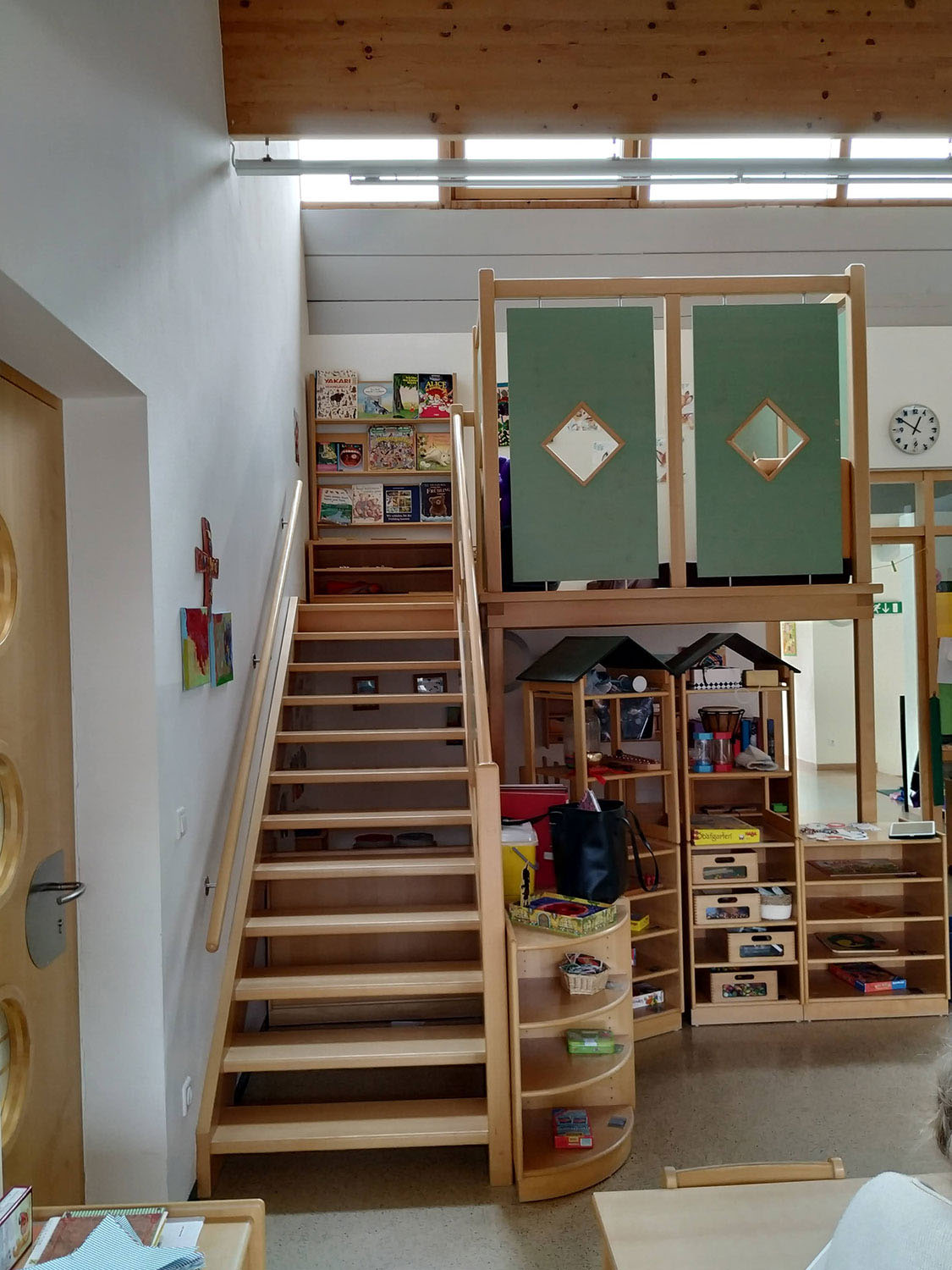

Supplement: Supplementary Data Sheet S5 — Photographic record of building tour. [file Data_Sheet_5.zip › homebase areas/group-room_11.jpg]

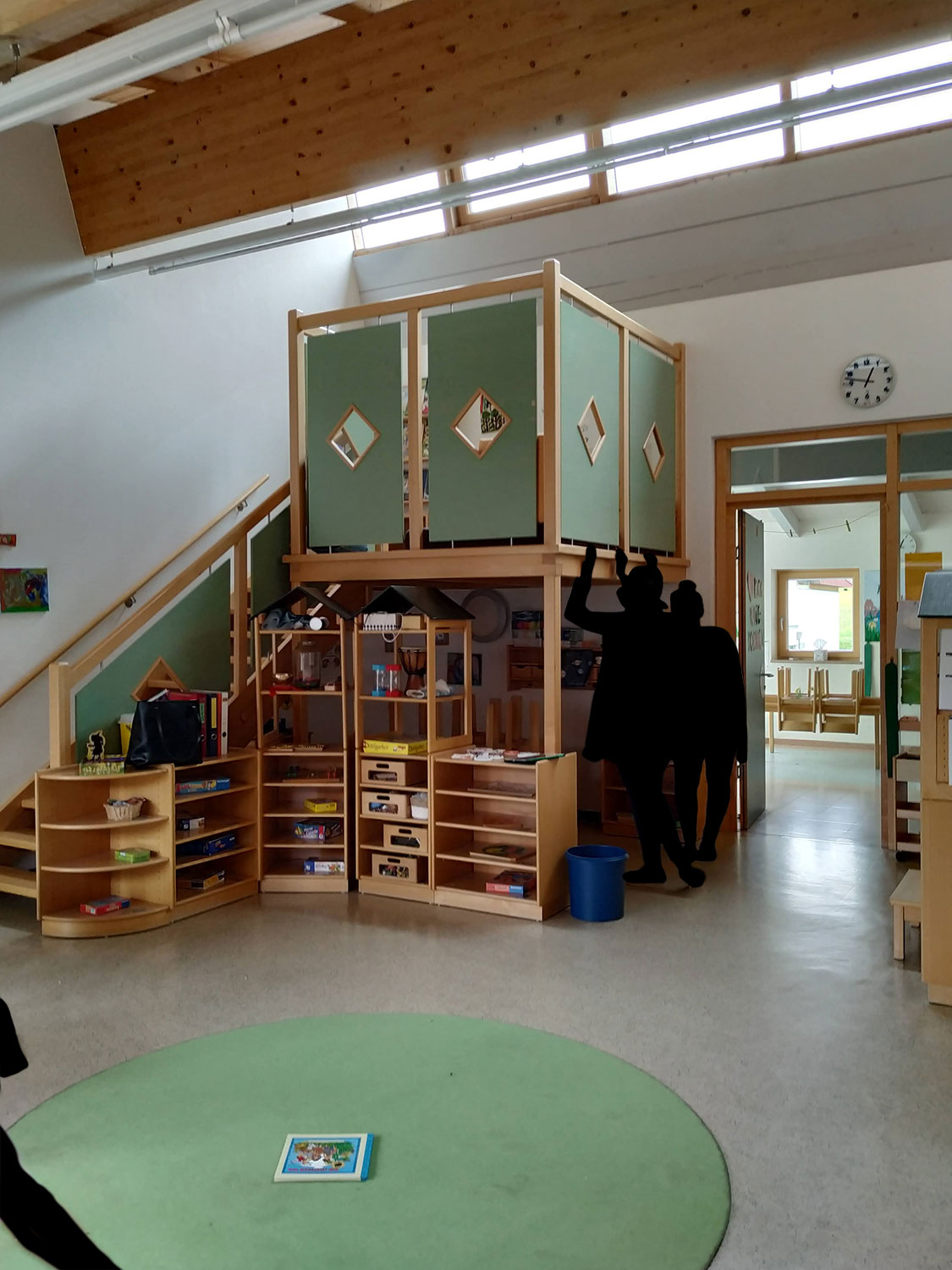

Supplement: Supplementary Data Sheet S5 — Photographic record of building tour. [file Data_Sheet_5.zip › homebase areas/group-room_12.jpg]

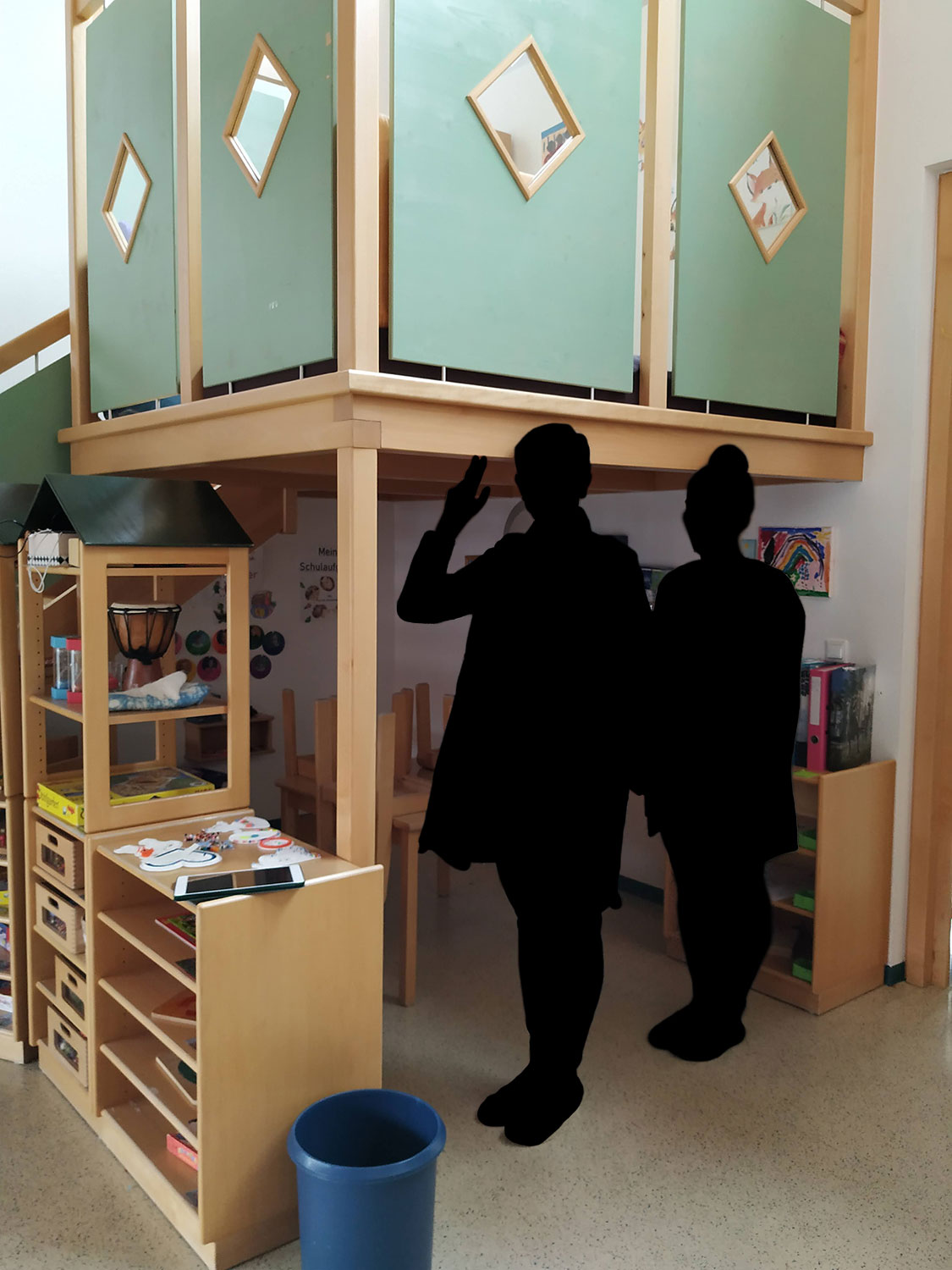

Supplement: Supplementary Data Sheet S5 — Photographic record of building tour. [file Data_Sheet_5.zip › homebase areas/group-room_13.jpg]

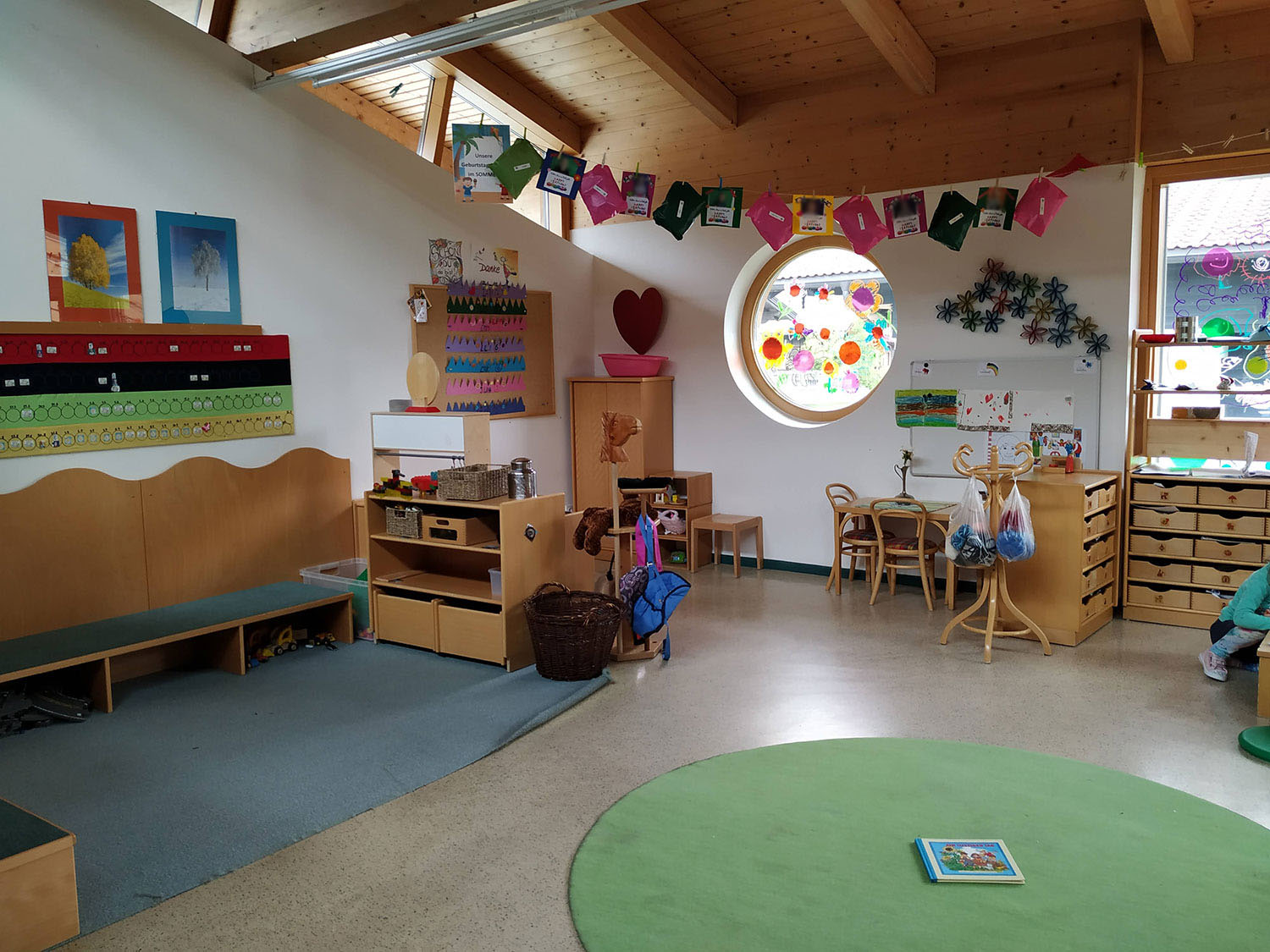

Supplement: Supplementary Data Sheet S5 — Photographic record of building tour. [file Data_Sheet_5.zip › homebase areas/group-room_14.jpg]

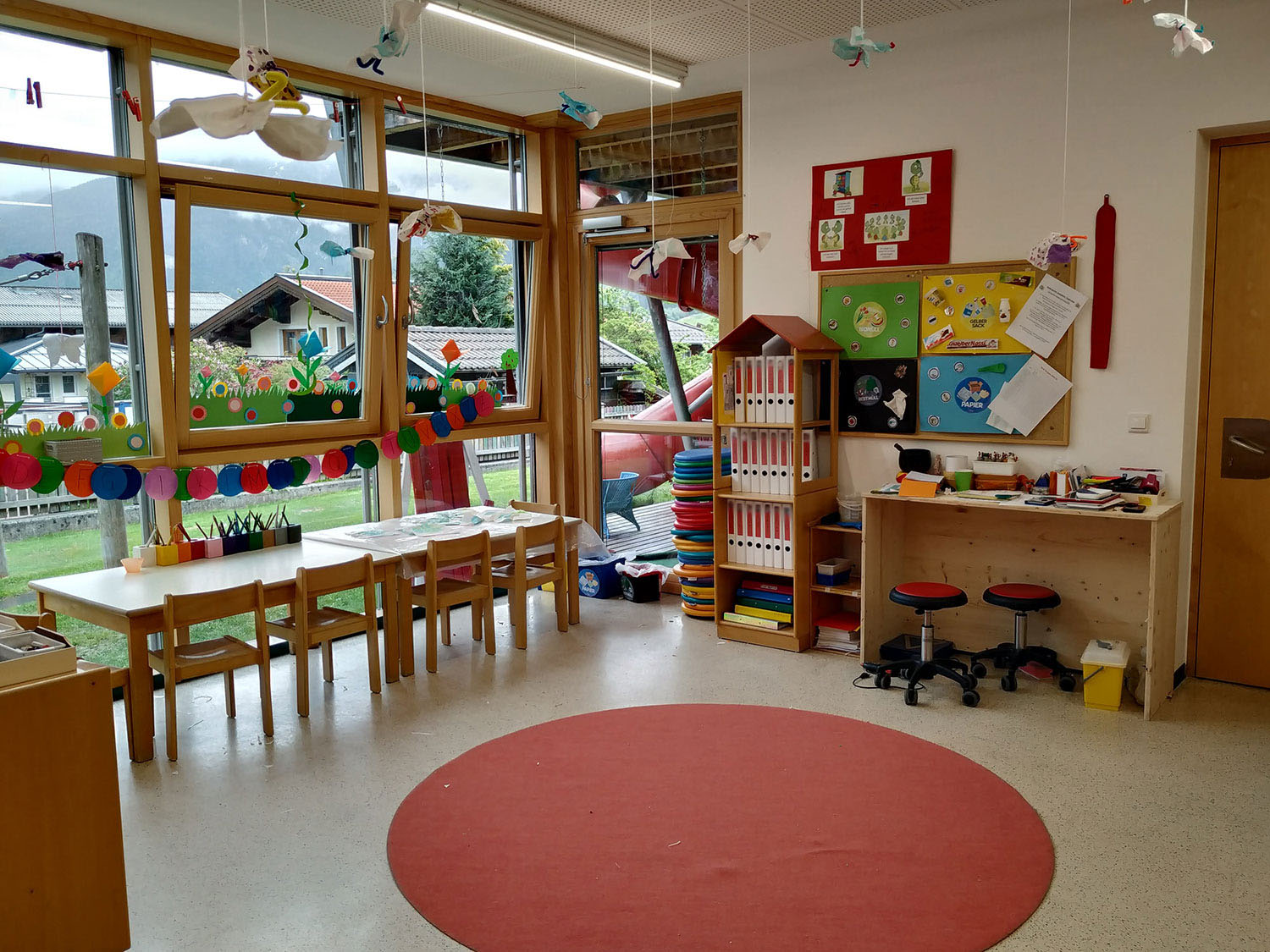

Supplement: Supplementary Data Sheet S5 — Photographic record of building tour. [file Data_Sheet_5.zip › homebase areas/group-room_15.jpg]

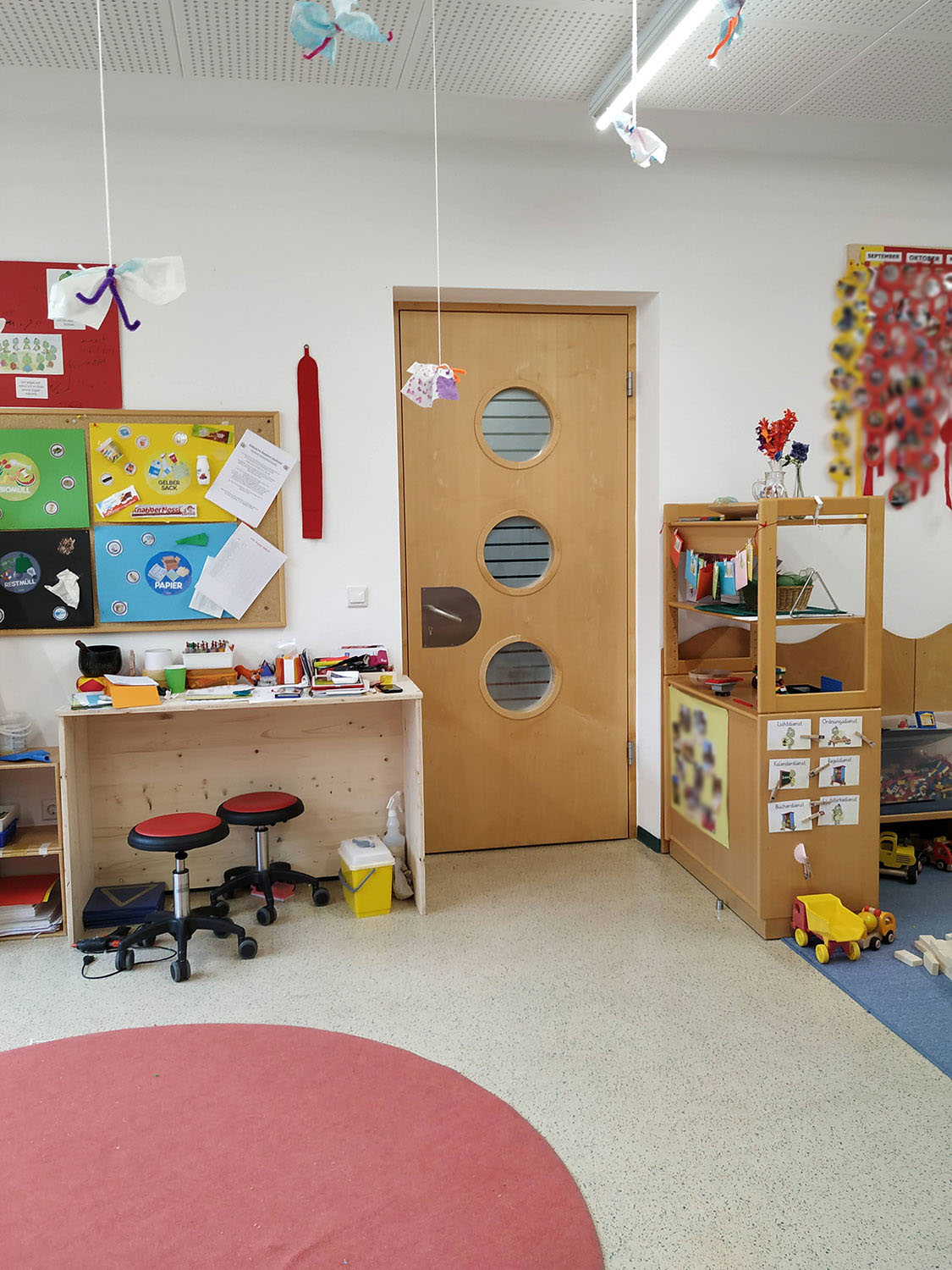

Supplement: Supplementary Data Sheet S5 — Photographic record of building tour. [file Data_Sheet_5.zip › homebase areas/group-room_16.jpg]

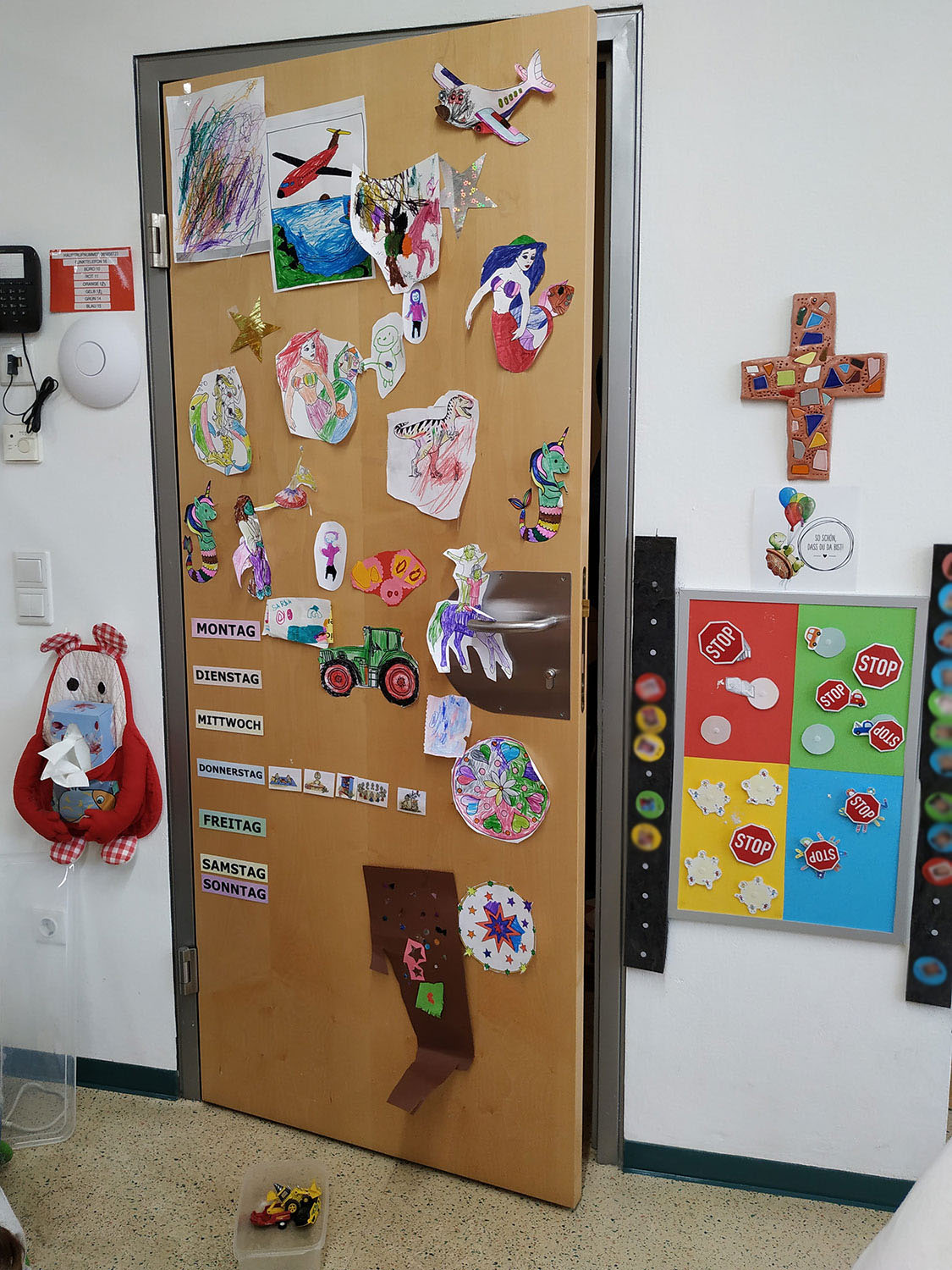

Supplement: Supplementary Data Sheet S5 — Photographic record of building tour. [file Data_Sheet_5.zip › homebase areas/group-room_17.jpg]

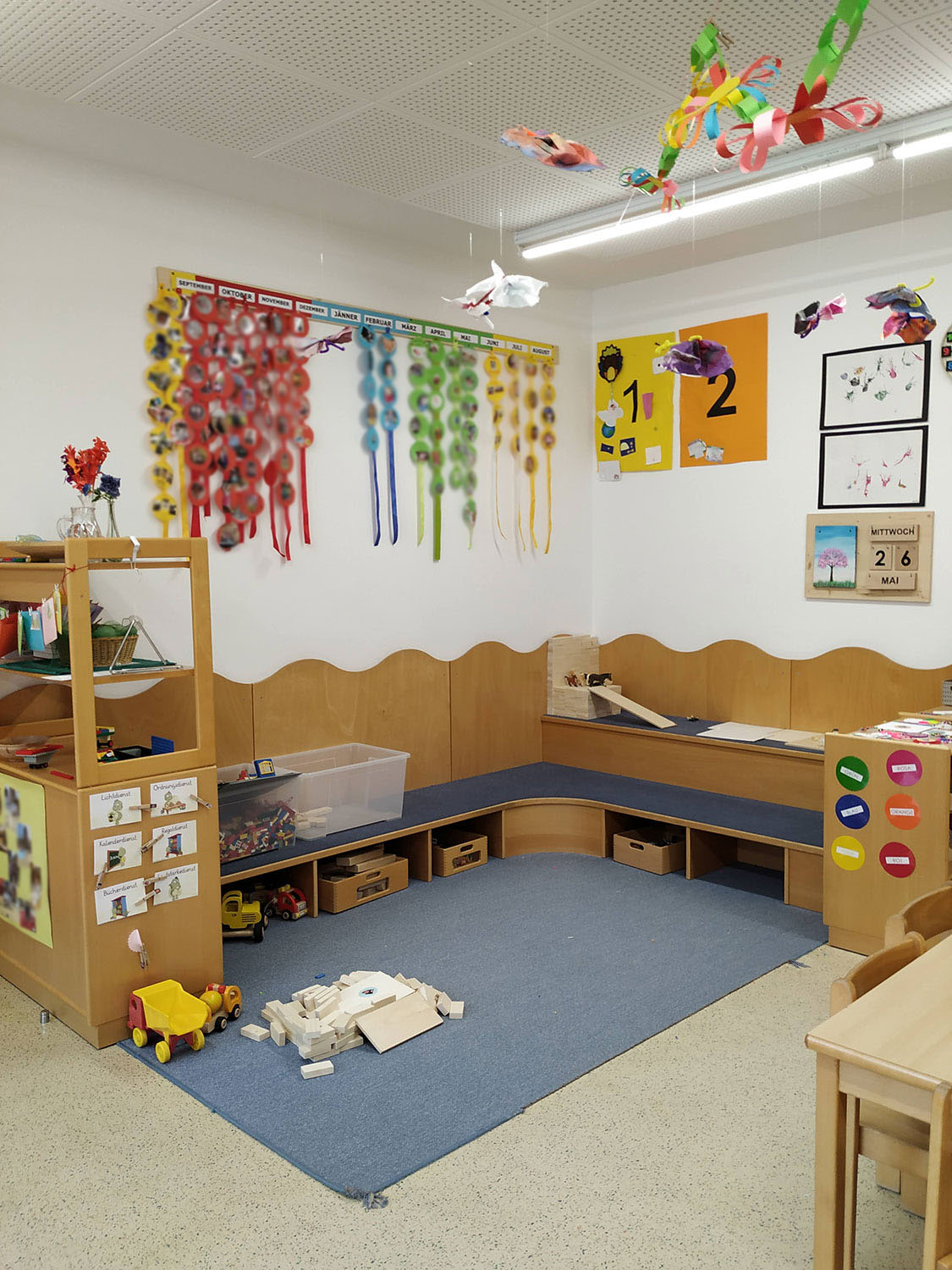

Supplement: Supplementary Data Sheet S5 — Photographic record of building tour. [file Data_Sheet_5.zip › homebase areas/group-room_18.jpg]

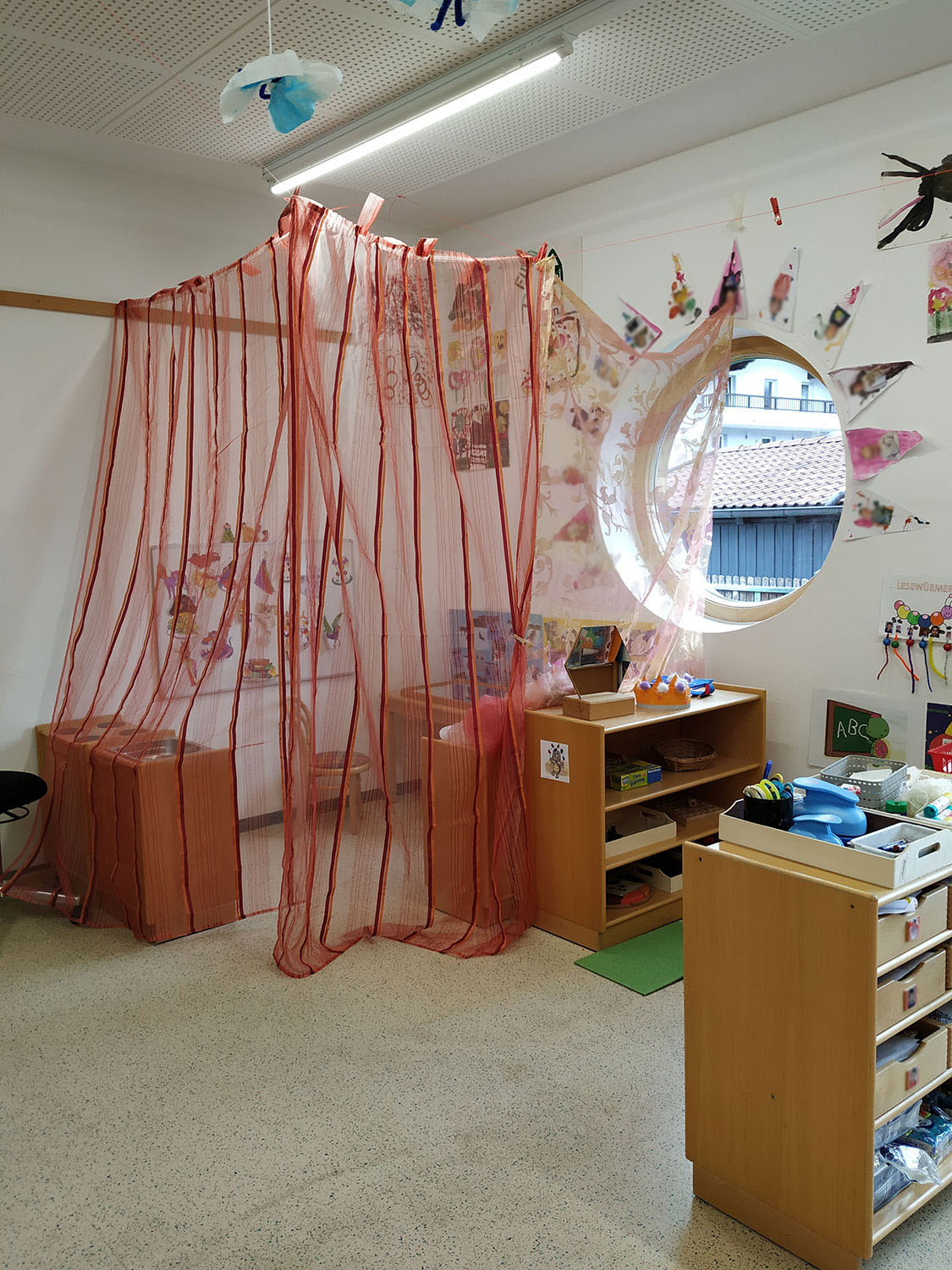

Supplement: Supplementary Data Sheet S5 — Photographic record of building tour. [file Data_Sheet_5.zip › homebase areas/group-room_19.jpg]

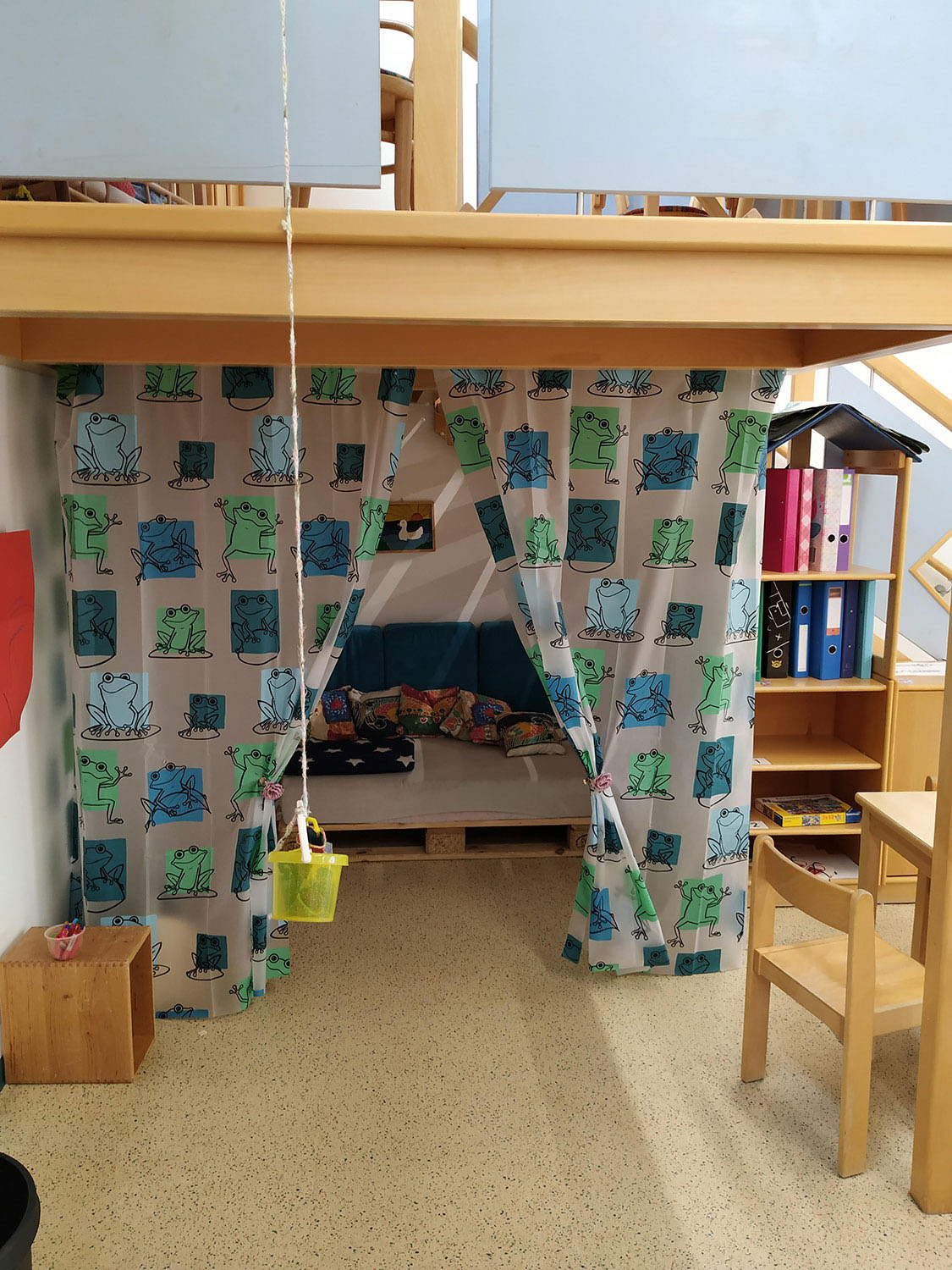

Supplement: Supplementary Data Sheet S5 — Photographic record of building tour. [file Data_Sheet_5.zip › homebase areas/group-room_2.jpg]

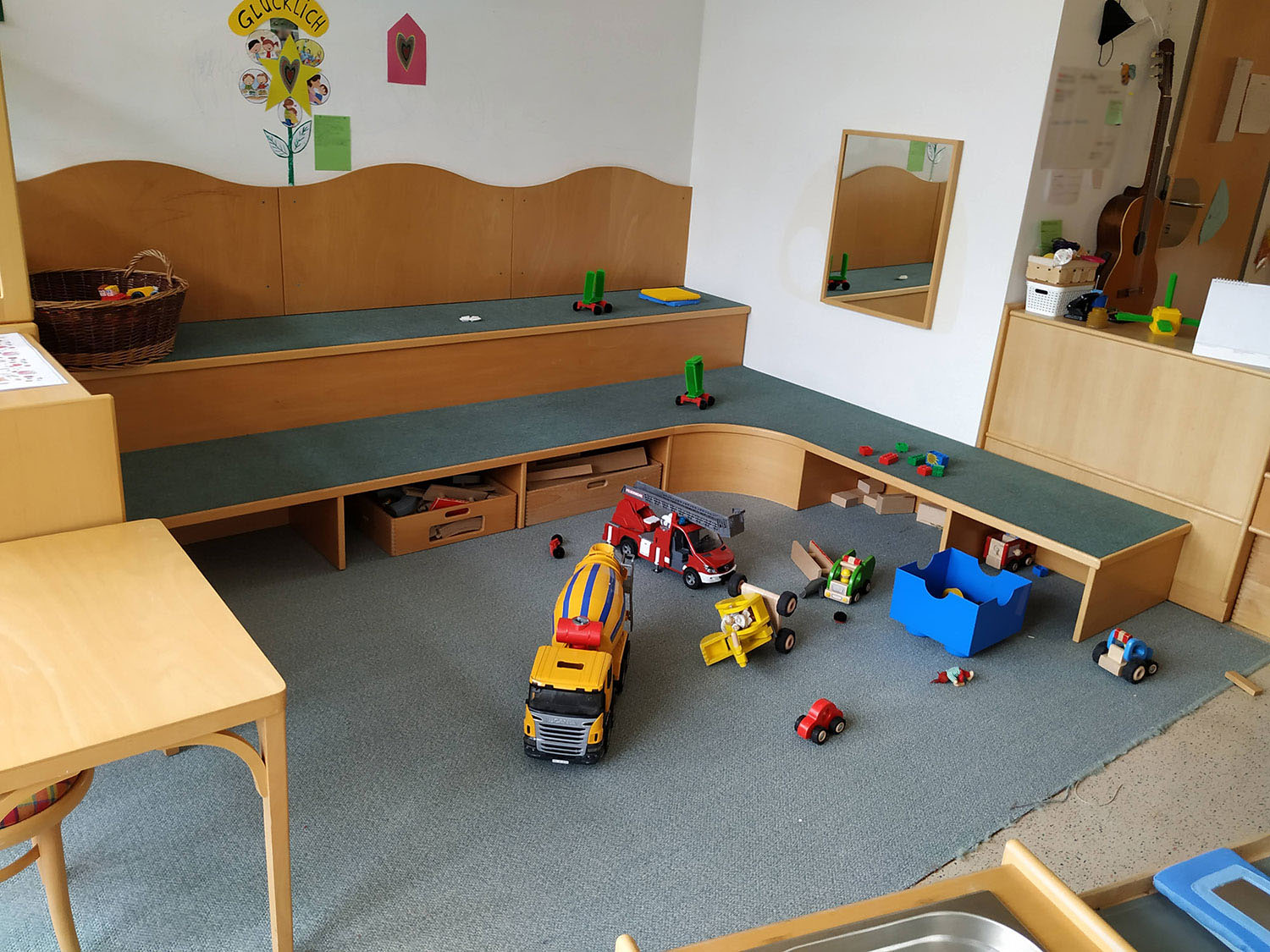

Supplement: Supplementary Data Sheet S5 — Photographic record of building tour. [file Data_Sheet_5.zip › homebase areas/group-room_20.jpg]

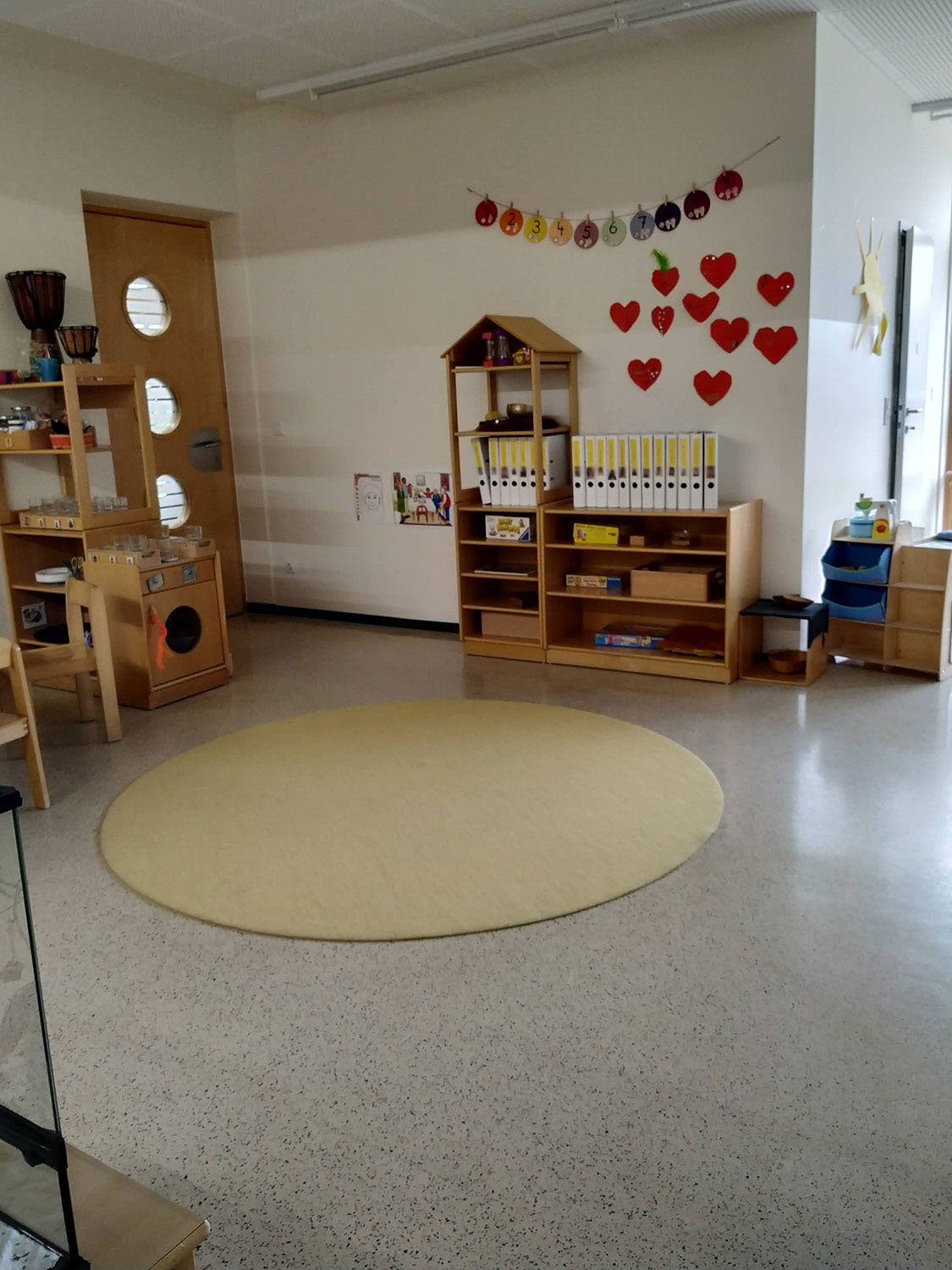

Supplement: Supplementary Data Sheet S5 — Photographic record of building tour. [file Data_Sheet_5.zip › homebase areas/group-room_21.jpg]

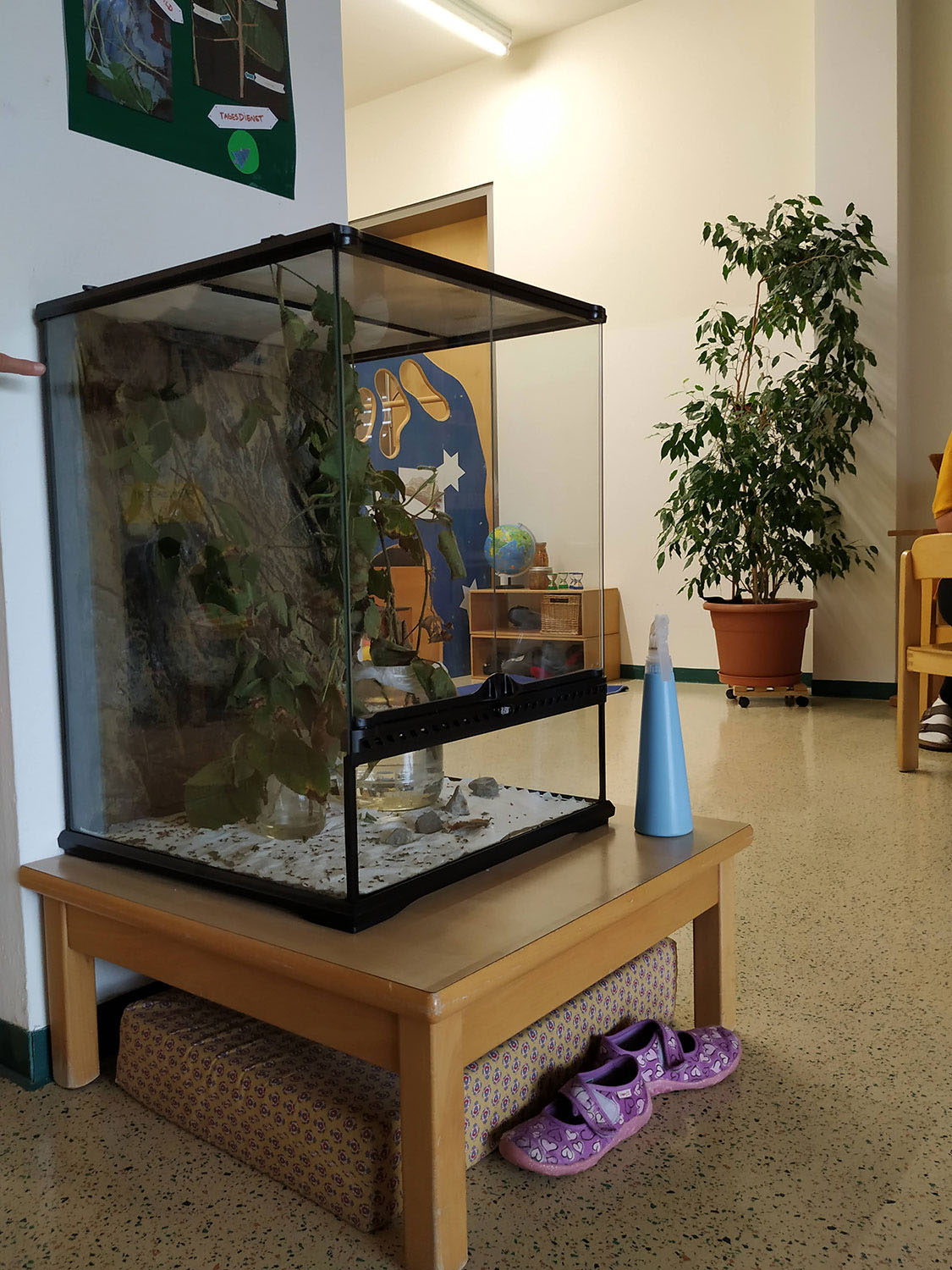

Supplement: Supplementary Data Sheet S5 — Photographic record of building tour. [file Data_Sheet_5.zip › homebase areas/group-room_22.jpg]

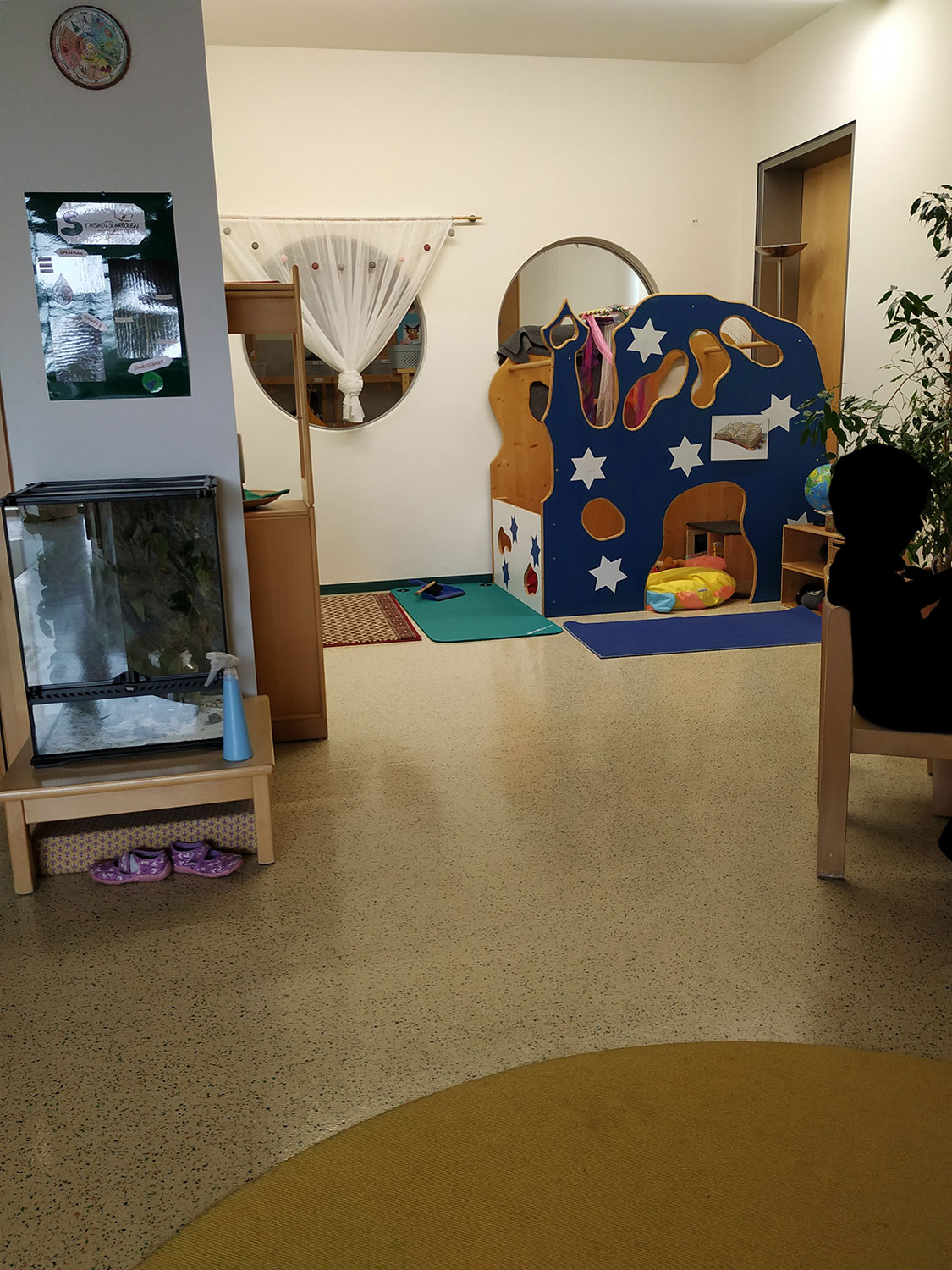

Supplement: Supplementary Data Sheet S5 — Photographic record of building tour. [file Data_Sheet_5.zip › homebase areas/group-room_23.jpg]

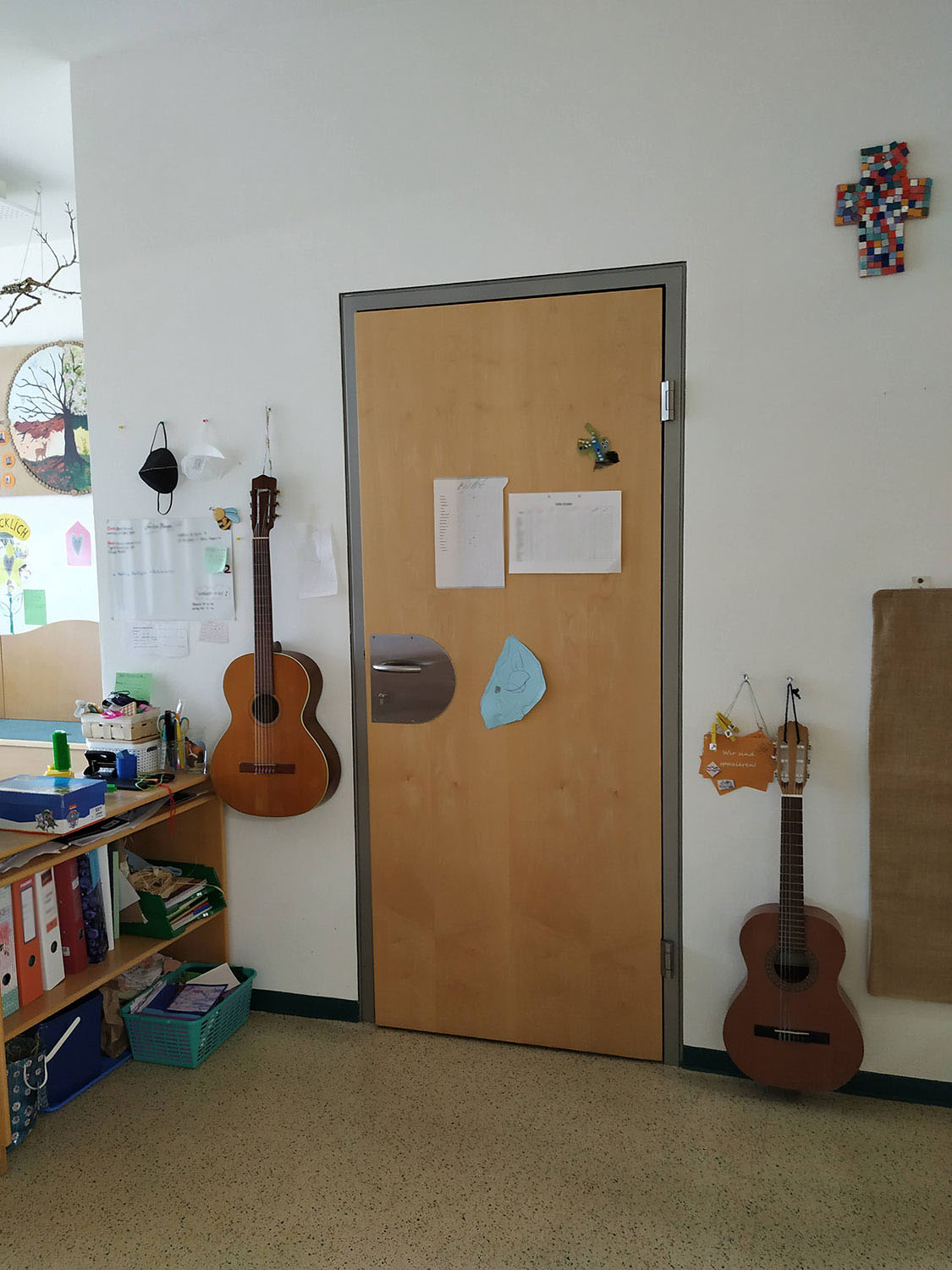

Supplement: Supplementary Data Sheet S5 — Photographic record of building tour. [file Data_Sheet_5.zip › homebase areas/group-room_24.jpg]

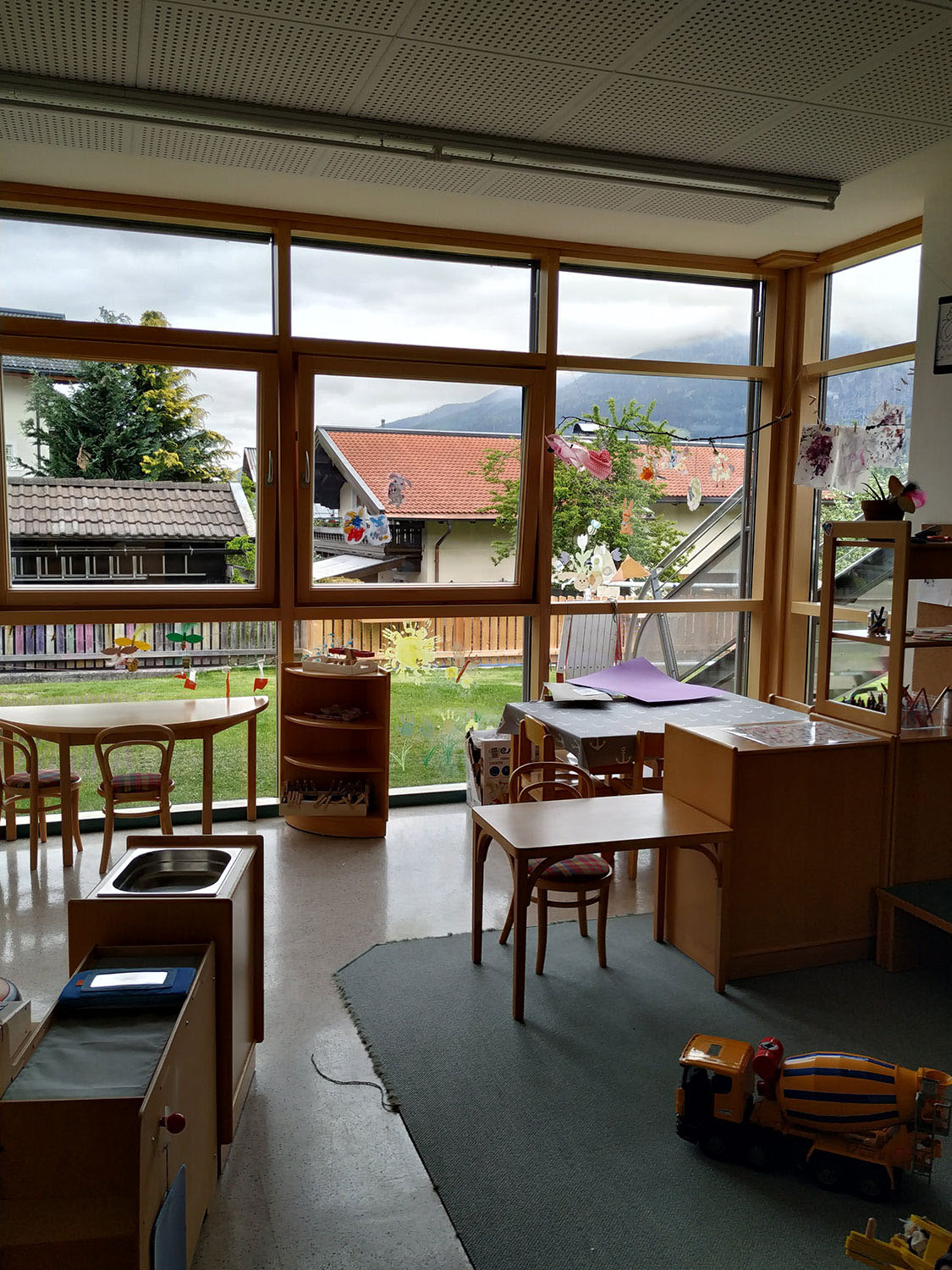

Supplement: Supplementary Data Sheet S5 — Photographic record of building tour. [file Data_Sheet_5.zip › homebase areas/group-room_25.jpg]

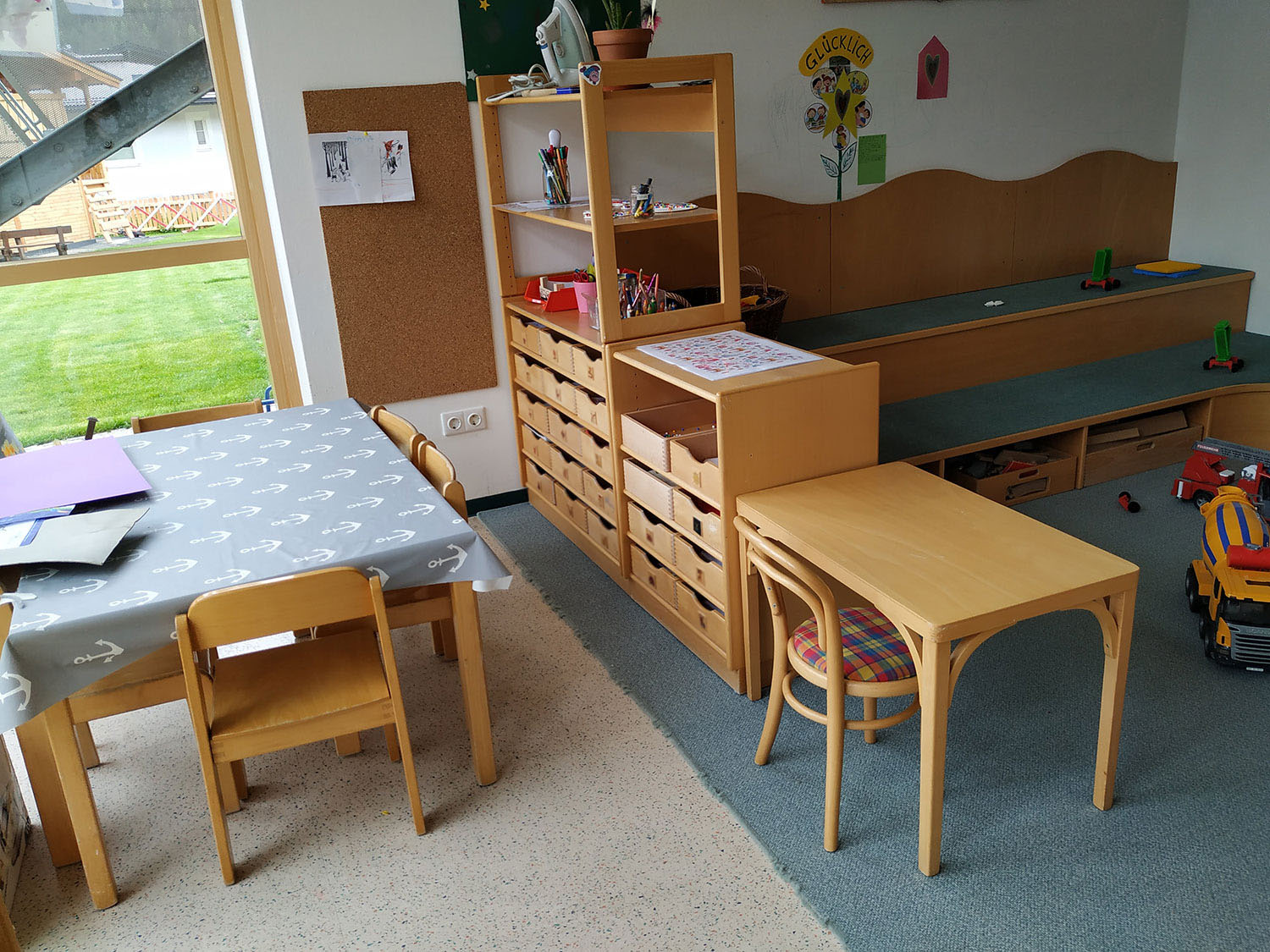

Supplement: Supplementary Data Sheet S5 — Photographic record of building tour. [file Data_Sheet_5.zip › homebase areas/group-room_26.jpg]

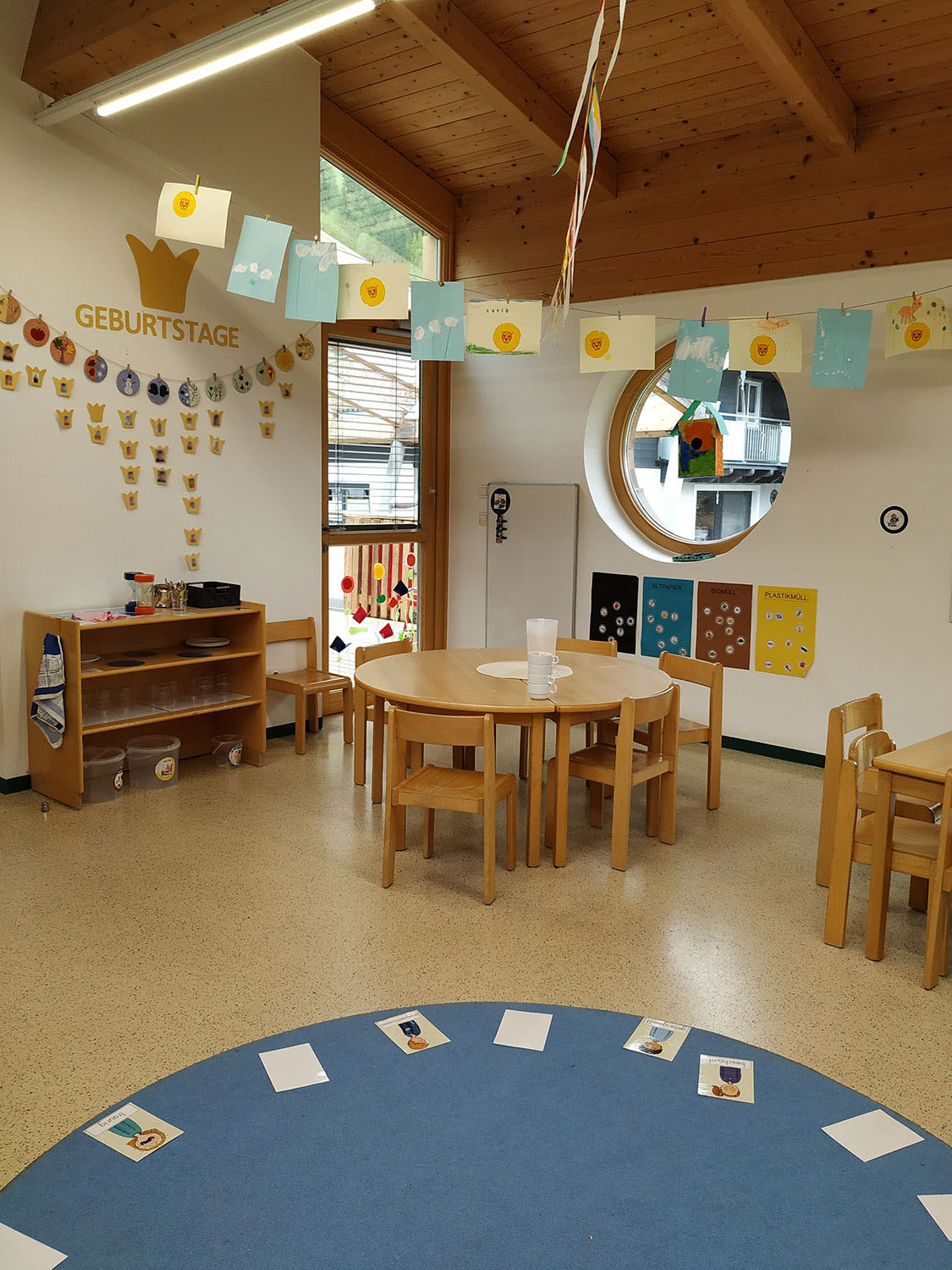

Supplement: Supplementary Data Sheet S5 — Photographic record of building tour. [file Data_Sheet_5.zip › homebase areas/group-room_3.jpg]

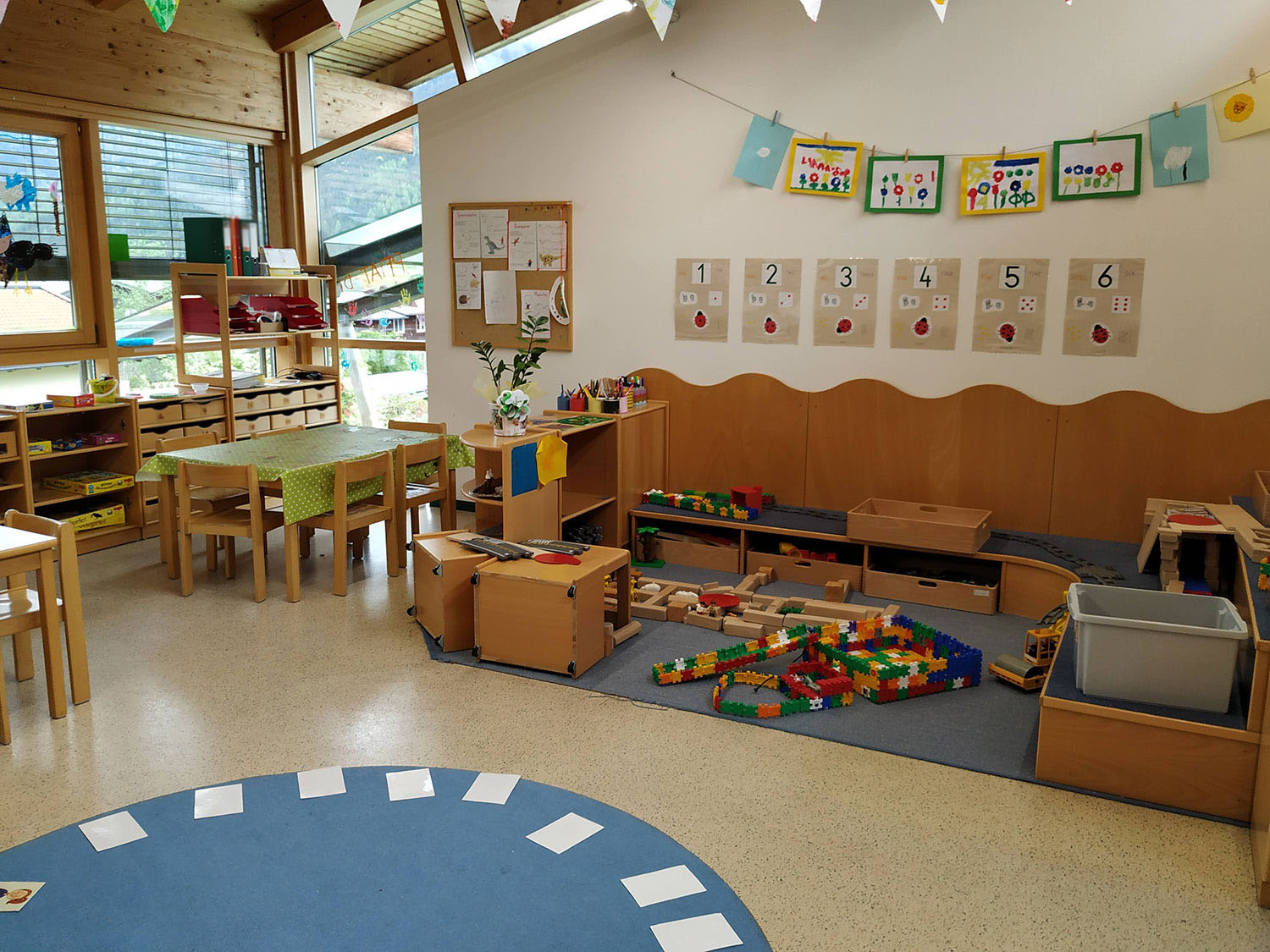

Supplement: Supplementary Data Sheet S5 — Photographic record of building tour. [file Data_Sheet_5.zip › homebase areas/group-room_4.jpg]

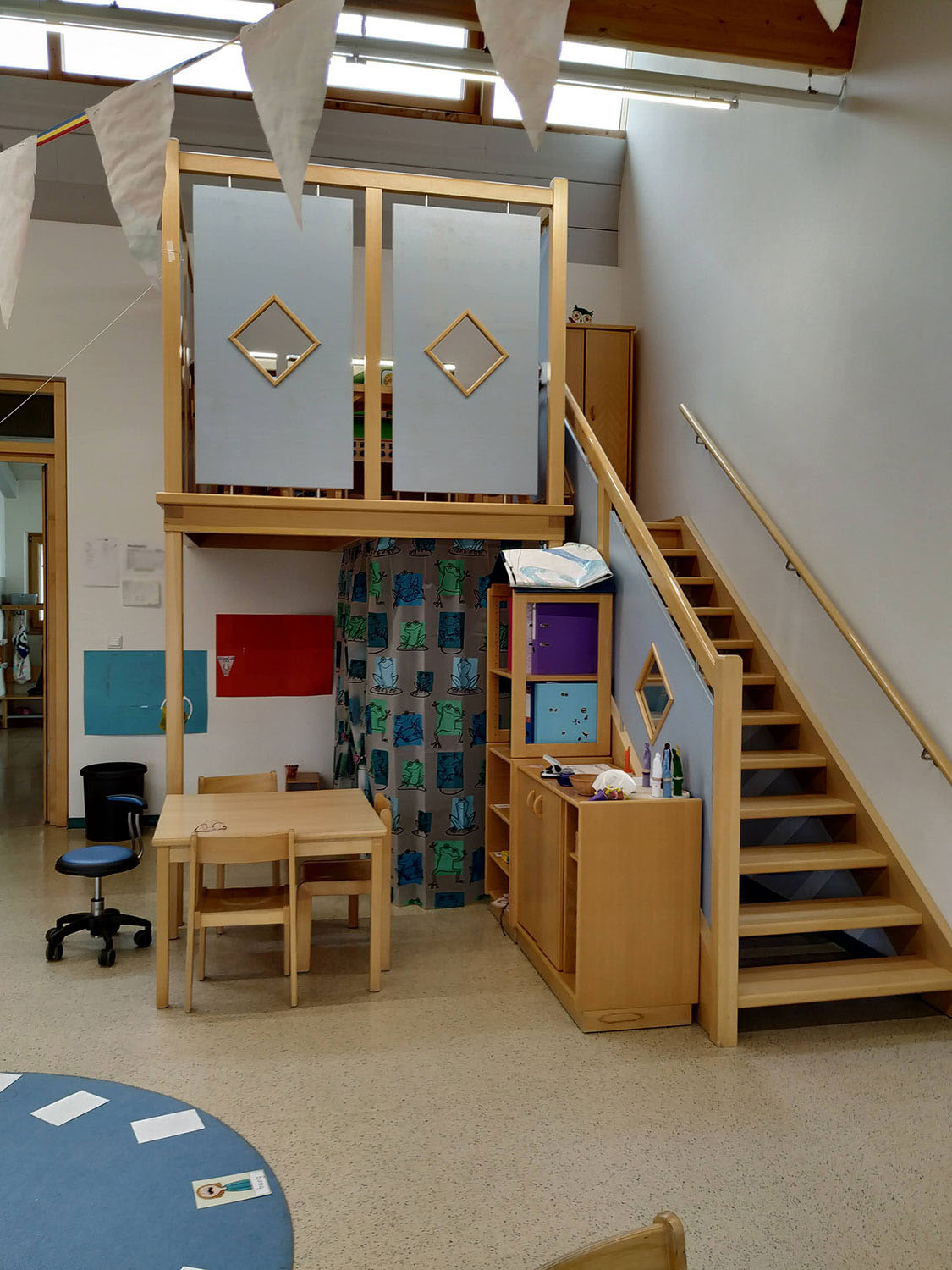

Supplement: Supplementary Data Sheet S5 — Photographic record of building tour. [file Data_Sheet_5.zip › homebase areas/group-room_5.jpg]

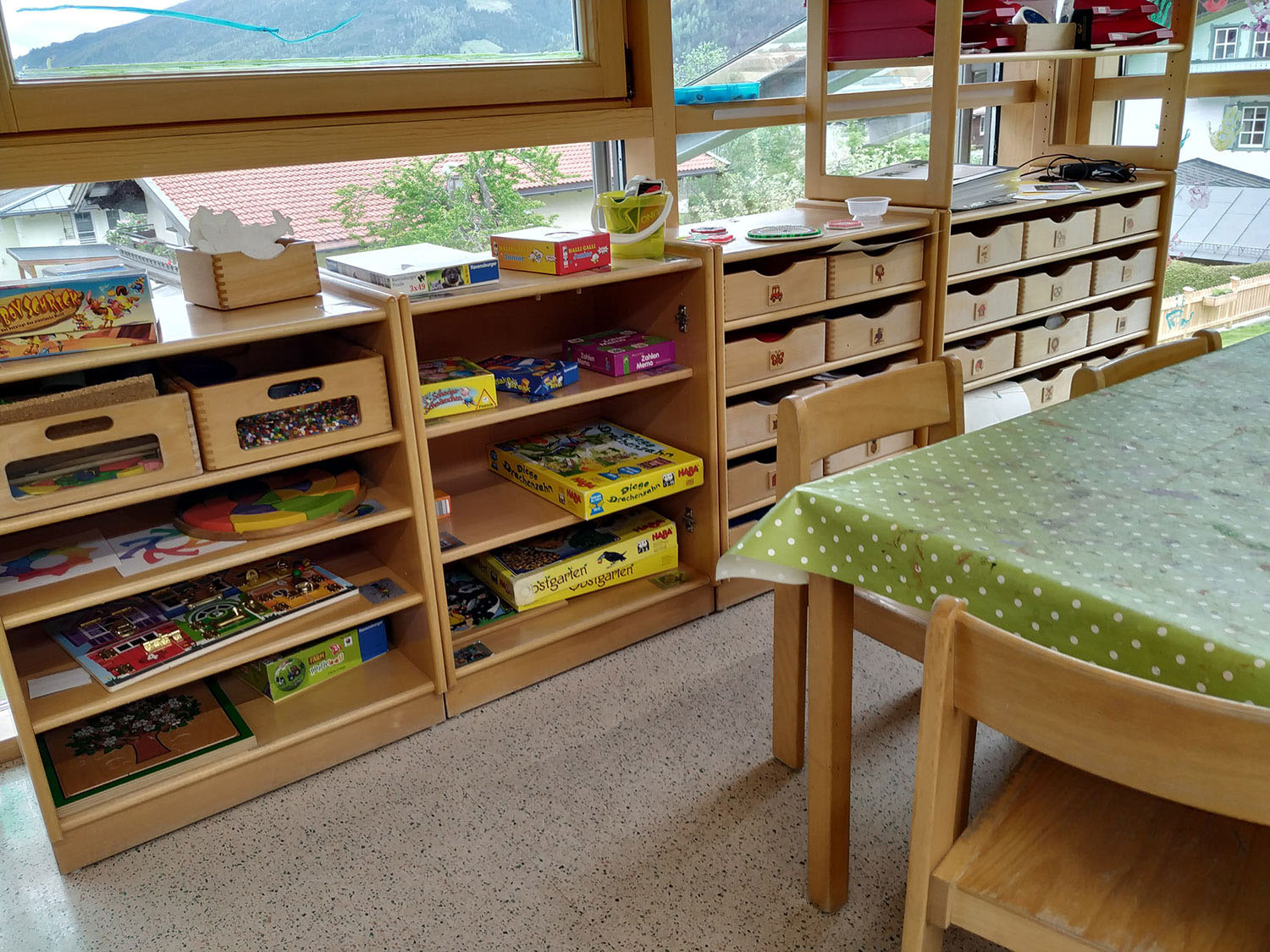

Supplement: Supplementary Data Sheet S5 — Photographic record of building tour. [file Data_Sheet_5.zip › homebase areas/group-room_6.jpg]

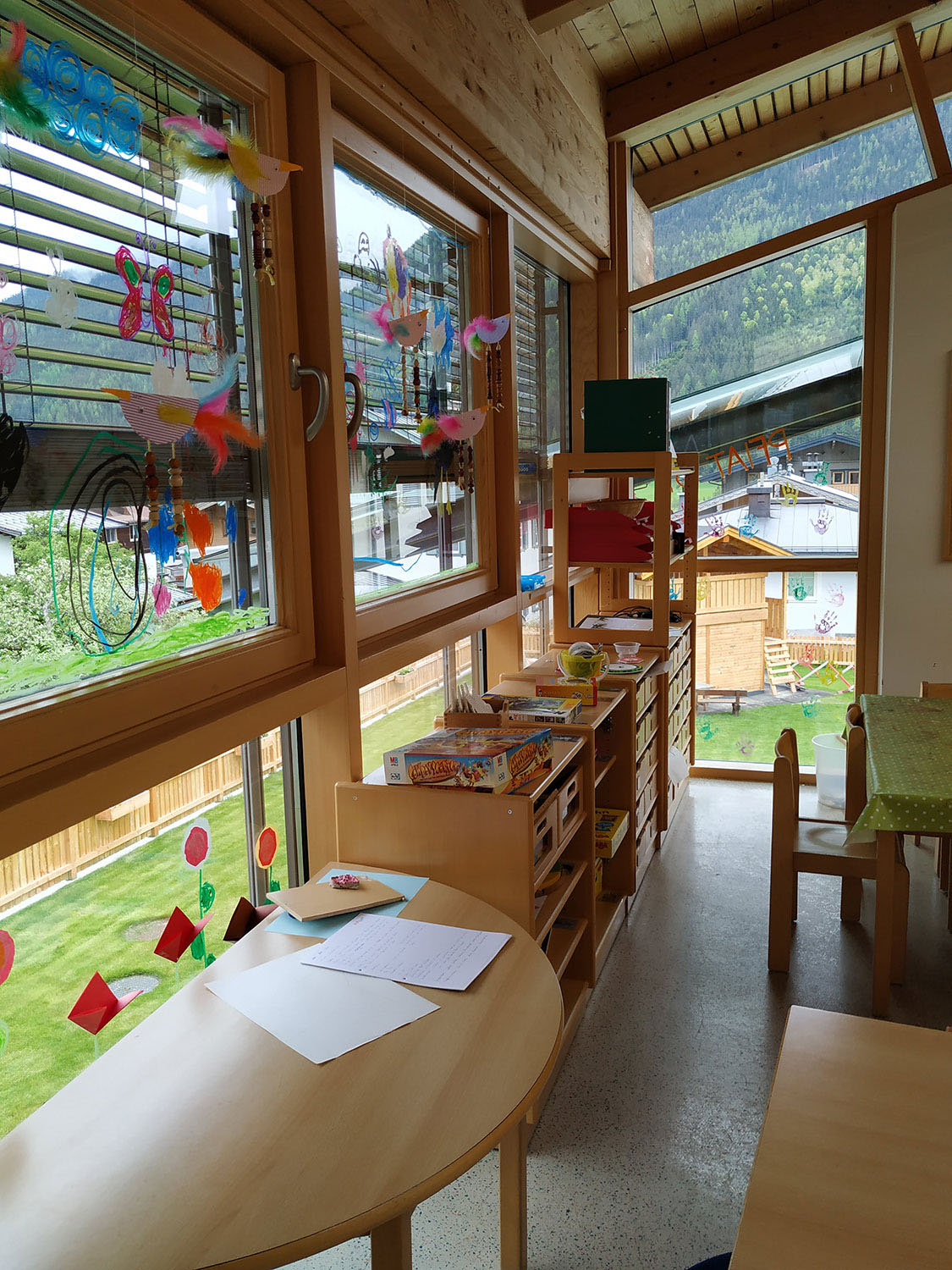

Supplement: Supplementary Data Sheet S5 — Photographic record of building tour. [file Data_Sheet_5.zip › homebase areas/group-room_7.jpg]

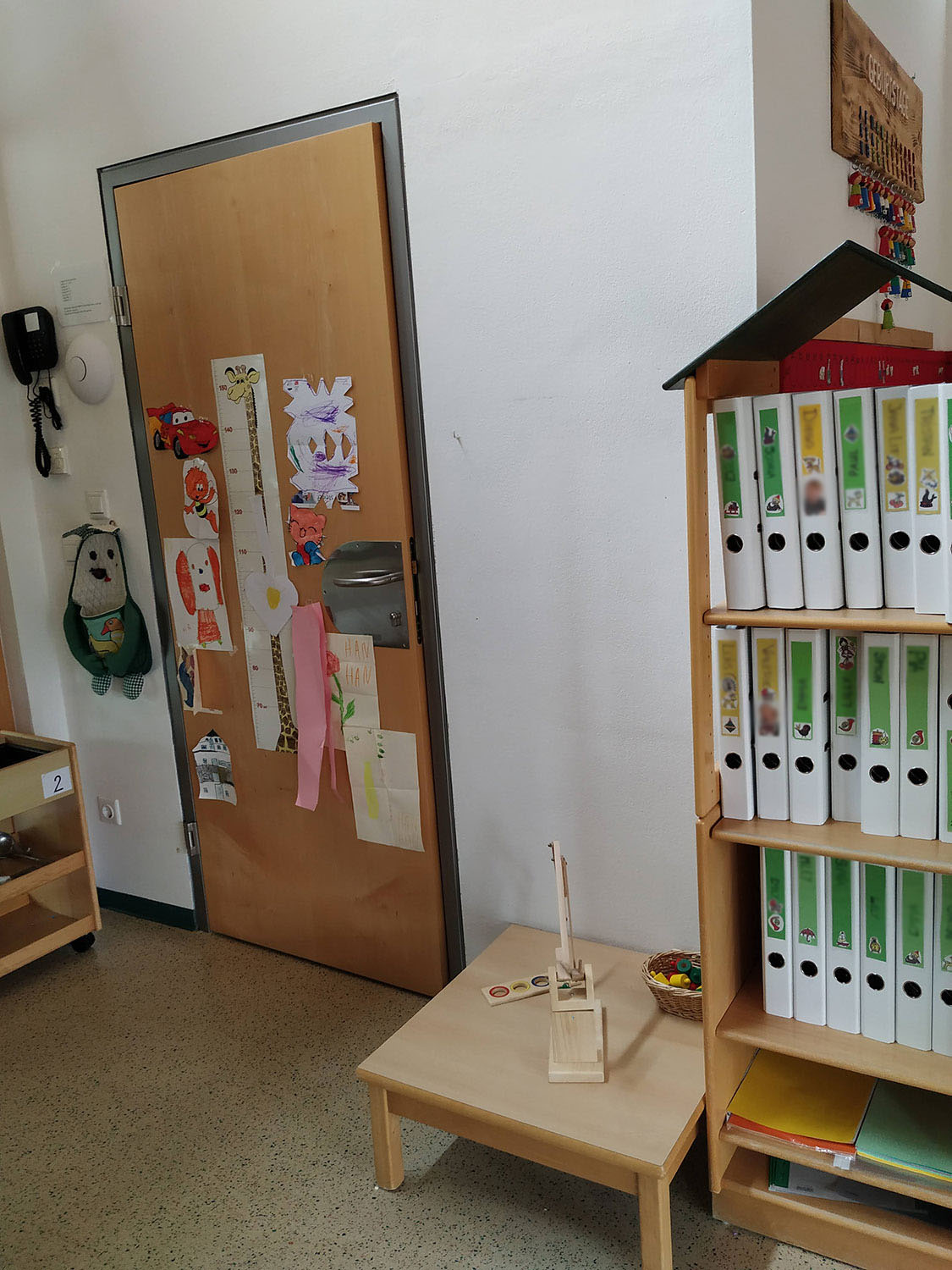

Supplement: Supplementary Data Sheet S5 — Photographic record of building tour. [file Data_Sheet_5.zip › homebase areas/group-room_8.jpg]

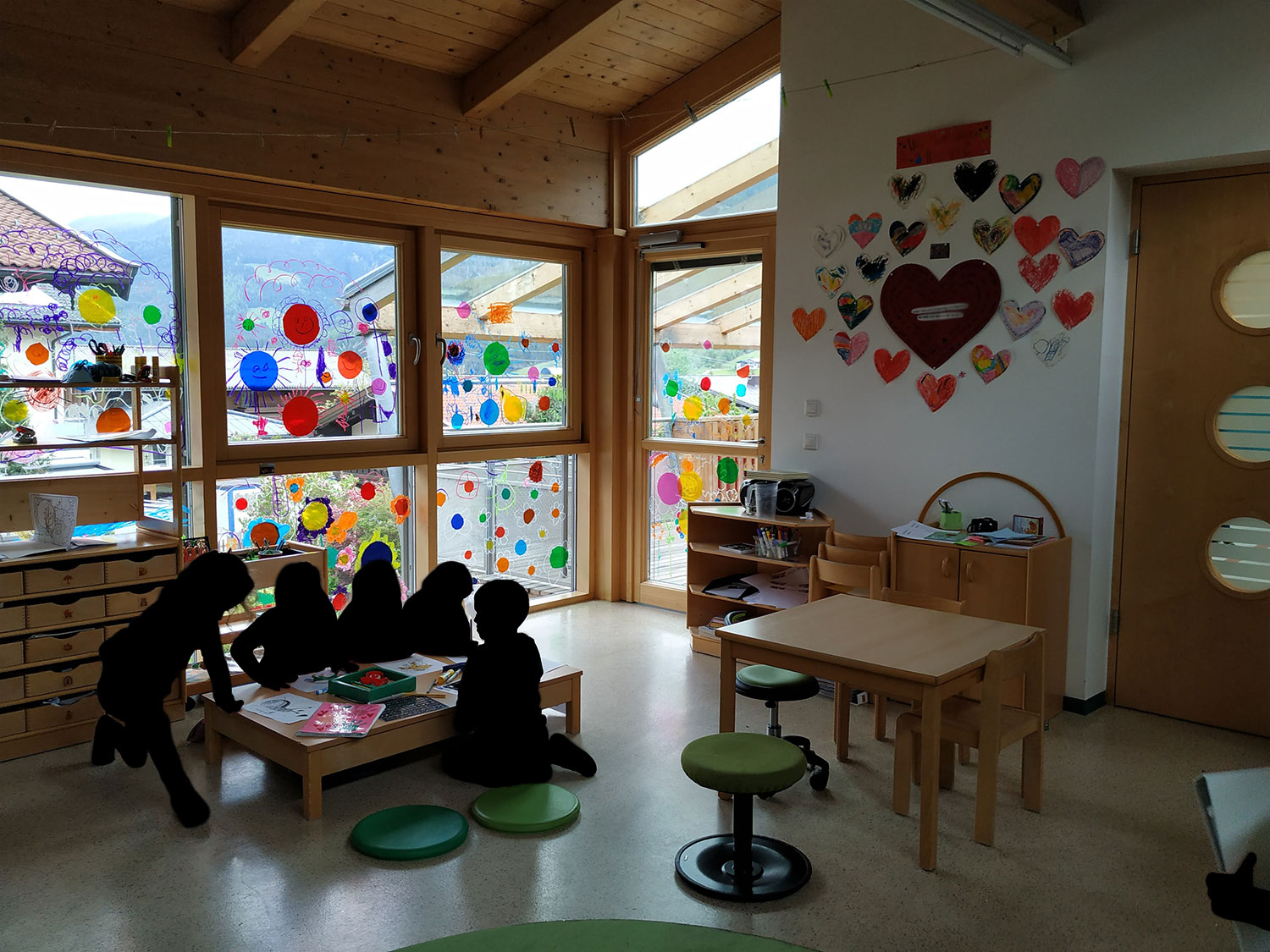

Supplement: Supplementary Data Sheet S5 — Photographic record of building tour. [file Data_Sheet_5.zip › homebase areas/group-room_9.jpg]

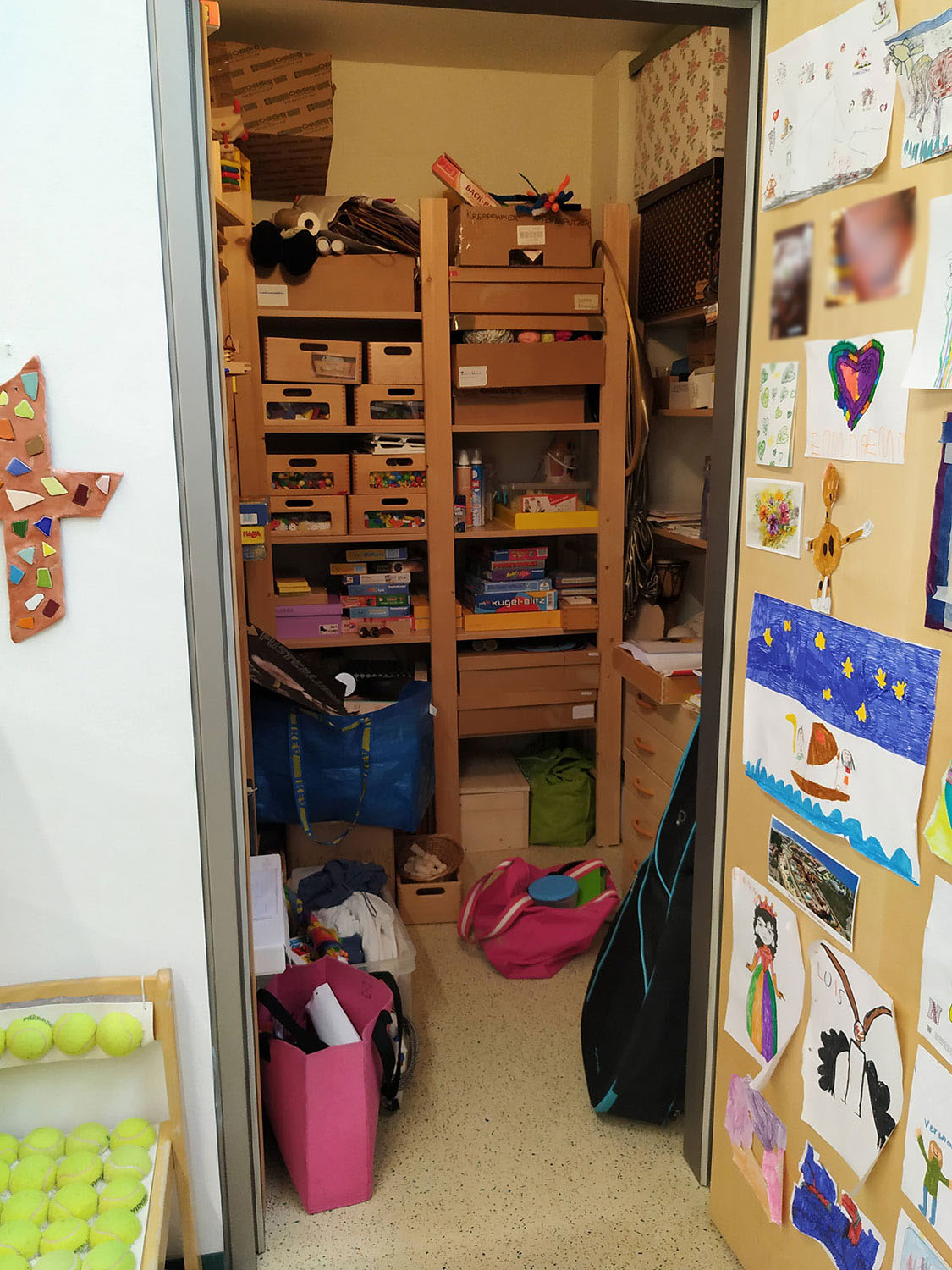

Supplement: Supplementary Data Sheet S5 — Photographic record of building tour. [file Data_Sheet_5.zip › homebase areas/group-room-storage_1.jpg]

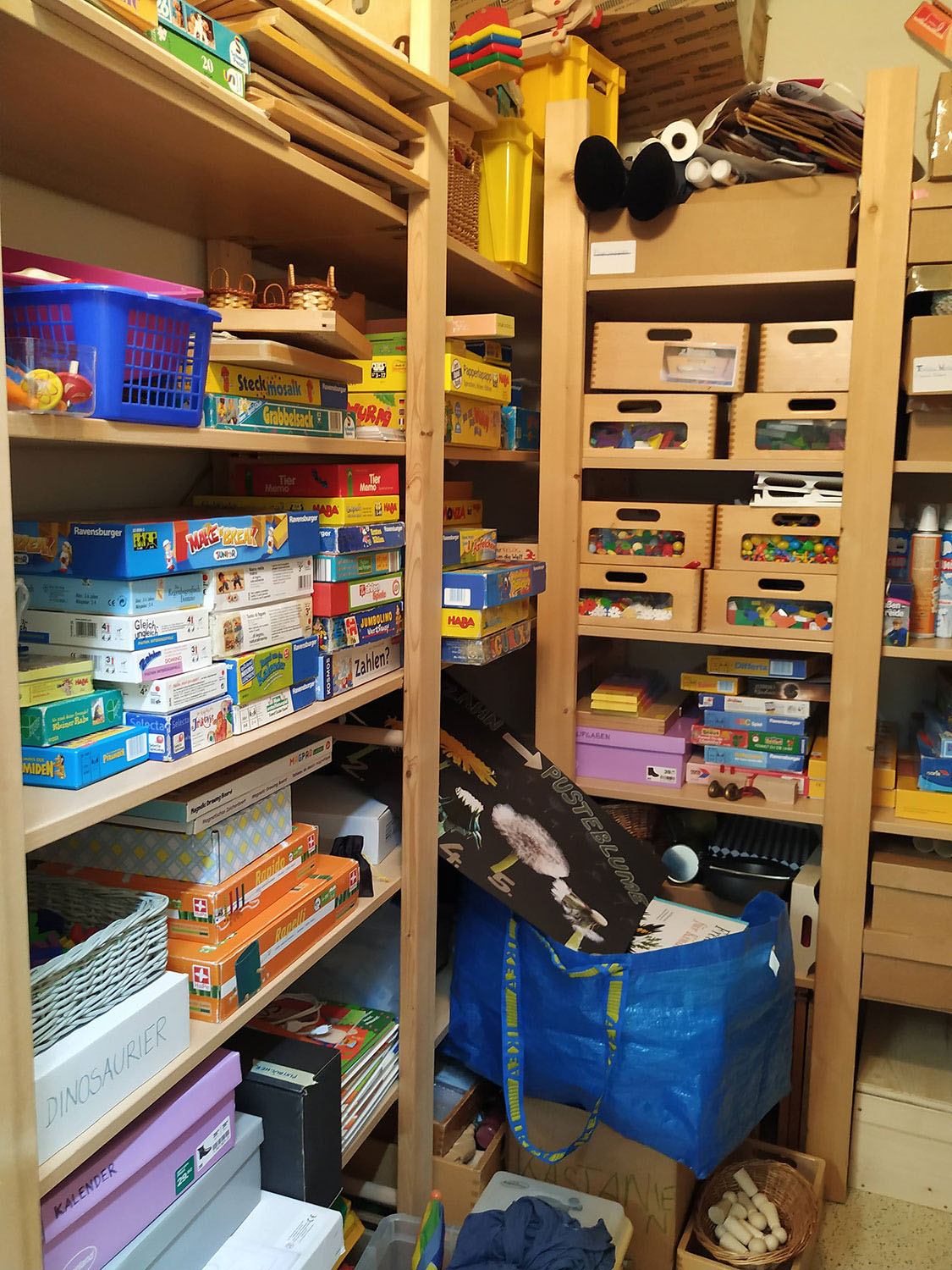

Supplement: Supplementary Data Sheet S5 — Photographic record of building tour. [file Data_Sheet_5.zip › homebase areas/group-room-storage_2.jpg]

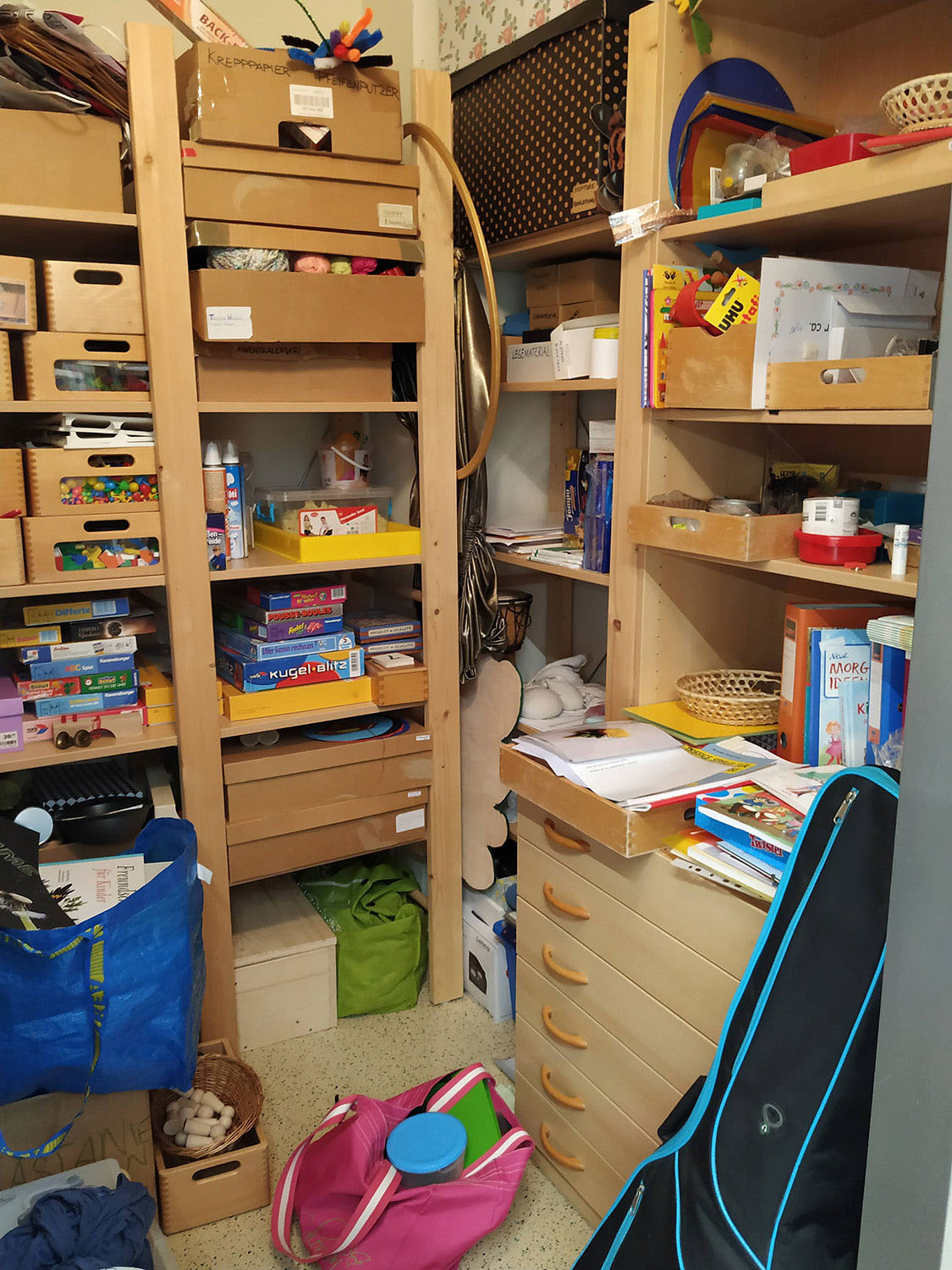

Supplement: Supplementary Data Sheet S5 — Photographic record of building tour. [file Data_Sheet_5.zip › homebase areas/group-room-storage_3.jpg]

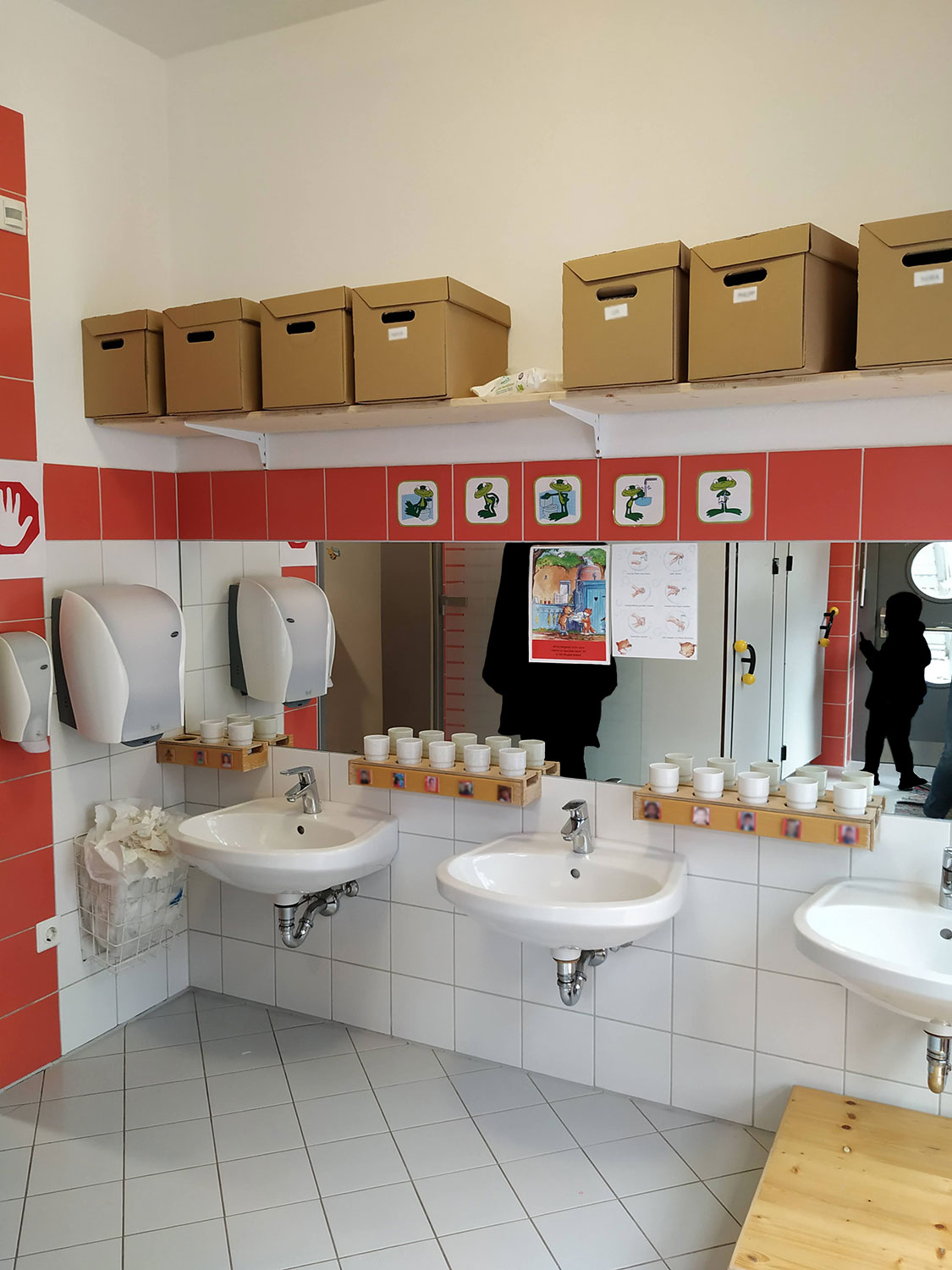

Supplement: Supplementary Data Sheet S5 — Photographic record of building tour. [file Data_Sheet_5.zip › homebase areas/lavatory_1.jpg]

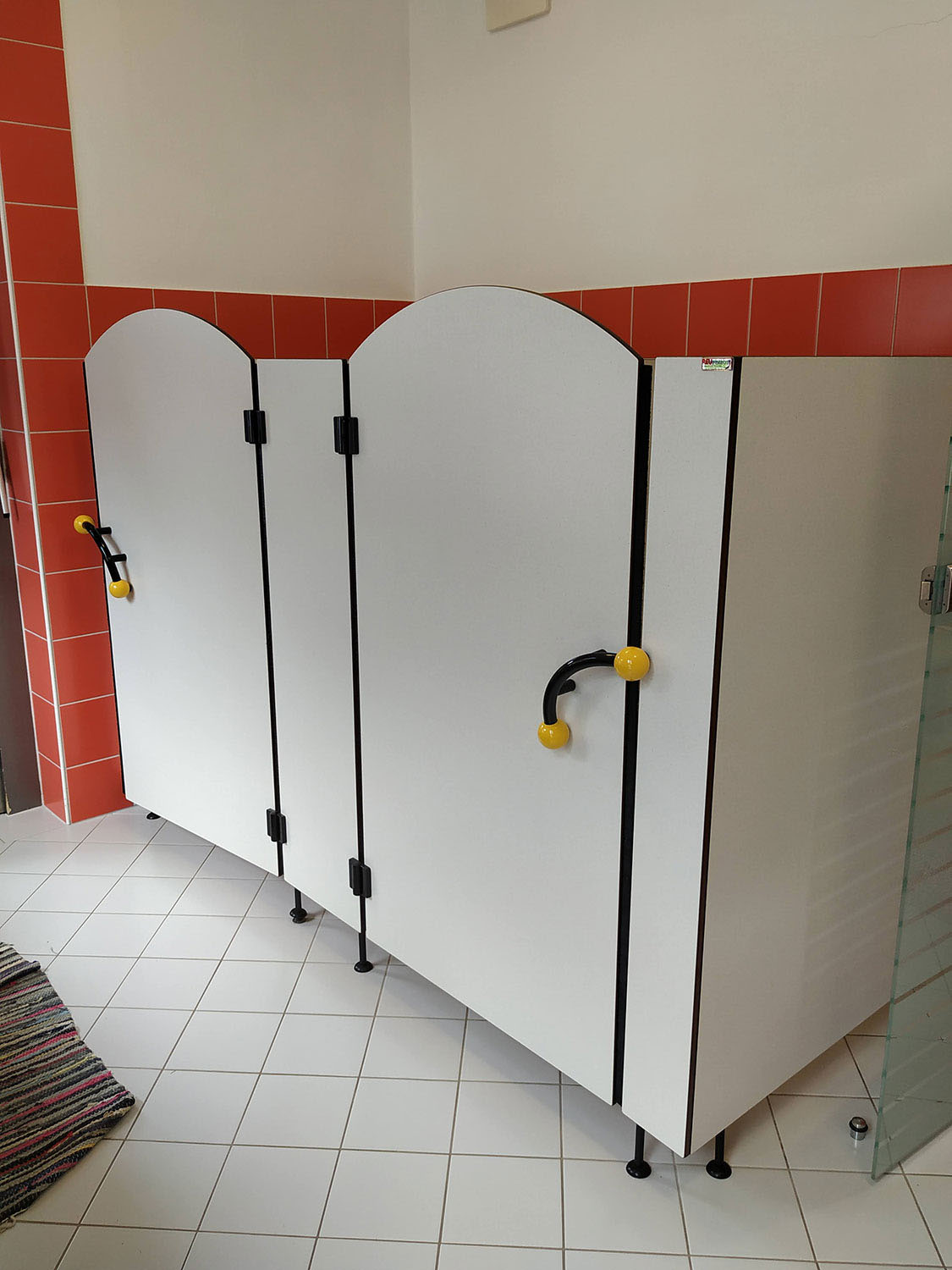

Supplement: Supplementary Data Sheet S5 — Photographic record of building tour. [file Data_Sheet_5.zip › homebase areas/lavatory_2.jpg]

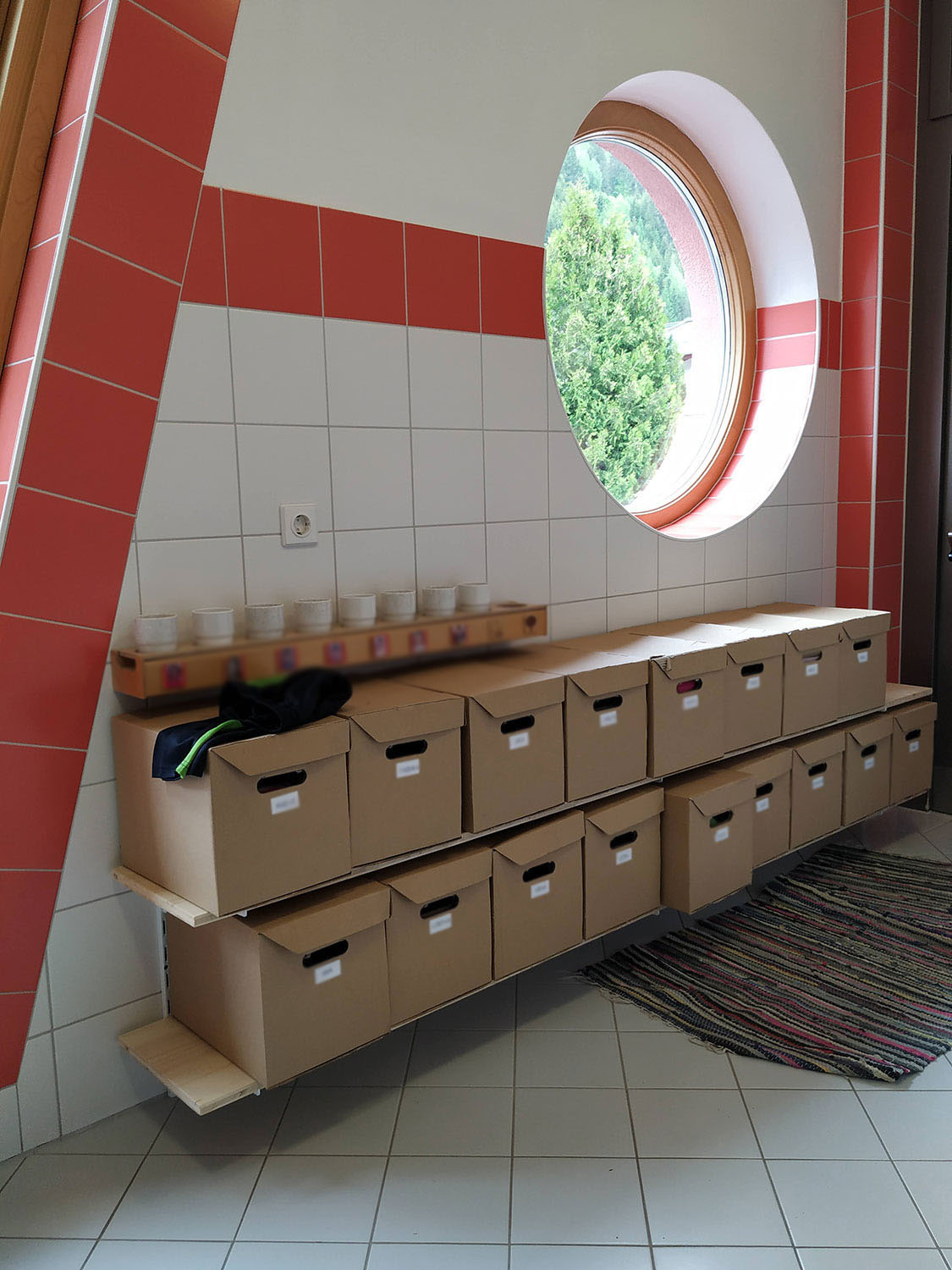

Supplement: Supplementary Data Sheet S5 — Photographic record of building tour. [file Data_Sheet_5.zip › homebase areas/lavatory_3.jpg]

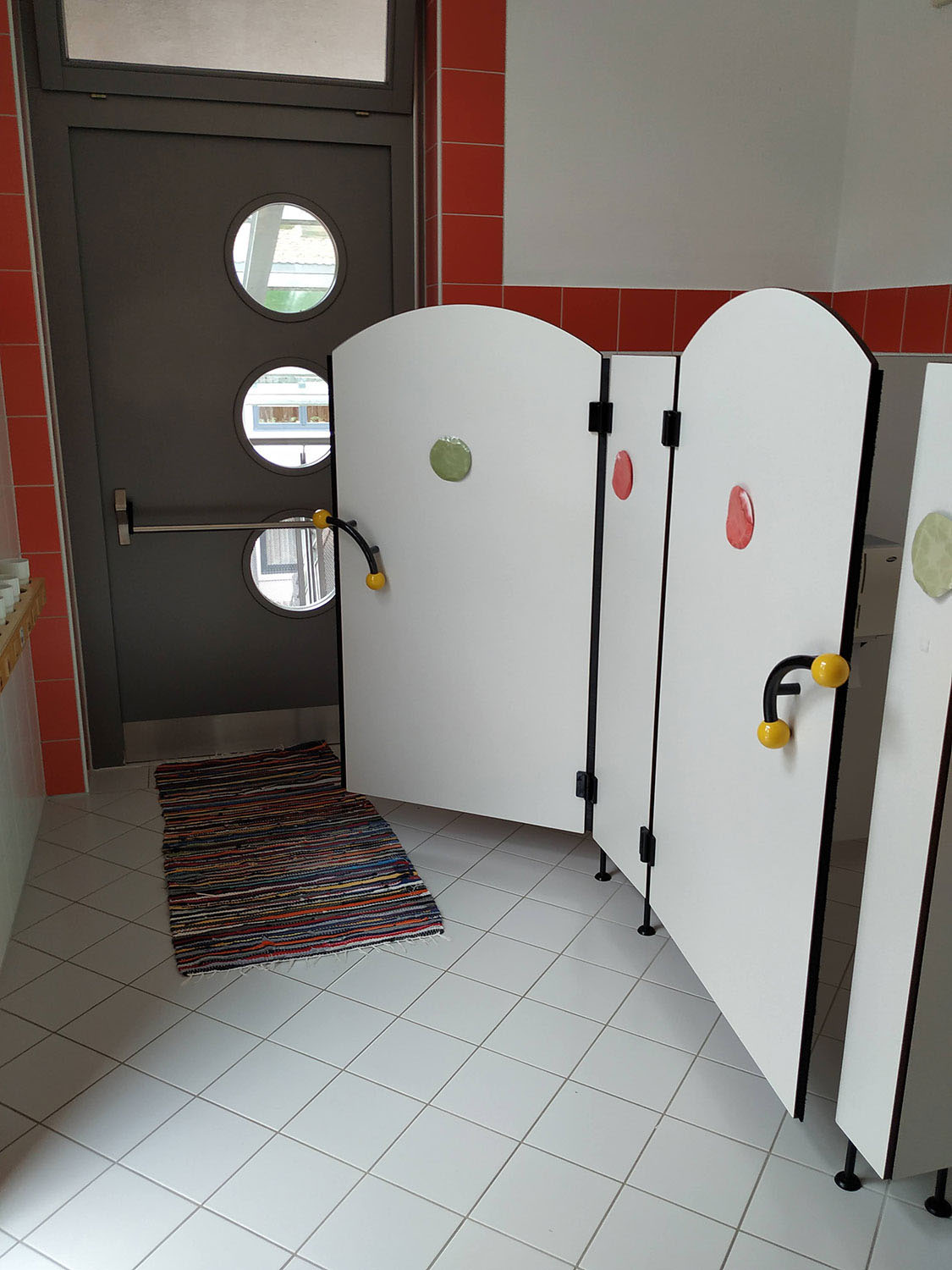

Supplement: Supplementary Data Sheet S5 — Photographic record of building tour. [file Data_Sheet_5.zip › homebase areas/lavatory_4.jpg]

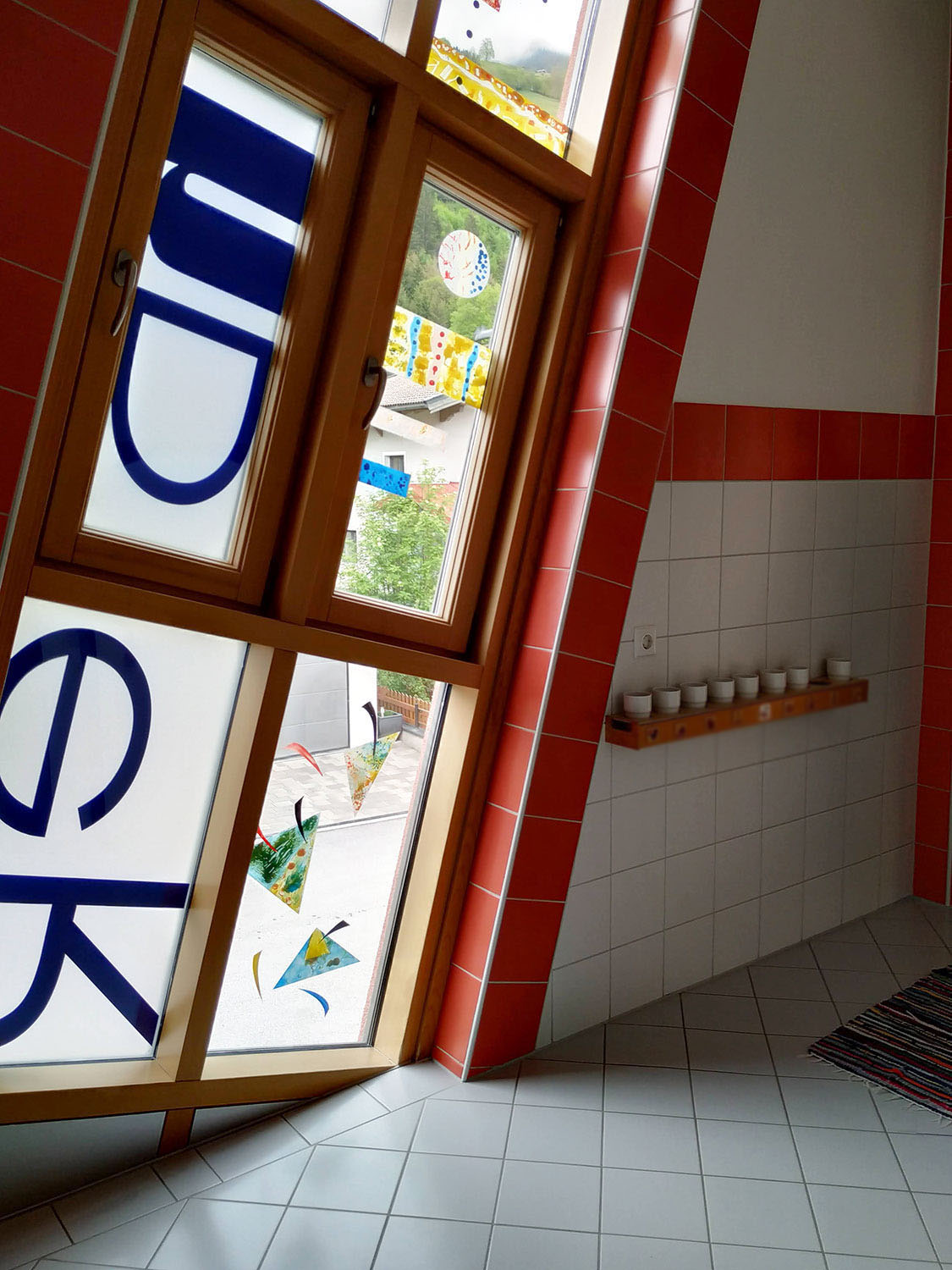

Supplement: Supplementary Data Sheet S5 — Photographic record of building tour. [file Data_Sheet_5.zip › homebase areas/lavatory_5.jpg]

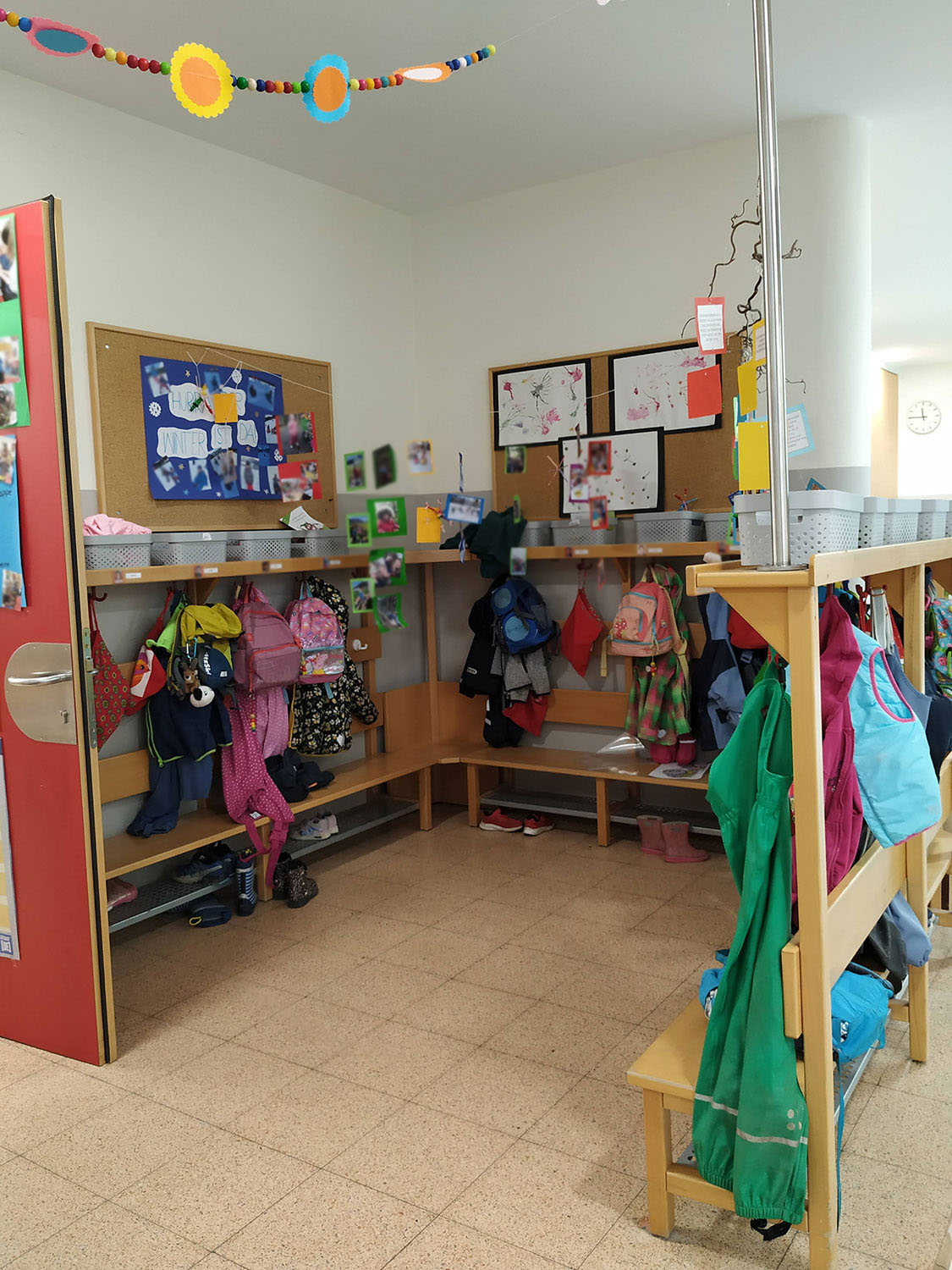

Supplement: Supplementary Data Sheet S5 — Photographic record of building tour. [file Data_Sheet_5.zip › homebase areas/wardrobe_1.jpg]

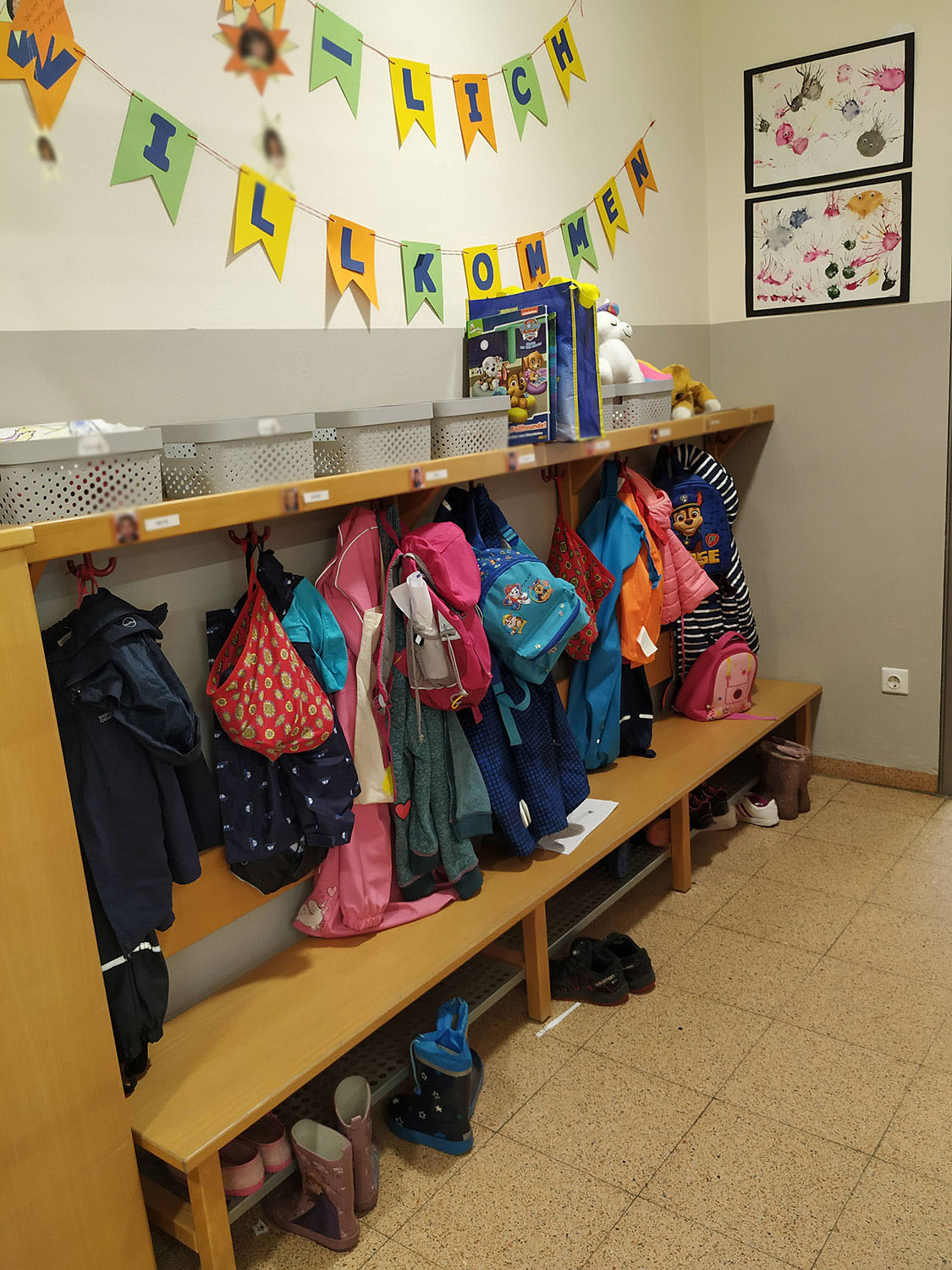

Supplement: Supplementary Data Sheet S5 — Photographic record of building tour. [file Data_Sheet_5.zip › homebase areas/wardrobe_2.jpg]

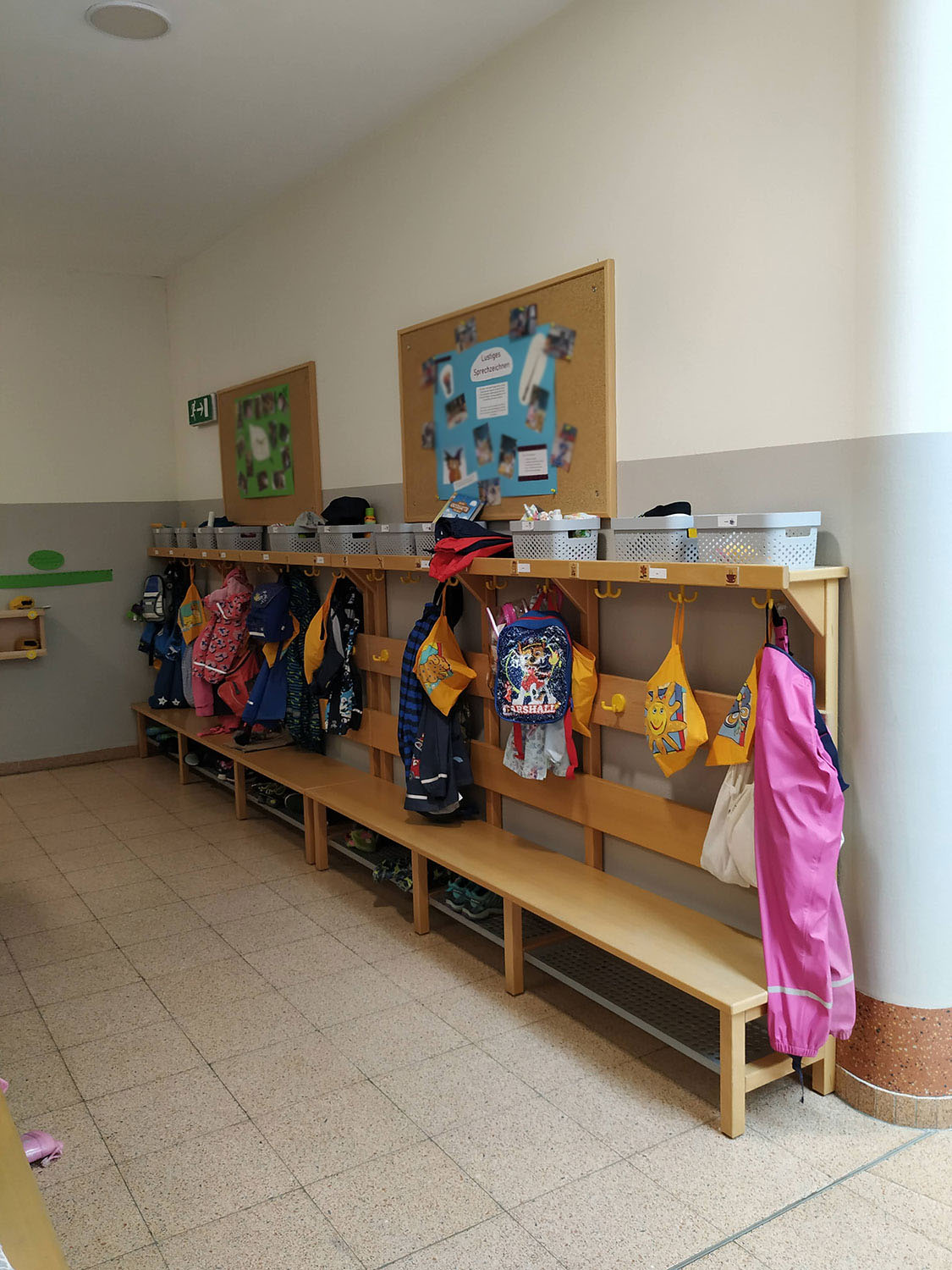

Supplement: Supplementary Data Sheet S5 — Photographic record of building tour. [file Data_Sheet_5.zip › homebase areas/wardrobe_3.jpg]

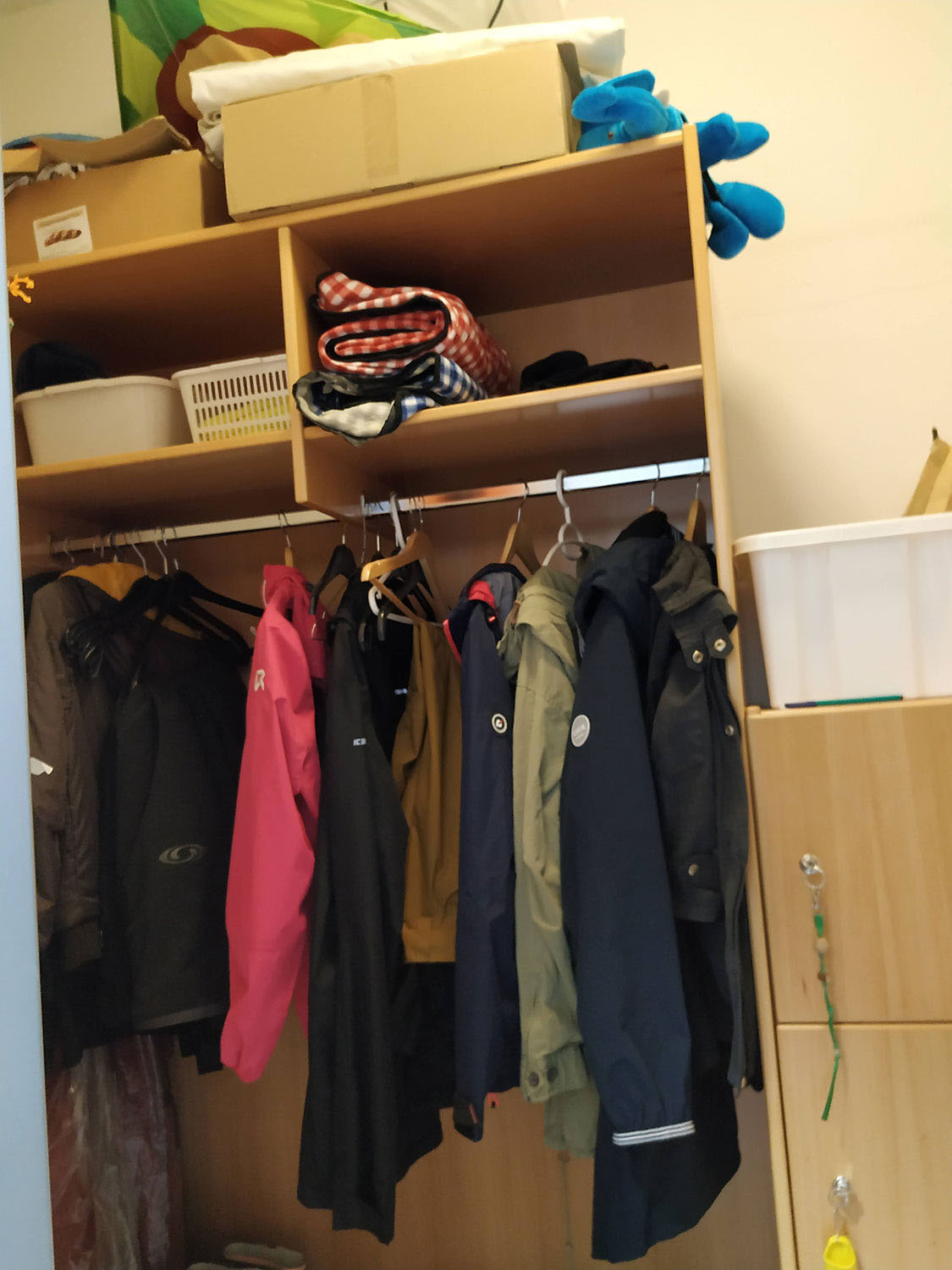

Supplement: Supplementary Data Sheet S5 — Photographic record of building tour. [file Data_Sheet_5.zip › staff areas/staff-cloakroom_1.jpg]

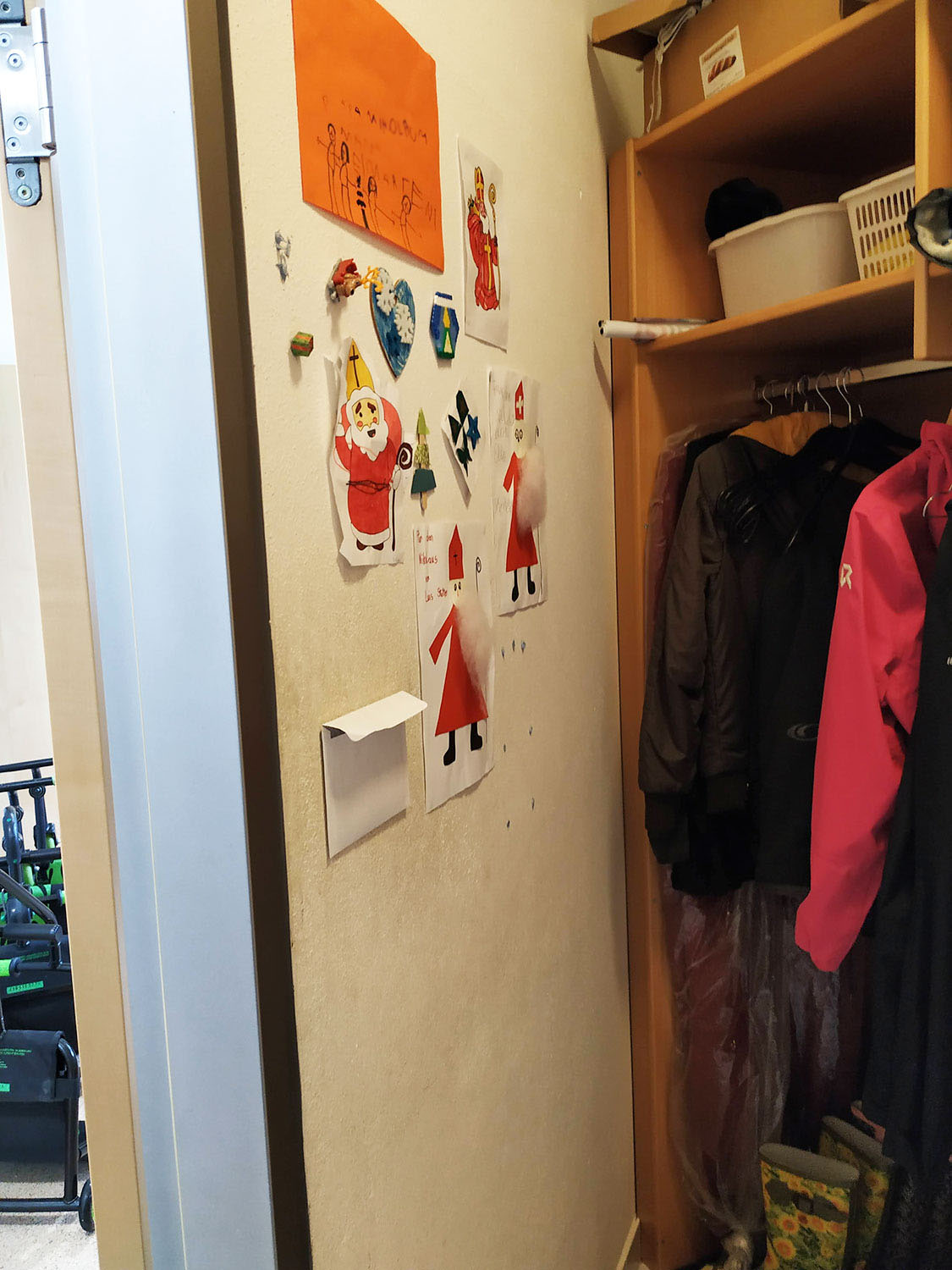

Supplement: Supplementary Data Sheet S5 — Photographic record of building tour. [file Data_Sheet_5.zip › staff areas/staff-cloakroom_2.jpg]

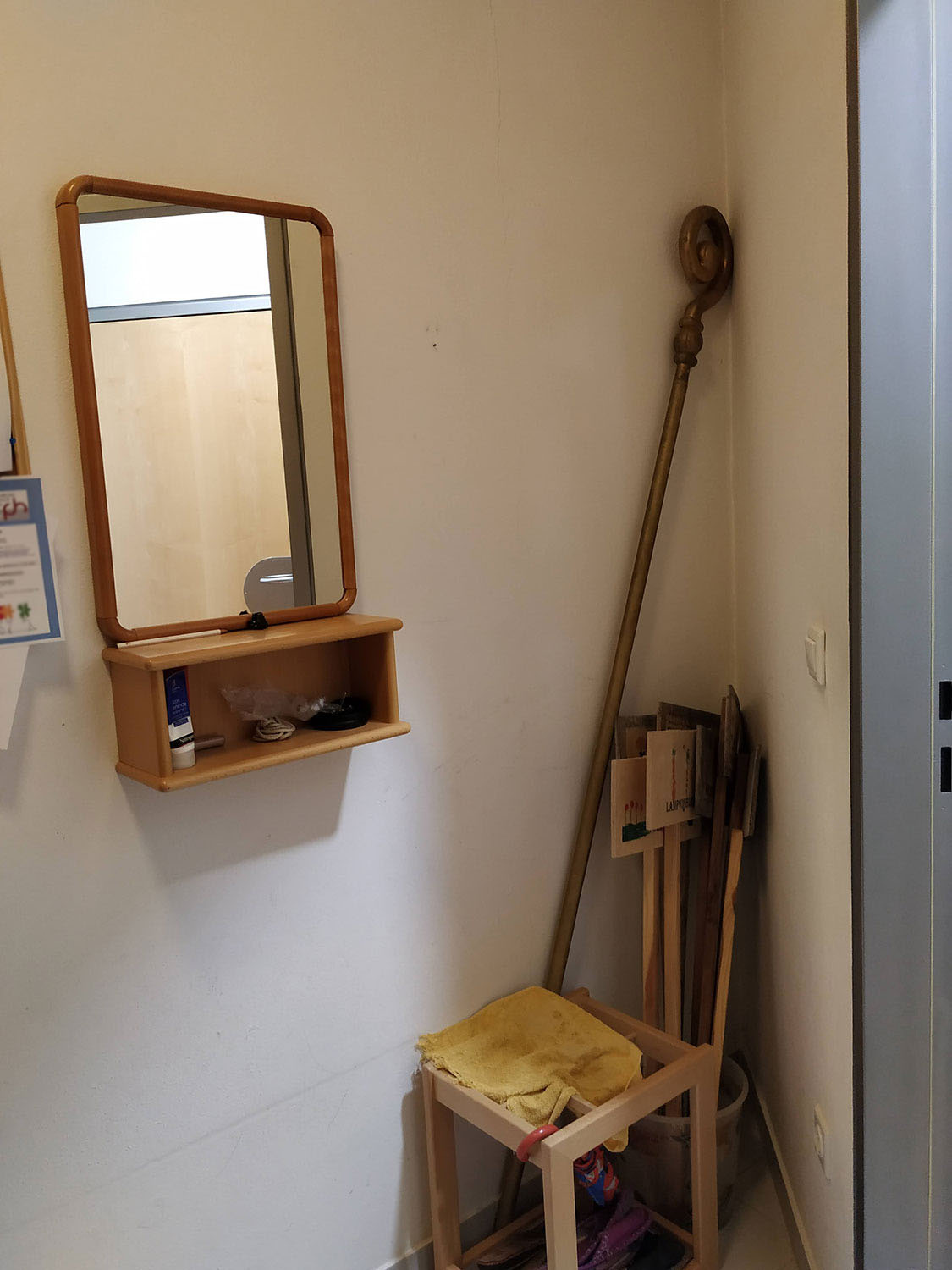

Supplement: Supplementary Data Sheet S5 — Photographic record of building tour. [file Data_Sheet_5.zip › staff areas/staff-cloakroom_3.jpg]

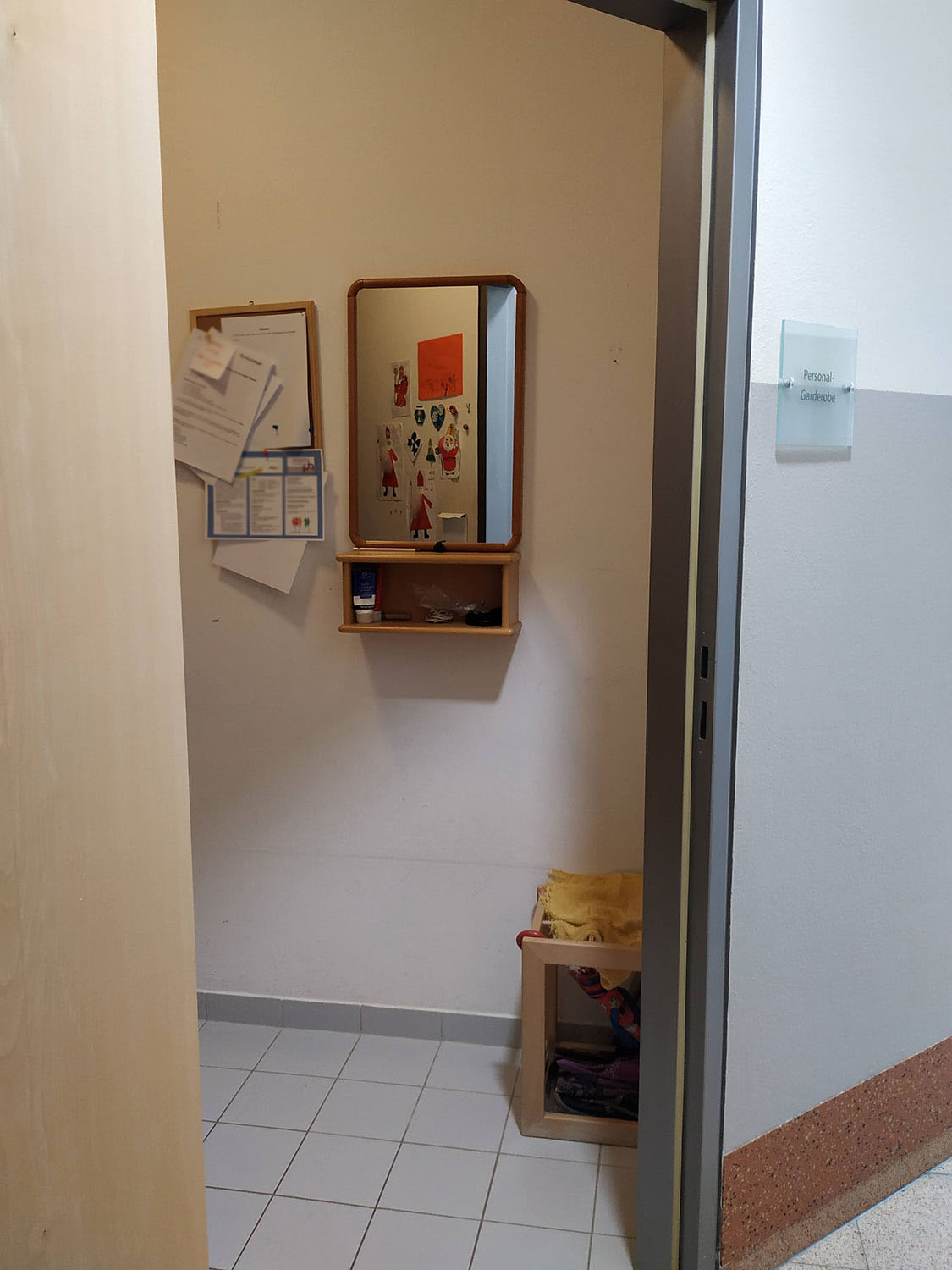

Supplement: Supplementary Data Sheet S5 — Photographic record of building tour. [file Data_Sheet_5.zip › staff areas/staff-cloakroom_4.jpg]

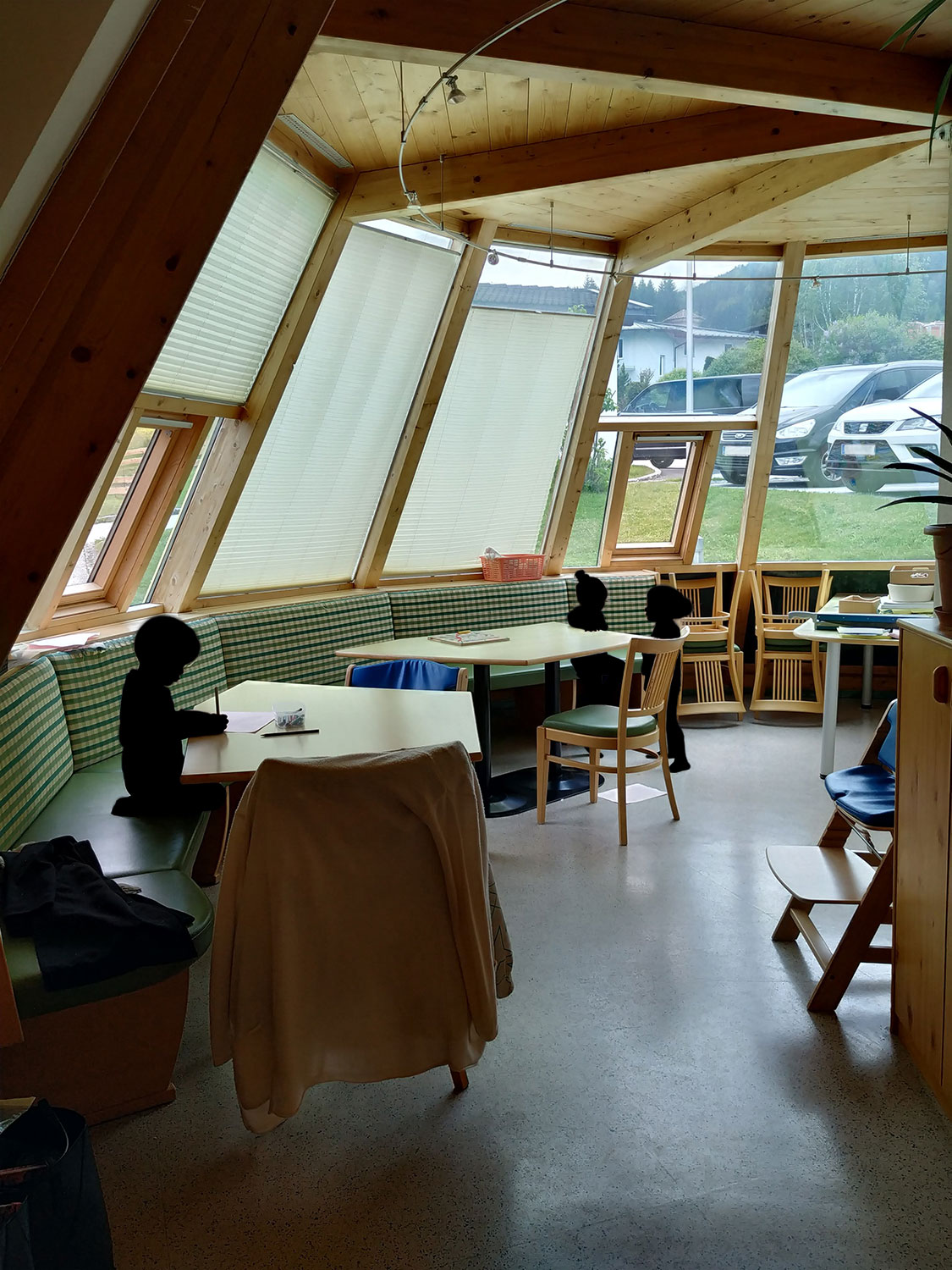

Supplement: Supplementary Data Sheet S5 — Photographic record of building tour. [file Data_Sheet_5.zip › staff areas/staffroom_1.jpg]

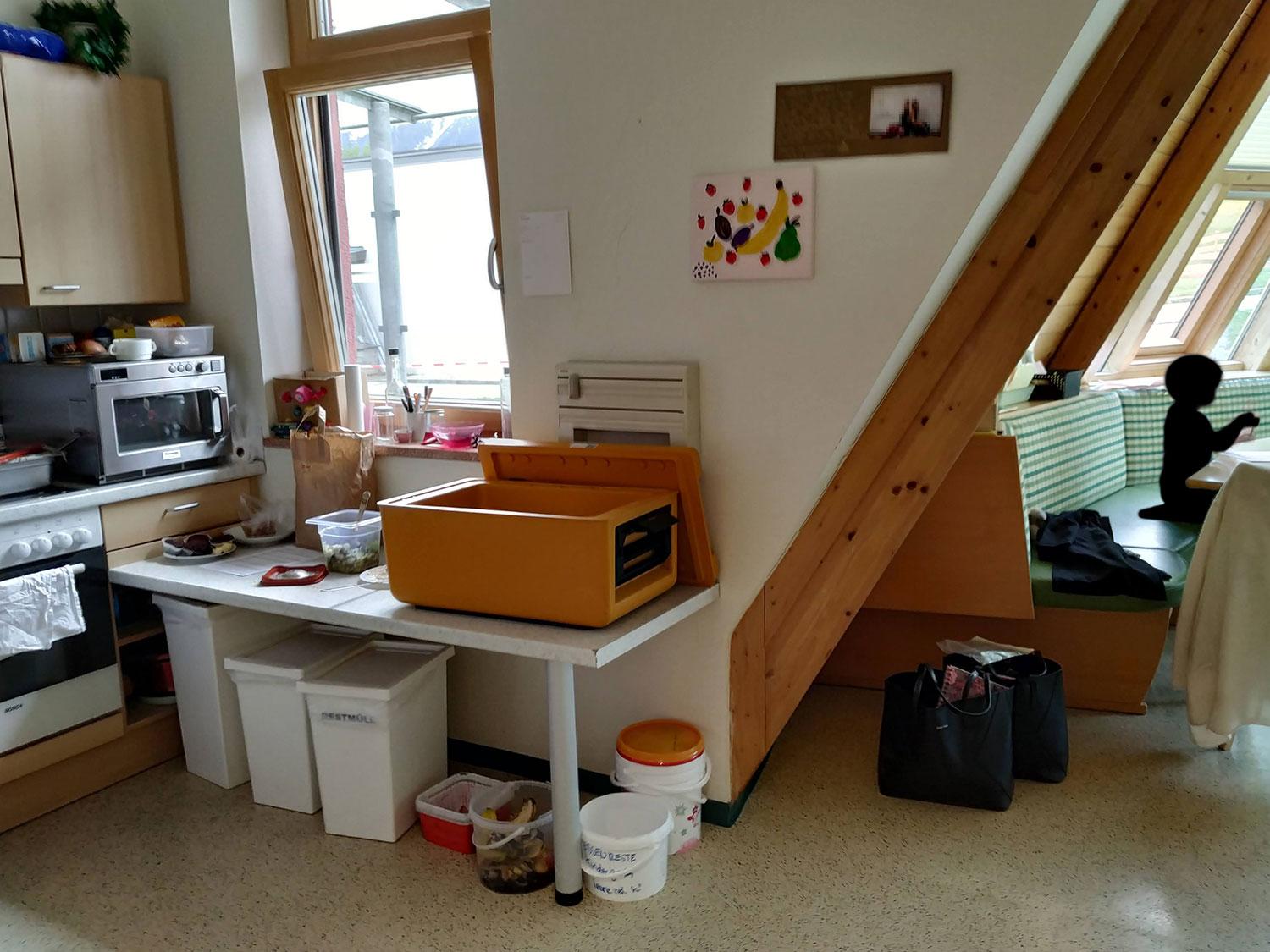

Supplement: Supplementary Data Sheet S5 — Photographic record of building tour. [file Data_Sheet_5.zip › staff areas/staffroom_2_anonym.jpg]

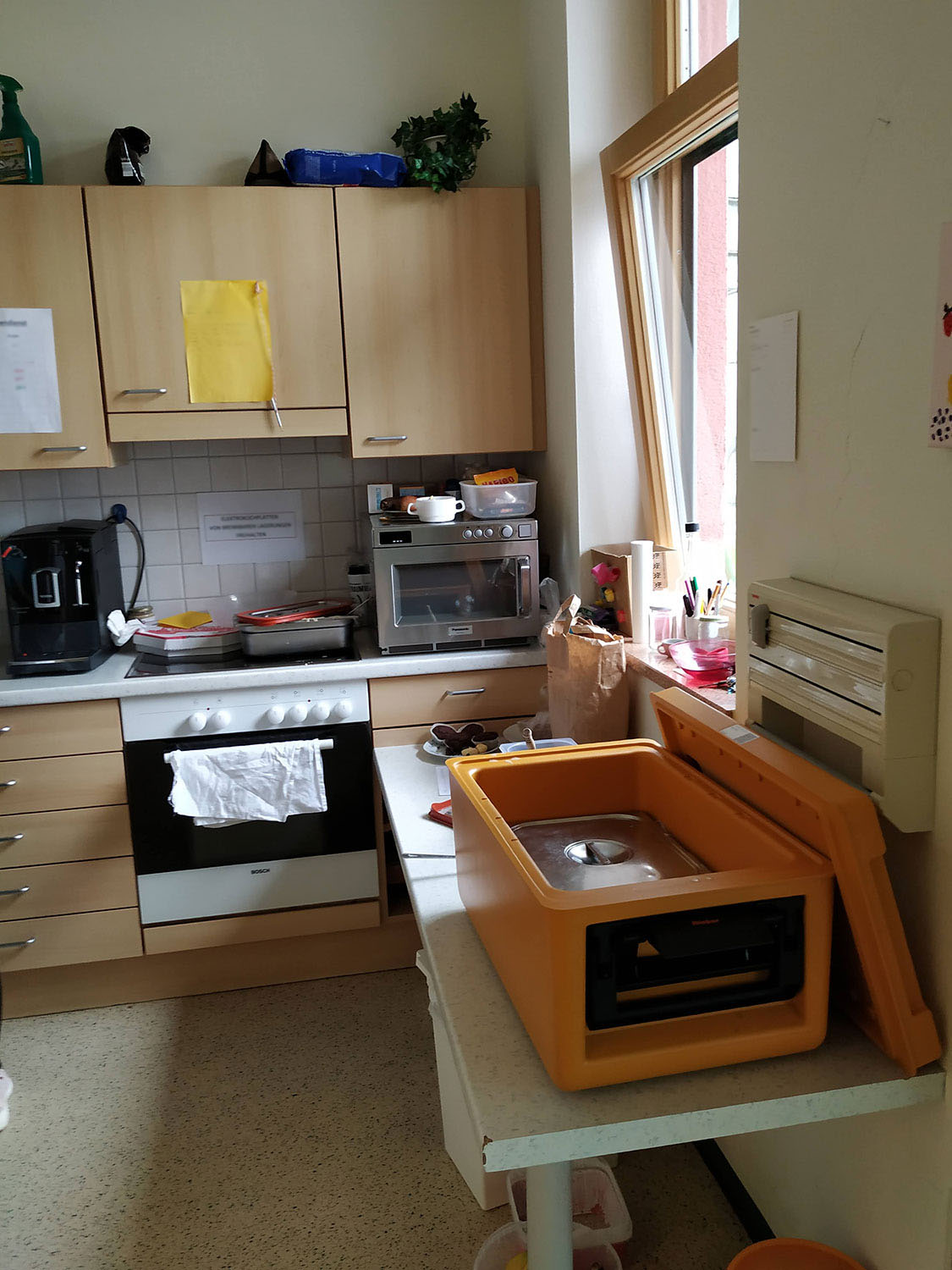

Supplement: Supplementary Data Sheet S5 — Photographic record of building tour. [file Data_Sheet_5.zip › staff areas/staffroom_3.jpg]

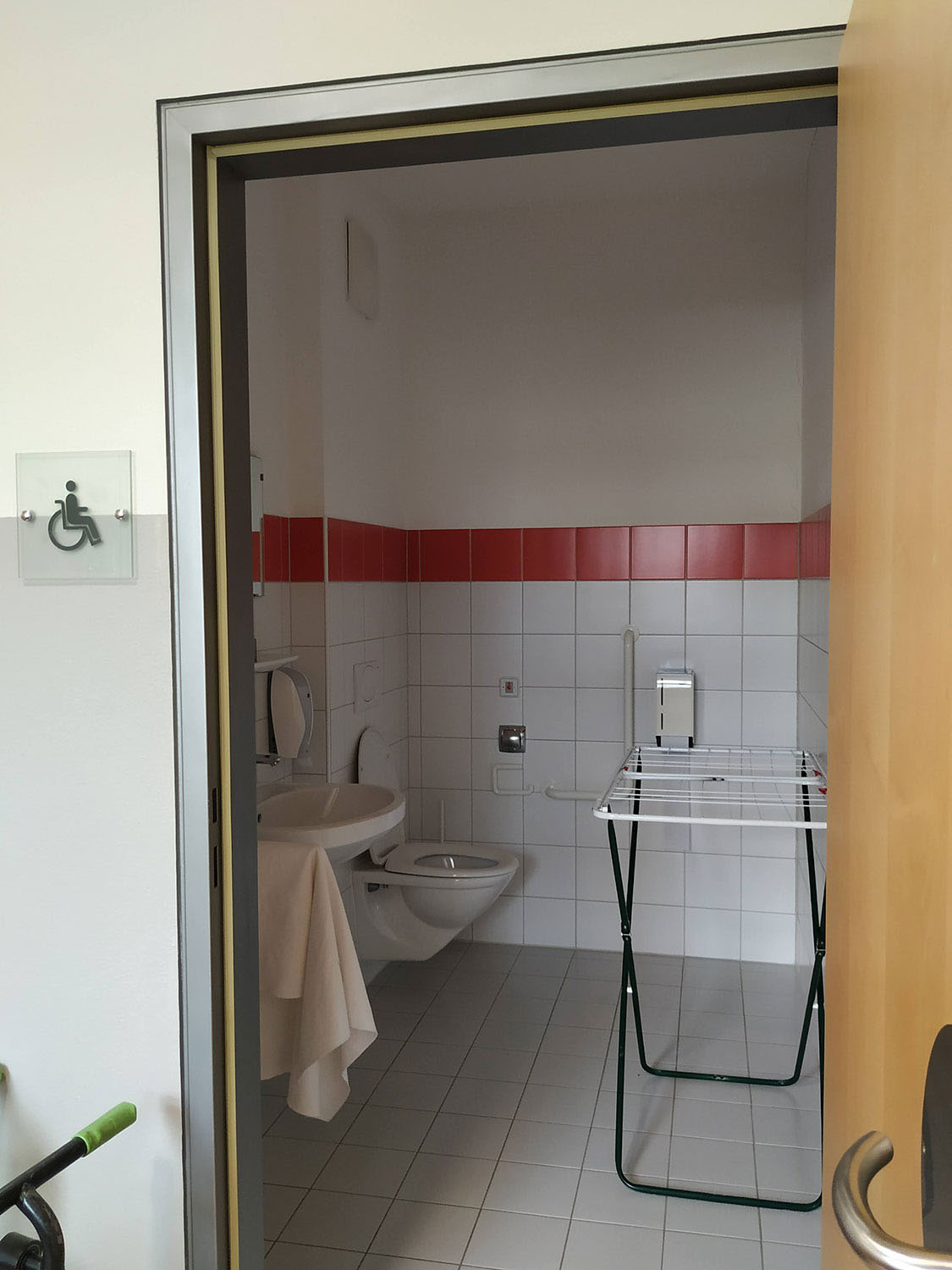

Supplement: Supplementary Data Sheet S5 — Photographic record of building tour. [file Data_Sheet_5.zip › staff areas/staff-toilet_1.jpg]

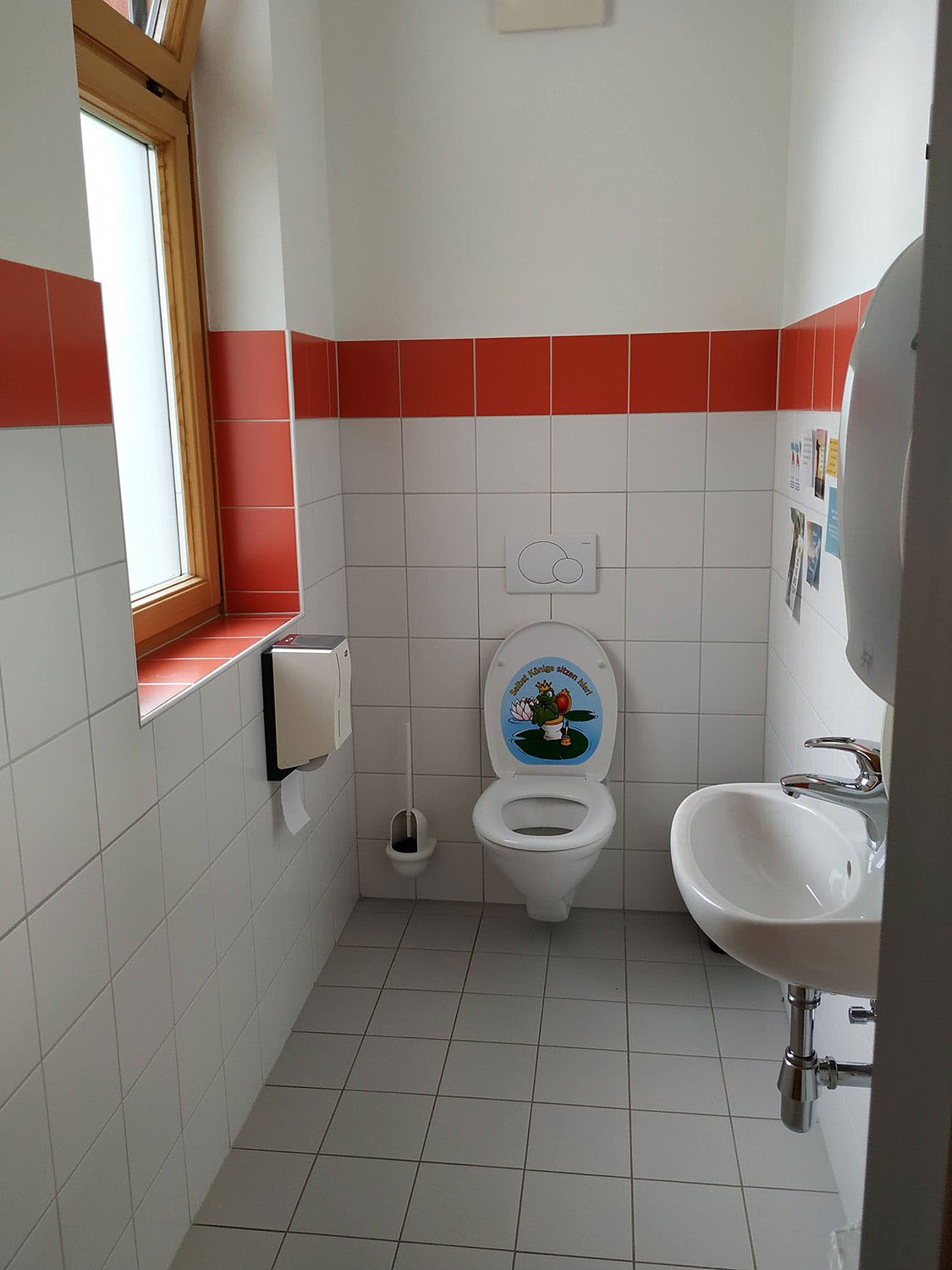

Supplement: Supplementary Data Sheet S5 — Photographic record of building tour. [file Data_Sheet_5.zip › staff areas/staff-toilet_2.jpg]
