# Supplementary material for: Uncovering placemaking needs with(in) a kindergarten community: a cross-disciplinary approach to participatory design
Source: Front Psychol. 2023 Jun 20;14:1126276. doi: 10.3389/fpsyg.2023.1126276 (PMC10319412; doi:10.3389/fpsyg.2023.1126276)
Supplement: Supplementary Data Sheet S6 — Data from building evaluation. [file Data_Sheet_6.pdf]

Data from building evaluation (anonymized)

The dataset contains the ECEC staff's responses that describe spatial qualities of their workplace and associated positive or negative experiences. The building evaluation was conducted in the existing kindergarten building during 3 weeks in May and June 2021.

Interview — The interview with the headmistress of the kindergarten was conducted during a building tour. The interviewee gave the building tour and decided upon the number, type, and sequence of areas visited around and inside the building. A focus was on areas used with the four groups of 3-to-6-year aged children, the target age group of the parallel conducted study arm with children. All homebase areas visited were similarly designed and equipped. The interviewee described one homebase area (blue) in detail and made respective corrections and amendments for the homebase areas of the remaining groups.

Total number of responses (#src): 111

Survey — The survey forms were delivered to the kindergarten and picked up after a period of 3 weeks. Each participant filled one paper-and-pencil form at an individually suitable time point. The participants decided upon the number, type, and sequence of areas they described. All the teaching and caregiving staff actively working in the evaluated building (n= 11) participated. This included two teachers who work with children younger than the target group of the parallel study arm. Thus, the responses include descriptions of additional areas used by a toddler's group (i.e., orange homebase areas, a separate gym and entrance area).

Total number of responses (#src): 309

Note, that the dataset reflects the comprehensive view of the ECEC staff at different occupational levels, responsibilities, and time of work experience in the evaluated building. The information is complementary and not comparable on a quantitative level. The data are available in the original language (German); additionally, a close – if possible verbatim – English translation of the content is provided. For translation of further relevant terms see below.

Translation (German / English)

Activity areas
Garten / garden
Kreativraum / creative area
Rhythmikraum / rhythmics area
Schlafraum / resting area
Turnraum / gym

Homebase areas
Garderobe / wardrobe
Gruppenraum / group room
Waschraum / lavatory

Common areas
Eingangsbereich / entrance area
Essbereich / eating area
Gebäude / building

Staff areas
Büroraum / office room
Personalgarderobe / staff cloakroom
Personalraum / staff room
Personal-WC / staff toilets

Group (color)
blau / blue
gelb / yellow
grün / green
orange / orange
rot / red

Quality
positiv / positive
negativ / negative

source	#src	id	#rid	group	area	quality	#r	original content (german)	#r	content translation (english)
interview	1	P00	1		Eingangsbereich	positiv	1	Dieser Raum wird zu COVID-Zeiten anders genutzt. Eltern und Kinder kommen über die vier Gruppen-Eingangsbereiche über den Garten direkt in den jeweiligen Gruppenraum.	1	This area is used differently during COVID times. Parents and children come directly into the respective group room via the four group entrance areas via the garden.
interview	2	P00	2		Eingangsbereich	positiv	2	Überdachung Eingangstür	2	entrance door canopy
interview	3	P00	3		Eingangsbereich	positiv	3	sehr naher Parkplatz für Mitarbeiterinnen	3	very close parking lots for staff
interview	4	P00	4		Eingangsbereich	negativ	4	Außenbereich: kein Grünbereich	4	outdoor area: no green areas
interview	5	P00	5		Eingangsbereich	negativ	5	Außenbereich: keine Möglichkeit, Fahrräder abzustellen	5	outdoor area: no option for parking bicycles
interview	6	P00	6		Eingangsbereich	negativ	6	Außenbereich: Sicherheitsbedenken, da Durchzugsstraße vor dem KiGa, keine Abtrennung (Kinder können sich nicht vor dem Kindergarten sammeln - zu nahe an der Straße)	6	outdoor area: safety concerns because there is a thoroughfare in front of the kindergarten, no separation (children cannot gather in front of the kindergarten - too close to the street)
interview	7	P00	7		Eingangsbereich	negativ	7	Innenbereich: zu klein für "schuhfreien KiGa" (angestrebtes Ziel!), Schuhe sollen bereits vor Betreten der KiGa-Räumlichkeiten ausgezogen werden	7	indoor area: too small for "shoe-free kindergarten" (pursued goal!), shoes should already be taken off before entering the kindergarten areas
interview	8	P00	8		Eingangsbereich	negativ	8	Innenbereich: künstliches Licht unangenehm - meist ausgeschalten	8	indoor area: artificial lighting unpleasant – mostly switched off
interview	9	P00	9		Eingangsbereich	negativ	9	Innenbereich: Schließsystem der Türe schlecht (Sicherheit für Kinder), wurde bereits offiziell bemängelt und soll ausgetauscht werden	9	indoor area: locking system of the door bad (safety for children), has been already officially critizised and should be replaced
interview	10	P00	10	blau	Garderobe	positiv	10	sehr gute Lage, direkt vor dem jeweiligen Gruppenraum (kann auch als Rückzugsort genutzt werden und trotzdem gut beobachtbar)	10	very good location, directly in front of the respective group room (can also be used as a place of refuge and still be easily observed)
interview	11	P00	11	blau	Garderobe	positiv	11	viel Platz zum Gestalten für MA und Kinder	11	plenty of space for employees and children to create
interview	12	P00	12	blau	Garderobe	positiv	12	eigener Platz für jedes Kind (hier liegen auch persönliche Dinge, z.B. das Kuscheltier)	12	own place for each child (here are also personal things, e.g. the cuddly toy)
interview	13	P00	13	blau	Garderobe	negativ	13	schlechte Lichtverhältnisse, zu wenig Licht	13	poor lighting conditions, too little light
interview	14	P00	14	blau	Garderobe	negativ	14	Zu wenig Platz, zu eng, es können nicht alle Kinder sitzen bleiben, wenn angezogen (das wäre wünschenswert, erleichtert die Arbeit der MA).	14	Too little space, too narrow, not all children can remain seated when dressed (that would be desirable, facilitates the work for employees).
interview	15	P00	15	blau	Garderobe	negativ	15	schlechte akustische Bedingungen, hoher Geräuschpegel	15	bad acoustic conditions, high noise level
interview	16	P00	16	blau	Garderobe	negativ	16	schlechte Luftqualität, Garderoben können schwer durchgelüftet werden	16	bad air quality, wardrobes are difficult to ventilate
interview	17	P00	17	blau	Garderobe	negativ	17	viel zu wenig Platz für Stauraum	17	far too little space for storage
interview	18	P00	18	blau	Waschraum	positiv	18	Jedes Kind hat einen eigenen Becher an den Waschbecken im Waschraum; es ist ausreichend Platz und Abstellfläche vorhanden.	18	Each child has an own mug at the sinks in the lavatory, there is sufficient space and storage area.
interview	19	P00	19	blau	Waschraum	positiv	19	In jedem Waschraum gibt es - für jede Gruppe separat - einen Ausgang zum Außenbereich / Spielbereich (dieser wird zu COVID-Zeiten auch als Eingang genutzt, wenn Eltern ihre Kinder bringen und abholen, um Ansammlungen zu vermeiden).	19	In each lavatory there is - for each group separately - an exit to the outdoor area / play area (this is also used as an entrance during COVID times when parents bring and pick up their children to avoid crowds).
interview	20	P00	20	blau	Waschraum	negativ	20	Zu wenige Toiletten, bei mittlerweile insgesamt 100 Kindern entstehen Staus im Waschraum, hierbei entstehen	20	Too few toilets, with a total of 100 children there are jams in the lavatories, which creates stressful situations.
interview	21	P00	21	blau	Gruppenraum	positiv	21	große Fensterfront, viel natürliches Licht, Blick auf Grünbereich	21	big window front, much natural light, view of green area
interview	22	P00	22	blau	Gruppenraum	positiv	22	Beschattungsmöglichkeiten teilweise vorhanden, aber nicht	22	shading options partially available, but not enough
interview	23	P00	23	blau	Gruppenraum	positiv	23	Möblierung flexibel verstellbar, es gibt nur ein fix verbautes Element und der Raum ist gut zu gestalten - jedes Jahr werden die Gruppenräume neugestaltet und MA sowie Kinder freuen sich sehr darauf (Stichwort: Abwechslung).	23	Furniture flexibly adjustable, there is only one permanently installed element, and the room is easy to design - every year the group rooms are redesigned and both employees and children look forward to it (keyword: variety).
interview	24	P00	24	blau	Gruppenraum	positiv	24	Jeder Gruppenraum hat einen abgetrennten Abstellraum mit ausreichend Platz.	24	Each group room has a separate storage room with sufficient space.
interview	25	P00	25	blau	Gruppenraum	positiv	25	natürliches Baumaterial (Holz) dominierend	25	natural building materials (wood) dominating
interview	26	P00	26	blau	Gruppenraum	positiv	26	Großer Teppichkreis (Gruppenfarbe) in der Mitte des Raumes als visuelles, sozial-verbindendes Element - hier werden Gruppenspiele/-übungen durchgeführt.	26	Large circular carpet (group’s colour) in the middle of the room as visual, socially connecting element - here occur group play / exercises.

source	#src	id	#rid	group	area	quality	#r	original content (german)	#r	content translation (english)
interview	27	P00	27	blau	Gruppenraum	positiv	27	Jeder Gruppenraum hat Tischgruppen-Elemente zur Verfügung (Stichwort: sozial-verbindende Raumelemente) - die MA entscheiden selbst, ob und wie viele Tische sie brauchen, einsetzen wollen - oberste Priorität: Flexibilität und Gestaltungsraum.	27	Each group room has table group elements available (keyword: socially connecting spatial elements); the staff decides for themselves, whether and how many tables they need or want to use - top priority: flexibility and space for design.
interview	28	P00	28	blau	Gruppenraum	positiv	28	Alle Gruppenräume bieten ausreichend gestalterische Möglichkeiten.	28	All group rooms provide sufficient design options.
interview	29	P00	29	blau	Gruppenraum	positiv	29	Offene Regalsysteme in kindgerechter Höhe sind kreativitätsfördernde Raumelemente - sie laden die Kinder zu Spiel und Kreativität ein (Stichwort: Präsentation).	29	Open shelving systems at child-appropriate height are room elements that promote creativity – they invite children to play and to be creative (keyword: presentation).
interview	30	P00	30	blau	Gruppenraum	positiv	30	Die Gruppenräume sind abgeschlossene Bereiche (geschlossene Räume), jede Gruppe kann ungestört sein.	30	Group rooms are enclosed areas (closed rooms), each group can be undisturbed.
interview	31	P00	31	blau	Gruppenraum	positiv	31	Jeder Gruppenraum bietet einen Rückzugsort für Kinder (die Räume Blau und Grün haben ein Raumgerüst - diese sind besonders beliebt; die Räume Gelb und Rot haben am Boden Refugien / Zelte gebaut - teilweise provisorisch); die Kinder fühlen sich am Rückzugsort versteckt, die Rückzugsorte sind aber dennoch gut einsehbar für die MA	31	Each group room provides a refuge for children (the Blue and Green rooms have a scaffolding – these are particularly popular; the Yellow and Red rooms have refuges / tents built on the ground - partly temporary); the children feel hidden in the place of refuge, but the places of refuge are still clearly visible for the employees.
interview	32	P00	32	blau	Gruppenraum	negativ	32	schlechte Akustik (hallende Räume), hoher Geräuschpegel, auch schlechte akustische Abschirmung von außen (andere Gruppen	32	bad acoustics (reverbarating rooms), high noise level, also poor acoustic shielding from outside (other groups are audible)
interview	33	P00	33	blau	Gruppenraum	negativ	33	Temperaturregulierung schwierig (Fensterfront) - im Winter viel zu kalt, im Sommer teilweise zu warm	33	temperature regulation difficult (window front) - much too cold in winter, sometimes too warm in summer
interview	34	P00	34	blau	Gruppenraum	negativ	34	Es ist nicht möglich, die Gruppenräume vollständig zu beschatten - dies wäre nicht nur zur besseren Temperaturregulierung, sondern auch für bestimmte Spiele/Übungen und für Ruhephasen wünschenswert.	34	It is not possible to completely shade the group rooms - this would be desirable not only for better temperature regulation but also for certain games / exercises and for resting periods.
interview	35	P00	35	blau	Gruppenraum	negativ	35	Essentieller Unterschied: Die Raumgerüsthöhe ist etwas zu niedrig - die MA stoßen sich häufig den Kopf.	35	Essential difference: The height of the scaffolding is a little bit too low – the staff often bump their heads.
interview	36	P00	36	grün	Gruppenraum	negativ	36	Essentieller Unterschied: extremere saisonale Temperaturempfindung (zu heiß im Sommer, zu kalt im Winter)	36	Essential difference: more extreme seasonal temperature perception (too hot in summer, too cold in winter)
interview	37	P00	37	grün	Gruppenraum	negativ	37	Essentieller Unterschied: weniger Grünbereich sichtbar durch die Fensterfront	37	Essential difference: less green area visible through the window front
interview	38	P00	38	grün	Gruppenraum	negativ	38	Essentieller Unterschied: Die Raumgerüsthöhe ist etwas zu niedrig - die MA stoßen sich häufig den Kopf.	38	Essential difference: The height of the scaffolding is a little bit too low – the staff often bump their heads.
interview	39	P00	39	rot	Gruppenraum	negativ	39	Essentieller Unterschied: weniger natürliches Baumaterial / Möblierung als in den anderen Gruppenräumen	39	Essential difference: less natural building materials / furnishings than in the other group rooms
interview	40	P00	40	rot	Gruppenraum	positiv	40	Essentieller Unterschied: kein Raumgerüst - daher kein Problem mit dessen Höhe; der Rückzugsort für Kinder ist ein einladend gestaltetes Zelt am Boden	40	Essential difference: no scaffolding - therefore no problem with its height; the refuge for children is an invitingly designed tent on the ground
interview	41	P00	41	rot	Gruppenraum	negativ	41	Essentielle Unterschiede: extremere saisonale Temperaturempfindung (zu heiß im Sommer, zu kalt im Winter)	41	Essential difference: more extreme seasonal temperature perception (too hot in summer, too cold in winter)
interview	42	P00	42	gelb	Gruppenraum	negativ	42	Essentieller Unterschied: weniger natürliches Baumaterial (Holz) als in anderen Gruppenräumen	42	Essential difference: less natural building materials than in the other group rooms
interview	43	P00	43	gelb	Gruppenraum	positiv	43	Essentieller Unterschied: In diesem Raum befindet sich eine Zimmerpflanze und außerdem Tiere (ein Terrarium mit Insekten und Pflanzen) - diese Elemente werden besonders von den MA geschätzt - den Kindern gefallen die Tiere besonders.	43	Essential difference: In this room there is an indoor plant and also animals (a terrarium with insects and plants) – these elements are particularly appreciated by the employees – the children especially like the animals.
interview	44	P00	44	gelb	Gruppenraum	negativ	44	Essentieller Unterschied: schlechterer Rückzugsort für Kinder (eine Wand eines ehemaligen Schlafraumes wurde entfernt, die verbliebene Nische wurde notgedrungen umfunktioniert)	44	Essential difference: less appropriate refuge for children (a wall from a former sleep area was removed, the resulting niche was converted out of necessity)
interview	45	P00	45	gelb	Gruppenraum	positiv	45	Essentieller Unterschied: Kein Raumgerüst vorhanden - daher kein Problem mit dessen Höhe	45	Essential difference: there is no scaffolding - therefore no problem with its height
interview	46	P00	46		Rhythmikraum	positiv	46	"Multifunktionsraum" im EG	46	"multifunctional room" on the ground floor

source	#src	id	#rid	group	area	quality	#r	original content (german)	#r	content translation (english)
interview	47	P00	47		Rhythmikraum	positiv	47	Dieser Raum wird nicht nur als klassischer Rhythmikraum benutzt, sondern auch für Tanz, Bewegung und steht den Kindern zur freien Benutzung zur Verfügung.	47	This room is not only used as a classic rhythm room, but also for dance and movement and is available to the children for open use.
interview	48	P00	48		Rhythmikraum	positiv	48	Blick auf Grünflächen im Garten	48	view of green areas in the garden
interview	49	P00	49		Rhythmikraum	positiv	49	Große Kreismarkierung in der Mitte des Raumes - optisches Merkmal für den Sammelplatz; die Größe des Kreises ist ausreichend für 25 Kinder und ist ein wichtiges sozial-verbindendes [illegible]	49	Large circular marking in the middle of the room - optical feature for the assembly point; the size of the circle is sufficient for 25 children and it is an important socially connecting spatial element.
interview	50	P00	50		Rhythmikraum	negativ	50	Schlechte Lichtverhältnisse trotz großer Fensterfront - die mittleren Fenster in der oberen Reihe können nicht verdunkelt werden. Die Möglichkeit, den Raum vollständig abzudunkeln wäre sehr wünschenswert für bestimmte Spiele und Übungen (z.B. Wahrnehmung des Gehörsinns bei Dunkelheit).	50	Poor lighting conditions despite the large window front; the middle windows in the upper row cannot be shaded. The possibility to completely darken the room would be very desirable for certain games and exercises (e.g., auditory perception in the dark).
interview	51	P00	51		Rhythmikraum	negativ	51	Schlechte akustische Bedingungen - Geräusche von außen (Gang und angrenzende Gruppenräume) sind insbesondere bei dieser Raumfunktion sehr störend. Man kann keine leisen Übungen machen.	51	Poor acoustic conditions - noises from outside (corridor and adjoining group rooms) are very disturbing, especially with this room function. We cannot do silent exercises.
interview	52	P00	52		Rhythmikraum	negativ	52	zu wenig Stauraum für Instrumente und Spiele	52	too little storage space for instruments and games
interview	53	P00	53		Rhythmikraum	negativ	53	Schlechte Präsentation von Instrumenten und Spielen - die Materialien sollten für die Kinder sichtbar sein und sie zum Spiel und zum Ausprobieren einladen.	53	Poor presentation of instruments and games - the materials should be visible for the children and invite them to play and try out.
interview	54	P00	54		Turnraum	positiv	54	Es ist ein separater Abstellraum (abgetrennter Bereich) mit Stauraum vorhanden.	54	There is a separate storage room (separated area) with storage space.
interview	55	P00	55		Turnraum	negativ	55	[Wunsch] mehr Stauraum ist allerdings wünschenswert	55	[request] more storage space is desirable, however
interview	56	P00	56		Turnraum	positiv	56	Der Turnraum bietet ausreichend Platz für Spiel und Bewegung.	56	The gym offers enough space for play and movement.
interview	57	P00	57		Turnraum	positiv	57	Keine bis minimale Möblierung, ermöglicht freies Spiel.	57	Little to no furniture allows free play.
interview	58	P00	58		Turnraum	positiv	58	großer Kreis am Boden in der Mitte des Raumes - sozial-verbindendes Raumelement	58	big circle on the floor in the middle of the room – socially connecting spatial element
interview	59	P00	59		Turnraum	positiv	59	Bodenheizung - der Raum kann auch barfuß genutzt werden.	59	Floor heating - the room can also be used barefoot.
interview	60	P00	60		Turnraum	negativ	60	Beschattung bei großer Fensterfront nicht vollständig möglich - es wäre wünschenswert, wenn der Raum vollständig abgedunkelt werden kann für bestimmte Übungen.	60	Shading is not completely possible with the large window front - it would be desirable if the room could be completely darkened for certain exercises.
interview	61	P00	61		Turnraum	negativ	61	schlechte akustische Bedingungen, sehr hoher Geräuschpegel	61	bad acoustic conditions, very high noise level
interview	62	P00	62		Turnraum	negativ	62	saisonale Temperaturprobleme: im Sommer viel zu heiß	62	seasonal temperature issues: much too hot in summer
interview	63	P00	63		Turnraum	positiv	63	Es werden Wesco-Turnelemente verwendet, diese stehen in einer Ecke im Turnraum; mehr Platz oder ein System, um diese Elemente besser zu verstauen wünschenswert.	63	We use Wesco gym modules, those are in a corner of the gym; more space or a system to better store these elements is desirable.
interview	64	P00	64		Turnraum	negativ	64	Der Turnraum hat zwei große runde Fenster auf den Gang. Es kommt vor, dass Eltern draußen warten und zusehen oder andere Kinder von außen an die Fenster klopfen; das lenkt die Kinder ab und stört die MA teilweise bei der Arbeit.	64	The gym has two large round windows onto the corridor. It happens that parents are waiting outside and watching or other children are knocking on the windows from outside; this distracts the children and sometimes disturbs the employees at work.
interview	65	P00	65		Kreativraum	positiv	65	gute Raumgröße / ausreichend Platz für kreatives Arbeiten	65	good room size / sufficient space for creative work
interview	66	P00	66		Kreativraum	positiv	66	gute Belüftungsmöglichkeit, obwohl im Keller gelegen	66	good ventilation possibility, although located in the basement
interview	67	P00	67		Kreativraum	positiv	67	Tischgruppen - sozial-verbindend	67	table groups – socially connecting
interview	68	P00	68		Kreativraum	negativ	68	Der Raum wird auch als Materiallager genutzt (ca. ein Viertel der Raumfläche). Der Raum bietet nicht genügend Platz für Stauraum - es wird teilweise außerhalb des Raumes (Treppenbereich) Material gelagert.	68	The room is also used as a material storage (approx. a quater of the room area). The room does not offer enough space for storage - material is sometimes stored outside the room (stair area).
interview	69	P00	69		Kreativraum	negativ	69	Es gibt Fenster, diese liegen aber in einem Schacht, Ausblick auf Wand. Es gibt kein natürliches Licht, der Raum muss immer mit künstlichem Licht beleuchtet werden.	69	There are windows, but these are in a shaft, view of the wall. There is no natural light, the room must always be illuminated with artificial light.

source	#src	id	#rid	group	area	quality	#r	original content (german)	#r	content translation (english)
interview	70	P00	70		Kreativraum	negativ	70	Unzureichende Möglichkeit zur Temperaturreguierung - der Raum ist im Winter zu warm.	70	Insufficient possibility to regulate the temperature – the room is too warm in winter.
interview	71	P00	71		Kreativraum	negativ	71	Das Kreativmaterial ist für Kinder nicht frei zugänglich bzw. kann von den MA nicht gezielt so positioniert und präsentiert werden, dass die Kinder zu kreativen Aktivitäten eingeladen werden. Es fehlt kindgerechte Präsentationsfläche.	71	The creative material is not freely accessible to children and cannot be specifically positioned and presented by the staff in such a way that the children are invited to creative activities. There is no child-appropriate presentation area.
interview	72	P00	72		Kreativraum	negativ	72	Der Raum wirkt insgesamt sehr chaotisch und unsystematisch, ein systematischer - ev. abgetrennter - Stauraum fehlt.	72	Overall, the room appears very chaotic and unsystematic, there is no systematic - possibly separated - storage space.
interview	73	P00	73		Kreativraum	negativ	73	zu wenig freie Raumgestaltungsmöglichkeiten (u.a. aus Platzgründen)	73	too few room design options (i.a. for reasons of space)
interview	74	P00	74		Essbereich	positiv	74	gute Lichtverhältnisse, Fensterfront mit sehr viel natürlichem Licht	74	good light conditions, window front with a lot of natural light
interview	75	P00	75		Essbereich	positiv	75	gute Belüftungsmöglichkeit	75	good ventilation option
interview	76	P00	76		Essbereich	positiv	76	Bodenmaterial - wenn Geschirr (Keramik) hinunterfällt, dann zerbricht es nicht.	76	Floor material - if crockery (ceramics) falls, it doesn't break.
interview	77	P00	77		Essbereich	negativ	77	Viel zu wenig Platz, enger Raum! Eine einzelne Gruppe muss aufgteilt werden, um zu jausnen. Die Sitzanordnung trotzdem viel zu eng. Grundsätzlich wird der Raum als wenig sozial-verbindend angesehen.	77	Far too little space, narrow space! A single group must be split up to have a snack. The seating arrangement is still way too narrow. Basically, the room is seen as little socially connecting.
interview	78	P00	78		Essbereich	negativ	78	Schlechte akustische Bedingungen, sehr hoher Geräuschpegel (hallend). Der Jausenbereich ist ein offener Raum - Lärm von allen Seiten.	78	Poor acoustic conditions, very high noise level (reverberating). The eating area is an open space - noise from all sides.
interview	79	P00	79		Essbereich	negativ	79	keine Beschattungsmöglichkeit - im Sommer viel zu heiß	79	no possibility of shading - much too hot in summer
interview	80	P00	80		Essbereich	negativ	80	Ausblick von der Fensterfront ins Graue - keine Grünbereiche	80	view from the window front into the grey - no green areas
interview	81	P00	81		Essbereich	negativ	81	Der Raum ist sehr eng mit Dachschrägen - für Erwachsene / MA zu wenig Platz, ein "Labyrinth" mit vielen Hindernissen.	81	The room is very narrow with sloping ceilings - not enough space for adults / staff, a "labyrinth" with many obstacles.
interview	82	P00	82		Essbereich	negativ	82	Insgesamt wenig einladend, um zu jausnen.	82	Overall not very inviting to have a snack.
interview	83	P00	83		Schlafraum	positiv	83	Es gab ursprünglich zwei kleine Schlafräume - beide durch den Gelben Gruppenraum zugänglich. Bei Schlafraum 1 (zum Gang gelgegen) wurde eine Wand entfernt. Die entstandene Nische im Gruppenraum wurde notgedrungen als Rückzugsort für die Kinder umfunktioniert. Schlafraum 2 (zur Außenwand gelegen) ist ein sehr kleiner Raum (Platz für ca. 3 Kinder). Derzeit wird dieser Raum als zusätzlicher Stauraum und nicht als Schlafraum genutzt, da kein Bedarf. Selbst bei Bedarf könnte der Raum nicht seiner angedachten Funktion entsprechend genutzt werden.	83	There were originally two small dormitories - both accessible through the yellow group room. A wall was removed from bedroom 1 (next to the corridor). Out of necessity, the resulting niche in the group room was converted into a retreat for the children. Bedroom 2 (facing the outside wall) is a very small room (space for about 3 children). Currently this space is used as extra storage space and not as a dormitory as there is no need. Even if necessary, the room could not be used according to its intended function.
interview	84	P00	84		Schlafraum	negativ	84	Platzmangel - zu klein, zu eng für mehrere Liegemöglichkeiten	84	lack of space - too small, too narrow for several reclining options
interview	85	P00	85		Schlafraum	negativ	85	Ungünstige Lage des Raums, nur durch Gelben Gruppenraum begehbar, Fenster mit Blick auf Spielbereich - Kinder, die schlafen wollen, werden abgelenkt bzw. gestört.	85	Unfavourable location of the room, only accessible through the yellow group room, window overlooking the play area - children who want to sleep are distracted or disturbed.
interview	86	P00	86		Schlafraum	negativ	86	schlechte akustische Bedingungen - Geräuschepegel von Nebenräumen - Kinder, die schlafen wollen, werden gestört	86	poor acoustic conditions - noise level from adjoining rooms - children who want to sleep are disturbed
interview	87	P00	87		Garten	positiv	87	vier Zugangsmöglichkeiten - separat für jede Gruppe (zwei Außentreppen für OG)	87	four access options – separately for each group (two external stairs for upper floor)
interview	88	P00	88		Garten	positiv	88	an den Außenwänden gibt es Haken für Kleidung und Rucksäcke	88	on the outer walls there are hooks for clothes and backpacks
interview	89	P00	89		Garten	positiv	89	überdachte Terrasse - bietet gute Beschattung und Regenschutz	89	covered terrace - offers good shade and rain protection
interview	90	P00	90		Garten	positiv	90	mittlerweile ausreichend Platz und Grünbereich (wurde kürzlich erweitert)	90	now sufficient space and green area (was recently expanded)
interview	91	P00	91		Garten	positiv	91	"Naschgarten" - an den Zäunen befinden sich in kindgerechter Höhe Pflanzenkistchen mit Erdbeeren, Ribisel etc., die Kinder können sich frei bedienen.	91	"Snack garden" – on the fences there are plant boxes with strawberries, currants, etc. at a child-appropriate height, the children can help themselves.

source	#src	id	#rid	group	area	quality	#r	original content (german)	#r	content translation (english)
interview	92	P00	92		Garten	positiv	92	Es ist eine asphaltierte Fläche vorhanden - hier können die Kinder besser mit Laufrädern spielen und Bälle prellen, als auf der Grünfläche.	92	There is an asphalt area - children can play with wheels and bounce balls better here than on the green area.
interview	93	P00	93		Garten	positiv	93	Die Kinder finden die Steine neben den Außenflächen sehr interessant und einladend. Sie haben große Freude daran, die Steine aufeinander zu klopfen, sie zu sammeln und mit ihnen unterhalb der Außentreppe etwas zu bauen.	93	The children find the stones next to the outside walls very interesting and inviting. They take great pleasure in knocking the stones together, collecting them and building something with them below the outside stairs.
interview	94	P00	94		Garten	negativ	94	zu wenig Schattenplätze	94	too few shadow places
interview	95	P00	95		Garten	negativ	95	keine Bäume	95	no trees
interview	96	P00	96		Garten	negativ	96	Ausstattung nicht ausreichend - bei derzeit 100 Kindern können theoretisch alle Kinder gleichzeitig in den Garten, aber sie können nicht alle gleichzeitig spielen (nur 1 Rutsche, 1 Schaukel etc.). Es entstehen Staus in den Spielbereichen. Dies wird als wenig sozial-verbindend empfunden.	96	Insufficient equipment - with currently 100 children, theoretically all children can go into the garden at the same time, but they cannot all play at the same time (only 1 slide, 1 swing, etc.). There are jams in the play areas. This is perceived as little socially connecting.
interview	97	P00	97		Garten	negativ	97	Kein Stauraum für Spielfahrzeuge vorhanden. Die Fahrzeuge werden derzeit am EG-Zugang an der Außenwand abgestellt - wird als störend empfunden.	97	There is no storage space for toy vehicles. The vehicles are currently parked at the ground floor access next to the outside wall - this is perceived as hindering.
interview	98	P00	98		Garten	negativ	98	Die Asphalfläche ist zu klein für bestimmte Spiele oder Aktivitäten.	98	The asphalt area is too small for certain games or activities.
interview	99	P00	99		Garten	positiv	99	Es wird zwar begrüßt, dass die Kinder mit den Steinen bauen, aber der Platz unterhalb der Außentreppe ist ungünstig, für die MA schwer zu kontrollieren - kann gefährlich sein.	99	We welcome that the children build with the stones, but the space below the outside stairway is unfavourable, it is difficult to control for the employees – can be dangerous.
interview	100	P00	100		Personalraum	positiv	100	Tische und Sitzplätze für Erwachsene vorhanden	100	tables and seats for adults available
interview	101	P00	101		Personalraum	positiv	101	Fensterfront mit natürlichem Licht und Blick ins Grüne	101	window front with natural light and view of the greenery
interview	102	P00	102		Personalraum	positiv	102	natürliche Bauelemente - viel Holz	102	natural building materials – lots of wood
interview	103	P00	103		Personalraum	negativ	103	Anzahl der Sitzplätze nicht ausreichend für alle MA	103	number of seats not enough for all staff
interview	104	P00	104		Personalraum	negativ	104	Temperatur nicht ausreichend regulierbar - saisonale Extreme, v.a. im Sommer viel zu heiß (+30°C)	104	temperature not sufficiently adjustable - seasonal extremes, especially in summer much too hot (+30°C)
interview	105	P00	105		Personalraum	negativ	105	Schlechte akustische Bedingungen, sehr hoher Geräuschpegel	105	poor acoustic conditions, very high noise level
interview	106	P00	106		Personalraum	negativ	106	Der Raum ist verwinkelt - teilweise ungünstige Lichtverhältnisse.	106	The room is angled - partially unfavourable lighting conditions.
interview	107	P00	107		Personalraum	negativ	107	Der Raum ist eine offene Kombination mit der Küche, in der auch Essen ausgegeben wird (Küche im Eingangsbereich, Jausenraum für Kinder vor dem Raum) - es gibt keinen geschlossenen Rückzugsort für MA.	107	The room is an open combination with the kitchen where food is also served (kitchen in the entrance area, eating area for children in front of the room) - there is no enclosed retreat for employees.
interview	108	P00	108		Personalgarderobe	negativ	108	viel zu klein, zu wenig Platz, zu wenig Stauraum (ca. $3m^2$) - "Abstellkammer"; mehrere MA können sich nicht gleichzeitig	108	far too small, too little space, not enough storage space (approx. $3m^2$) – "storage room"; several employees cannot change at the
interview	109	P00	109		Personalgarderobe	negativ	109	schlechte Lichtverhältnisse, keine Fenster	109	bad lighting conditions, no windows
interview	110	P00	110		Personalgarderobe	negativ	110	schlechte Luft, da keine Lüftungsmöglichkeit	110	bad air, as there is no possibility of ventilation
interview	111	P00	111		Personal-WC	negativ	111	keine Belüftungsmöglichkeit im barrierefreien Personal-WC	111	no possibility of ventilation in the barrier-free staff toilet
survey	1	P01	1	orange	Gruppenraum	positiv	112	Küchenzeile	112	kitchenette
survey	2	P01	2	orange	Garderobe	positiv	113	extra Garderobenraum mit Türe (also nicht im offenen Vorhaus); für AEG/ Krabbelgruppe sehr positiv	113	separate cloakroom with door (so not in the open entrance hall); for AEG / toddler group very positive
survey	3	P01	3	orange	Gruppenraum	negativ	114	Lichtverhältnisse	114	lighting conditions
survey	4	P01	4	orange	Gruppenraum	negativ	115	"ohne Fenster" (bzw. kein Tageslicht)	115	"without windows" (or no daylight)
survey	5	P01	5	orange	Gruppenraum	negativ	116	keine Akustikdecke	116	no acoustic ceiling
survey	6	P01	6	orange	Gruppenraum	negativ	117	beim Lüften kommt keine frische Luft herein	117	when airing, no fresh air comes in
survey	7	P01	7	orange	Waschraum	positiv	118	unser "Erwachsenen WC" ist gleich nebenbei	118	our "adult’s toilet" is right next
survey	8	P01	8	orange	Waschraum	negativ	119	zu weit weg vom Gruppenraum	119	too far away from the group room
survey	9	P01	9	orange	Waschraum	negativ	120	Lichtqualität	120	lighting quality
survey	10	P01	10	orange	Waschraum	negativ	121	kein ausreichendes Lüften möglich	121	airing not sufficiently possible

source	#src	id	#rid	group	area	quality	#r	original content (german)	#r	content translation (english)
survey	11	P01	11	orange	Waschraum	negativ	122	Größe des WC's (Erwachsenenklo); Stufen gebaut und Kloring so ist es in Ordnung	122	size of toilets (adult’s toilet); built stages and a toilet seat now it’s fine
survey	12	P01	12	orange	Waschraum	negativ	123	Waschbecken + Seifenspender für kleine Kinder nicht erreichbar, trotz Stufe	123	sink + soap dispenser not accessible for small children, despite stage
survey	13	P01	13		Turnraum	positiv	124	anschließend zum Gruppenraum	124	adjoining to the group room
survey	14	P01	14		Turnraum	negativ	125	viel zu klein	125	much too small
survey	15	P01	15		Turnraum	negativ	126	keine Kästen	126	no cabinets
survey	16	P01	16		Turnraum	negativ	127	Lichtqualität	127	lighting quality
survey	17	P01	17		Turnraum	negativ	128	ebenfalls kein Lüften möglich	128	also ventilation not possible
survey	18	P01	18		Eingangsbereich	negativ	129	langer, dunkler Gang, ohne Fenster, ohne Gestaltungsmöglichkeit (außer Bilder an der Wand)	129	long, dark corridor, without windows, without design options (except for pictures on the wall)
survey	19	P01	19		Eingangsbereich	negativ	130	ohne Akustikdecke - sehr laut	130	without acoustic ceiling - very loud
survey	20	P01	20		Garten	positiv	131	neues Gartenhaus	131	new garden house
survey	21	P01	21		Garten	positiv	132	Kräuter, Obst, Bepflanzung (Naschgarten)	132	herbs, fruits, plants (snack garden)
survey	22	P01	22		Garten	positiv	133	Terrasse im Schatten	133	terrace in the shadow
survey	23	P01	23		Garten	negativ	134	zu wenig Schattenplätze	134	too few shaded places
survey	24	P01	24		Garten	negativ	135	keine Klettermöglichkeiten	135	no climbing options
survey	25	P01	25		Garten	negativ	136	Fahrzeuge Parkplatz (z.B. Laufrad) liegt derzeit under der Stiege zum oberen Stock	136	vehicle parking (e.g. impeller) is currently under the stairs to the upper floor
survey	26	P01	26		Garten	negativ	137	mehr Spielmöglichkeiten fehlen (z.B. Matschbereich, Wasserbereich, Kugelbahn, Klettergerüst)	137	more playing options are lacking (e.g. mud area, water area, marble run, climbing frame)
survey	27	P01	27		Garten	negativ	138	sehr wenig Sitzgelgenheiten und diese wenigen sind nicht abgeschliffen; Kinder bekommen Schiefer/"Splitter"	138	very little options to sit and those few available are not sanded down; children get splinters
survey	28	P01	28		Garten	negativ	139	nicht sehr praktisch	139	not very practical
survey	29	P01	29		Eingangsbereich	positiv	140	sehr hell und groß	140	very bright and big
survey	30	P01	30		Eingangsbereich	positiv	141	Glastüren	141	glass doors
survey	31	P01	31		Eingangsbereich	negativ	142	kein Türöffner (Sicherung in Erwachsenenhöhe)	142	no door opener (safety at adult height)
survey	32	P01	32		Eingangsbereich	negativ	143	[Wunsch] Einrichtung für Schuhfreien KiGa (z.B. Sitzmöglichkeit, Abstellplatz f. Schuhe, Garderobe)	143	[request] furniture for shoe-free kindergarten (e.g. seating option, storage space for shoes, wardrobe)
survey	33	P01	33		Personalraum	positiv	144	wirkt hell und freundlich	144	looks bright and friendly
survey	34	P01	34		Personalraum	negativ	145	viel zu klein	145	much too small
survey	35	P01	35		Personalraum	negativ	146	Kinderküche und Erwachsenenküche und Essmöglichkeiten sind nicht getrennt (auch in der Pause sind Kinder im Raum oder die Mittagstischdienst-Damen sind immer im Raum)	146	children's kitchen and adult's kitchen and eating areas are not separated (even during the break children are in the room or the lunch service ladies are always in the room)
survey	36	P01	36		Essbereich	negativ	147	Kegel (im Winter kalt, im Sommer Sauna)	147	Cone (cold in winter, sauna in summer)
survey	37	P01	37		Personalraum	negativ	148	für den Mittagstisch (Kinder) sehr unpraktisch aufgeteilt	148	for lunch table (children) very impractically divided
survey	38	P01	38		Essbereich	positiv	149	wirkt hell und freundlich	149	looks bright and friendly
survey	39	P01	39		Essbereich	negativ	150	ohne Tür zum Eingangsbereich (Aula) - kein abgeschlossener Raum	150	without a door to the entrance area (hall) - not an enclosed room
survey	40	P01	40		Essbereich	negativ	151	Kegelform	151	cone shape
survey	41	P01	41		Essbereich	negativ	152	viel zu klein daher sehr unpraktisch	152	way too small so very impractical
survey	42	P01	42		Essbereich	negativ	153	viel zu wenig Kinder haben Platz	153	far too few children have space
survey	43	P01	43		Essbereich	negativ	154	[Wunsch] Kleinere Tische - dafür mehr wären super! Wenn eine lange Tafel ist, besteht immer Unruhe.	154	[request] Smaller tables - but more would be great! When there is a long table, there is always unrest.
survey	44	P01	44		Personalgarderobe	positiv	155	Spint	155	locker
survey	45	P01	45		Personalgarderobe	negativ	156	zu wenig Spint (6 Stk. für 14 Mitarbeiter)	156	too few lockers (6 pc for 14 employees)
survey	46	P01	46		Personalgarderobe	negativ	157	zu klein für so viele Mitarbeiter	157	too small for so many employees
survey	47	P01	47		Personalgarderobe	negativ	158	kein Fenster	158	no windows
survey	48	P01	48		Personalgarderobe	negativ	159	fast keine Abstellmöglichkeit (nicht genug für jeden)	159	barely any storage options (not enough for everyone)
survey	49	P01	49		Personalgarderobe	negativ	160	weit weg vom Eingangsbereich	160	far away from the entrance area
survey	50	P02	1	N/A	Gruppenraum	negativ	161	[Wunsch] bessere Abtrennungen in Gruppenräumen	161	[request] better separation in group rooms

source	#src	id	#rid	group	area	quality	#r	original content (german)	#r	content translation (english)
survey	51	P02	2	N/A	Gruppenraum	negativ	162	[Wunsch] größere Gruppenräume	162	[request] larger group rooms
survey	52	P02	3		Gebäude	negativ	163	[Wunsch] mehrere Ausweichmöglichkeiten	163	[request] more alternative room options
survey	53	P02	4		Essbereich	negativ	164	[Wunsch] größerer Jausenbereich	164	[request] larger eating area
survey	54	P02	5		Turnraum	positiv	165	Wesco-Teile	165	Wesco parts
survey	55	P02	6		Turnraum	positiv	166	Langbänke	166	long benches
survey	56	P02	7		Turnraum	positiv	167	Lichtverhältnisse	167	lighting conditions
survey	57	P02	8		Turnraum	negativ	168	keine Sprossenwand	168	no wall bars
survey	58	P02	9		Turnraum	negativ	169	wenige Geräte für einen Parcours aufbauen	169	few equipment for setting up an obstacle course
survey	59	P02	10	N/A	Garderobe	positiv	170	die Bänke sind sehr gut zum sitzen für die Kinder	170	the benches are very good for the children to sit on
survey	60	P02	11	N/A	Garderobe	positiv	171	der Platz der Garderobe (vor dem Gruppenraum) ist sehr gut	171	the location of the wardrobes (in front of the group room) is very
survey	61	P02	12	N/A	Garderobe	negativ	172	die Sitzbänke sind getrennt, somit kann man in der Garderobe schlecht etwas besprechen, da nicht alle Kinder beisammen sitzen	172	the benches are separate, so it is difficult to discuss things in the wardrobe area, since not all the children are sitting together
survey	62	P02	13	N/A	Garderobe	negativ	173	sehr eng	173	very narrow
survey	63	P02	14		Essbereich	negativ	174	[Wunsch] abgeschlossener Bereich	174	[request] enclosed area
survey	64	P02	15		Essbereich	negativ	175	sehr eng, wenn alle Kinder gemeinsam jausnen	175	very narrow when all children eat together
survey	65	P02	16		Essbereich	negativ	176	sehr heiß im Sommer	176	very hot in summer
survey	66	P02	17	N/A	Waschraum	positiv	177	gut für Kinder erreichbar	177	easily accessible for children
survey	67	P02	18	N/A	Waschraum	negativ	178	[Wunsch] mehr Staumöglichkeiten	178	[request] more storage options
survey	68	P03	1	N/A	Gruppenraum	negativ	179	[Wunsch] wenn man Gruppenräume besser abtrennen könnte	179	[request] if we could divide the group rooms better
survey	69	P03	2		Essbereich	negativ	180	[Wunsch] größerer Jausenraum	180	[request] larger eating area
survey	70	P03	3		Turnraum	positiv	181	Langbänke	181	long benches
survey	71	P03	4		Turnraum	negativ	182	wenig Geräte	182	few equipment
survey	72	P03	5	N/A	Garderobe	positiv	183	sehr gut, dass die Garderobe vor dem Gruppenraum ist	183	very good that the wardrobes are in front of the group room
survey	73	P03	6	N/A	Garderobe	negativ	184	zu wenig Platz	184	too little space
survey	74	P03	7		Kreativraum	positiv	185	ausreichend Platz	185	sufficient space
survey	75	P03	8		Kreativraum	negativ	186	sehr heiß	186	very hot
survey	76	P03	9		Essbereich	negativ	187	zu wenig Platz, wenn alle gemeinsam jausnen	187	too little space when everyone is eating together
survey	77	P04	1		Eingangsbereich	negativ	188	zu klein	188	too small
survey	78	P04	2		Eingangsbereich	negativ	189	soll offener sein	189	should be more open
survey	79	P04	3		Eingangsbereich	negativ	190	Garderobe für KiGa Personal fehlt	190	wardrobe for kindergarten staff is lacking
survey	80	P04	4		Personalraum	positiv	191	relativ gut ausgestattet	191	relatively well equipped
survey	81	P04	5		Personalraum	negativ	192	zu klein ist die Küche	192	the kitchen is too small
survey	82	P04	6		Personalraum	negativ	193	Abtrennung zum Personalraum fehlt	193	separtation to staff room is lacking
survey	83	P04	7		Personalraum	negativ	194	viel zu heiß oder zu kalt	194	much too hot or too cold
survey	84	P04	8		Essbereich	positiv	195	Möbel OK	195	furniture okay
survey	85	P04	9		Essbereich	negativ	196	zu nieder	196	too low
survey	86	P04	10		Essbereich	negativ	197	zu klein	197	too small
survey	87	P04	11		Essbereich	negativ	198	zu unpraktisch	198	too impractical
survey	88	P04	12		Essbereich	negativ	199	keine Abtrennung	199	no separation
survey	89	P04	13	grün	Garderobe	positiv	200	Garderobe ist groß genug	200	wardrobe is big enough
survey	90	P04	14	grün	Waschraum	positiv	201	WC passt!	201	WC alright!
survey	91	P04	15	grün	Gruppenraum	negativ	202	zu klein (Gruppenraum)	202	too small (group room)
survey	92	P04	16	grün	Gruppenraum	negativ	203	zu viele Möglichkeiten, sich den Kopf anzustoßen	203	too many opportunities to bump your head
survey	93	P04	17	grün	Gruppenraum	negativ	204	Gruppenraum ist zu heiß/kalt	204	group room is too hot/cold
survey	94	P04	18		Eingangsbereich	positiv	205	alles gut	205	everything alright
survey	95	P04	19	grün	Waschraum	negativ	206	zu klein (keine Möglichkeit den Wäscheständer aufzustellen)	206	too small (no possibility to set up the drying rack)
survey	96	P04	20		Kreativraum	positiv	207	gut ausgestattet	207	well equipped
survey	97	P04	21		Kreativraum	negativ	208	keine Abtrennung	208	no separation
survey	98	P05	1		Gebäude	negativ	209	für mich zu viele Fenster	209	too many windows for me

source	#src	id	#rid	group	area	quality	#r	original content (german)	#r	content translation (english)
survey	99	P05	2		Eingangsbereich	negativ	210	Garderobe für Personal fehlt	210	wardrobe for staff is lacking
survey	100	P05	3		Eingangsbereich	negativ	211	zu klein, soll größer sein	211	too small, should be larger
survey	101	P05	4		Personalraum	positiv	212	Küche relativ gut ausgestattet	212	kitchen is relatively well equipped
survey	102	P05	5		Personalraum	negativ	213	zu klein ist die Küche	213	the kitchen is too small
survey	103	P05	6		Personalraum	negativ	214	Abtrennung zum Personalraum fehlt	214	separtation to staff room is lacking
survey	104	P05	7		Personalraum	negativ	215	viel zu heiß im Sommer, im Winter zu kalt	215	much too hot in summer, too cold in winter
survey	105	P05	8		Essbereich	positiv	216	Tisch, Sessel und Bank sehr gut für die Kinder	216	table, chairs and bench very good for the children
survey	106	P05	9		Essbereich	negativ	217	zu klein ist der Raum	217	the room is too small
survey	107	P05	10		Essbereich	negativ	218	keine Abtrennung vorhanden	218	no separation available
survey	108	P05	11	grün	Garderobe	positiv	219	Garderobe ist groß genug für die Anzahl der Kinder	219	wardrobe is big enough for the number of children
survey	109	P05	12	grün	Waschraum	positiv	220	WC passt auch für die Gruppengröße	220	WC is alright too for the group size
survey	110	P05	13	grün	Gruppenraum	negativ	221	zu klein	221	too small
survey	111	P05	14	grün	Gruppenraum	negativ	222	rechts vom Eingang stößt man sich mehrmals den Kopf	222	to the right of the entry we bump our heads several times
survey	112	P05	15	grün	Gruppenraum	negativ	223	Gruppenraum zu heiß im Sommer, zu kalt im Winter	223	group room too hot in summer, too cold in winter
survey	113	P05	16		Eingangsbereich	positiv	224	ist offen	224	is open
survey	114	P05	17		Eingangsbereich	positiv	225	passt gut	225	is alright
survey	115	P05	18	grün	Gruppenraum	negativ	226	zu klein	226	too small
survey	116	P05	19	grün	Waschraum	negativ	227	keine Möglichkeit, um die Wäsche zu trocknen	227	no possibility to dry the laundry
survey	117	P05	20		Kreativraum	positiv	228	Möbel genug vorhanden	228	enough furniture available
survey	118	P05	21		Kreativraum	positiv	229	gut ausgestattet mit Bastel- und Malsachen	229	well equipped with handicraft and painting materials
survey	119	P05	22		Kreativraum	negativ	230	keine Abtrennung im Raum für Bastelsachen	230	no separation in the room for handicraft materials
survey	120	P05	23		Gebäude	positiv	231	Ich arbeite sehr gerne hier und die Kollegen sind sehr nett, hilfsbereit. Alles in bester Ordnung.	231	I like to work here and the colleagues are very nice, helpful. Everything is alright.
survey	121	P06	1	orange	Gruppenraum	positiv	232	Küchenzeile	232	kitchenette
survey	122	P06	2	orange	Garderobe	positiv	233	Garderobenraum mit Türe (nicht im offenen Vorraum / Vorhaus)	233	wardrobe with a door (not in the open hall / entrance hall)
survey	123	P06	3	orange	Gruppenraum	negativ	234	Licht; kein Tageslicht bzw. kein Blick ins Freie	234	light; no daylight or rather no view of the outside
survey	124	P06	4	orange	Gruppenraum	negativ	235	keine Akustikdecke (teilweise laut)	235	no acoustic ceiling (sometimes loud)
survey	125	P06	5	orange	Gruppenraum	negativ	236	kommt keine richtig frische Luft in den Raum (Lüften)	236	no fresh air is coming into the room (airing)
survey	126	P06	6		Personal-WC	positiv	237	Personal WC neben Kinder-WC	237	staff toilet next to children’s toilet
survey	127	P06	7	orange	Waschraum	negativ	238	Entfernung zum Gruppenraum ist zu weit (nicht praktisch)	238	distance to the group room is too far (not practical)
survey	128	P06	8	orange	Waschraum	negativ	239	Lichtqualität (nur künstliches Licht)	239	lighting quality (only artificial light)
survey	129	P06	9	orange	Waschraum	negativ	240	Lüften nicht möglich (nur über Kellerabgangstür)	240	airing not possible (only via basement door)
survey	130	P06	10	orange	Waschraum	negativ	241	WC Größe (Erwachsenengröße) --> Stufen gebaut + Kloring --> so ist es in Ordnung	241	toilet size (adult’s size) --> built stages + toilet seat --> it's fine like this
survey	131	P06	11	orange	Waschraum	negativ	242	Waschbecken zu groß --> Seife für Kinder trotz Stufe nicht	242	sinks too large --> soap for children not reachable despite stage
survey	132	P06	12		Turnraum	positiv	243	anschließend zum Gruppenraum	243	adjoining to the group room
survey	133	P06	13		Turnraum	negativ	244	viel zu klein	244	much too small
survey	134	P06	14		Turnraum	negativ	245	keine Kästen	245	no cabinets
survey	135	P06	15		Turnraum	negativ	246	Licht --> nicht ausreichend (Tageslicht)	246	light --> insufficient (daylight)
survey	136	P06	16		Turnraum	negativ	247	ebenfalls keine richtige frische Luft beim Lüften	247	also no really fresh air when airing
survey	137	P06	17		Eingangsbereich	negativ	248	langer, dunkler Flur ohne Fenster, ohne Gestaltungsmöglichkeiten	248	long, dark corridor, without windows, without design options
survey	138	P06	18		Eingangsbereich	negativ	249	Akustik, sehr schlecht, sehr laut…	249	acoustics, very bad, very loud…
survey	139	P06	19		Garten	positiv	250	Neugestaltung von Gartenhaus und Sandkiste	250	redesign of garden house and sandpit
survey	140	P06	20		Garten	positiv	251	Naschgarten	251	snack garden
survey	141	P06	21		Garten	positiv	252	Terrasse mit Hängematten im Schatten	252	terrace with hammocks in the shadow
survey	142	P06	22		Garten	negativ	253	zu wenig Schattenplätze	253	too few shaded places
survey	143	P06	23		Garten	negativ	254	keine Klettermöglichkeiten	254	no climbing options
survey	144	P06	24		Garten	negativ	255	Fahrzeuge (z.B. Laufrad) kein Parkplatz (liegen nur rum)	255	vehicles (e.g. impellers) no parking space (just lying around)

source	#src	id	#rid	group	area	quality	#r	original content (german)	#r	content translation (english)
survey	145	P06	25		Garten	negativ	256	[Wunsch] mehr Spielmöglichkeiten (z.B. "Gatsch-Wasser-Bereich", Klettergerüst, Baumstamm)	256	[request] more playing options (e.g. "mud-water-area", climbing frame, tree trunk)
survey	146	P06	26		Garten	negativ	257	wenig Sitzgelegenheiten --> diese, was wir haben sind jedes Jahr zum Abschleifen, sonst haben Ki. Splitter in Hand stecken oder irgendwo drinnen	257	few seating options --> those we have, have to be sanded down every year, otherwise the children get splinters stuck in their hands or elsewhere
survey	147	P06	27		Eingangsbereich	positiv	258	Aula sehr hell und groß	258	hall very bright and big
survey	148	P06	28		Eingangsbereich	positiv	259	Glastüren; hell, kommt viel Licht rein	259	glass doors; bright, much light comes in
survey	149	P06	29		Eingangsbereich	negativ	260	kein Türöffner in Höhe, wo es Kinder nicht erreichen können; Kinder können einfach raus	260	no door opener at a height where children cannot reach it; kids can just go out
survey	150	P06	30		Eingangsbereich	negativ	261	[Wunsch] Einrichtung für Schuhfreien-Kindergarten, z.B. Sitzmöglichkeit, Abstellplatz für Schuhe	261	[request] furniture for a shoe-free kindergarten, e.g. seating options, storage space for shoes
survey	151	P06	31		Personalraum	positiv	262	Raum wirkt hell und freundlich	262	room looks bright and friendly
survey	152	P06	32		Personalraum	positiv	263	gut zu Lüften	263	well to ventilate
survey	153	P06	33		Personalraum	negativ	264	viel zu klein	264	much too small
survey	154	P06	34		Personalraum	negativ	265	Kinderküche für Mittagessen und Personalraum für Pause ist alles eines; zu klein, in der Pause keine Ruhe	265	children's kitchen for lunch and staff room for breaks are all one; too small, no rest during the break
survey	155	P06	35		Personalraum	negativ	266	Kinderküche / Mittagessenraum sollte extra sein und eine eigene Tür haben (abgetrennter Raum)	266	children's kitchen / lunch room should be extra and have an own door (separate room)
survey	156	P06	36		Essbereich	negativ	267	"Kegel" --> heiß im Sommer, kalt im Winter	267	"Cone" --> hot in summer, cold in winter
survey	157	P06	37		Personalraum	negativ	268	Raum für Besprechungen zu klein, fast kein Platz für alle Mitarbeiter	268	room for meetings too small, almost no space for all staff
survey	158	P06	38		Essbereich	negativ	269	Kegelform viel zu klein	269	Cone shape much too small
survey	159	P06	39		Essbereich	negativ	270	keine Türe zur Aula / Eingangsbereich	270	no door to the hall / entrance hall
survey	160	P06	40		Essbereich	negativ	271	große Tische mit viel Kindern --> Unruhe; zum Mittagessen wäre besser kleinere Tische, wo wenige Kinder Platz haben (ruhiger beim Essen)	271	large tables with lots of children --> unrest; for lunch it would be better to have smaller tables where fewer children have space (quieter when eating)
survey	161	P06	41		Personalgarderobe	positiv	272	Spint (Kastenfach)	272	locker (locker compartment)
survey	162	P06	42		Personalgarderobe	negativ	273	leider zu wenige Spints für alle Mitarbeiter	273	unfortunately too few lockers for all employees
survey	163	P06	43		Personalgarderobe	negativ	274	etwas zu klein für so viele Mitarbeiter	274	a bit too small for so many employees
survey	164	P06	44		Personalgarderobe	negativ	275	weiter weg vom Eingangsbereich; besser wäre gleich neben dem Eingang	275	further away from the entrance area; would be better right next to the entrance
survey	165	P07	1		Gebäude	negativ	276	keine Ausweichräume für Zusatzangebote :-(	276	no alternative rooms for additional activities :-(
survey	166	P07	2		Personal-WC	negativ	277	zu weit weg - langer Weg	277	too far away - long way
survey	167	P07	3		Kreativraum	negativ	278	Stauraum unter der Stiege zu klein - eigentlich nicht dafür	278	storage space under the stairs too small - actually not intended for it
survey	168	P07	4		Personalgarderobe	negativ	279	zu wenig Fächer, dass jede Kollegin ein Fach hat	279	too few compartments, that every colleague has a compartment
survey	169	P07	5		Personalgarderobe	negativ	280	zu wenig Platz für Schuhe bzw. im Winter mit den Jacken	280	too little space for shoes or in winter with the jackets
survey	170	P07	6		Personalraum	positiv	281	hell	281	bright
survey	171	P07	7		Personalraum	negativ	282	wird schnell heiß	282	gets hot fast
survey	172	P07	8		Personalraum	negativ	283	stickig	283	stuffy
survey	173	P07	9		Personalraum	negativ	284	nicht reiner Personalraum aus Platzmangel	284	not pure staff room due to lack of space
survey	174	P07	10		Personalraum	negativ	285	Stauraum für Geschirr	285	storage space for dishes
survey	175	P07	11		Personalraum	negativ	286	Ameisen!!	286	ants!!
survey	176	P07	12		Personalraum	negativ	287	[Wunsch] die Küchenzeile sollte dem Zweck angepasst werden	287	[request] the kitchenette should be adapted to the purpose
survey	177	P07	13		Essbereich	positiv	288	hell	288	bright
survey	178	P07	14		Essbereich	negativ	289	schnell heiß	289	quickly hot
survey	179	P07	15		Essbereich	negativ	290	stickige Luft	290	stuffy air
survey	180	P07	16		Essbereich	negativ	291	Ameisen!	291	ants!
survey	181	P07	17		Turnraum	positiv	292	Markierungen am Boden	292	markings on the floor
survey	182	P07	18		Turnraum	positiv	293	Schaumstoffteile!	293	foam parts!
survey	183	P07	19		Turnraum	negativ	294	Geräteraum ist gleichzeitig Putzkammerl	294	equipment room is also cleaning room
survey	184	P07	20		Turnraum	negativ	295	extrem heiß + stickig	295	extremely hot + stuffy

source	#src	id	#rid	group	area	quality	#r	original content (german)	#r	content translation (english)
survey	185	P07	21		Turnraum	negativ	296	Dach undicht	296	roof leaky
survey	186	P07	22		Turnraum	negativ	297	Kinder tun sich schwer beim Öffnen der Tür zum Gang	297	children find it difficult to open the door to the hall
survey	187	P07	23		Garten	positiv	298	Der neue Platz ist super!	298	The new place is great!
survey	188	P07	24		Garten	negativ	299	[Wunsch] eine Klettermöglichkeit (Gerüst?!)	299	[request] a climbing option (frame?!)
survey	189	P07	25		Garten	negativ	300	[Wunsch] Schattenmöglichkeiten ?!	300	[request] shadow options!?
survey	190	P07	26		Garten	negativ	301	[Wunsch] ein Unterstellplatz für die Fahrzeuge	301	[request] a covered parking space for vehicles
survey	191	P07	27		Kreativraum	negativ	302	Malraum = Stauraum :-(--> für die Materialien, --> für die einzelnen Gruppen	302	painting room = storage room :-(--> for the materials, --> for the individual groups
survey	192	P08	1	blau	Gruppenraum	positiv	303	Raumgerüst	303	room scaffolding
survey	193	P08	2	blau	Gruppenraum	positiv	304	blauer Teppich, blaue Türe --> blaue Gruppe	304	blue carpet, blue door --> blue group
survey	194	P08	3	blau	Gruppenraum	positiv	305	Nähe zu Turnsaal	305	proximity to gym
survey	195	P08	4	blau	Gruppenraum	negativ	306	Temperaturverhältnisse	306	temperature conditions
survey	196	P08	5	blau	Gruppenraum	negativ	307	"fixer Baubereich"	307	"fixed constructing area"
survey	197	P08	6	blau	Gruppenraum	negativ	308	kann nicht abgedunkelt werden	308	cannot be darkened
survey	198	P08	7	blau	Gruppenraum	negativ	309	Geräusche von Turnsaal stören bei ruhigen Aktivitäten	309	noise from the gym disturbs during quiet activities
survey	199	P08	8		Turnraum	positiv	310	Wesco-Teile	310	Wesco parts
survey	200	P08	9		Turnraum	positiv	311	Möglichkeiten zum Lüften	311	options for airing
survey	201	P08	10		Turnraum	positiv	312	Nähe zu Gruppenraum	312	proximity to group room
survey	202	P08	11		Turnraum	positiv	313	Kreis auf dem Boden	313	circle on the floor
survey	203	P08	12		Turnraum	negativ	314	Temperaturverhältnisse --> sehr warm im Sommer	314	temperature conditions --> very warm in summer
survey	204	P08	13		Turnraum	negativ	315	wenig Geräte (keine Kletterwand / Sprossenwand)	315	few equipment (no climbing wall / wall bars)
survey	205	P08	14		Garten	positiv	316	neues Spielhaus	316	new playing house
survey	206	P08	15		Garten	positiv	317	Sträucher und Beeren	317	bushes and berries
survey	207	P08	16		Garten	positiv	318	Hängesessel	318	hanging chairs
survey	208	P08	17		Garten	positiv	319	Fahrzeuge	319	vehicles
survey	209	P08	18		Garten	positiv	320	Wasserschlauch	320	water hose
survey	210	P08	19		Garten	positiv	321	viel Sandspielzeug	321	a lot of sand toys
survey	211	P08	20		Garten	negativ	322	unübersichtlich / zu klein wenn 100 Kinder draußen sind	322	confusing / too small when 100 children are outside
survey	212	P08	21		Garten	negativ	323	[Wunsch] Parkmöglichkeiten für Fahrzeuge	323	[request] parking options for vehicles
survey	213	P08	22		Personalgarderobe	positiv	324	[Wunsch] eigenes Fach für jede Pädagogin	324	[request] own compartment for every teacher
survey	214	P08	23		Personalgarderobe	negativ	325	zu klein	325	too small
survey	215	P08	24		Personalgarderobe	negativ	326	Entfernung zum Gruppenraum	326	distance to group room
survey	216	P08	25		Personal-WC	positiv	327	Motivationssprüche an der Wand	327	quotes for motivation on the wall
survey	217	P08	26		Personal-WC	negativ	328	Entfernung zu Gruppenraum	328	distance to group room
survey	218	P08	27		Personalraum	positiv	329	Küche gut ausgestattet, Mikrowelle	329	kitchen well equipped, microwave
survey	219	P08	28		Personalraum	negativ	330	Lärmpegel in der Mittagspause	330	noise level during the lunch break
survey	220	P08	29		Personalraum	negativ	331	Temperaturverhältnisse	331	temperature conditions
survey	221	P08	30		Personalraum	negativ	332	zu klein	332	too small
survey	222	P08	31		Essbereich	positiv	333	Hochstühle für jüngere Kinder	333	high chairs for younger children
survey	223	P08	32		Essbereich	negativ	334	zu klein	334	too small
survey	224	P08	33		Kreativraum	positiv	335	viele Materialien	335	many materials
survey	225	P08	34		Kreativraum	positiv	336	Malwand	336	painting wall
survey	226	P08	35		Kreativraum	negativ	337	wenig Stauraum	337	little storage space
survey	227	P08	36		Kreativraum	negativ	338	kein natürliches Licht	338	no natural light
survey	228	P09	1	gelb	Gruppenraum	positiv	339	der vordere Bereich in der Gruppe ist sehr hell (kein Kunstlicht nötig)	339	front area in the group is very bright (no artificial lighting needed)
survey	229	P09	2	gelb	Gruppenraum	negativ	340	der hintere Bereich leider sehr dunkel durch verwinkelte Raumaufteilung (oft Kunstlicht nötig --> dieses ist aber eher unangenehm und grell)	340	the rear area is unfortunately very dark due to the angled room layout (artificial lighting often necessary --> but this is rather unpleasant and glaring)

source	#src	id	#rid	group	area	quality	#r	original content (german)	#r	content translation (english)
survey	230	P09	3		Garten	negativ	341	Dachlawinengefahr im Winter! Oft sind Teile des Gartens gesperrt.	341	Danger of roof avalanches in winter! Parts of the garden are often closed.
survey	231	P09	4		Kreativraum	positiv	342	viele Arbeitsplätze für Kinder	342	many work places for children
survey	232	P09	5		Kreativraum	positiv	343	große Materialvielfalt	343	large variety of materials
survey	233	P09	6		Kreativraum	positiv	344	v.a. im Sommer kühl	344	especially cool in summer
survey	234	P09	7		Kreativraum	negativ	345	Lichtverhältnisse (kein Tageslicht)	345	lighting conditions (no daylight)
survey	235	P09	8		Turnraum	positiv	346	hohe Decke	346	high ceiling
survey	236	P09	9		Turnraum	positiv	347	Zugang zu Terrasse	347	access to terrace
survey	237	P09	10		Turnraum	positiv	348	viel Tageslicht	348	much daylight
survey	238	P09	11		Turnraum	negativ	349	v.a. im Sommer sehr heiß	349	particularly in summer very hot
survey	239	P09	12		Turnraum	negativ	350	Dach ist nicht dicht bei Regen --> Rutschgefahr!	350	roof is leaky when it rains --> danger of slipping!
survey	240	P09	13		Turnraum	negativ	351	schlechte Luft - stickig	351	bad air – stuffy
survey	241	P09	14		Turnraum	negativ	352	keine Klettermöglichkeit (Wand, Sprossenwand, ...)	352	no climbing options (wall, wall bars, …)
survey	242	P09	15		Rhythmikraum	positiv	353	die Größe	353	the size
survey	243	P09	16		Rhythmikraum	positiv	354	vielseitig anwendbar	354	versatile usable
survey	244	P09	17		Rhythmikraum	positiv	355	Zugang auf Terrasse und Garten	355	access to terrace and garden
survey	245	P09	18		Rhythmikraum	positiv	356	Schiebetüren zu Aula (für Veranstaltungen)	356	sliding doors to the hall (for events)
survey	246	P09	19		Rhythmikraum	negativ	357	Aufbewahrungsmöglichkeit für Instrumente	357	storage opportunity for instruments
survey	247	P09	20		Personalraum	positiv	358	Arbeitsfläche für Kinder	358	work surface for children
survey	248	P09	21		Personalraum	negativ	359	Küchenausstattung	359	kitchen equipment
survey	249	P09	22		Personalraum	negativ	360	im Sommer sehr heiß, trotz Sonnenschutz	360	very hot in summer, despite sun protection
survey	250	P09	23		Personalraum	negativ	361	verwinkelter Raum --> wenig Platz	361	angled room --> little space
survey	251	P09	24		Garten	positiv	362	Gruppenzugang über Waschraum, 2 Gruppen (EG) Zugang auch über Terrasse möglich	362	group access via the lavatory, 2 groups (ground floor) access is also possible via terrace
survey	252	P09	25		Garten	positiv	363	Naschgarten für alle Kinder	363	snack garden for all children
survey	253	P09	26		Garten	positiv	364	Spielgeräte	364	playing equipment
survey	254	P09	27		Garten	positiv	365	großer Sonnenschirm über Sandkiste	365	large parasol over sandbox
survey	255	P09	28		Garten	negativ	366	keine Klettermöglichkeit	366	no climbing option
survey	256	P09	29		Garten	negativ	367	wenig Sonnenschutz	367	little sun protection
survey	257	P09	30		Garten	negativ	368	Holzterrasse splittert ab	368	wooden terrace splinters
survey	258	P10	1		Gebäude	negativ	369	teilweise undicht	369	partially leaking
survey	259	P10	2		Rhythmikraum	positiv	370	hell	370	bright
survey	260	P10	3		Rhythmikraum	negativ	371	keine Staumöglichkeiten für Materialen --> wenig	371	no storage opportunities for materials --> little
survey	261	P10	4		Turnraum	positiv	372	hohe Decken	372	high ceiling
survey	262	P10	5		Turnraum	positiv	373	Größe	373	size
survey	263	P10	6		Turnraum	negativ	374	im Sommer sehr heiß	374	very hot in summer
survey	264	P10	7		Turnraum	negativ	375	"Kammerl" nicht besonders groß	375	"little chamber" not very large
survey	265	P10	8		Personalraum	negativ	376	Arbeitsflächen (Waschbecken, …) zu klein + Kühlschrank	376	working surfaces (sink, …) too small + fridge
survey	266	P10	9		Personalraum	negativ	377	Ameisenbefall im Sommer	377	ant attack in summer
survey	267	P10	10		Personalraum	negativ	378	im Sommer sehr heiß	378	very hot in summer
survey	268	P10	11		Personalraum	negativ	379	keine ungestörten Mittagspausen möglich	379	no undisturbed lunch breaks possible
survey	269	P10	12		Kreativraum	positiv	380	Größe	380	size
survey	270	P10	13		Kreativraum	positiv	381	Malwand	381	painting wall
survey	271	P10	14		Kreativraum	negativ	382	[Wunsch] mehr Regale wären schön	382	[request] more shelves would be nice
survey	272	P10	15		Büroraum	positiv	383	zwei Computer	383	two computers
survey	273	P10	16		Büroraum	positiv	384	hell und freundlich	384	bright and friendly
survey	274	P10	17		Büroraum	negativ	385	[Wunsch] Platz zum Vorbereiten wäre sehr angenehm	385	[request] place for preparation would be very pleasant
survey	275	P10	18		Garten	positiv	386	Größe	386	size
survey	276	P10	19		Garten	positiv	387	freie Flächen und Spielgeräte	387	open space and play equipment

source	#src	id	#rid	group	area	quality	#r	original content (german)	#r	content translation (english)
survey	277	P10	20		Garten	positiv	388	etwas abgeschirmt	388	somewhat shielded
survey	278	P10	21		Garten	negativ	389	fast kein Schatten	389	barely any shadow
survey	279	P10	22		Garten	negativ	390	keine Klettermöglichkeit	390	no climbing option
survey	280	P11	1	gelb	Gruppenraum	negativ	391	durch die verwinkelte Aufteilung des Gruppenraums ist der vordere Bereich sehr hell (Fenster) und der hintere Bereich sehr dunkel (Kunstlicht notwendig --> wird als unangenehm empfunden)	391	due to angled layout of the group room, the front area is very bright (windows) and the rear area is very dark (artificial lighting necessary --> is perceived as unpleasant)
survey	281	P11	2		Garten	negativ	392	Dachlawinengefahr im Winter (Garten)	392	danger of roof avalanches in winter (garden)
survey	282	P11	3		Kreativraum	positiv	393	Werktische & Materialwände	393	work tables & material walls
survey	283	P11	4		Kreativraum	positiv	394	das Material ist griffbereit	394	the material is at hand
survey	284	P11	5		Kreativraum	positiv	395	im Sommer ist es angenehm kühl	395	in summer it is pleasantly cool
survey	285	P11	6		Kreativraum	negativ	396	die Lichtverhältnisse --> kein Tageslicht (Keller)	396	the lighting conditions --> no daylight (basement)
survey	286	P11	7		Kreativraum	negativ	397	es ist ein "Durchgangsraum"	397	it is a "passage room"
survey	287	P11	8		Turnraum	positiv	398	hohe Decke	398	high ceiling
survey	288	P11	9		Turnraum	positiv	399	Ausgang auf eine Terrasse	399	exit to a terrace
survey	289	P11	10		Turnraum	positiv	400	viel Tageslicht	400	much daylight
survey	290	P11	11		Turnraum	negativ	401	im Sommer ist es sehr heiß (trotz Sonnenschutz)	401	in summer it is very hot (despite sun protection)
survey	291	P11	12		Turnraum	negativ	402	wenn es regnet, tropft es von der Decke	402	when it rains, it drips from the ceiling
survey	292	P11	13		Turnraum	negativ	403	es ist oft stickig	403	it is often stuffy
survey	293	P11	14		Turnraum	negativ	404	es gibt keine Klettermöglichkeiten für die Kinder	404	there are no climbing options for children
survey	294	P11	15		Rhythmikraum	positiv	405	die Größe	405	the size
survey	295	P11	16		Rhythmikraum	positiv	406	vielseitig anwendbar	406	versatile usable
survey	296	P11	17		Rhythmikraum	positiv	407	Zugang zur Terrasse & Garten	407	access to the terrace & garden
survey	297	P11	18		Rhythmikraum	positiv	408	Schiebetüren zur Aula (für Veranstaltungen)	408	sliding doors to the hall (for events)
survey	298	P11	19		Rhythmikraum	negativ	409	die Aufbewahrung der Instrumente	409	the storage for instruments
survey	299	P11	20		Personalraum	positiv	410	Arbeitsfläche für Kinder	410	work surface for children
survey	300	P11	21		Personalraum	negativ	411	Küchenausstattung (Geräte)	411	kitchen equipment (appliances)
survey	301	P11	22		Personalraum	negativ	412	sehr heiß im Sommer	412	very hot in summer
survey	302	P11	23		Personalraum	negativ	413	verwinkelter Raum	413	angled room
survey	303	P11	24		Garten	positiv	414	Gruppenzugänge über die Waschräume (EG + OG)	414	group accesses via lavatories (ground floor + upper floor)
survey	304	P11	25		Garten	positiv	415	Naschgarten	415	snack garden
survey	305	P11	26		Garten	positiv	416	Geräte zum Spielen	416	equipment for playing
survey	306	P11	27		Garten	positiv	417	Beschattung bei der Sandkiste	417	shading at the sandbox
survey	307	P11	28		Garten	negativ	418	keine Klettermöglichkeit	418	no climbing option
survey	308	P11	29		Garten	negativ	419	kaum Sonnenschutz	419	hardly any sun protection
survey	309	P11	30		Garten	negativ	420	Holzterrasse splittert ab	420	wooden terrace splinters
