# Supplementary material for: Uncovering placemaking needs with(in) a kindergarten community: a cross-disciplinary approach to participatory design
Source: Front Psychol. 2023 Jun 20;14:1126276. doi: 10.3389/fpsyg.2023.1126276 (PMC10319412; doi:10.3389/fpsyg.2023.1126276)
Supplement: Supplementary Data Sheet S7 — Thematic analysis of cultural fiction probes. [file Data_Sheet_7.pdf]

Cultural Fiction Probes Thematic Analysis

#	Probe #	Group	Return no.	Probe data	Code	Themes	Meta theme expressions
1	1	Group A	1	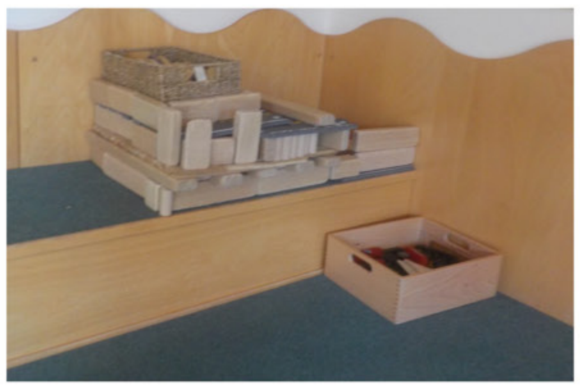	Play corner of different heights, with construction toys	Temporal structure, structure creation	Place-making, Control of the environment, time making, spatial qualities
2	1	Group A	3	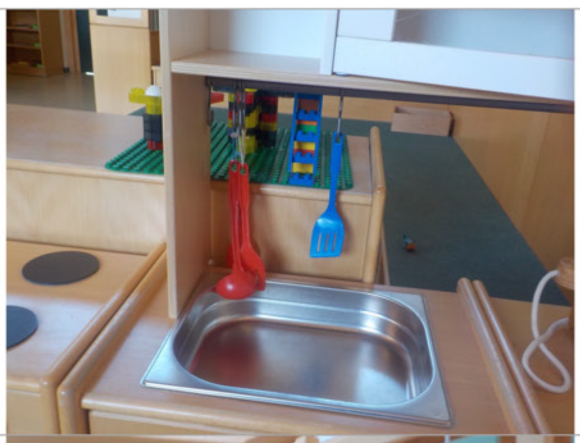	pretend play corner resembling a kitchen	Pretend play space	Place making
3	1	Group A	12	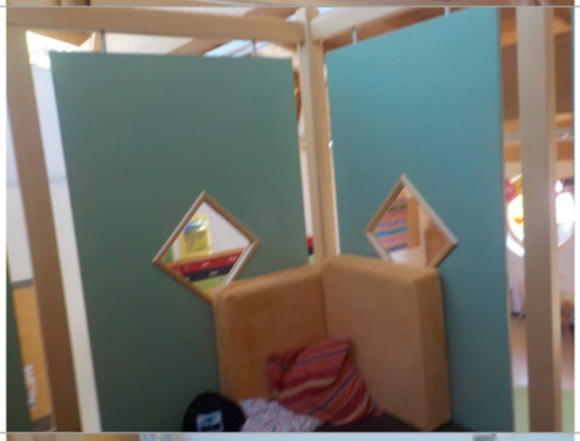	area for rest and refuge on a higher level	Ready made- Refuge	Place making
4	1	Group A	13	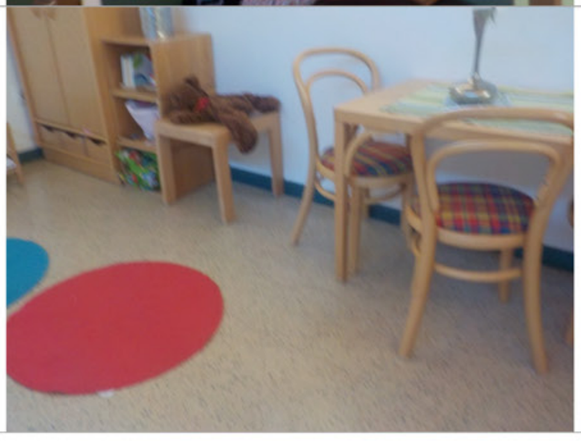	pretend play corner resembling a dining room	Pretend play space	Place making
5	1	Group A	9	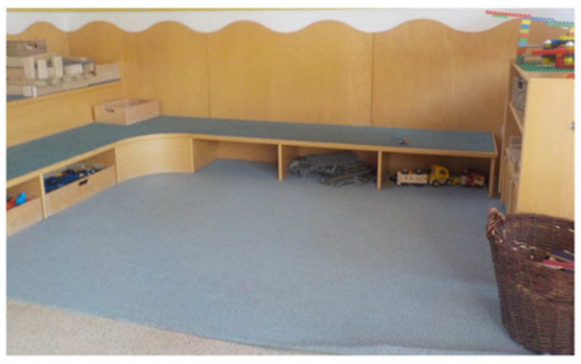	Play corner of different heights, with construction toys	Temporal structure, structure creation	Place-making, Control of the environment, time making, spatial qualities
6	1	Group A	17	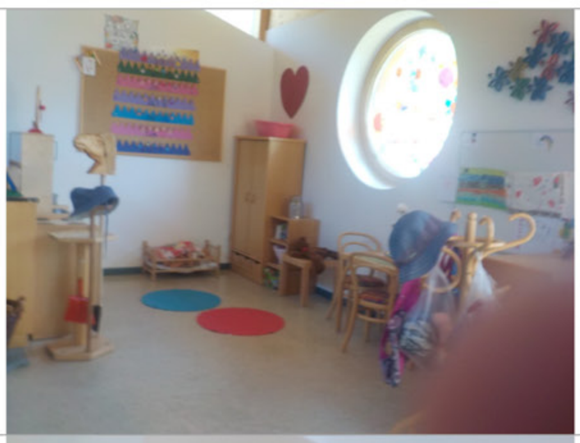	pretend play corner resembling a domestic environment, home	Pretend play space	Place making
7	1	Group A	19	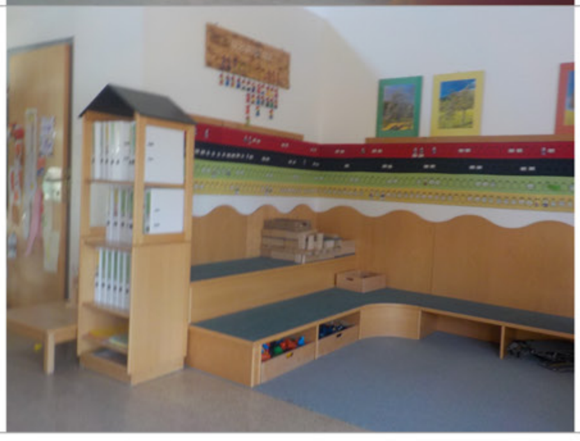	Play corner of different heights, with construction toys	Temporal structure, structure creation	Place-making, Control of the environment, time making, spatial qualities

#	Probe #	Group	Return no.	Probe data	Code	Themes	Meta theme expression s
8	1	Group A	20	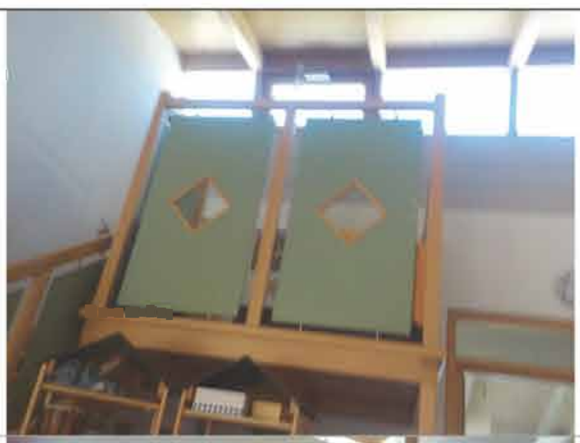	area for rest and refuge on a higher level taken from lower level	Ready made-Refuge	Place making, spatial qualities
9	1	Group A	25	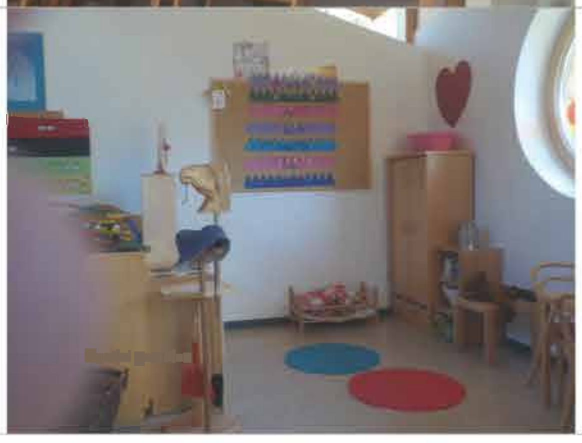	pretend play corner resembling a domestic environment, home	Pretend play space	Place making
10	1	Group A	29	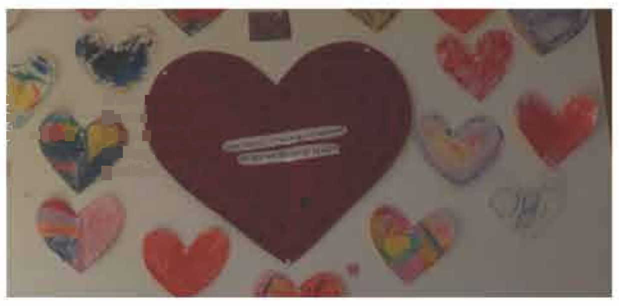	Wall showing affection and community, sociality	Sociality	Place making
11	1	Group A	36	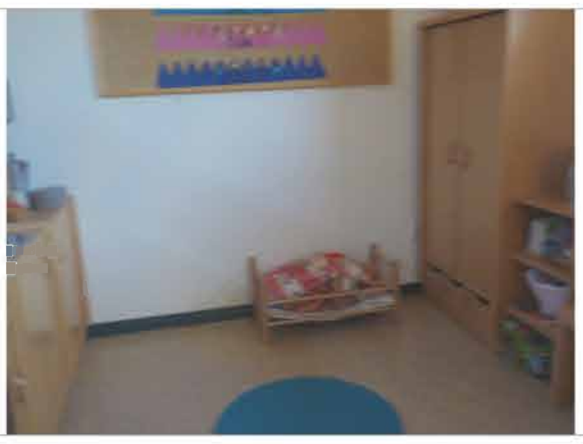	pretend play area wardrobe and dolls in a bed	Pretend play space	Place making
12	1	Group A	45	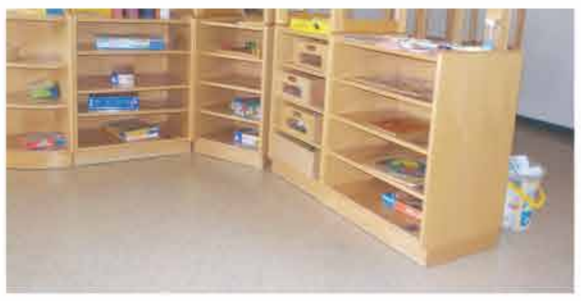	Shelves system with toys and music instruments		Spatial qualities
13	1	Group A	54	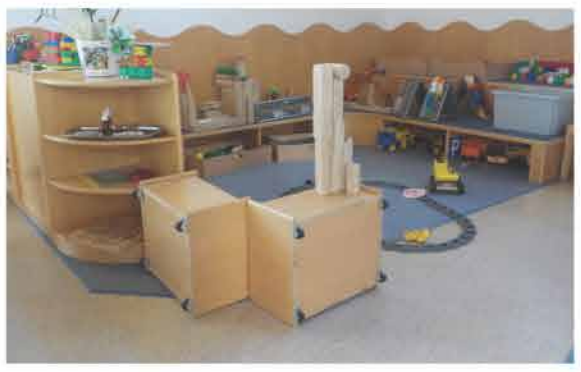	Play area, shielded	Temporal structure, structure creation	Place-making, Control of the environment, time making, spatial qualities
14	1	Group A	55	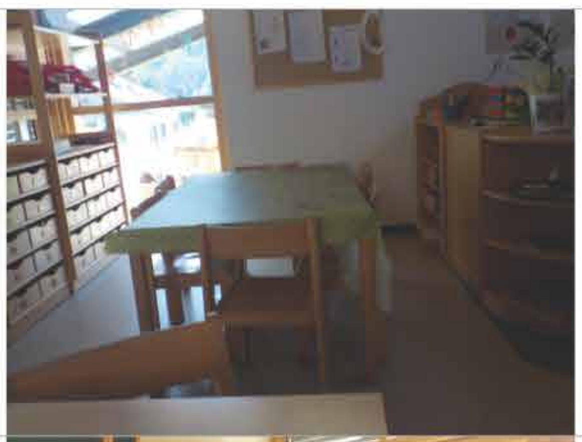	Group desk with chairs	Sitting in groups	Spatial qualities
15	1	Group A	57	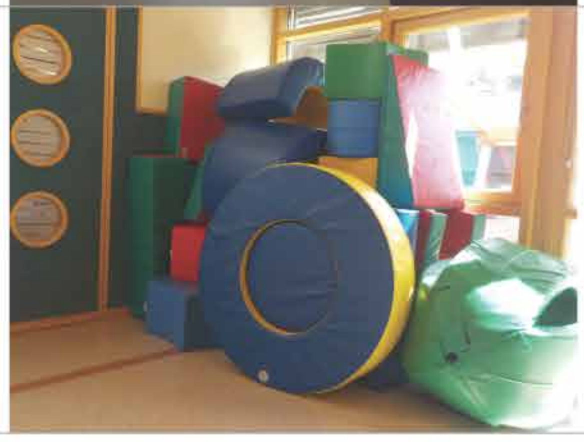	Large building elements for constructing structures	Temporal structure, structure creation, control of space	Place-making, Control of the environment, time making, spatial qualities

#	Probe #	Group	Return no.	Probe data	Code	Themes	Meta theme expressions
16	1	Group A	60	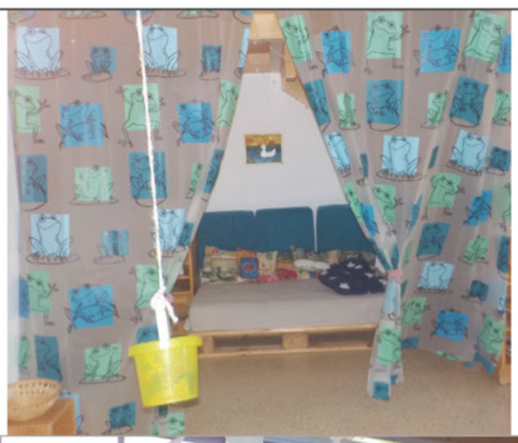	Floor Refuge with pillows that can be closed off using curtains	Ready made-Refuge	Place-making
17	1	Group A	63	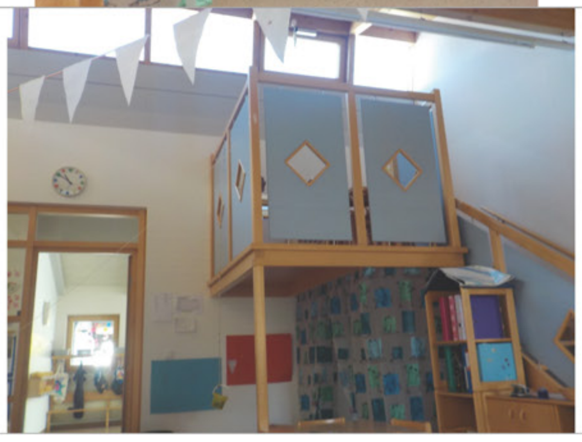	Refuge on another level with a lower one underneath	Ready made-Refuge	Place-making
18	1	Group A	66	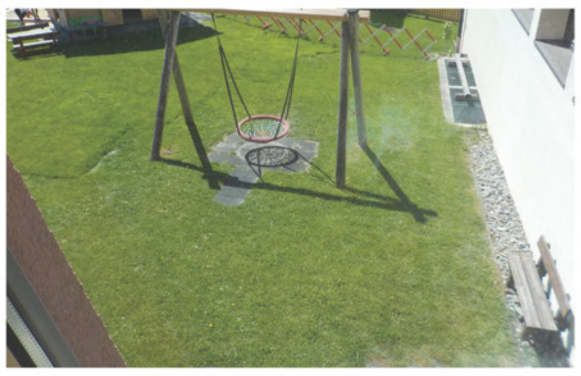	Outdoor playground swing structure	Outdoor play	Place-making
19	1	Group A	71	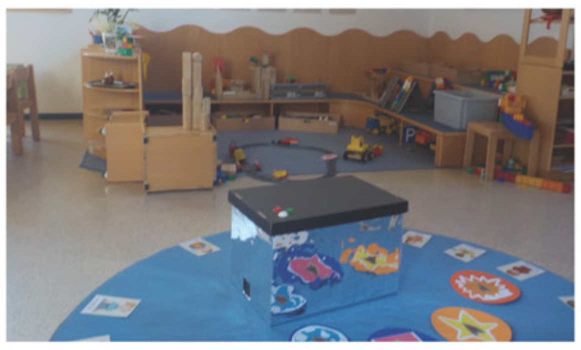	Round area for gathering and play area in the background	Sitting in groups, play, temporal structure	Place-making, Control of the environment, time making, spatial qualities
20	1	Group B	3	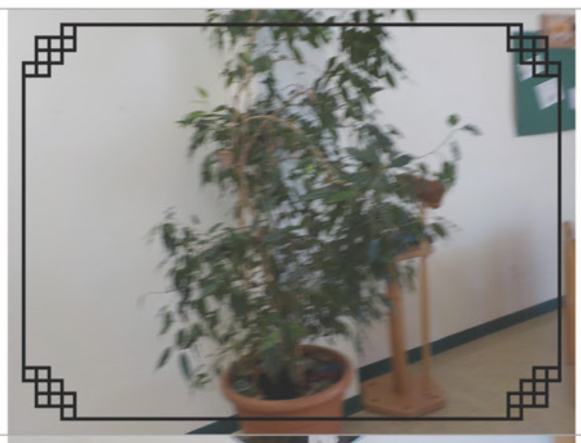	Natural element	Natural element indoors	Place-making
21	1	Group B	5	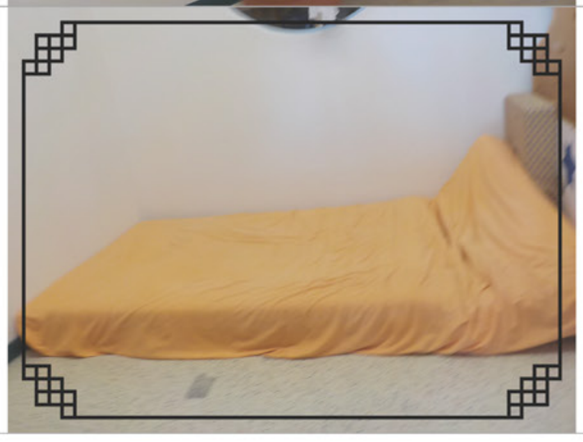	Resting area with a mattress, exposed	Ready made-Refuge	Place-making
22	1	Group B	6	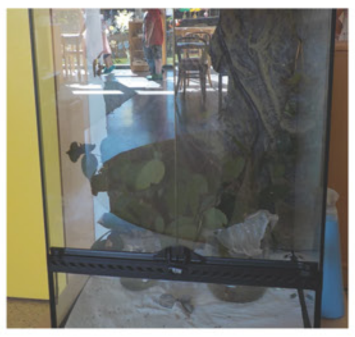	Large plant terrarium	Natural element indoors	Place-making
23	1	Group B	9	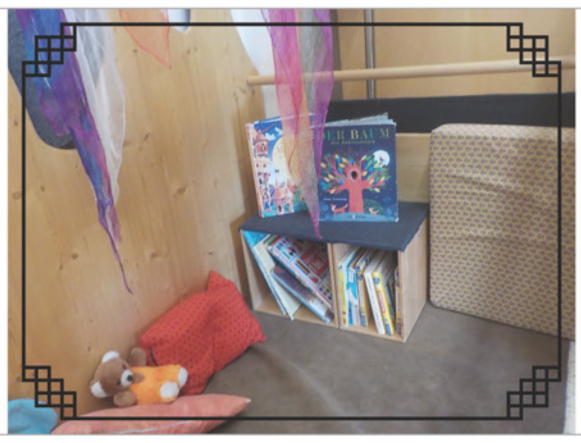	Reading corner in a resting area with pillows	Ready made-Refuge, reading	Place-making

#	Probe #	Group	Return no.	Probe data	Code	Themes	Meta theme expressions
24	1	Group B	10	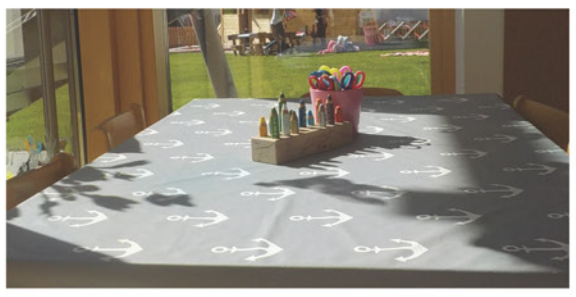	Working area desk overlooking the garden	Sitting in groups, sociality	Spatial qualities
25	1	Group B	11	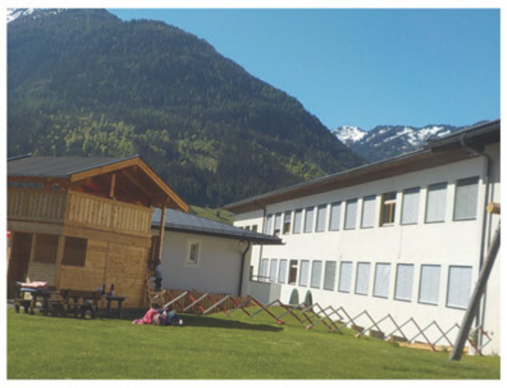	Garden playhouse, view to the mountains	Outdoor play, nature	Place making
26	1	Group B	12	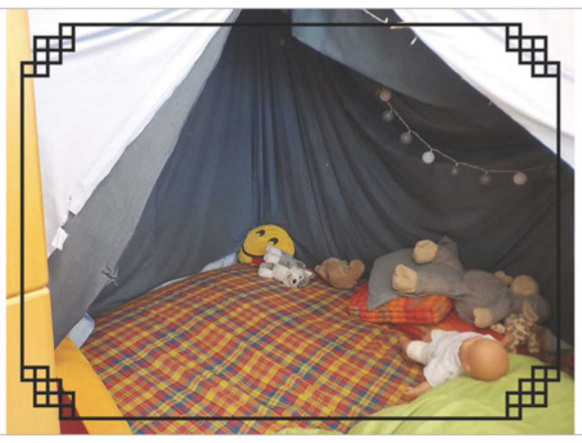	Resting area in tent form with pillows, fabrics, light, dolls and plush toys	Ready made-Refuge	Place making
27	1	Group B	19	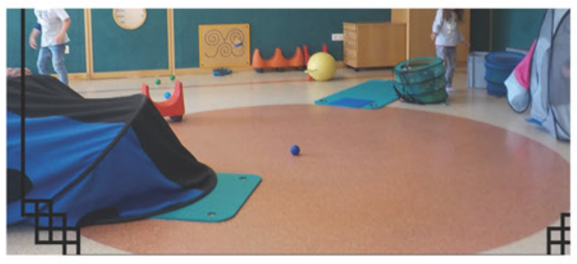	Fabric based tent like structures in an open area	Self-made structures, structure creation	Place-making, Control of the environment, time making, spatial qualities
28	1	Group B	20	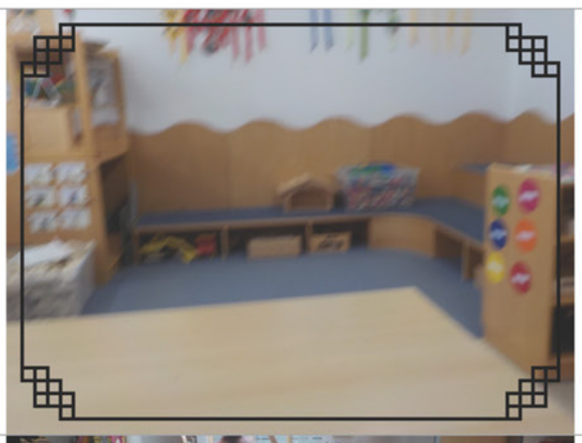	Play corner of different heights, with construction toys	Temporal structure, structure creation	Control of the environment, time making, spatial qualities
29	1	Group B	21	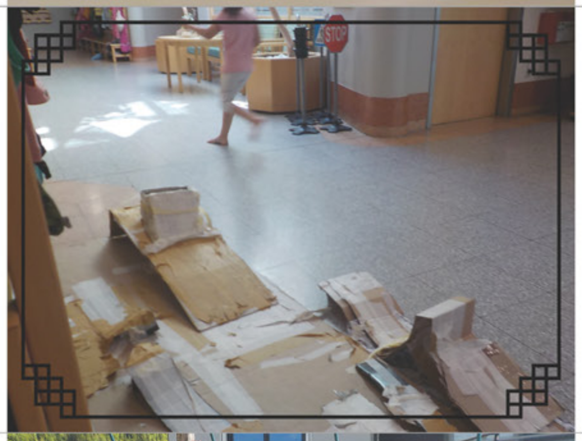	Corridor with cardboard floor constructions	Temporal structure, structure creation, control of space	Place-making, Control of the environment, time making, spatial qualities
30	1	Group B	2	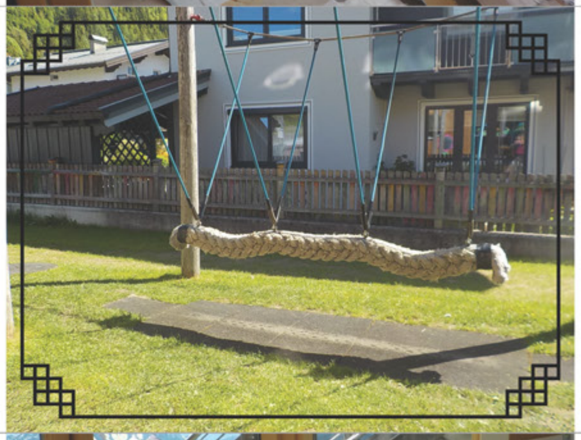	Active Playground equipment in outdoor area	Outdoor play	Control of the environment, time making, spatial qualities
31	1	Group C	1	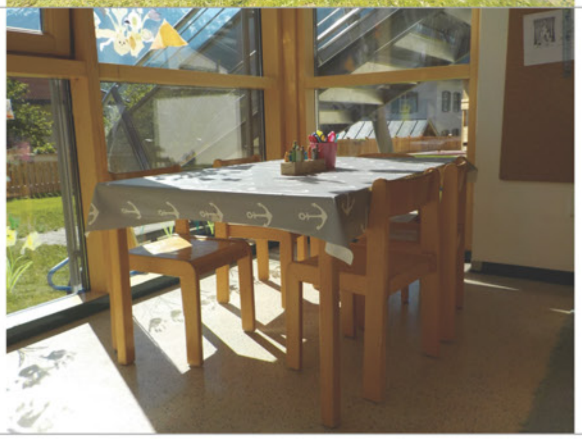	Sunny, Working area desk overlooking the garden	sociality, sitting in groups	spatial qualities

#	Probe #	Group	Return no.	Probe data	Code	Themes	Meta theme expressions
32	1	Group C	6	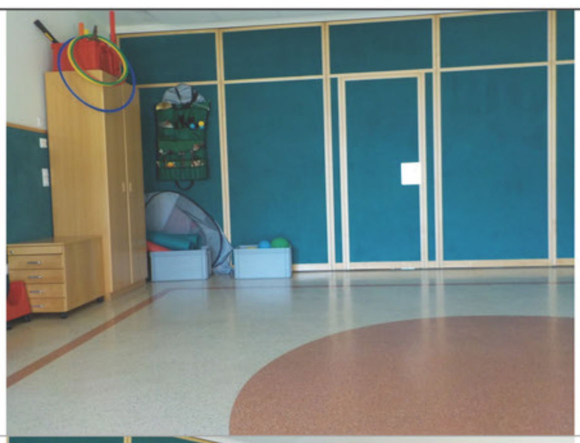	Open area with gym equipment	Open space	Control of the environment, time making, spatial qualities
33	1	Group C	7	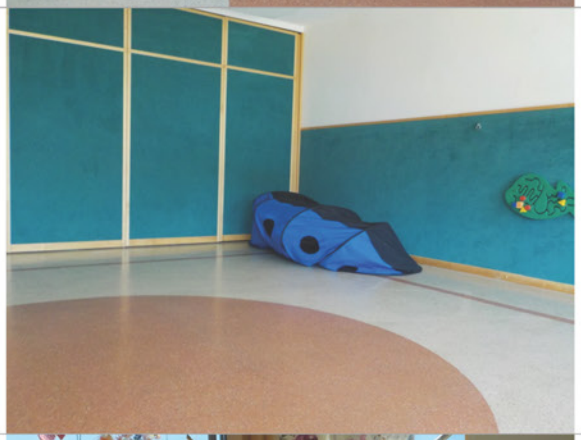	Open area with tent structure	structure creation, open space, self-made refuge	Place-making, Control of the environment, time making, spatial qualities
34	1	Group C	12	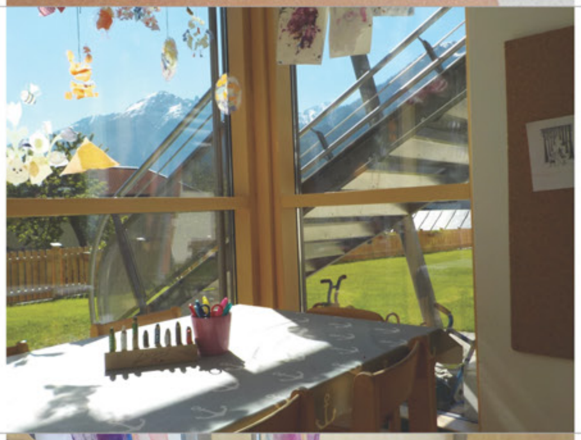	Sunny, Working area desk overlooking the garden	Sitting in groups, creativity	spatial qualities
35	1	Group C	13	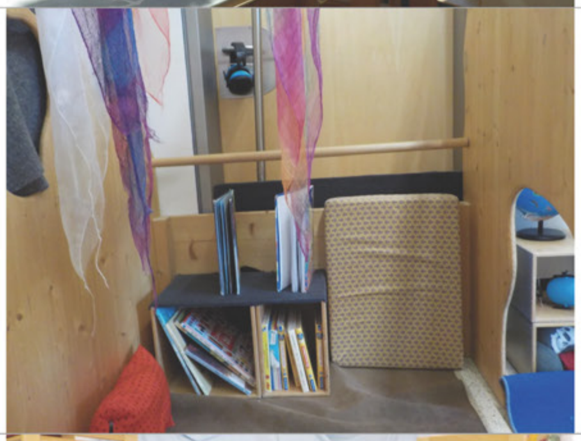	Resting area with pillows and books	Ready made-Refuge, reading	Place making, spatial qualities
36	1	Group C	37	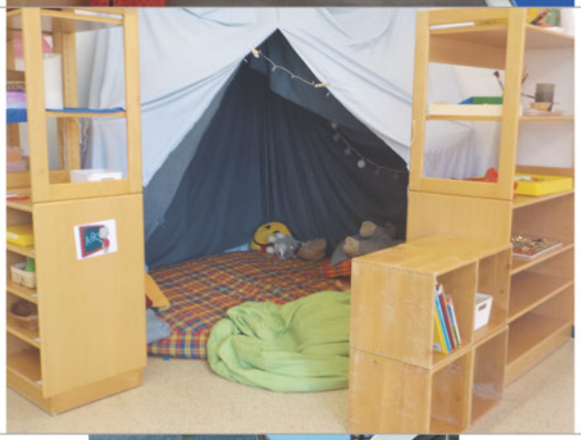	resting area in tent form with pillows, fabrics, light, dolls and plush toys, Closed off using shelves	Ready made-Refuge	Place making, spatial qualities
37	1	Group C	39	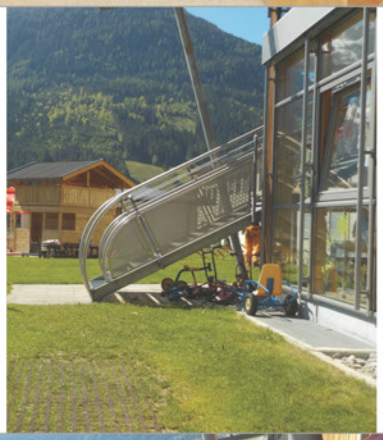	Outdoors playground	Outdoor play, nature	spatial qualities
38	1	Group C	43	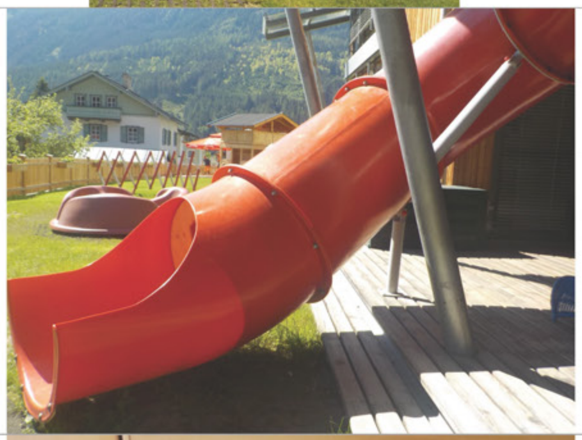	Slide, playground equipment	Outdoor play	spatial qualities
39	1	Group D	5	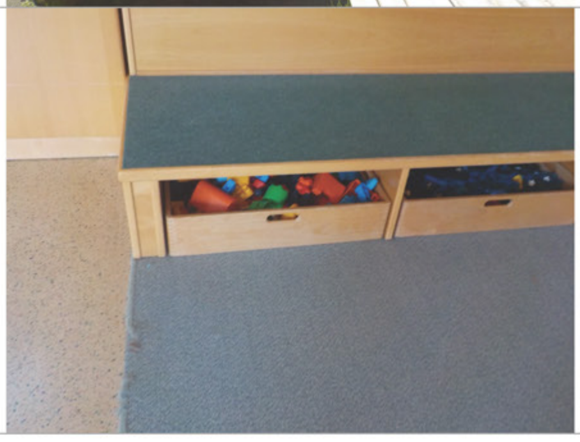	Play area with stored toys in drawers	Temporal structure, structure creation, organisation	Spatial qualities, time making

#	Probe #	Group	Return no.	Probe data	Code	Themes	Meta theme expressions
40	1	Group D	7	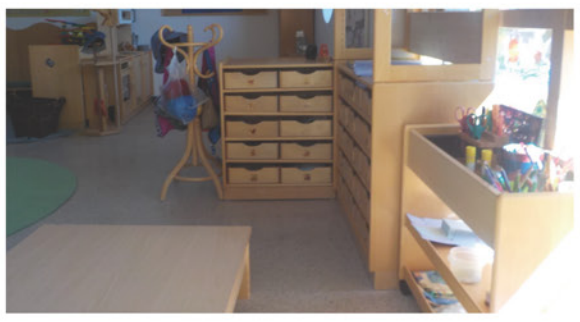	Room with craft material in the foreground and drawers	Creativity corner	spatial qualities
41	1	Group D	9	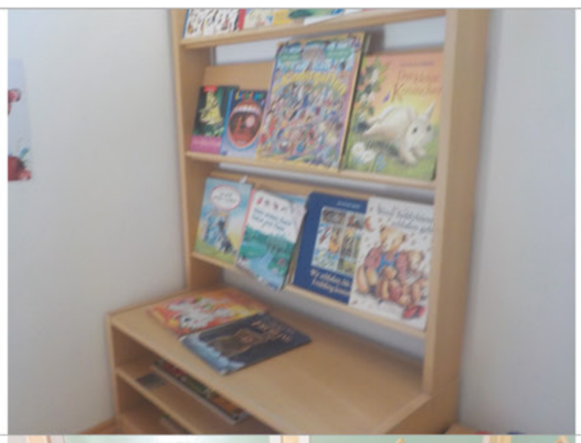	Reading corner with books and shelves	Reading	spatial qualities
42	1	Group D	10	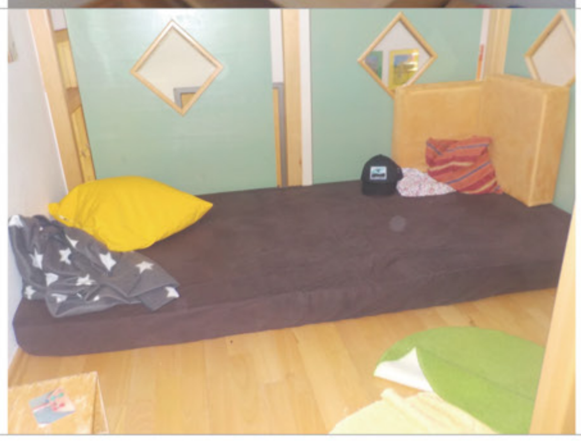	Resting corner with pillows	Ready made-Refuge	Place making
43	1	Group D	12	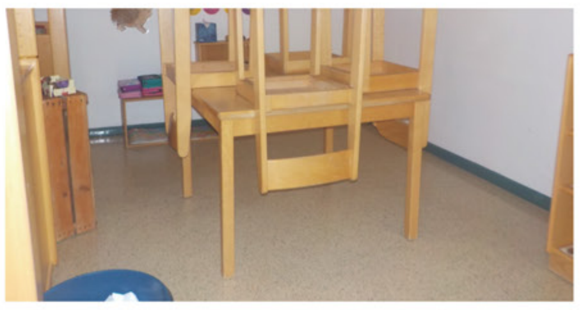	Working table with chairs	Sitting in groups	Control of the environment, time making, spatial qualities
44	1	Group D	13	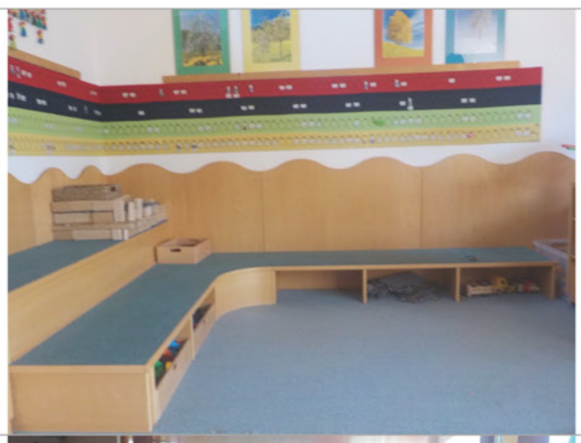	Round area for gathering and play area in the background	Temporal structure, structure creation, organisation	Control of the environment, time making, spatial qualities
45	1	Group D	21	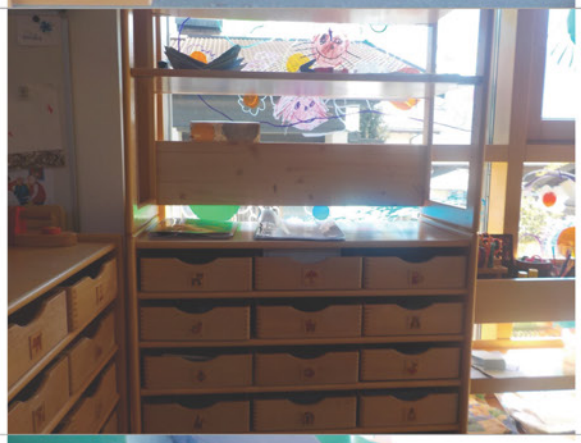	Shelf unit with drawers with different themes	Organisation, creativity	Physical qualities of space
46	1	Group D	22	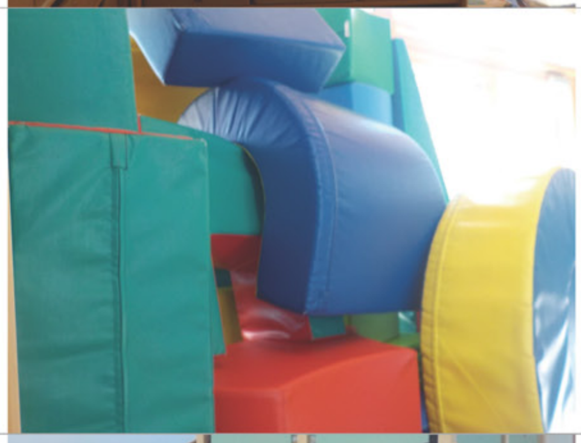	Structural Building equipment	Temporal structure, structure creation, control of space	Place making, Control of the environment
47	1	Group D	25	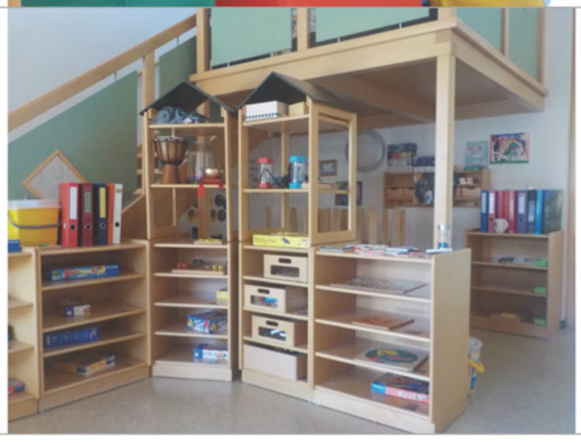	Shelves system with toys and music instruments	organisation	Physical qualities of space

#	Probe #	Group	Return no.	Probe data	Code	Themes	Meta theme expression s
48	1	Group D	27	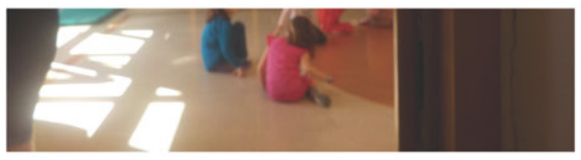	Children sitting in a circle in an open room area	sociality, sitting in groups	Control of the environment, time making, spatial qualities, place making
49	1	Group D	38	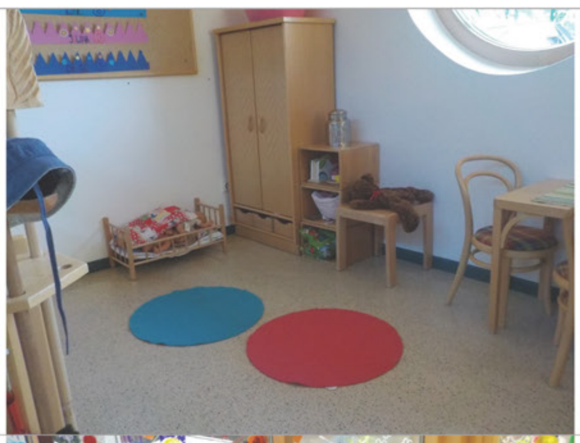	pretend play corner resembling a domestic environment, home	Pretend play space	spatial qualities
50	1	Group D	42	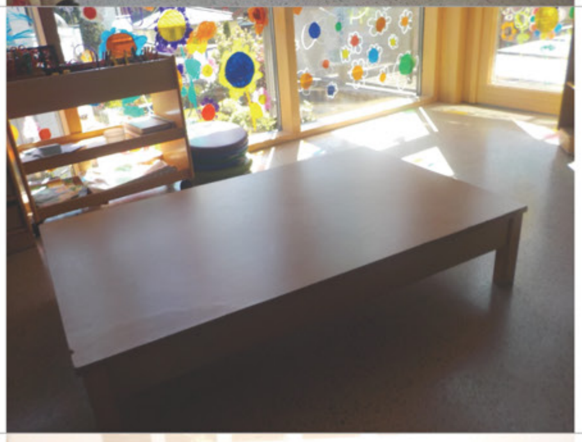	Short table for craft activities in the foreground, windows with drawings on them	Creativity, scale	Control of the environment, spatial qualities
51	1	Group D	51	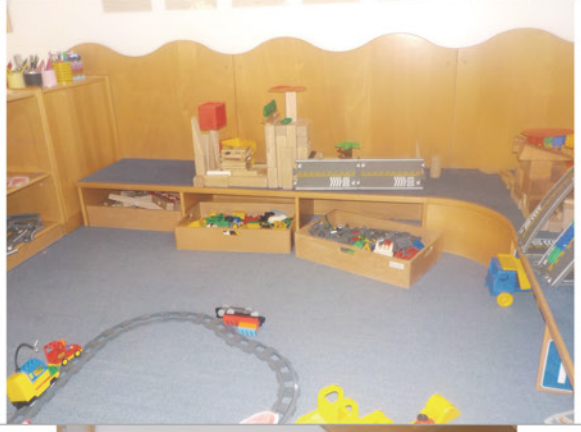	Play area with built structures using building blocks and a train track	Temporal structure, structure creation	Control of the environment, time making, spatial qualities
52	1	Group D	52	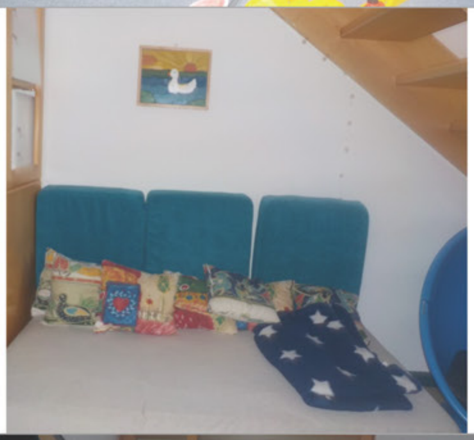	Resting area under stairs with pillows, not isolated	Ready made- Refuge	Spatial qualities
53	1	Group D	54	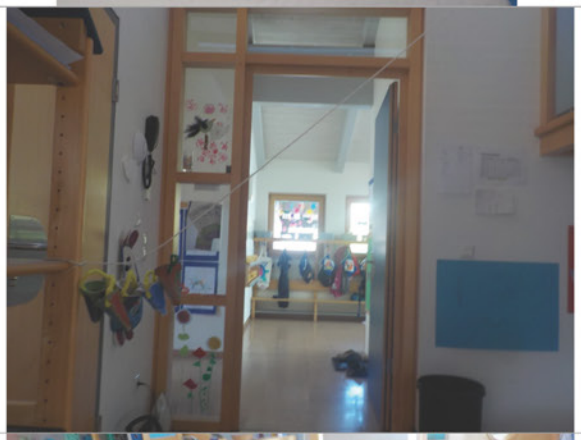	Created toy that connects two levels in the same room using a rope	Control of space	Control of the environment
54	1	Group D	55	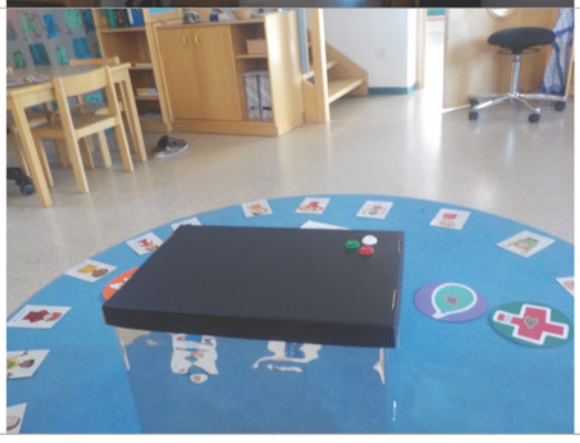	Communicator box with circle around it for sitting in a group	Sitting in groups, sociality	Spatial qualities, time making
55	1	Group D	56	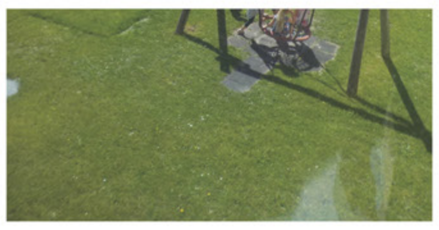	Outdoor playground swing with children playing	Outdoor play, nature	Place making, spatial qualities

#	Probe #	Group	Return no.	Probe data	Code	Themes	Meta theme expressions
56	1	Group D	59	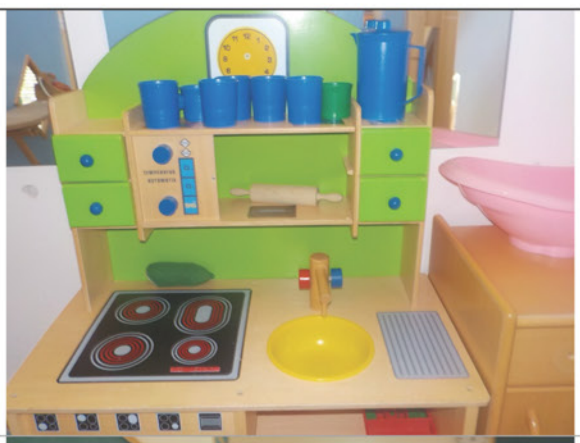	pretend play corner resembling a kitchen	Pretend play space	Place making, spatial qualities, control of the environment
57	1	Group D	61	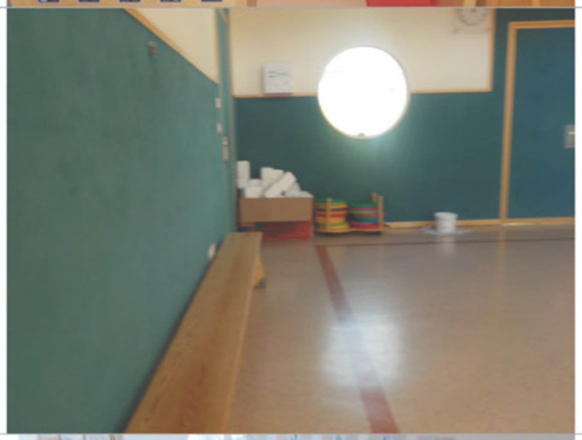	Open area with gym equipment and bench	Open space, control of space	Place making, spatial qualities
58	1	Group D	62	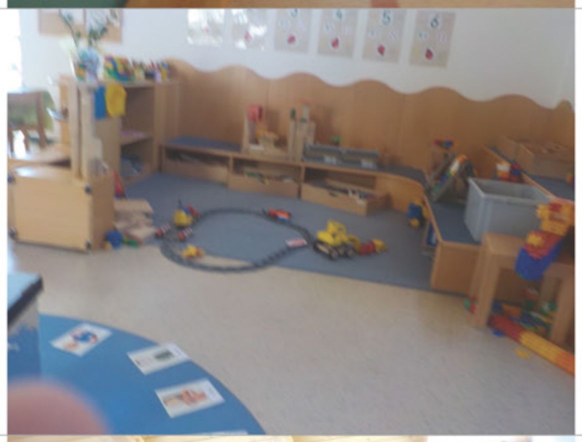	Play area with toys placed on the floor	Temporal structure, structure creation	Control of the environment, time making, spatial qualities
59	1	Group D	63	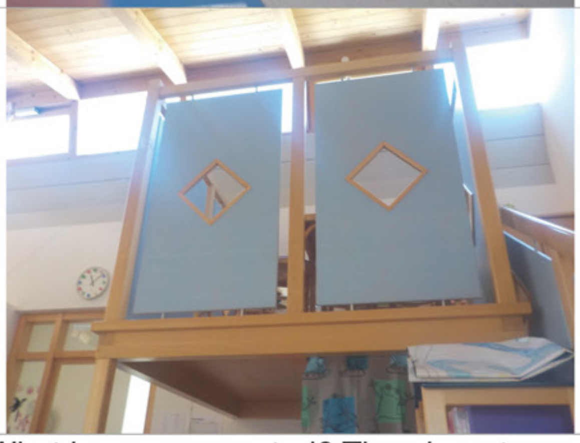	area for rest and refuge on a higher level taken from lower level	Ready made-Refuge	ontrol of the environment
60	3	Group D	2	What have you created? There's a straw with a sheet of paper and I glued it shut, and if it's too hot for you, you can blow into the straw and it will cool you down again. What can it do? It's only for the summer.	A straw device for making it cooler when it is warm	Control of temperature	Control of the environment
61	5A	A.1	1	Child: I make music	Music	Music	Control of the environment
62	5A	A.1	2	K1: I like the drum.	Drum	Music	Control of the environment
63	5A	A.1	3	K2: I like the singing bowl.	Music bowl	Music	Control of the environment
64	5A	A.1	4	K3: I like xylophone.	Xylophone	Music	Control of the environment
65	5A	A.1	5	K4: I like scissor sounds.	Scissors	Craft sounds	Control of the environment
66	5A	A.1	6	K: I don't like the car sounds.	Cars	Mechanic sounds	Control of the environment
67	5A	A.1	7	K1: I don't like the telephone sound.	Telephone	Technology sounds	Control of the environment
68	5A	A.1	8	K1: I like a song... La la la... Rock song, a rock song (sings English words into the microphone several times)	Singing	Singing	Control of the environment
69	5A	A.1	9	K2: I like (a hammering sound is heard)	Rhythmic sound	Music	Control of the environment
70	5A	A.1	10	K3: I like (you hear "fart" sounds, the word "dog shit," and subsequent laughter)	Flatulence	Humour	Control of the environment
71	5A	A.2	1	K1: I don't like anything loud.	High volume	Noise	Control of the environment
72	5A	A.2	2	K2: I like the drum.	Drum	Music	Control of the environment
73	5A	A.2	3	K3: I like the singing bowl.	Music bowl	Music	Control of the environment
74	5A	A.2	4	K4: I don't like any...not that loud.	Low volume	Quiet	Control of the environment
75	5A	A.2	5	K5: I like scissor sounds.	Scissors	Craft sounds	Control of the environment
76	5A	A.2	6	K6: Yes, I also like sounds.	Sounds		
77	5A	A.2	7	K7: I like scissors sounds.	Scissors	Craft sounds	Control of the environment
78	5A	A.2	8	K8: I like (you can hear (probably) scissors).	Scissors	Craft sounds	Control of the environment
79	5A	A.2	9	K9: I also like scissor sounds.	Scissors	Craft sounds	Control of the environment
80	5A	A.2	10	K10: Not both the same thing Doris said!	Different preference	Disagreement	Control of the environment
81	5A	A.2	11	K11: I like the bell.	Bell sounds	Music	Control of the environment
82	5A	A.2	12	K12: I like (makes a noise)	sound		

#	Probe #	Group	Return no.	Probe data	Code	Themes	Meta theme expressions
83	5A	A.2	13	K13: But I like very quiet.	Very quiet	Quiet	Control of the environment
84	5A	A.2	14	K14: I don't like the telephone noise.	Telephone	Technology sounds	Control of the environment
85	5A	A.2	15	K15: I like clapping (claps).	Clapping	Human sounds	Control of the environment
86	5A	A.2	16	K16: We can make a plug noise too. (makes a rattling sound)	Plug	Technology sounds	Control of the environment
87	5A	A.2	17	K17: That's a good sound.	Rattling	Control of the environment	Control of the environment
88	5A	A.2	18	K18: I like (you can hear (probably) ironing beads).	Ironing beads	Craft sounds	Control of the environment
89	5A	A.2	19	K19: I like (makes a noise).			
90	5A	A.2	20	K20: I like (makes a noise).			
91	5A	A.2	1	K1: Clapping (claps).	Clapping	Human sounds	Control of the environment
92	5A	A.2	2	K2: Uh, you crack(??), I ???. It's a good idea. So cracking is like that. Now you show! I like to crack (someone claps) Yeah, right. See ???, that's how it goes... ??? (someone plays xylophone)	Clapping	Human sounds	Control of the environment
93	5A	A.3	1	K1: I love... I don't like dog noises.	Dog sounds	Animal sounds	Control of the environment
94	5A	A.3	2	K2: I don't like cat sounds.	Cat sounds	Animal sounds	Control of the environment
95	5A	A.3	3	K3: Yeah, we love that too. (begins to sing)	Painting noise	Craft sounds	Control of the environment
96	5A	A.3	4	K4: I like car noises.	Car noises	Mechanic sounds	Control of the environment
97	5A	A.3	5	K5: I like car noises too.	Car noises	Mechanic sounds	Control of the environment
98	5A	A.3	6	K6: I like painting noises.	Painting sounds	Craft sounds	Control of the environment
99	5A	A.3	7	K7: I like... I like painting sounds.	Painting sounds	Craft sounds	Control of the environment
100	5A	A.3	8	K8: I like... story sounds and... and all the sounds I hear.	Story sounds	Human sounds	Control of the environment
101	5A	A.3	9	K1: I like music.	Music	Music	Control of the environment
102	5A	A.3	10	*K: in the background: "I like music."*	Music	Music	Control of the environment
103	5A	A.3	11	K2: I don't like anything loud	Not loud	Quiet	Control of the environment
104	5A	A.3	12	you hear different noises (e.g. from a pair of scissors???)	Scissors	Craft sounds	Control of the environment
105	5A	A.3	13	one hears hammering sounds	Hammering	Music	Control of the environment
106	5A	A.4	1	child sings softly	Singing	Singing	Control of the environment
107	5A	A.4	2	K1: I like cat...cat sounds.	Cat sounds	Animal sounds	Control of the environment
108	5A	A.4	3	K2: I like a horse.	Horse	Animal sounds	Control of the environment
109	5A	A.4	4	K3: I like horse sounds.	Horse sounds	Animal sounds	Control of the environment
110	5A	A.4	5	K4: I like horse sounds.	Horse sounds	Animal sounds	Control of the environment
111	5A	A.4	6	*K1 says several times that it has already recorded*			
112	5A	A.4	7	K2: I like clapping (claps)	Clapping	Human sounds	Control of the environment
113	5A	A.4	8	*you can hear many different sounds (hammering, building blocks?, croaking)*	Hammering	Music	Control of the environment
114	5A	A.5	1	K1: I like raven, games and this is really good.	Ravens and toys	Playing sounds	Control of the environment
115	5A	A.5	2	K2: I like to play games.	Games	Playing sounds	Control of the environment
116	5A	A.5	3	K3: I like car noises.	Car noises	Mechanic sounds	Control of the environment
117	5A	A.5	4	K4: I like painting sounds. I like car noises. I like finger noises and kneading noises. And people sounds. All the sounds I like.	painting sounds, car sounds, finger noises, kneading noises, people sounds. All kinds	Mechanic sounds, craft sounds, human sounds	Control of the environment
118	5A	A.5	5	*K. in the background: All the sounds I hear.*	All sounds	All sounds	Control of the environment
119	5A	A.5	6	*K5 sings about ironing beads, among other things*	Singing	Singing	Control of the environment
120	5A	A.5	7	*K. in the background: "I like music."*	Music	Music	Control of the environment
121	5A	A.5	8	K1: I like music.	Music	Music	Control of the environment
122	5A	A.5	9	K2: I don't like anything loud	Not loud	Quiet	Control of the environment
123	5A	A.5	10	K3: I like the drum	Drum	Music	Control of the environment
124	5A	A.5	11	K4: I like the singing bowl	Music bowl	Music	Control of the environment
125	5A	A.5	12	K1:I like scissors sounds. (One can hear scissors)	Scissors	Craft sounds	Control of the environment
126	5A	A.5	13	K: I like the magnet sounds.	Magnet sounds	Craft sounds	Control of the environment
127	5A	A.5	14	K: I like the cars.	Cars	Mechanic sounds	Control of the environment
128	5A	A.5	15	K: I don't like sometimes... (implies shouting). the rest is not understandable	Not shouting	Quiet	Control of the environment

#	Probe #	Group	Return no.	Probe data	Code	Themes	Meta theme expressions
129	5A	B.1	1	K1: I don't like the sound (makes a clanging noise).	Clanging	Noise	Control of the environment
130	5A	B.1	2	K2: And I don't like the sound of that. (knocks on something) I don't like that.	Knocking	Noise	Control of the environment
131	5A	B.1	3	K3: I don't like the sound (it is not clear which sound is meant).	Unknown		
132	5A	B.1	4	K4: Squeak, squeak, squeak...	Squeaking		
133	5A	B.1	5	K: Quack???K: Quack, quack, quack. In the reading corner.	Reading corner		Control of the environment
134	5A	B.1	6	K: Elsa-Music.	Film music	Music	Control of the environment
135	5A	B.2	1	K1: And I don't like the sound. (makes a noise) I don't like to listen to that.	Unknown		
136	5A	B.2	2	*several children shout into the microphone; then there is probably sawing and hammering*	Unknown	Unknown	
137	5A	B.2	3	K: Okay. ... I like it when it's quiet.I like reading in the reading corner.	Quiet at reading corner	Quiet	Control of the environment
138	5A	B.2	4	K: I like it in the reading corner and it's loud there?<br>P: What noises do you make in the reading corner?<br>K: Loud.<br>P: Loud noises? K: Okay. ... Do you want to record a ??? sound?<br>K: Uh, yeah I always want to be alone ??? At home.	Loud, Be alone	Noise	Control of the environment
139	5A	B.2	5	P: Are there any other sounds that you think are good?<br>K1: Quiet.	Quiet	Quiet	Control of the environment
140	5A	B.2	6	K: The closet is quiet.<br>P: You like it best when it's quiet?<br>K: Yes.<br>P: And this is a little chamber?<br>K: Yes.	Quiet	Quiet	Control of the environment
141	5A	B.2	7	P: What sounds would you like to record for the children of Harkarosh?<br>K: Uh, the ones I don't like very much.<br>P: The ones you don't like well? And what sounds are those?<br>K. shouting<br>P: Screaming?<br>K: Yes. I don't like that.P: And you don't like it so much when it's so loud in the gym?<br>K: No.	Shouting in gym	Noise	Control of the environment
142	5A	B.3	1	P: Scream really loud!<br>K. screams	Unknown	Unknown	
143	5A	B.3	2	Can you say what sound that is that you recorded?<br>K: Screaming and loud.<br>P: And is that good for your ears?<br>K: Not.<br>P: Not good.	Loud, screaming	Noise	Control of the environment
144	5A	B.3	3	*K. drums.*<br>*P: Do you like the drumming sound?*<br>*K: Yes.*	Drum	Music	Control of the environment
145	5A	B.3	4	K: Singing.<br>P: You like it when someone sings?<br>K: I already...<br>P: Did you say it anyway?<br>K: Mhm.	Singing	Singing	Control of the environment
146	5A	B.4	1	*K. whispers Hello, my name is *********. makes breathing and blowing sounds and strikes a triangle*	Breathing, blowing, triangle	Music	Control of the environment
147	5A	B.4	2	K: Hello, I think the sound of the reading corner is really cool, because it's so quiet and...<br>P: Do you want to show them another sound?<br>K: I think so.<br>P: What do you think?<br>K: Something loud. Yes, something else loud.<br>P: Where is it always particularly loud in our kindergarten?<br>K: In the gym.<br>P: In the gym? Then you can still record it. And is it good for your ears when it's so loud or not?<br>K: No. I'll show them. goes into the gym "I like it in there ??? Perfect.	Quiet reading corner, gym loud.	Quiet, Noise	Control of the environment

#	Probe #	Group	Return no.	Probe data	Code	Themes	Meta theme expressions
148	5A	B.4	3	K: And like to sing.<br>P: Singing is good for you?<br>K: Mhm.<br>P: When we sing or when the radio sings?<br>K: When we sing.<br>P: When we sing, you like that?<br>K: And, when we are in the gym, it is always so loud and I don't like that. And... and I don't like anything else.	Singing, gym is loud	Songs, Noise	Control of the environment
149	5A	B.4	4	K: The Bayern song. sings a FC Bayern song.<br>P: Daniele's favorite sound or song is FC Bayern and he sang that to you now.<br>K: Yes. I like all the songs there.<br>P: You like all the FC Bayern songs? All the soccer songs?<br>K: Mhm. Can I still record what I don't like? I don't like the building corner.<br>P: You don't like the noise in the corner? You don't like the noise from the building corner?<br>K: No. And I like the doll corner, but I don't like it up there either. Now I've already finished.<br>P: Thank you. Then we'll send it up with the teleporter.	FC Bayern songs, don't like noise from building corner	Songs, Noise	Control of the environment
150	5A	B.4	5	K: I don't like the sound very much in the gym.<br>P: Is there any other sound that you would like to record? That you like or don't like so much?<br>K: Mhm. I like ironing beads building.<br>P: The sound? When you're building ironing beads, you're usually....<br>K: Quiet and I like that.<br>P: You like that when it's quiet. Good. Thank you in the meantime.	Gym noise, quiet ironing beads building.	Noise	Control of the environment
151	5A	B.5	1	K1: I don't like that sound (makes a noise) I don't like that ???<br>K2: I don't like that (makes a noise)	Unknown	Unknown	Control of the environment
152	5A	B.5	2	K: My sound is quiet.<br>P: Now I think you have to say if that's a good sound for your ears....<br>K: It's a good sound for my ears.<br>P: And you were in the reading corner now.	Quiet at reading corner	Quiet	Control of the environment
153	5A	B.5	3	P: You're up in the doll's corner now, and what sound do you like there?<br>K: Uhh... The kitchen.<br>P: Kitchen sound?<br>K: Yes.<br>P: What kind of noise does the kitchen make?<br>K: Uhh... I don't know.<br>P: Okay. Is there any other sound that you like or don't like for your ears? That you want to record?<br>K: Uhh... The dishes.<br>P: The dishes, when that rattles? Is that a good sound or a bad sound for your ears then?<br>K: Uhh... loud.<br>P: A loud sound.<br>K: Yes.<br>P: And is fine if it's loud or not?<br>K: No.<br>P: Good.	Kitchen sound, The dishes rattling are bad sounds	Noise	Control of the environment
154	5A	B.5	4	K: My sound is quiet.	Quiet	Quiet	Control of the environment
155	5A	B.5	5	the loud coming of children is recorded<br>P: Now was the sound good or bad?<br>K: Bad	Loud	Noise	Control of the environment
156	5B	Group A	1	"That's unfair"	Unfair	Unfair	Control of the environment
157	5B	Group A	2	"That's just sad"	Sad	Sadness	Control of the environment
158	5B	Group A	3	"If you have to stop, then we forget what we have to finish"	Fear of forgetting what they made	Activity disruption	Control of the environment
159	5B	Group A		"Then is to put things in order"	Realisation		Control of the environment
160	5B	Group A	5	"That you have to disassemble everything is not good"	Not good		Control of the environment
161	5B	Group A	6	"That's fully mean actually because when I do something cool I have to break it extra"	Mean		Control of the environment
162	5B	Group A	7	"That's mean that he disturbed us"	Mean		Control of the environment

#	Probe #	Group	Return no.	Probe data	Code	Themes	Meta theme expressions
163	5B	Group A	8	"-If I just built something, then I have to take it apart again quickly”	Built something and quickly dismantle	Unfair	Control of the environment, time making
164	5B	Group A	9	“Unfair”	Unfair	Unfair	Control of the environment
165	5B	Group A	10	“Unfair”	Unfair	Unfair	Control of the environment
166	5B	Group A	11	“Sad”	Sad	Unfair	Control of the environment
167	5B	Group A	12	“You are interrupted and don't know anymore what you wanted to paint/do”	Interruption disrupts activity	Unfair	Control of the environment
168	5B	Group A	13	"Dismantle everything - then I get sick, that's not good"	Not good	Unfair	Control of the environment
169	5B	Group A	14	“Very mean actually, first you make it and then you have to break it again"	Mean	Unfair	Control of the environment
170	5B	Group B	1	"Not nice"	not nice	Unfair	Control of the environment
171	5B	Group B	2	"Not good at all"	Not good	Unfair	Control of the environment
172	5B	Group B	3	"It's fine for me, I'm already bear-hungry"	Good	Good	Time-making
173	5B	Group B	4	"I think it's mean and unfair"	Mean and unfair	Unfair	Control of the environment
174	5B	Group B	5	"I could have just eaten that one"			
175	5B	Group B	6	"I don't like it, why does he have to say that?"	Dislike	Dislike	Control of the environment
176	5B	Group C	1	"clean up very quickly”	Cleaning up	Action	Control of the environment
177	5B	Group C	2	"Child was surprised”	Surprise	Dislike	Control of the environment
178	5B	Group C	3	"child was frightened => cleans up quickly”	Frightened start cleaning up	Action	Control of the environment
179	5B	Group C	4	Child is excited	Excited	Action	Control of the environment
180	5B	Group C	5	Child thinks nothing	Indifferent	Inaction	
181	5B	Group C	6	"Voice was cute before" - now child doesn't like the voice	Not nice	Dislike	Control of the environment
182	5B	Group C	7	Child thinks he gets to go into space	Random thought		
183	5B	Group C	8	"Why clean up?"	Why clean up	Reasoning	Control of the environment
184	5B	Group C	9	"Prick up ears"	Attentive	Action	
185	5B	Group D	1	" Abundantly, well"	Good	Preference	Control of the environment
186	5B	Group D	2	“That is mega gut”	Good	Preference	Control of the environment
187	5B	Group D	3	“We're happy when we're allowed to clean up“	Happy	Preference	Control of the environment
188	5B	Group D	4	“Mommy says it for me“	Authority		Control of the environment
189	5B	Group D	5	“I know myself when I have to tidy up“	Agency	Action	Control of the environment
190	5B	Group D	6	“But he had a fine voice“	Surprise	Unfair	
