# Supplementary material for: Uncovering placemaking needs with(in) a kindergarten community: a cross-disciplinary approach to participatory design
Source: Front Psychol. 2023 Jun 20;14:1126276. doi: 10.3389/fpsyg.2023.1126276 (PMC10319412; doi:10.3389/fpsyg.2023.1126276)
Supplement: Supplementary Data Sheet S8 — Codebook for content analysis of building evaluation. [file Data_Sheet_8.PDF]

CODEBOOK Spatial qualities for experience of place

Theme	Definition	Category	Description	Examples positive [+] | negative [–]	Spatial Quality
Embeddedness	need for orientation in the immediate surrounding, the relationship to the environmental context, and the feeling of local relatedness	Sensory Environment	factors that support the sensory experience and the affective ambience	“[group room] big window front, much natural light, view of green area [+][+]”, “[hall] hall very bright and big [+]”, “[entrance area] glass doors; bright, much light comes in [+]”, “[gym] much daylight [+]”, “[gym] Floor heating - the room can also be used barefoot. [+]”, “[creative area] in summer it is pleasantly cool [+]”, “[staff room] well to ventilate [+]”, “[rhythmics area] Large circular marking in the middle of the room [...] [+]”, “[gym] markings on the floor [+]”, “[group room] blue carpet, blue door → blue group [+]”<br>“[group room] cannot be darkened [–]”, “[group room] light; no daylight or rather no view of the outdoors [–][–]”, “[garden] not enough shadow places [–]”, “[wardrobe] bad lighting conditions, too little light [–]”, “[eating area] Cone (cold in winter, sauna in summer) [–]”, “[staff room] gets hot fast [–]”, “[group room] when airing, no fresh air comes in [–]”, “[gym] it is often stuffy [–]”, “[gym] in summer it is very hot (despite of sun protection) [–]”, “[rhythmics area] Poor acoustic conditions - noises from outside (corridor and adjoining group rooms) are very disturbing, especially with this room function. We cannot do silent exercises. [–]”, “[entrance area] without acoustic ceiling – very loud [–]”	Light, vision, orientation<br>- lighting conditions (natural lighting, brightness)<br>- light regulation (shading, darkening)<br>- visual support / orientation (colour code, shapes, view)<br>Climate, temperature, smell<br>- thermal conditions<br>- indoor air quality<br>Acoustics, noise, hearing<br>- acoustic condition (noise)<br>- sound moderation (insulation, arrangement)
		Natural Environment	factors that support the experience of flora and fauna	“[group room] big window front, much natural light, view of green area [+]”, “[garden] herbs, fruits, plants (snack garden) [+]”, “[group room] In this room there is an indoor plant and also animals (a terrarium with insects and plants) [...] [+][+]”, “[garden] snack garden for all children [+]”, “[garden] The children find the stones next to the outside walls very interesting and inviting. They take great pleasure in knocking the stones together, collecting them and building something with them below the outside stairs [+]”, “[staff room] natural building materials – lots of wood [+]”<br>“[garden] no trees [–]”, “[garden] [request] more playing options (e.g., ‘mud-water-area’, climbing frame, tree trunk) [–]”, “[garden] wooden terrace splinters [–]”, “[eating area] view from the window front into the grey - no green areas [–]”, “[group room] [...] less natural building materials / furnishings [–]”	nature perception<br>- plants, green space<br>- animals<br>- natural materials<br>interaction with nature<br>- nature education<br>- free play with natural materials or elements<br>- sensory / snack garden
		Contextual Environment	factors that support the experience of and relationship to the environmental context, neighbourhood, and socio-economic environment	“[entrance area] glass doors; bright, much light comes in [+]”, “[garden] somewhat shielded [+]”, “[garden] There is an asphalt area – children can play with wheels and bounce balls better here than on the green area. [+]”, “[garden] size [+]”, “[entrance area] very close parking lots for staff [+]”, “[garden] four access options – separately for each group (two external stairs for upper floor) [+]”, “[lavatory] In each lavatory there is - for each group separately - an exit to the outdoor area / play area (this is also used as an entrance during COVID times when parents bring and pick up their children to avoid crowds). [+][+]”, “[garden] new garden house [+]”, “[garden] now sufficient space and green area (was recently expanded) [+]”<br>“[group room] light; no daylight or rather no view of the outdoors [–]”, “[entrance area] no option for parking bicycles [–]”, “[entrance area] safety concerns because there is a thoroughfare in front of the kindergarten, no separation (children cannot gather in front of the kindergarten - too close to the street) [–][–]”, “[entrance area] should be more open [–]”, “[hall] too open [–]”, “[entrance area] too small [–]”, “[building] too many windows for me [–]”, “[garden] Danger of roof avalanches in winter! Parts of the garden are often closed. [–]”	Contact to the contextual environment<br>- neighbourhood and infrastructure<br>- access points (entrance/exit)<br>- transition areas (space for arrival/departure)<br>- delineation of territory (building and grounds)<br>- view of the contextual environment (outdoors)

Theme	Definition	Category	Description	Examples positive [+] | negative [–]	Spatial Quality
Protectedness	need for maintenance of health and physical integrity, the prevention of disease and injuries, and the sense of security	Safety	factors that promote safety behaviour, physical integrity, and the feeling of safety and security	"[group room] [...] the children feel hidden in the place of refuge, but the places of refuge are still clearly visible for the employees. [+]", "[garden] covered terrace - offers good shade and rain protection [+]", "[garden] large parasol over sandbox [+]", "[eating area] highchairs for younger children [+]", "[garden] somewhat shielded [+]"<br><br>"[garden] We welcome that the children build with the stones, but the space below the outside stairway is unfavourable, it is difficult to control for the employees – this can be dangerous. [–]", "[entrance area] safety concerns because there is a thoroughfare in front of the kindergarten, no separation (children cannot gather in front of the kindergarten - too close to the street) [–]", "[garden] few seating options → those we have, have to be sanded down every year, otherwise the children get splinters stuck in their hands or elsewhere [–]", "[gym] The roof is leaky when it rains → danger of slipping! [–]", "[group room] too many opportunities to bump your head [–]", "[garden] little sun protection [–]", "[entrance area] no door opener in height where children cannot reach it; kids can just get out [–]"	<u>Risk prevention</u><br>- physical separation, barriers, and delineation of children's scope of action<br>- visibility and accessibility of children for staff<br><u>Injury prevention</u><br>- building maintenance<br>- user-appropriate scales and proportions<br><u>Protection</u><br>- weather protection<br>- sight protection
		Hygiene	factors that promote hygiene behaviour and cleanliness	"[lavatory] well accessible for children [+]", "[lavatory] lavatory alright! [+]", "[lavatory] Each child has an own mug at the sinks in the lavatory, there is sufficient space and storage area. [+][+]", "[lavatory] In each lavatory there is - for each group separately – [...] an entrance during COVID times when parents bring and pick up their children to avoid crowds). [+]", "[garden] on the outer walls there are hooks for clothes and backpacks [+]", "[eating area] Floor material - if crockery (ceramics) falls, it doesn't break. [+]", "[staff cloakroom] lockers [+]", "[garden] water hose [+]"<br><br>"[lavatory] too few toilets, [...] there are jams in the lavatories [...] [–]", "[lavatory] sinks too large → soap for children not reachable despite stage [–]", "[lavatory] no options to dry laundry [–]", "[lavatory] too far away from the group room [–]", "[eating area] ants!! [–]", "[staff cloakroom] far away from the entrance area [–]", "[entrance area] [request] furniture for shoe-free kindergarten (e.g., seating, storage space for shoes, wardrobe) [–]", "[staff cloakroom] too little space for shoes or in winter with the jackets [–]", "[staff room] [request] storage for dishes [–]"	<u>Cleanliness</u><br>- adequate location and furniture of wardrobes<br>- laundry options<br>- easy accessibility of lavatories for children<br>- general cleanliness (kitchen, eating areas, lavatories, etc.)<br><u>Disease prevention</u><br>- socio-spatial separation<br>- availability and accessibility of hygiene equipment<br>- separate dishes (drinking beakers)
		Well-being	factors that promote healthy behaviour and resilience	"[gym] The gym offers enough space for play and movement. [+]", "[group room] proximity to the gym [+]", "[wardrobe] very good location, directly in front of the respective group room (can also be used as a place of refuge and still be easily observed) [+][+]", "[garden] terrace with hammocks in the shadow [+]", "[group room] kitchenette [+]", "[wardrobe] the benches are very good for the children to sit on [+]", "[staff room] tables and seats for adults available [+]", "[creative area] in summer it is pleasantly cool [+]", "[staff room] kitchen is well equipped, microwave [+]", "[staff toilet] quotes for motivation on the wall [positive]"<br><br>"[gym] much too small [–]", "[gym] there are no climbing options for children [–]", "[gym] extremely hot + stuffy [–][–]", "[dormitory] lack of space – too small, too narrow for several reclining options [–]", "[staff room] [...] there is no enclosed retreat for employees. [–]", "[staff room] noise level during the lunch break [–]", "[wardrobe] Too little space, too narrow, not all children can remain seated when dressed (that would be desirable, facilitates the work for employees). [–]", "[eating area] large tables with lots of children --> unrest; for lunch it would be better to have smaller tables where fewer children have space (quieter when eating) [–]", "[eating area] way too small so very impractical [–]", "[lavatory] [...] there are jams in the lavatories, which creates stressful situations. [–]", "[staff toilet] too far away - long way [–]"	<u>Physical well-being, health prevention</u><br>- options for physical activity<br>- recreational options<br>- ergonomics of furniture<br>- comfortable ambience<br><u>Physical well-being, stress prevention, job satisfaction</u><br>- resource-friendly space (proximity, accessibility, general usability)<br>- adequate socio-spatial arrangement (focus, communication, rest)<br>- decoration (attractivity)

Theme	Definition	Category	Description	Examples positive [+] | negative [–]	Spatial Quality
Connectedness	need for favourable social experiences, and the feeling of social relatedness	Belonging	factors that promote a sense of belonging, group identity and group privacy	“[group room] blue carpet, blue door → blue group [+]”, “[group room] Group rooms are enclosed areas [...], each group can be undisturbed. [+]”, “[group room] Large circle-shaped carpet (group’s colour) in the middle of the room [...] - here occur group play / exercises.[+]”, “[wardrobe] own place for each child (here are also personal things, e.g. the cuddly toy) [+][+]”, “[rhythmics area] Large circular marking in the middle of the room [...] assembly point; the size of the circle is sufficient for 25 children and it is an important socially connecting spatial element. [+][+]”, “[group room] [...] every year the group rooms are redesigned and both employees and children look forward to it [...] [+]”, “[group room] All group rooms provide sufficient design options. [+]”, “[wardrobe] separate cloakroom with door (so not in the open entrance hall); for AEG / toddler group very positive [+]”<br><br>“[group room] too small [–]”, “[group room] [...] poor acoustic shielding from outside (other groups are audible) [–]”, “[eating area] Far too little space, narrow space! A single group must be split up to have a snack. [...]. [–]”, “[staff cloakroom] [request] own compartment for every teacher [–]”	Inclusiveness of space<br>- homebase / assembly areas<br>- group size – space ratio<br>- common representation and expression of group identity (colour scheme, decoration)<br>- space for personal objects<br>Exclusiveness of space<br>- physical separation and differentiation from other groups (spatial, visual, acoustic) – refuge for groups
		Interaction	factors that foster social experiences with peer group members, and privacy of individuals	“[creative area] table groups – socially connecting [+]”, “[group room] Each group room provides a refuge for children (the Blue and Green rooms have a scaffolding – these are particularly popular; the Yellow and Red rooms have refuges / tents built on the ground - partly temporary); [...]. [+]”<br><br>“[wardrobe] the benches are separated, so it is difficult to discuss things [...], if not all the children are sitting together [–]”, “[staff room] Room for meetings too small, almost no space for all staff [–]”, “[garden] Insufficient equipment – [...] they cannot all play at the same time (only 1 slide, 1 swing, etc.). There are jams in the play areas. [...] [–]”, “[staff room] The room is an open combination with the kitchen where food is also served (kitchen in the entrance area, eating area for children in front of the room) - there is no enclosed retreat for employees. [–]”, “[group room] [request] better separation in the group rooms [–]”, “[group room] noise from the gym disturbs during quiet activities [–]”, “[resting area] [...] window overlooking the play area - children who want to sleep are distracted or disturbed. [–]”	Inclusiveness of space<br>- availability of sufficient space and equipment<br>- socio-spatial arrangement that supports interaction<br>Exclusiveness of space<br>- availability of refuge areas for individuals<br>- separation of “quiet/resting areas” from “loud/activity areas” (spatial, visual, acoustic)
		Community	factors that delineate a general social structure, and promote a shared culture and a sense of community	“[rhythmics area] sliding doors to the hall (for events) [+]”, “[garden] snack garden for all children [+]”, “[garden] somewhat shielded [+]”, “[staff toilet] staff toilet next to children’s lavatory [+]”, “[garden] four access options – separately for each group [...] [+]”, ”[entrance hall] is open [+]”, “[entrance area] very close parking lots for staff [+]”, “[staff room] tables and seats for adults available [+]”<br><br>“[garden] [...] too small when 100 children are outside [–]”, “[eating area] The room is very narrow with sloping ceilings - not enough space for adults / employees, a "labyrinth" with many obstacles. [–]”, “[eating area] very narrow, when all children eat together [–]”, “[gym] The gym has two large round windows onto the corridor. It happens that parents are waiting outside and watching or other children are knocking on the windows from outside; this distracts the children and sometimes disturbs the employees at work. [–][–]”, “[entrance area] long, dark corridor, without windows, without design options (except for pictures on the wall) [–]”, “[entrance area] locking system of the door bad [...] [–]”, “[staff cloakroom] too few lockers (6 pc for 14 employees) [–]”, “[gym] children find it difficult to open the door to the hall [–]”, “[entrance area] too small for "shoe-free kindergarten" (pursued goal!), shoes should already be taken off before entering the kindergarten areas [–]”	Inclusiveness of space<br>- common areas<br>- enough and appropriate space and equipment for all community members<br>- common representation of the community<br>Exclusiveness of space<br>- separation from the neighbourhood / context<br>- exclusive access to building<br>- social transition areas (exclusive space vs. shared space, space for parents)

Theme	Definition	Category	Description	Examples positive [+] | negative [–]	Spatial Quality
Enactedness	need for self-directed activity, the feeling of autonomy, self-determination, and competence	Staff	factors that support teaching, caregiving, and other work-related activities	"[creative area] well equipped with handicraft and painting materials [+]", "[creative area] the material is at hand [+]", "[gym] markings on the floor [+]", "[gym] proximity to the group room [+]", "[group room] kitchenette [+]", "[rhythmics area] versatile usable [+]", "[rhythmics area] the size [+]", "[rhythmics area] access to terrace & garden [+]", "[office] two computers [+]", "[lavatory] size of toilets (adult's size) → built stages + toilet seat → it is fine like this [+]", "[wardrobe] plenty of space for employees and children to create [+]", "[group room] Furniture flexibly adjustable, there is only one permanently installed element, and the room is easy to design [...] [+]", "[group room] [...] the staff decides for themselves, whether and how many tables they need or want to use - top priority: flexibility and space for design. [+]"<br><br>"[group room] It is not possible to completely shade the group rooms - this would be desirable not only for better temperature regulation but also for certain games/ exercises and for resting periods. [–][–]", "[group room] too small [–]", "[group room] [request] if we could divide the group rooms better [–]", "[group room] 'fixed constructing area' [–]", "[creative area] painting room = storage room :-([...] [–]", "[gym] few equipment for setting up an obstacle course [–]", "[garden] There is no storage space for toy vehicles. The vehicles are currently parked at the ground floor access [...] next to the outside wall - this is perceived as hindering. [–]", "[rhythmics area] Poor acoustic conditions - noise from outside (corridor and adjoining group rooms) are very disturbing, especially with this room function. We cannot do silent exercises. [–]", "[creative area] too few options for room design [...] [–]", "[creative area] [..] material cannot be specifically positioned and presented by the staff [...] There is no child-appropriate presentation area. [–]", "[eating area] [request] Smaller tables - but more would be great! When there is a long table, there is always unrest. [–]", "[office] [request] place for preparation would be very pleasant [–]", "[building] no alternative rooms for additional activities :-([–]", "[rhythmics area] too little storage space for instruments and games [–]", "[lavatory] too small (no possibility to set up the drying rack) [negative]", "[gym] no cabinets [–]"	Classroom management<br>- spatial options, adaptability, multifunctionality for flexible delineation of activity areas<br>- adaptable ambience<br>- adaptable socio-spatial arrangement (teacher-class interaction, children's social dynamics)<br>- space and equipment for presentation<br>- open and adaptable space, and material for classroom design and decoration<br><br>Caregiving<br>- space and equipment for caregiving and supporting children's autonomy<br><br>Organisation<br>- spatial efficiency, functional space and sufficient equipment (time-resource management)<br>- storage space<br>- space and equipment for preparation
		Children	factors that promote self-directed activities and decision-making of children	"[group room] Each group room provides a refuge for children [...] scaffolding – these are particularly popular; [...] children feel hidden [...]. [+]", "[garden] There is an asphalt area - children can play with wheels and bounce balls better here than on the green area. [+]", "[garden] shading at the sandbox [+]", "[creative area] many work places for children [+]", "[gym] high ceiling [+]", "[garden] a lot of sand toys [+]", "[garden] new playing house [+]", "[garden] open space and playground equipment [+][+]", "[gym] little or no furniture allows free play [+]", "[garden] 'Snack garden' – [...], etc. at a child-appropriate height, the children can help themselves. [+]", "[group room] Open shelving systems at child-appropriate height are room elements that promote creativity – they invite children to play and to be creative (keyword: presentation).[+]", "[rhythmics area] [...] is available to children for open use. [+]"<br><br>"[creative area] [..] material is not freely accessible to children [...] There is no child-appropriate presentation area. [–]", "[garden] more playing options are lacking (e.g. mud area, water area, marble run, climbing frame) [–]", "[gym] children find it difficult to open the door to the hall [–]", "[lavatory] distance to the group room is too far (not practical) [–]", "[rhythmics area] Poor presentation of instruments and games - the materials should be visible for the children and invite them to play and try out. [–]", "[garden] no climbing options [–]", "[resting area] [...] window overlooking the play area - children who want to sleep are distracted or disturbed. [–]"	Stimuli and motivators<br>- visibility and attractivity of space and materials<br>- appropriate ambience for different activities<br>- clearly recognizable activity areas / scope of action<br><br>Empowerment<br>- child-appropriate heights, scales and proportions (accessibility, applicability)<br>- open space<br>- options (space, equipment, materials)

Coding rules

#	Coding rule	Example
1	Each complete response (i.e., word, phrase, sentence) is associated with (a) a functional area and (b) a quality of response content (see below). The analysis of the response relates to this contextual information.	“**[rhythmics area]** Poor acoustic conditions - noises from outside (corridor and adjoining group rooms) are very disturbing, especially with this room function. We cannot do silent exercises. **[–]**”<br>“**[wardrobe]** very good location, directly in front of the respective group room (can also be used as a place of refuge and still be easily observed) **[+]**”
2	Each response forms an analysis unit with at least one semantic content. Each semantic content that relates either to (c) the perception of the built environment, or (d) the action within the built environment, or (e) the interaction with the built environment, is eligible for analysis.	“[rhythmics area] Poor acoustic conditions - noises from outside (corridor and adjoining group rooms) are very disturbing, especially with this room function. We cannot do silent exercises. [–]”<br>“[wardrobe] very good location, directly in front of the respective group room (can also be used as a place of refuge and still be easily observed) [+]”
3	Any quality of space associated with the built environment can serve different needs, and thus, elicit a different quality of place experience. Also, each analysis unit (response) can reflect more than one semantic content. Create (f) a duplicate unit (response) for each additional interpretation that is (g) clearly assignable to one category.	Theme: **embeddedness** | Category: **sensory environment**<br>“[rhythmics area] Poor acoustic conditions - noises from outside (corridor and adjoining group rooms) are very disturbing, especially with this room function. We cannot do silent exercises. [–]”<br>Theme: **enactedness** | Category: **teacher**<br>“[rhythmics area] Poor acoustic conditions - noises from outside (corridor and adjoining group rooms) are very disturbing, especially with this room function. We cannot do silent exercises. [–]”
4	In case an analysis unit contains more than one clearly differentiable content assignable to the same category, create (h) duplicate markings for each additional interpretation (content weighting). *	Theme: **connectedness** | Category: **belonging**<br>“[wardrobe] own place for each child (here are also personal things, e.g., the cuddly toy) [+] [**+**]”<br>Theme: **enactedness** | Category: **children**<br>“[wardrobe] own place for each child (here are also personal things, e.g., the cuddly toy) [+] [**+**]”

Note: * In the present analysis, rule #4 applied to only a minimum of cases. The content was differentiable regarding the spatial quality and the related experience.

Functional areas

Activity areas	creative area, gym, garden/outdoor play area, resting area, rhythmics area
Common areas	eating area, entrance area, auditorium/hall, building in general, areas for parents
Homebase areas	group rooms, group wardrobes, group lavatories, group entrance/exits
Staff areas	office, staff room, staff cloakroom, staff toilet

Quality of content

positive	beneficial for teachers and/or children
negative	hindering for teachers and/or children
