# Supplementary material for: Uncovering placemaking needs with(in) a kindergarten community: a cross-disciplinary approach to participatory design
Source: Front Psychol. 2023 Jun 20;14:1126276. doi: 10.3389/fpsyg.2023.1126276 (PMC10319412; doi:10.3389/fpsyg.2023.1126276)
Supplement: Supplementary Data Sheet S9 — Joint analysis and cross-disciplinary research consolidation. [file Data_Sheet_9.PDF]

JOINT ANALYSIS Placemaking qualities for children and staff in the built kindergarten environment

**Placemaking qualities**	**Examples from children** (C: child, T: teacher)	**Examples from staff** (H: headmistress, T: teacher)	**Description**
**Space**	spatial qualities that influence the perception of space, and the interaction within space and with space		
**Availability**	#1B - Available crafting and construction equipment.<br>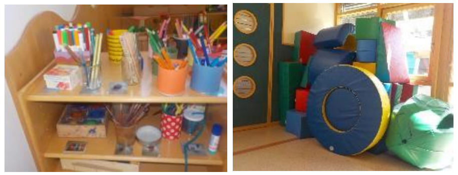<br>#4* - Available physical space and proportions related to it.<br>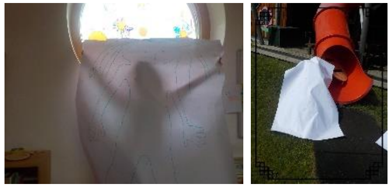	H: “[wardrobe] own place for each child (here are also personal things, e.g., the cuddly toy) [+]”, “[gym] the gym offers enough space for play and movement [+]”, “[garden] There is an asphalt area - children can play with wheels and bounce balls better here than on the green area. [+], “[garden] The asphalt area is too small for certain games or activities. [–]”, “[eating area] Far too little space, narrow space! A single group must be split up to have a snack. [...] [–]”, “[rhythmics area] too little storage space for instruments and games [–]”<br>T: “[gym] high ceiling [+]”, “[creative area] well equipped with handicraft and painting materials [+]”, “[staff room] kitchen well equipped, microwave [+]”, “[garden] a lot of sand toys [+], “[group room] too small [–]”, “[staff room] Room for meetings too small, almost no space for all staff [–]”, “[office] [request] space for preparation would be very pleasant [–]”, “[garden] [request] more playing options (e.g., “mud-water-area”, climbing frame, tree trunk) [–]”, “[garden] few seating options [...] [–]”, “[staff cloakroom] [request] own compartment for every teacher [–]”	basic space requirement, and quantity of available physical space and equipment
**Accessibility**	#1B - Reachable shelving units, materials at children’s height, furniture proportional to children.<br>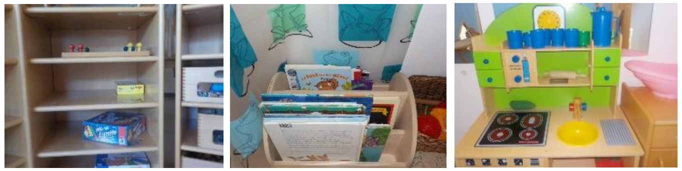	H: “[garden] four access options – separately for each group (two external stairs for upper floor) [+], “[group room] [...] the children feel hidden in the place of refuge, but the places of refuge are still clearly visible for the employees. [+]”, “[garden] ’snack garden’- on the fences there are plant boxes with strawberries, currants, etc. at a child-appropriate height, the children can help themselves [+]”, “[entrance area] locking system of the door bad (safety for children) [...] [–]”, “[staff room] [...] - there is no enclosed retreat for employees. [–]”<br>T: “[rhythmics area] access to terrace and garden [+]”, “[group room] blue carpet, blue door → blue group [+]”, “[lavatory] well accessible for children [+]”, “[gym] children find it difficult to open the door to the hall [–]”, “[wardrobe] separate cloakroom with door (so not in the open entrance hall); for AEG / toddler group very positive [+]”, “no door opener at a height where children cannot reach it; kids can just get out [–]”	physical delineation of people’s scope of action and orientation
**Arrangement**	#1B - Storage area next to play area, play area dictated by carpet with storage underneath. Circular carpet delineates central activity taking place currently.<br>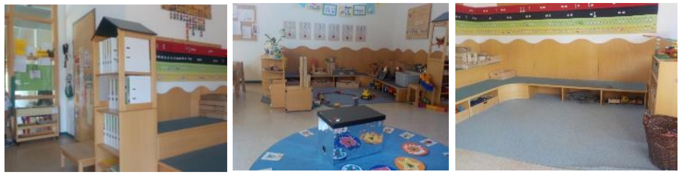	H: “[group room] Large circular carpet (group’s colour) in the middle of the room [...] here occur group play/exercises. [+]”, “[wardrobe] very good location, directly in front of the respective group room (can also be used as a place of refuge and still be easily observed) [+], “[eating area] The eating area is an open space - noise from all sides. [–]”, “[dormitory] [...] noise level from adjoining rooms - children who want to sleep are disturbed [–]”<br>T: “[gym] circle on the floor [+]”, “[wardrobe] the location of the wardrobes (in front of the group room) is very good [+]”, “[gym] proximity to the group room [+]”, “[wardrobe] the benches are separate, so it is difficult to discuss things in the wardrobe area, since not all the children are sitting together [–]”, “[creative area] it is a ‘passage room’ [–]”, “[creative room] no separation in the room for handicraft materials [–]”, “[entrance area] should be more open [–]”	spatial layout, furniture arrangement, and the functional delineation of space

**Placemaking qualities**	**Examples from children** (C: child, T: teacher)	**Examples from staff** (H: headmistress, T: teacher)	**Description**
**Space**	spatial qualities that influence the perception of space, and the interaction within space and with space		
**Applicability**	#1B - Reachable furniture and equipment at children's height and based on their scale and body proportions 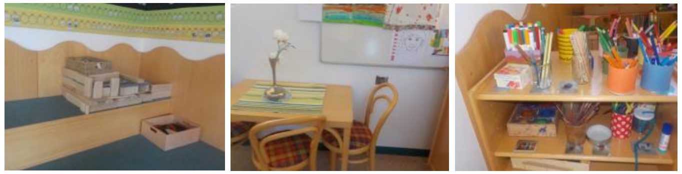	H: "[staff room] tables and seats for adults available [+]", "[group room] The height of the scaffolding is a little bit too low – the staff often bump their heads. [–]", "[rhythmics area] Poor acoustic conditions - noises from outside (corridor and adjoining group rooms) are very disturbing, especially with this room function. We cannot do silent exercises. [–]", "[creative area] The creative material is not freely accessible to children and cannot be specifically positioned and presented by the staff [...]. There is no child-appropriate presentation area. [–]"<br><br>T: "[eating area] highchairs for younger children [+]", "[creative area] painting room = storage room :-(→ for the materials → for the individual groups [–]", "[group room] too many opportunities to bump your head [–]", "[lavatory] sink + soap dispenser not accessible for small children, despite stage [–]", "[staff room] [request] the kitchenette should be adapted to the purpose [–]"	ergonomics and general functionality of space
**Adaptability**	#1B - Space is flexible and can be modified according to each activity taking place 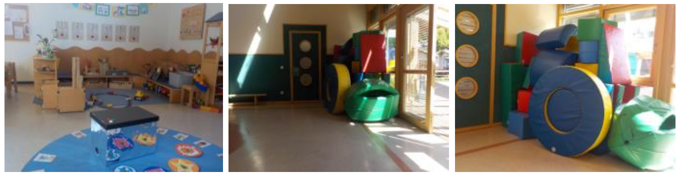	H: "[group room] [...] top priority: flexibility and space for design [+]", "[group room] Furniture flexibly adjustable, there is only one permanently installed element, and the room is easy to design [...] [+]", "[rhythmic area] This room is not only used as a classic rhythm room, but also for dance and movement and is available to the children for open use. [+]"<br><br>T: "[rhythmics area] sliding doors to the hall (for events) [+]", "[rhythmics area] versatile usable [+]", "[group room] 'fixed constructing area' [–]", "[building] no alternative rooms for additional activities :-([–]"	alternative space, and adaptability of space according to situational requirements
**Attractivity**	#1B - Decorations of windows and walls with children-made drawings, visible available material at a convenient height, places of refuge, solitude, and quiet 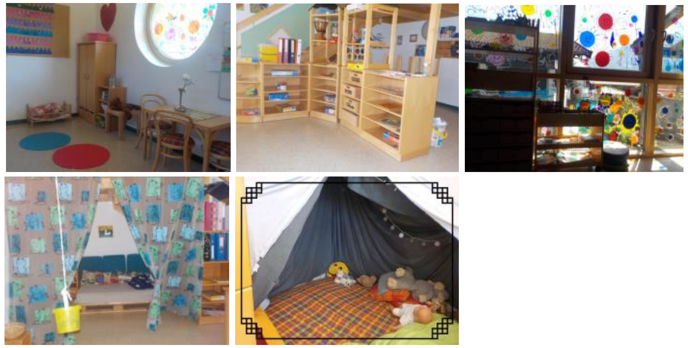	H: "[group room] [...] indoor plant and also animals (a terrarium with insects and plants) – these elements are particularly appreciated by the employees – the children especially like the animals [+]", "[group room] open shelving system at child-appropriate height [...] – they invite children to play and try out and to be creative [...] [+]", "[group room] the refuge for children is an invitingly designed tent on the ground. [+]", "[garden] The children find the stones next to the outside walls very interesting and inviting. They take great pleasure in knocking the stones together, collecting them and building something with them [...] [+]", "[eating area] overall, not very inviting to have a snack [–]", "[eating area] view from the window into the grey – no green areas [–]"<br><br>T: "[creative area] in summer it is pleasantly cool [+]", "[garden] terrace with hammocks in the shadow [+]", "[garden] hanging chairs [+]", "[staff toilet] quotes for motivation on the wall [+]", "[office room] bright and friendly [+]", "[gym] bad air – stuffy [–]", "[group room] light; no daylight or rather no view of the outside [–]", "[staff room] much too hot or too cold [–]", "[building] too many windows for me [–]", "[staff room] noise level during the lunch break [–]"	aesthetics of space, and spatial aspects of convenience, comfort and well-being

Placemaking qualities	Examples from children (C: child, T: teacher)	Examples from staff (H: headmistress, T: teacher)	Description
**Time**	spatial qualities that influence the perception of time flow, and time quality		
**Continuity**	#1B - A continuity of activities indoors and outdoors. Views of inside environments looking out of windows and allowing for observation of activities in the garden area.<br>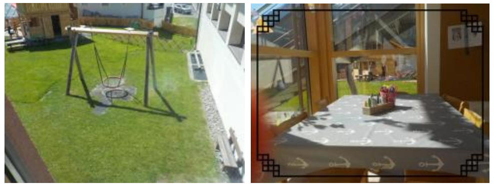<br>#5B - Reactions to the cease of activities<br>C: "We're happy when we're allowed to tidy up"<br>C: "You get interrupted and then you don't know what you wanted to do"<br>C: "Dismantle everything - then I feel sick, that's not good"	H: "[group rooms] Group rooms are enclosed areas [...] each group can be undisturbed. [+]", "[dormitory] Unfavourable location of the room [...] window overlooking the play area - children who want to sleep are distracted or disturbed. [–]", "[garden] Insufficient equipment - with currently 100 children, theoretically all children can go into the garden at the same time, but they cannot all play at the same time (only 1 slide, 1 swing, etc.). There are jams in the play areas. [...] [–]", "[gym] The gym has two large round windows onto the corridor. It happens that parents are waiting outside and watching or other children are knocking on the windows from outside; this distracts the children and sometimes disturbs the employees at work. [–]"<br>T: "[group room] noises from the gym disturb during quiet activities [–]", "[staff room] children's kitchen for lunch and staff room for breaks are all one; [...] no rest during the break [–]", "[staff room] no undisturbed lunch breaks possible [–]"	aspects of flow and disruption
**Efficiency**	#1B – Available storage space and organisation of materials based on colour coding and symbols<br>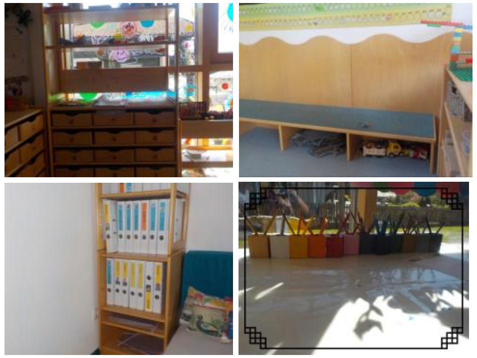	H: "[entrance area] very close parking lots for staff [+]", "[eating area] Floor material – if crockery (ceramics) falls, it doesn't break. [+]", "[garden] There is no storage space for toy vehicles. The vehicles are currently parked at the ground floor access next to the outside wall – this is perceived as hindering. [–]", "[wardrobe] Too little space, too narrow, not all children can remain seated when dressed (that would be desirable, facilitates the work for employees) [–]", "[staff cloakroom] far too small, too little space [...] several employees cannot change at the same time [–]", "[creative area] Overall, the room appears very chaotic and unsystematic, there is no systematic – possibly separated – storage space.[–]"<br>T: "[creative area] the material is at hand [+]", "[gym] proximity to group room [+], "[lavatory] distance to the group room is too far (not practical) [–]", "[staff toilet] too far away – long way [–]", "[staff cloakroom] farther away from the entrance area; [request] would be better right next to the entrance [–]", "[garden] vehicles (e.g. impellers) no parking space (just lying around) [–]", "[wardrobe] the benches are separate, so it is difficult to discuss things in the wardrobe area, since not all the children are sitting together [–]", "[eating area] way too small so very impractical [–]"	timesaving and practical aspects

**Placemaking qualities**	**Examples from children** (C: child, T: teacher)	**Examples from staff** (H: headmistress, T: teacher)	**Description**
**Control**	opportunities to change or preserve the qualities of space according to situational needs		
**Sensory environment**	#1B – Qualities of space related to noise, light conditions and affordance possibilities. Places of refuge, flexibility of changing space arrangements.<br>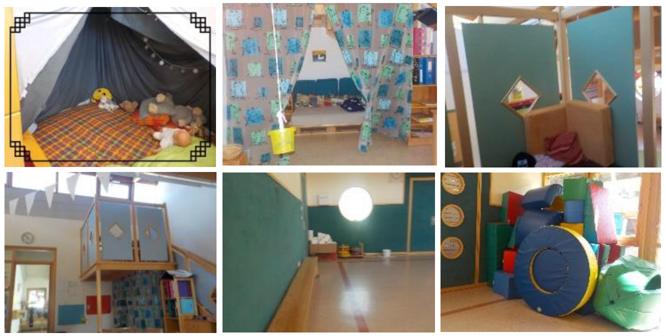<br>#3* - Crafting smart materials that alter spatial atmospheric qualities<br>T: “What have you made?”<br>C: ”There’s a straw with a sheet of paper and I glued it shut, and if it’s too hot for you, you can blow into the straw and it will cool you down again.”<br>#5A – Inquiring favourite and disliked sounds<br>C: “I don’t like anything loud”<br>K: “What sounds would you like to record for the children of Harkarosh?”<br>C: “Uh, the ones I don’t like very much.”<br>K: “That you don’t like? And what sounds are those?”<br>C. “Shouting”<br>K: “Screaming?”<br>C: “Yes. I don’t like that.”<br>K: “And you don’t like it so much when it’s so loud in the gym?”<br>C: “No”.	H: “[group room] It is not possible to completely shade the group rooms – this would be desirable not only for better temperature regulation but also for certain games / exercises and for resting periods. [–]”, “[rhythmic area] Poor acoustic conditions – noises from outside (corridor and adjoining group rooms) are very disturbing, especially with this room function. We cannot do silent exercises. [–]”, “[staff room] temperature not sufficiently adjustable – seasonal extremes, especially in summer much too hot (+30°C) [–]”, “[wardrobe] bad air quality, wardrobes are difficult to ventilate [–]”, “[entrance area] indoor area: artificial lighting unpleasant – mostly switched off [–]”, “[gym] The gym has two large round windows onto the corridor. It happens that parents are waiting outside and watching or other children are knocking on the windows from outside; this distracts the children and sometimes disturbs the employees at work. [–]”<br>T: “[garden] large parasol over sandbox [+], “[group room] cannot be darkened [–]”, “[group room] when airing, no fresh air comes in [–]”, “[staff room] very hot in summer, despite of sun protection [–]”, [group room] noise from the gym disturbs during quiet activities [–]”, “[staff room] noise level during the lunch break [–]”	control of the affective atmosphere and the sensory qualities of space

**Placemaking qualities**	**Examples from children** (C: child, T: teacher)	**Examples from staff** (H: headmistress, T: teacher)	**Description**
**Control**	opportunities to change or preserve the qualities of space according to situational needs		
**Physical environment**	#1B – Placemaking and creation of dens and other refuge areas using available materials like cardboard, fabric tents and mattresses.<br>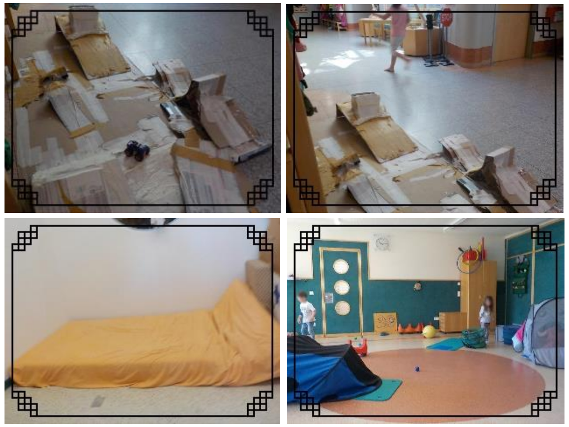	H: "[group room] [...] the staff decides for themselves, whether and how many tables they need or want to use - top priority: flexibility and space for design. [+]", "[group room] [...] every year the group rooms are redesigned and both employees and children look forward to this [...] [+]", "[group room] [...] tents built on the ground - partly temporary [...] [+]", "[creative area] The creative material is not freely accessible to children and cannot be specifically positioned and presented by the staff in such a way that the children are invited to creative activities. [...] [–]", "[garden] We welcome that the children build with the stones, but the space below the outside stairway is unfavourable, it is difficult to control for the employees – can be dangerous. [–]"<br>T: "[garden] redesign of garden house and sandpit [+]", "[group room] [request] if we could divide the group rooms better [–]", "[gym] few equipment for setting up an obstacle course [–]","[entrance area] long, dark corridor, without windows, without design options (except for pictures on the wall) [–]", "[lavatory] size of toilets (adult's toilet); built stages and a toilet seat now it's fine [–]", "[garden] few seating options → those we have, have to be sanded down every year otherwise the children get splinters stuck in their hands or elsewhere [–]", "[garden] Danger of roof avalanches in winter! Parts of the garden are often closed. [–]"	control of the visual appearance and the arrangement of space
**Social environment**	#1B - Social area as a favourite place. Walls and rooms decorated with crafted artefacts by each child put together, sense of belongingness in a community.<br>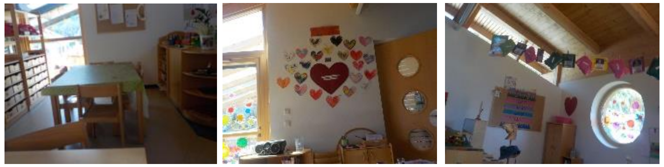	H: "[group room] [...] circular marking in the middle of the room - optical feature for the assembly point [...]. [+]", "[lavatory] [...] for each group separately - an exit to the outdoor area/play area (this is also used as an entrance during COVID times when parents bring and pick up their children to avoid crowds).[+]", "[group room] [...] the Blue and Green rooms have a scaffolding – these are particularly popular; the Yellow and Red rooms have refuges/tents [...]; the children feel hidden [...] but the places of refuge are still clearly visible for the employees. [+]", "[entrance area] too small for 'shoe-free kindergarten' (pursued goal!), shoes should be taken off already before entering the kindergarten area [–]"<br>T: "[group room] blue carpet, blue door --> blue group [+]", "[eating area] large tables with lots of children → unrest; for lunch it would be better to have smaller tables where fewer children have space (quieter when eating) [–]", "[entrance area] no door opener at a height where children cannot reach it; kids can just get out [–]", "[garden] confusing / too small when 100 children are outside [–]", "[staff room] [...] eating areas are not separated (even during the break children are in the room or the lunch service ladies are always in the room) [–]"	control of socio-spatial aspects that influence social interaction, behaviours, and dynamics

Note: For examples from children: #1B= Data derived from Probe 1: Photo elicitation of favourite places and play activities. #3*= Data derived from Probe 3: Crafting of Material Samples for play using provided crafting materials. #4*= Data derived from Probe 4: Drawing 1:1 Body outlines on large pieces of paper and placement of the paper at favourite places. #5A= Data derived from Probe 5: Audio-recordings and descriptions of favourite and least favourite sounds. #5B = Data derived from Probe 5: Cease of activity and reactions to it. *Not included in the first round of analysis. For examples from adults: markings [+] and [–] indicate the positive or negative quality of the response content as described by the respondents, no weighting information was added.
